# Mechanical Activation of Zero-Valent Metal Reductants for Nickel-Catalyzed Cross-Electrophile Coupling

**DOI:** 10.1021/acscatal.2c03117

**Published:** 2022-10-25

**Authors:** Andrew C. Jones, Matthew T. J. Williams, Louis C. Morrill, Duncan L. Browne

**Affiliations:** † Cardiff Catalysis Institute, School of Chemistry, Cardiff University, Main Building, Park Place, Cardiff, CF10 3AT, U.K.; ‡ School of Pharmacy, University College London, 29-39 Brunswick Square, Bloomsbury, London, WC1N 1AX, U.K.

**Keywords:** cross-coupling, nickel catalysis, mechanochemistry, ball-milling, solvent free

## Abstract

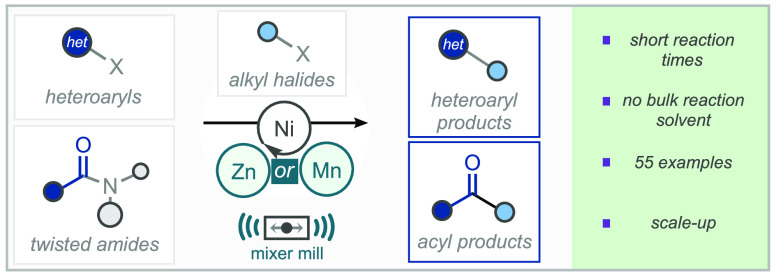

The cross-electrophile coupling of either twisted-amides or heteroaryl halides with alkyl halides, enabled by ball-milling, is herein described. The operationally simple nickel-catalyzed process has no requirement for inert atmosphere or dry solvents and delivers the corresponding acylated or heteroarylated products across a broad range of substrates. Key to negating the necessity of inert reaction conditions is the mechanical activation of the raw metal terminal reductant: manganese in the case of twisted amides and zinc for heteroaryl halides.

## Introduction

**Scheme 1:**
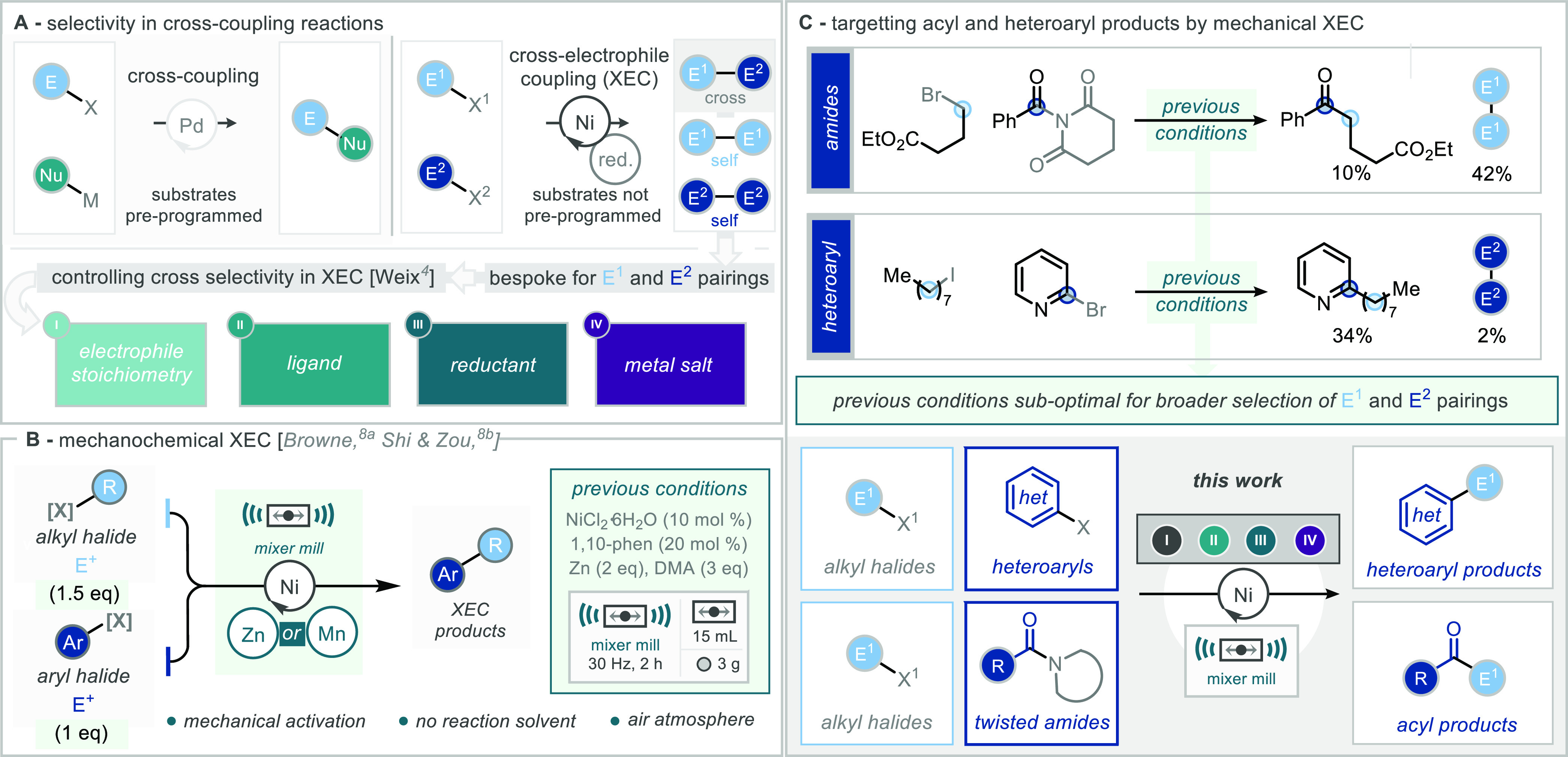
Cross-Electrophile Coupling. (A) Overview. (B) Prior Conditions Developed for Ball-Mill XEC. (C) This Work: Heteroaryl Products and Acyl Products

Over the last half-century, transition-metal-catalyzed cross-coupling reactions have been delivered from proof of concept to a staple of organic synthesis, celebrated in 2010 with the award of the Nobel prize to Heck, Suzuki, and Negishi, for the development of palladium catalyzed methods.^1^ The development of these processes has required many key breakthroughs from the community to deliver a versatile methodology platform capable of permitting the forging of C–C or C–heteroatom bonds from a very broad selection of input starting materials.^2^ In recent years, attention has somewhat shifted to the implementation of more earth-abundant nickel catalyzed versions of these reactions.^3^ Furthermore, in 2010, Weix and his team pioneered and demonstrated the ability for nickel to catalyze cross-electrophile coupling (XEC), a complementary but distinctly different process from palladium-catalyzed cross-couplings.^4^ This process involves two electrophilic substrates such as a C(sp^2^) aryl halide and a C(sp^3^) alkyl halide, reacting to selectively form the cross-coupled product (Scheme 1A). A key attraction of this approach over traditional cross-coupling is the avoidance of a nucleophilic reaction component, such as boronic acids/esters, organozincs or Grignard reagents, which can cause selectivity issues, reduced functional group tolerance and often require point-of-use-preparation. However, these XEC processes are not without their own drawbacks, such as the need for activation of a reducing species, typically zinc or manganese, the use of inert glovebox set-ups, the need for long reaction times, and conditions for selectively forming the desired heterocoupled rather than homocoupled product.

Mechanochemistry features the use of grinding jars and balls to elicit chemical reactions and has seen accelerated interest in recent years in several areas of organic synthesis.^5^ With regards to cross-coupling, mechanochemistry has highlighted that reactions conducted by this method are characterized by reduced reaction times, reactivity of poorly soluble starting materials, and circumventing the necessity for inert reaction setups.^6^ Additionally, the simple and efficient mechanical activation of zerovalent metals such as zinc and manganese by ball-milling has also been demonstrated.^7^ Most recently, combining these observations, XEC using zinc or manganese metal as terminal reductant for a nickel-catalyzed process has been established employing grinding techniques (Scheme 1B).^8^ This includes previous work from our group, where XEC of aryl halides and alkyl halides was successfully carried out in a mixer ball-mill (Scheme 1B). This work demonstrated a dramatic reduction in reaction time (2 h vs >16 h in solution) and the avoidance of both inert reaction conditions and activation of the reductant (zinc). In addition, the process is solvent minimized where *N,N*-dimethylacetamide (DMA) is used in liquid-assisted grinding (LAG) quantities.^9^ Encouraged by this early promise of XEC by ball-milling, we sought to further elaborate the applicable substrate scope to twisted amides, leading to the corresponding acyl product series and heteroaryl halides leading to analogous heteroaryl products.

With regard to twisted amides, their nonplanar structure allows for exquisite reactivity of an otherwise comparatively inert carbonyl containing functional group. We note that use of the term “twisted amides” is somewhat inaccurate for many examples here which could be more accurately described as *N*-acyl imides. Pioneering work in 2015 by the groups of Szostak, Garg and Zou demonstrated the powerful electrophilic capability of a selection of twisted/activated amides for use with nucleophiles in organic synthesis.^10^

This has since been applied to several reactions, including transamidation,^11a^ transesterification,^11b^ and traditional cross-coupling.^11c−11k^ Application of twisted amides to reductive XEC using *N*-acyl glutarimide has emerged as a preferred twisted amide motif owing to a near full orthogonal twist (up to τ = 89.1°) and its facile synthesis.^12^ However, conventional solution methods are thus far limited to three studies, including a photoredox (Ir)/Ni dual catalysis approach.^13^ A more direct approach to this class of products could also arise from the cross-electrophile coupling of acid chlorides or anhydrides.^14^

Exploration of the use of twisted amides under ball-milling conditions was first reported this year by Zhang and Szostak, demonstrating their participation in palladium catalyzed Suzuki–Miyaura cross-coupling with boronic acids.^15^ Upon implementing our previously successful XEC ball-milling conditions for both of the substrate classes of interest, it was found that in the case of the twisted amides, the desired acyl product could be formed in 10% yield with the major product of this process returned as the self-coupled product derived from homocoupling of the alkyl-halide (Scheme 1C). Whereas for 2-bromopyridine, the desired cross-electrophile coupled product was delivered in 34% yield, with only a minor amount of the bipyridine dimer observed. The latter observations correspond well with the solution processes explored by Weix and co-workers, where heteroaromatic halides such as pyridines are not well tolerated under these reaction conditions and require subsequent and significant development of different optimal reaction conditions.^16^ In order to deliver these substrates as competent input starting materials for the ball milling XEC process, we set out to develop conditions for each substrate class, with a focus on the stoichiometry of the electrophile, the ligand, the reductant, and the metal salt (I, II, III, IV, Scheme 1C).

## Results and Discussion

**Scheme 2:**
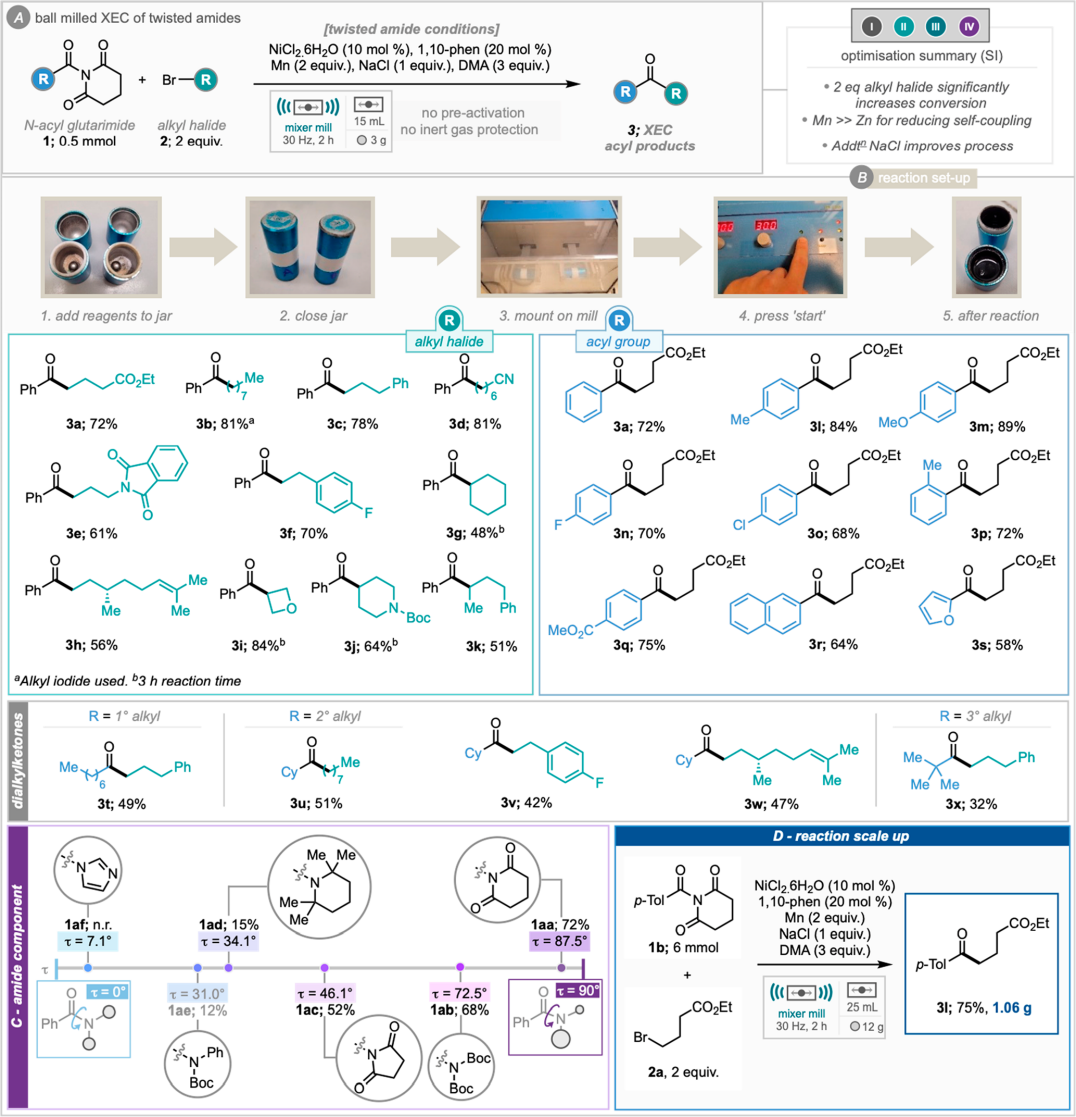
Mechanochemical XEC of Twisted Amides, Optimization, Substrate Scope, and Scale-up

Our studies into mechanochemical cross-electrophile coupling of activated amides began by assessing model reaction of *N*-benzoyl glutarimide and ethyl 4-bromobutyrate. Highlights of the optimization studies (see Table S1 in the Supporting Information for full details) revealed that (1) 2 equiv of alkyl halide were required for effective cross-coupling, (2) manganese rather than zinc was much more effective at delivering cross-coupled product over self-coupled product, and (3) inclusion of 1 equiv of NaCl was also a key difference to enable high yields (Scheme 2A). While NaCl has previously been demonstrated for use as a grinding auxiliary, in this case, NaCl may have a less innocent role in the reaction with a direct effect on the catalytic cycle.^17^ The use of 2 equiv of alkyl halide was found to be optimal (compared to 1.5 and 2.5 equiv early in the optimization, Table S1), affording a compromise between desired cross-coupled product versus homocoupled side product. The optimized conditions could be reliably applied to the mechanochemical XEC process leading selectively to the acylation product with little to no decarbonylated product seen in any case. A scope of this reaction process was then devised whereby the tolerance of each of the reaction components could be explored (Scheme 2A). First, a range of alkyl halides were subjected to the reaction conditions where simple chain alkyl halides (**3b**–**d**, **3f**) along with a range functionality was tolerated, including primary alkyl halides containing a carboxylic ester (**3a**), nitrile (**3d**), protected amine (**3e**), and distal alkene (**3h**). Secondary alkyl halides also proved to be successful under the reaction conditions, although they required a longer reaction time of 3 h to achieve comparable yields (**3i, 3j**). Pleasingly, saturated heterocyclic oxetane (**3i**, 84%) and piperidine (**3j**, 64%) fragments could be introduced using this methodology. Unfortunately, coupling of tertiary alkyl halides proved to be unsuccessful as discovered by the reaction of *N*-benzoyl glutarimide with *tert*-butyl iodide, resulting in no desired product. With regards to the backbone of the activated amides, the reductive mechanochemical methodology was shown to be effective across a range of electronics with electron-poor amides (**3q**), electron-rich amides (**3m**), and electron-neutral systems (**3l**, **3n**, **3p**, **3r)** all tolerated in good to excellent yield. Notably, the sterically hindered ortho-substituted acyl glutarimide was coupled successfully affording the product in good yield (**3p**, 72%). A small range of amides bearing alkyl chains at the carbonyl were shown to successfully furnish dialkyl ketones albeit in reduced yield (**3t**–**x**). A range of twisted amides exhibiting a variety of out-of-plane “twist” (τ) values were examined for application to this newly developed process (Scheme 2C). The results show a clear correlation between torsional twist and product yield. Amides bearing glutarimide functionality have a very high twist of 87.5° and this affords the highest product yield (**1aa**, 72%). From here, decreasing the N–C(O) bond twist gave decreasing yields through di-*tert*-butylcarbonate amide **1ab** (72.6°, 68%), *N*-acyl succinimide **1ac** (46.1°, 52%), TMP-amide **1ad** (34.1°, 15%), and *N*-Ph,Boc amide **1ae** (31°, 12%).

*N*-Acyl imidazole **1af** afforded no product upon attempted coupling, suggesting oxidative insertion did not occur. While the torsional twist for this amide is not exact, it is estimated to be around 7°.^18^ From the results, it is clear that with a decrease in torsional twist, a decrease in activation occurs, and subsequently, a reduction in yield is observed with a rotational limit for this mechanochemical protocol lying between 7° (**1af**, acyl imidazole) and 31° (**1ae**, *N*-Ph,Boc). An example of this reaction was also scaled up to 6 mmol (12-fold), delivering the cross coupled product with versatile carbonyl functionality on a gram scale (**3l**, 75%, Scheme 2D).

**Scheme 3:**
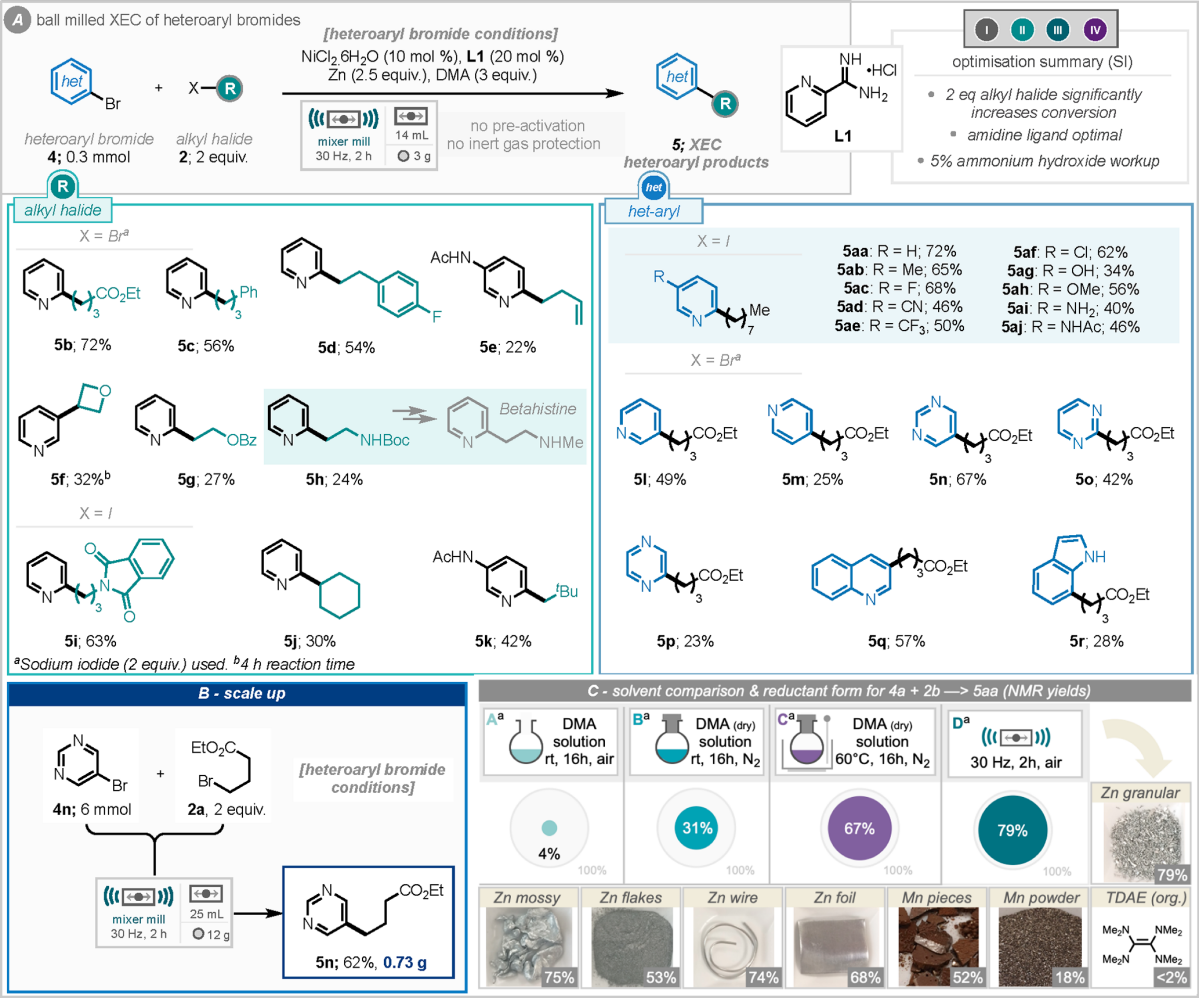
Mechanochemical XEC of Heteroaryl Bromides, Optimization, Substrate Scope, Scale-up, Solution Comparisons, and Reductants

Next, we turned attention to developing the avenue of ball-milling enabled cross-electrophile coupling of heteroaryl halides. Heteroaryl substrates present a particular problem when they contain coordinating nitrogen atoms which can tie-up/inhibit/slow-down catalysis. Our studies began by assessing model reaction of 2-bromopyridine and 1-iodooctane. Highlights of the optimization studies (see Table S3 in the Supporting Information for full details) revealed that (1) 2 equiv of alkyl halide were required for effective cross-coupling, (2) utility of amidine ligand (**L1**, Scheme 3A), led to significantly improved performance,^16,19^ (3) alteration of the workup process to incorporate 5% ammonium hydroxide was imperative for reproducibility in product isolation. The use of 2 equiv of alkyl halide in this instance (compared to 1.5 and 3.0 equiv early in the optimization, Table S2), affords the best conversion to cross-coupled product. Assessment of the metal to ligand ratio identified that 1:2 rather than 1:1 or 1:4, led to the greatest yields of the desired product (see Table S2). With optimal conditions in hand, the model target product (**5aa**) could be reliably prepared in 72% isolated yield, and so application of these conditions to a broader substrate scope was explored. Initially, a variety of pyridines, substituted at the 5-position were reacted with 1-iodooctane under the optimized conditions, providing a total of nine further analogues (**5ab**–**5aj**) to the model reaction product (**5aa**) in moderate to good yields.

This included tolerance for electron-withdrawing trifluoromethyl (**5ae**) and cyano (**5ad**) groups, along with electron-donating methoxy (**5ah**) and acetamido (**5aj**) groups. A chloride-substituted bromopyridine was also tolerated (**5af**), chemoselectively reacting at C–Br, opening the possibility for further subsequent functionalization. Gratifyingly, a free hydroxyl (**5ag**) and a free amine (**5ai**) were also tolerated, albeit in lower yield. In lower yielding instances, such as these, homocoupling and protodebromination of the heteroaromatic halide are observed as byproducts. Following this, a range of *N*-heteroaromatic bromides were tested under the optimized conditions; however, the alkyl halide coupling partner was altered to ethyl 4-bromobutyrate to provide more synthetically versatile products. In this case, it was found that 2 equiv of sodium iodide were required to improve product yields (see Supporting Information, Table S4 for details). To this end, products of 3-bromopyridine (**5l**), 4-bromopyridine (**5m**), 5- and 2-bromopyrimidine (**5n** and **5o**), 2-bromopyrazine (**5p**), 3-bromoquinoline (**5q**), and a free indole (**5r**) were successfully formed in moderate to good yields. The scope of alkyl halides was also assessed, revealing that a wide variety of functionalized alkyl bromides and iodides could be tolerated. These include a fluorinated homobenzyl substrate (**5d**), phthalimide (**5i**), cyclic alkyl halides including cyclohexyl (**5j**) and oxetane (**5f**), terminal alkene (**5e**), and neopentyl (**5k**), although in the latter two cases acetamido substituted pyridine was used to combat volatility issues encountered with 2-bromopyridine. Synthetically useful benzoate ester substituted pyridine (**5g**) and *N*-*tert*-butyloxycarbonyl- (Boc) protected amine (**5h**) were tolerated, albeit in reduced yields. The latter of which is closely related to Betahistine, used to treat Ménière’s disease. It was also demonstrated that the reaction could be scaled 20-fold (0.3 to 6 mmol), affording 730 mg of pyrimidine coupled product **5n** in 62% yield (Scheme 3B). 2-Iodopyridine and 2-chloropyridine were also suitable coupling partners, providing the cross-coupled product with iodooctane in 36% and 31% (NMR yield); a marked improvement on our previous attempts at aryl chloride coupling.^8a^ Additional studies included investigations into the influence of the reductant form and comparison to solution reaction conditions. Solution comparison studies included a room temperature reaction under an air atmosphere for 16 h (conditions A, Scheme 3C), room temperature reaction under a nitrogen atmosphere for 16 h (conditions B, Scheme 3C), and a 60 °C reaction under a nitrogen atmosphere for 16 h (conditions C, Scheme 3C). Conditions A, the most comparable to our ball-milled process, only led to 5% NMR yield of **5aa** product after 16 h. Conditions B led to 31% NMR yield of product and conditions C, the most comparable to Weix and co-workers’ conditions, led to 67% NMR yield of product. While not a 1:1 comparison with solution-phase reports, the comparable yield achieved with conditions C validates these conditions as competent also for a solution-phase approach.^16,20^ Notably, application of solvent conditions C to *N*-acyl imide substrates led to relatively poor reaction performance. These results highlight the potential operational/protocol improvements that the ball-milled process can achieve over solution-based processes. Applying the developed ball mill conditions to a variety of zinc forms (including moss, flakes, wire, and foil), identified that all forms could effectively participate in this reaction process.

Equally, manganese could be utilized as the reductant, with manganese pieces proving to be more successful than the powder form. The organic reductant tetrakis(dimethylamino)ethylene (TDAE) was unsuitable in this process.

**Scheme 4:**
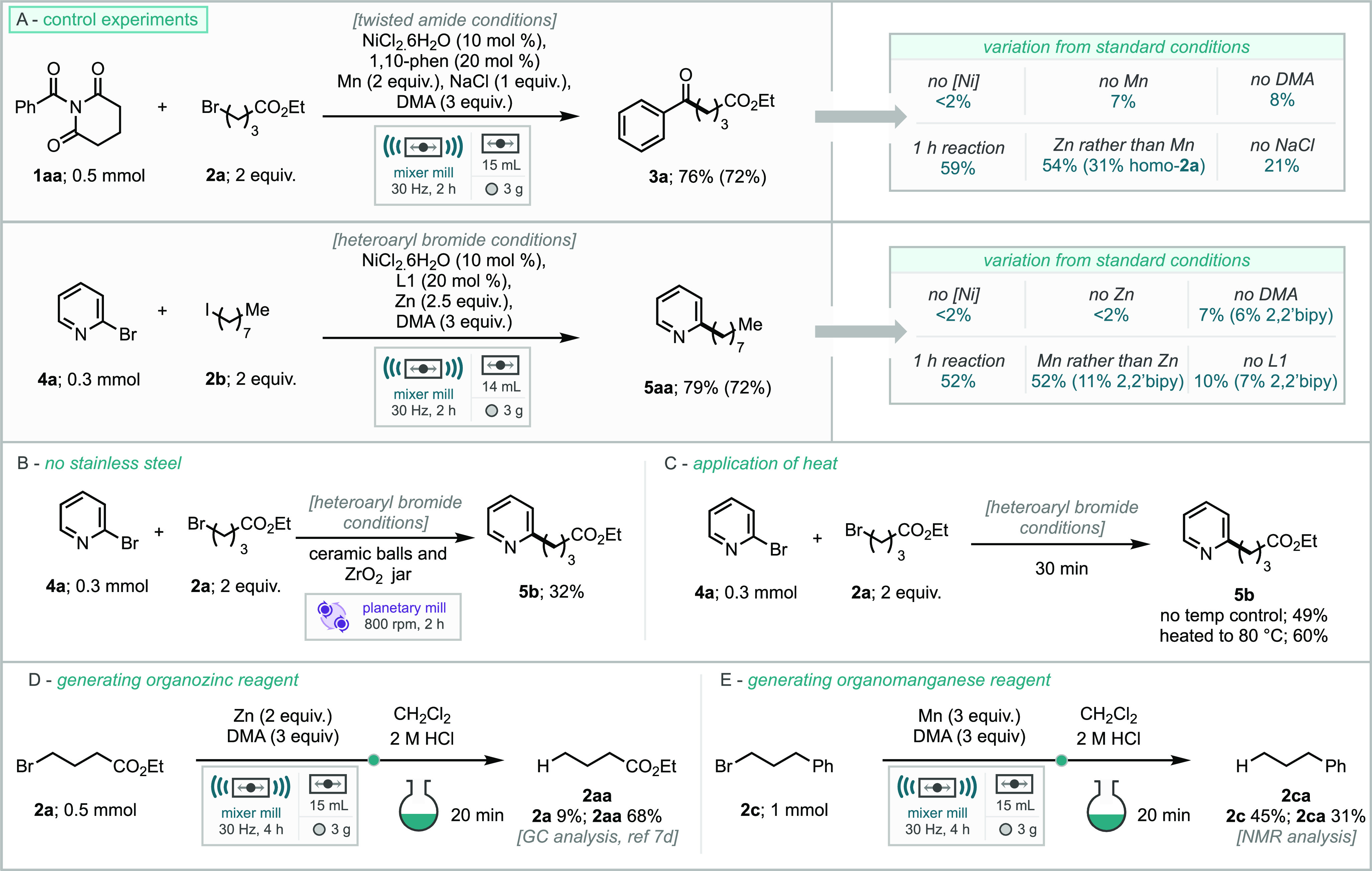
Control Experiments for the Mechanochemical XEC of Heteroaryl Bromides and *N*-Acyl Imides

A summary of key control experiments are shown in Scheme 4A. In the case of *N*-acyl imide (**1aa**), the experiments show that the nickel catalyst, manganese reductant, DMA LAG, and sodium chloride are all imperative for effective reaction, and in the absence of any of these, the reaction performance significantly drops. Furthermore, 1 h reaction time is insufficient for complete consumption of starting materials. Control experiments with 2-bromo pyridine (Scheme 4A), also demonstrate that the nickel catalyst, zinc reductant, and DMA LAG are essential for the reaction process. In the absence of both DMA LAG or the ligand, the reaction is significantly poorer for the desired product but also loses selectivity versus homocoupling of pyridine, leading to 2,2’bipyridine. As an additional control experiment, the reaction process was reproduced in a planetary mill with jars and balls fabricated from zirconia (ZrO_2_, Scheme 4B). In this experiment, the XEC product was formed in 32% yield, which is a significant drop from the stainless steel mixer mill protocol (72%); however, given that planetary milling imparts reduced impact forces to the reaction mixture, we interpret this as good evidence that stainless steel is not critical for an effective reaction. We have also explored the reaction under active heating (Scheme 4C). Milling pyridine **4a** and alkylhalide **2a** at room temperature (i.e., with no temperature control) for 30 min delivered the product (**5b**) in 49% NMR yield, whereas milling for 30 min with the heating device set to 80 °C returned a 60% NMR yield.

From a mechanistic perspective the understood solution based mechanism for both of the substrate classes describes a single electron transfer for the activation of the alkyl halide (as seen through much of Weix’s work).^4g^ However, given the change of reactor technology and absence of bulk solvent, *in situ* generation of organozinc or organomanganese intermediates and subsequent reaction with active Ni(II) species may be possible by the direct reaction of Zn(0) or Mn(0) with the alkyl bromide partner. Previous work has shown that the generation of organozinc reagents from alkyl halides and zinc metal, using ball-milling, is possible (Scheme 4D).^7d^ To explore this for the use of manganese, milling just the alkyl halide (**2c**) with Mn and DMA for 4 h followed by an acid quench led to a 31% NMR yield of the hydrolyzed compound (**2ca**, Scheme 4D).

**Scheme 5:**
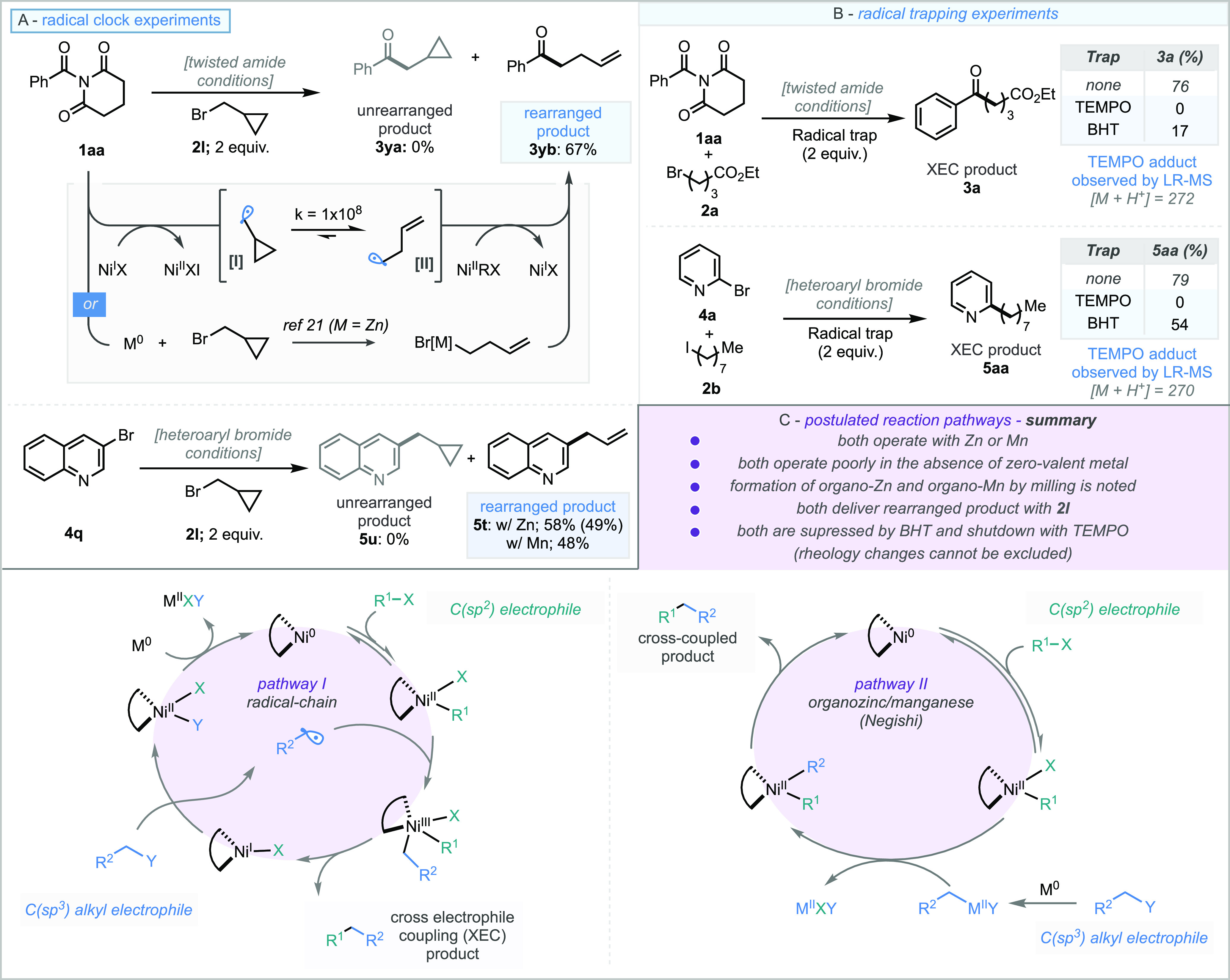
Control Experiments for the Mechanochemical XEC of Heteroaryl Bromides and *N-*Acyl Imides

This result demonstrates that the formation of an organomanganese intermediate is possible under these reaction conditions, also, which is in agreement with previous reports.^6c,7a^ To probe this mechanistic aspect further, cyclopropylmethyl bromide (**2l**) was used as the alkyl halide substrate, whereby formation of the rearranged product (i.e., ring-opened cyclopropyl) could suggest radical intermediates are present, due to the large rate constant associated with this process (Scheme 5A). In both systems presented here, the rearranged products **3yb** and **5t** were formed exclusively (67% for the *N*-acyl imide and 49% for the heteroaromatic substrates) and none of the unrearranged products were observed. However, it should be noted that the rearranged products could alternatively arise from ring opening of cyclopropylmethyl bromide (**2l**) by action of activated zinc metal.^21^ Such an intermediate could then participate in a traditional Negishi cross-coupling process. Radical trapping experiments using two equivalents of either 2,2,6,6-tetramethylpiperidine-1-oxyl (TEMPO) or butylated hydroxy-toluene (BHT) were carried out in both substrate sets (Scheme 5B). In the experiments using TEMPO, the adduct arising from the interception of the alkyl halide (from both **2a** and **2b**) could be observed by low resolution-mass spectrometry (LR-MS, included in the SI file). Perhaps even more significant was the absence of any XEC derived product from these reactions, suggesting that intercepting the alkyl radical shuts-down the reaction process leading to the XEC product. However, it is possible that these adducts could arise from single-electron reduction of the TEMPO radical to the *N*-oxide species, followed by an S_N_2 reaction onto the alkyl halides. The experiments involving BHT led to reduced yields in both systems (17% by NMR for twisted amides and 54% by NMR for heteroaryl), which could suggest suppression of the radical pathway of the reaction. It is noted that addition of either TEMPO or BHT alters both the filling degree and the rheology of the materials in the jar, and this could play a part in rendering the process ineffective (specifically transmission of mechanical energy). These combined observations suggest that the mechanochemical protocol operates either via a radical-chain mechanism analogous to that described for solution-based XEC reactions, or a Negishi-type mechanism via organo-zinc or manganese intermediates (Scheme 5C).

## Conclusion

In conclusion, nickel catalyzed cross-electrophile coupling can be readily achieved using ball-milling conditions, where the mechanical action of impact and grinding of the balls and jars against the sample, specifically the zero-valent metal reagent (manganese or zinc), is sufficient for an operationally more simplified process. In the case of twisted amides, optimal conditions (those with minimized homocoupling) require manganese, NaCl as a solid additive and are applicable to a range of out of plane twists (τ > 31°). Whereas for the heteroaryl halide coupling, an amidine ligand and zinc are imperative for an effective protocol. Both sets of conditions can be scaled to yield ∼1 g of product, and these reaction processes appear to proceed either in a manner similar to that rationalized in solution (i.e., via a single electron radical pathway) or via a Negishi-type pathway.

## Notes

Information about the data that underpins the results presented in this article, including how to access them, can be found in the University College London Research Data Repository 10.5522/04/19733707.v1.

## Supporting Information Available

Experimental procedures, characterization, and further details (PDF)
