# Supplementary material for: Mechanical Activation of Zero-Valent Metal Reductants for Nickel-Catalyzed Cross-Electrophile Coupling
Source: ACS Catal. 2022 Oct 25;12(21):13681–9. doi: 10.1021/acscatal.2c03117 (PMC9638985; doi:10.1021/acscatal.2c03117)
Supplement: Supplementary file 1 — cs2c03117_si_001.pdf [file cs2c03117_si_001.pdf]

SUPPORTING INFORMATION

Mechanical Activation of Zero Valent Metal Reductants for Nickel Catalyzed Cross-Electrophile Coupling

Andrew C. Jones,[a,†] Matthew T. J. Williams,[a,†] Louis C. Morrill,*[a] and Duncan L. Browne*[b]

*E-mail: Duncan.Browne@ucl.ac.uk

*E-mail: MorrillLC@cardiff.ac.uk

[†] *These authors contributed equally*

[a]*Cardiff Catalysis Institute, School of Chemistry, Cardiff University, Main Building, Park Place, Cardiff, CF10 3AT, UK*

[b]*School of Pharmacy, University College London, 29-39 Brunswick Square, Bloomsbury, London, WC1N 1AX, UK*

Table of Contents

1. General Information S3

1.1. Equipment used in this Study S4

1.2. A Practical Guide to Setting up a Ball-Milling Reaction S5

2. Experimental and Characterisation Data S6

2.1. Mechanochemical Cross-Electrophile Coupling (XEC) of Twisted Amides: Synthesis of Starting Materials S6

2.1.1. General Procedure A – Synthesis of *N*-Acyl Glutarimides from Acyl Chlorides S6

2.1.2. General procedure B – Synthesis of *N*-Acyl Glutarimides from Carboxylic Acids S6

2.2. Optimisation of Model Reaction S13

2.3. General Procedure C – Mechanochemical XEC of *N*-Acyl Glutarimides with Alkyl Halides S15

2.4. Scale-Up Experiment S23

2.5. Mechanistic Studies S24

2.5.1. Radical Clock Experiment S24

2.5.2. Radical Trapping Experiments S25

3.1. Mechanochemical XEC of Heteroaryl Halides S26

3.1.1. General Procedure D – Synthesis of Amidine Ligands S26

3.1.2. Optimisation of Model Reaction S27

3.1.3. Determination of Optimal Sodium Iodide Equivalents for Use of Alkyl Bromide Coupling Partners S29

3.1.4. General Procedure E – Mechanochemical XEC between Heteroaryl Bromides and Alkyl Halides S29

3.2. Scale-Up Experiment S39

3.3. Mechanistic Studies S39

3.3.1. Radical Clock Experiment S39

3.3.2. Radical Trapping Experiments S40

3.4. Solution-Phase Comparisons S41

3.5. Organomanganese Formation Studies S41

3.6. Heated Ball-Mill Experiments S42

3.7. Stainless Steel-Free Reactions in Planetary Ball-Mill S42

4. NMR Spectra S43

5. References S120

1. General Information

Unless otherwise stated, all reagents were purchased from commercial sources and used without further purification.

Room temperature (rt) refers to 20 – 25 °C. Ice/water baths were used to obtain temperatures of 0 °C. All reactions involving heating were carried out using DrySyn blocks and a contact thermometer.

Analytical thin layer chromatography was carried out using aluminium plates coated with silica (Kieselgel 60 F_{254} silica) and visualization was achieved using ultraviolet light (254 nm), followed by staining with a 1% aqueous $KMnO_4$ solution, unless otherwise stated. Flash column chromatography (FCC) used Kieselgel 60 silica in the solvent system stated. The petroleum ether (PE) utilised was in the 40 – 60 °C boiling range and the hexane used was HPLC grade (>95%).

Melting points (m.p.) were recorded on a Gallenkamp melting point apparatus and are reported corrected by linear calibration to benzophenone (47 – 49 °C) and benzoic acid (121 – 123 °C).

Infrared spectra were recorded on a Shimadzu IRAffinity-1 Fourier Transform ATIR spectrometer as thin films using a Pike MIRacle ATR accessory, with absorbance peaks quoted (v_{max}/cm^{-1}).

^{1}H, ^{13}C, ^{19}F NMR spectra were obtained on a Bruker Avance 300 (300 MHz ^{1}H, 75 MHz ^{13}C, 282 MHz ^{19}F), a Bruker Avance 400 (400 MHz ^{1}H, 101 MHz ^{13}C, 376 MHz ^{19}F) or a Bruker Avance 500 (500 MHz ^{1}H, 126 MHz ^{13}C, 471 MHz ^{19}F) spectrometer at rt in the solvent stated. ^{13}C experiments run as attached proton tests (APT) are specified in the tabulated data, where CH's and CH_3's are in positive mode and C's and CH_2's are in negative mode. Chemical shifts are reported in parts per million (ppm) relative to the residual solvent signal. All coupling constants, *J*, are quoted in Hertz (Hz). Multiplicities are reported based on their apparent appearance, with the following symbols: s = singlet, d = doublet, t = triplet, q = quartet, m = multiplet and multiples thereof.

High resolution mass spectral (HRMS) data were obtained on a Waters MALDI-TOF mx in Cardiff University.

The mixer ball-mill used was either an In Solido Technologies (IST) 500 Mixer Mill or a Retsch Mixer Mill 400. Unless otherwise stated, mechanochemical reactions were carried out in 14 mL IST or 15 mL FormTech Scientific (FTS) stainless steel jars, with a 3 g, 9 mm stainless steel ball.

The planetary ball-mill used was a Fritsch Planetary Micro Mill model "Pulverisette 7" using 12 mL zirconium oxide grinding bowls containing 3 g, 10 mm ceramic grinding balls. The grinding cycle was set to 15 minutes and to alternate direction between cycles after a 1 minute pause, unless stated otherwise. The mill was set to repeat these cycles until the reaction time had been reached.

1.1. Equipment used in this Study

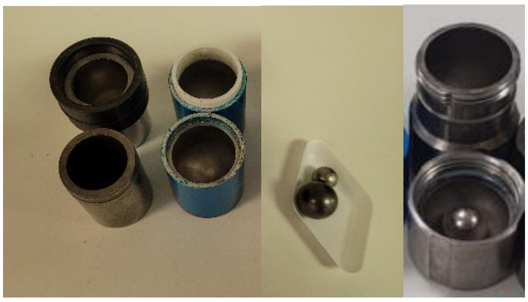

Figure S1 (from left to right):

14 mL IST stainless steel jar with retaining collar (http://www.insolidotech.org/accessories.html)

15 mL FTS Smartsnap™ stainless steel jar with PTFE retaining washer (https://formtechscientific.com/fts-1000-shakermill/products.html?section=accessories&accessory=smartsnap-grinding-jars)

25 mL Retsch stainless steel jar

(https://www.retsch.com/products/milling/ball-mills/mixer-mill-mm-400/order-data-quote-request/)

3 g, 9 mm and 12 g, 14 mm stainless steel milling balls from Retsch (https://www.retsch.com/products/milling/ballmills/mixer-mill-mm-400/order-data-quote-request/)

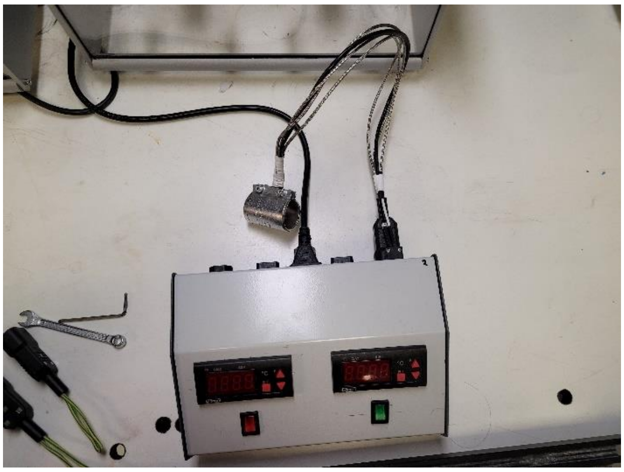

Figure S2. PID-controlled heating device used for heated experiments. For full description, see https://onlinelibrary.wiley.com/doi/10.1002/anie.202210508

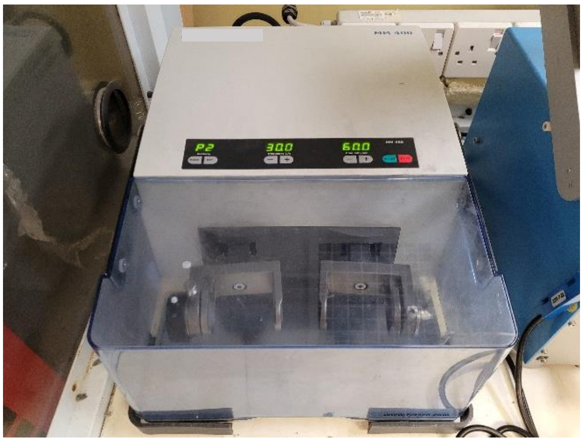

Figure S3. Retsch mixer mill 400 (MM400).

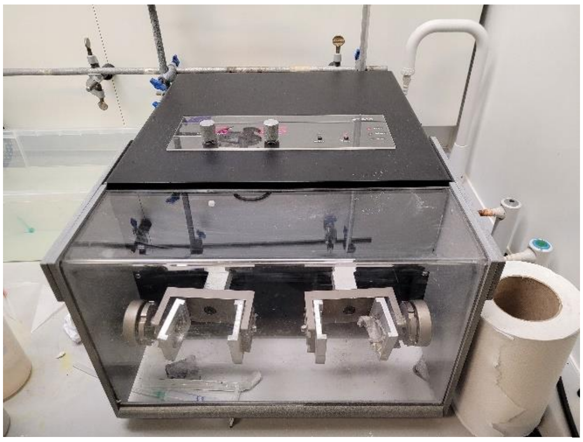

Figure S4. IST mixer mill 500 (IST 500).

Note: Retsch milling jars are only compatible with Retsch devices, whereas IST and FTS jars are compatible with both IST and Retsch devices.

1.2. A Practical Guide to Setting up a Ball-Milling Reaction

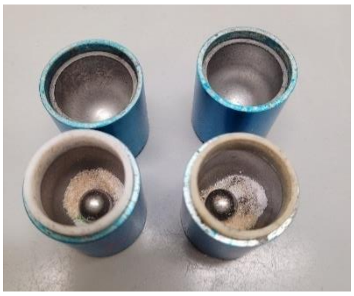

Step 1: Add solid reagents and grinding ball/balls to jar.

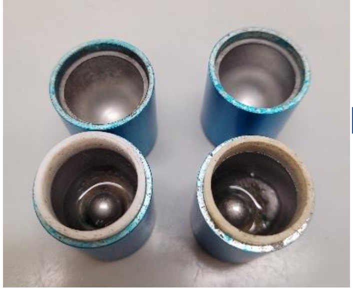

Step 2: Add liquid reagents to jar.

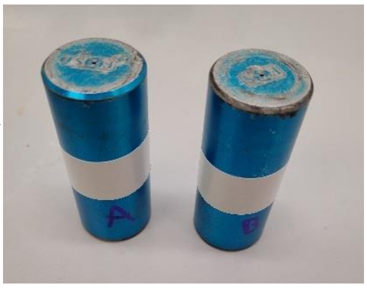

Step 3: Close jar (electrical tape can be used to prevent leaks).

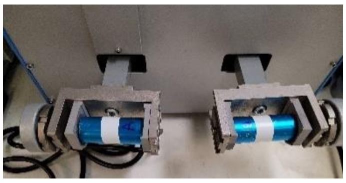

Step 4: Mount jars on mixer ball-mill.

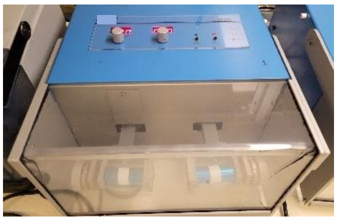

Step 5: Close lid of mixer ball-mill.

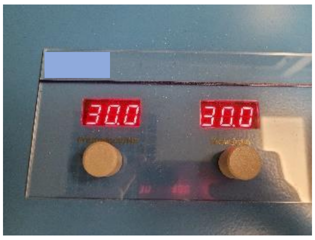

Step 6: Set desired milling frequency and reaction time.

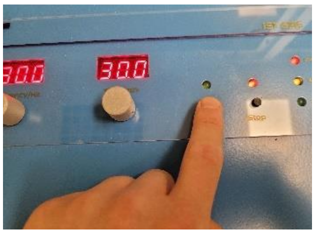

Step 7: Press 'start'.

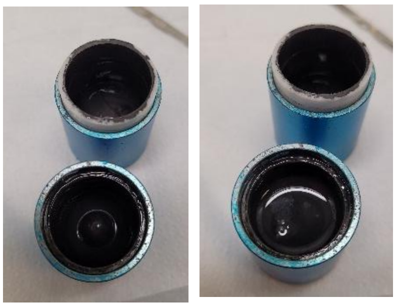

Step 8: Once complete, remove jar from mixer mill, open jar, then add small amount of organic solvent + water to help mobilize residue.

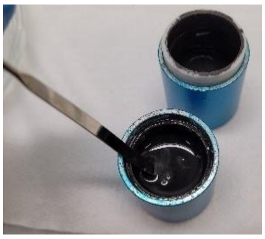

Step 9: Use a spatula to mobilize residue into the liquid.

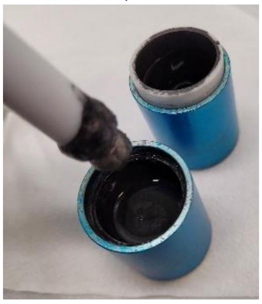

Step 10: Remove grinding ball e.g., using magnetic rod.

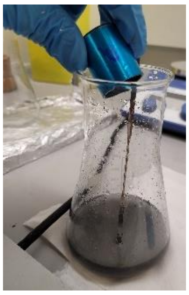

Step 11: Transfer mixture to a conical flask containing the appropriate aqueous quench (e.g., 1 M HCl), then repeat steps 8 and 9 until all residue has been transferred from the jar.

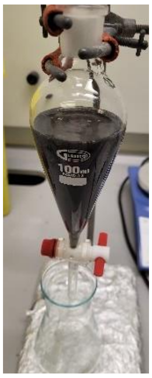

Step 12: Transfer to separating funnel and complete workup.

2. Experimental and Characterisation Data

2.1. Mechanochemical Cross-Electrophile Coupling (XEC) of Twisted Amides: Synthesis of Starting Materials

2.1.1. General Procedure A – Synthesis of *N*-Acyl Glutarimides from Acyl Chlorides

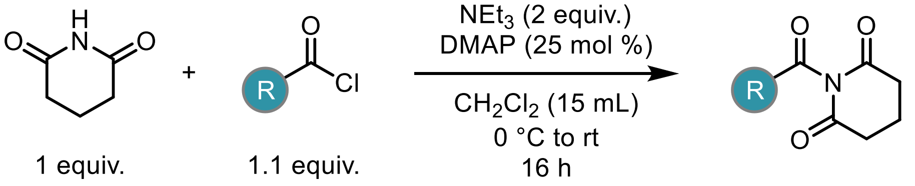

To a round-bottomed flask, equipped with a magnetic stirrer, was added glutarimide (1.13 g, 10 mmol, 1 equiv.), triethylamine (2.79 mL, 20 mmol, 2 equiv.), 4-dimethylaminopyridine (0.31 g, 2.5 mmol, 25 mol %) and CH_2Cl_2 (15 mL). Acyl chloride (11 mmol, 1.1 equiv.) was added dropwise over a period of 5 minutes at 0 °C with vigorous stirring. The reaction mixture was warmed to room temperature and stirred at this temperature for 16 hours. After this period, the mixture was diluted with CH_2Cl_2 (20 mL). The organic layer was then washed with 1 M HCl (25 mL), water (25 mL) and then brine (5 mL). The organic layer was then dried over magnesium sulfate, filtered and concentrated *in vacuo* to afford the crude product. Unless stated otherwise, the crude product was purified by recrystallisation, from toluene, to afford the pure *N*-Acyl glutarimide.

2.1.2. General procedure B – Synthesis of *N*-Acyl Glutarimides from Carboxylic Acids

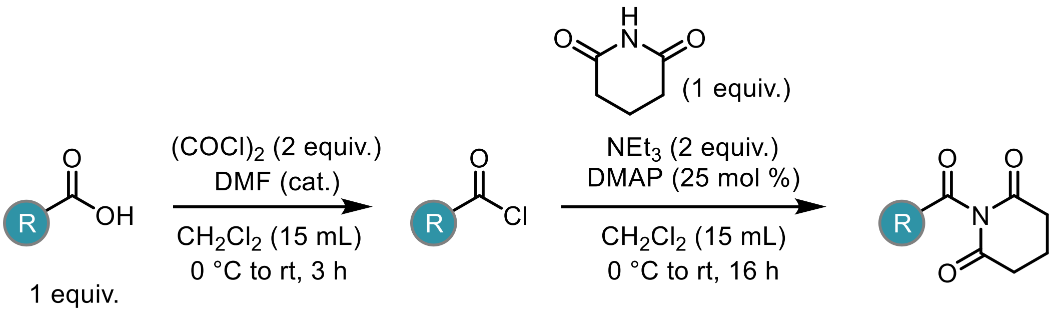

To a round-bottomed flask, equipped with a magnetic stirrer, was added carboxylic acid (10 mmol, 1 equiv.) and CH_2Cl_2 (15 mL). Oxalyl chloride (1.72 mL, 20 mmol, 2 equiv.) was added dropwise at 0 °C. A coupled of drops of *N,N*-dimethylformamide were added and the reaction warmed to room temperature. The reaction was stirred until gas evolution ceased (typically 3 hours). After this period, the mixture was concentrated under reduced pressure to remove HCl and excess oxalyl chloride to give the corresponding acid chloride, which was used without further purification. In a separate round-bottomed flask, glutarimide (0.79 g, 7 mmol, 1 equiv.), triethylamine (1.95 mL, 14 mmol, 2 equiv.), 4-(dimethylamino)pyridine (0.21 g, 1.75 mmol, 25 mol % with respect to glutarimide) and CH_2Cl_2 (15 mL) were added. The mixture was cooled to 0 °C and the acid chloride was added dropwise. The reaction mixture was warmed to room temperature and stirred for 16 hours. After this period, the mixture was diluted with CH_2Cl_2 (20 mL). The organic layer was then washed with 1 M HCl (25 mL), water (25 mL) and then brine (25 mL). The organic layer was then dried with magnesium sulfate, filtered and concentrated *in vacuo* to afford the crude product. Unless stated otherwise, the crude product was purified by recrystallisation, from toluene, to afford the pure *N*-Acyl glutarimide.

1-benzoylpiperidine-2,6-dione (1aa)

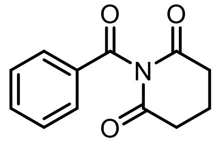

Prepared according to general procedure A to give the title compound (1.82 g, 84%) as a beige solid; m.p.111 – 113 °C.

^{1}**H NMR** (500 MHz, $CDCl_3$) δ 7.88 – 7.84 (m, 2H), 7.66 – 7.62 (m, 1H), 7.52 – 7.46 (m, 2H), 2.78 (t, *J* = 6.5 Hz, 4H), 2.15 (p, *J* = 6.5 Hz, 2H).

^{13}**C {^{1}H} NMR** (126 MHz, $CDCl_3$) δ 172.0, 170.9, 135.1, 131.9, 130.3, 129.3, 32.5, 17.6.

These data are consistent with the literature.[1]

1-(4-methylbenzoyl)piperidine-2,6-dione (1b)

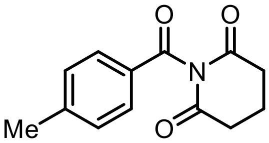

Prepared according to general procedure A to give the title compound (1.66 g, 72%) as an off-white solid; m.p. 168 – 170 °C.

^{1}**H NMR** (500 MHz, $CDCl_3$) δ 7.75 (d, *J* = 8.2 Hz, 2H), 7.28 (d, *J* = 8.1 Hz, 2H), 2.77 (t, *J* = 6.5 Hz, 4H), 2.42 (s, 3H), 2.14 (p, *J* = 6.5 Hz, 2H).

^{13}**C {^{1}H} NMR** (126 MHz, $CDCl_3$) δ 172.0, 170.5, 146.5, 130.5, 130.0, 129.3, 32.5, 22.0, 17.6.

These data are consistent with the literature.[2]

1-(4-fluorobenzoyl)piperidine-2,6-dione (1c)

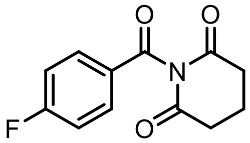

Prepared according to general procedure A to give the title compound (1.76 g, 75%) as a beige solid; m.p.169 – 172 °C.

^{1}**H NMR** (500 MHz, $CDCl_3$) δ 7.89 (dd, *J* = 8.7, 5.3 Hz, 2H), 7.16 (app t, *J* = 8.5 Hz, 2H), 2.78 (t, *J* = 6.5 Hz, 4H), 2.15 (p, *J* = 6.5 Hz, 2H).

^{13}**C {^{1}H} NMR** (126 MHz, $CDCl_3$) δ 171.9, 169.5, 166.7 (d, *J* = 258.4 Hz), 133.0 (d, *J* = 9.9 Hz), 128.3 (d, *J* = 2.8 Hz), 116.5 (d, *J* = 22.4 Hz), 32.4, 17.5.

^{19}**F NMR** (471 MHz, $CDCl_3$) δ -101.32 – -101.24 (m).

These data are consistent with the literature.[2]

1-(4-methoxybenzoyl)piperidine-2,6-dione (1d)

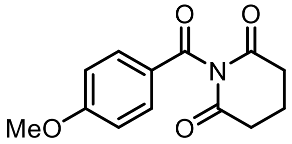

Synthesised according to the general procedure A on a 5 mmol scale to give the title compound (1.00 g, 81%) as a beige solid; m.p. 150 – 152 °C.

^{1}H NMR (500 MHz, $CDCl_3$) δ 7.84 – 7.80 (m, 2H), 6.97 – 6.91 (m, 2H), 3.87 (s, 3H), 2.76 (t, *J* = 6.6 Hz, 4H), 2.13 (p, *J* = 6.6 Hz, 2H).

^{13}C {^{1}H} NMR (126 MHz, $CDCl_3$) δ 172.0, 169.6, 165.2, 132.9, 124.6, 114.6, 55.8, 32.6, 17.7.

These data are consistent with the literature.[2]

1-(4-chlorobenzoyl)piperidine-2,6-dione (1e)

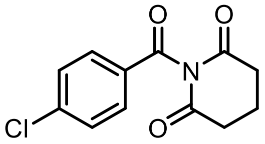

Prepared according to the general procedure A to the title compound (2.05 g, 82%) as a white solid; m.p. 167 – 169 °C.

^{1}H NMR (500 MHz, $CDCl_3$) δ 7.81 – 7.77 (m, 2H), 7.48 – 7.45 (m, 2H), 2.80 – 2.76 (m, 4H), 2.19 – 2.11 (m, 2H).

^{13}C {^{1}H} NMR (126 MHz, $CDCl_3$) δ 172.0, 170.0, 141.8, 131.6, 130.4, 129.7, 32.5, 17.6.

These data are consistent with the literature.[2]

Methyl 4-(2,6-dioxopiperidine-1-carbonyl)benzoate (1f)

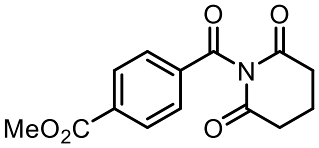

Prepared according to general procedure B to give the title compound (1.65 g, 85%) as a beige solid; m.p. 93 – 94 °C.

^{1}H NMR (500 MHz, $CDCl_3$) δ 8.16 – 8.13 (m, 2H), 7.93 – 7.89 (m, 2H), 3.96 (s, 3H), 2.82 – 2.77 (m, 4H), 2.20 – 2.14 (m, 2H).

^{13}C {^{1}H} NMR (126 MHz, $CDCl_3$) δ 172.0, 170.5, 165.9, 135.6, 135.3, 130.4, 130.1, 52.8, 32.6, 17.6.

These data are consistent with the literature.[2]

1-(cyclohexanecarbonyl)piperidine-2,6-dione (1g)

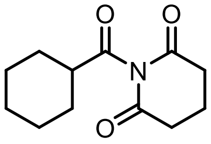

Prepared according to the general procedure A to give the title compound (1.92 g, 86%) as a white solid; m.p. 69 – 72 °C.

^{1}H NMR (500 MHz, $CDCl_3$) δ 2.68 – 2.58 (m, 5H), 2.05 – 1.95 (m, 1H), 1.84 – 1.77 (m, 4H), 1.68 – 1.62 (m, 2H), 1.52 – 1.41 (m, 1H), 1.31 – 1.17 (m, 3H).

^{13}C {^{1}H} NMR (126 MHz, $CDCl_3$) δ 181.0, 172.0, 48.8, 32.5, 28.1, 25.7, 25.5, 17.5.

These data are consistent with the literature.[3]

1-(2-methylbenzoyl)piperidine-2,6-dione (1h)

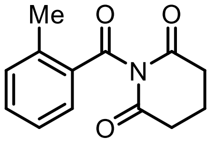

Prepared according to general procedure A. Purified by FCC (50% EtOAc/PE) to give the title compound (1.70 g, 73%) as a pale yellow solid; m.p. 91 – 94°C.

^{1}H NMR (500 MHz, $CDCl_3$) δ 7.49 (dd, *J* = 7.8, 1.3 Hz, 1H), 7.46 (td, *J* = 7.5, 1.4 Hz, 1H), 7.33 – 7.31 (m, 1H), 7.27 – 7.23 (m, 1H), 2.75 (t, *J* = 6.6 Hz, 4H), 2.12 (p, *J* = 6.6 Hz, 2H).

^{13}C {^{1}H} NMR (126 MHz, $CDCl_3$) δ 172.1, 170.8, 142.7, 133.9, 132.6, 131.3, 130.8, 126.3, 32.6, 22.0, 17.6.

These data are consistent with the literature.[1]

1-(2-naphthoyl)piperidine-2,6-dione (1i)

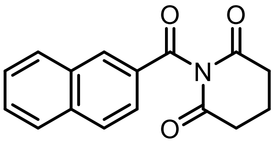

Prepared according to procedure A to give the title compound as a beige solid (2.10 g, 79%); m.p. 150 – 152 °C.

^{1}H NMR (500 MHz, $CDCl_3$) δ 8.34 (d, *J* = 0.7 Hz, 1H), 7.96 – 7.92 (m, 3H), 7.89 (dd, *J* = 8.2, 0.6 Hz, 1H), 7.64 (ddd, *J* = 8.2, 6.9, 1.2 Hz, 1H), 7.57 (ddd, *J* = 8.1, 6.9, 1.2 Hz, 1H), 2.83 (t, *J* = 6.6 Hz, 4H), 2.21 (p, *J* = 6.5 Hz, 2H).

^{13}C {^{1}H} NMR (126 MHz, $CDCl_3$) δ 172.1, 171.0, 136.6, 132.8, 132.6, 130.0, 129.7, 129.4, 129.3, 128.1, 127.3, 124.9, 32.6, 17.7.

These data are consistent with the literature.[2]

1-(furan-2-carbonyl)piperidine-2,6-dione (1j)

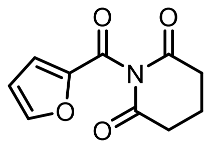

Prepared according to procedure A to give the title compound (1.74 g, 84%) as a white solid; m.p. 104 – 105 °C.

^{1}H NMR (500 MHz, $CDCl_3$) δ 7.60 (s, 1H), 7.40 (d, *J* = 2.8 Hz, 1H), 6.61 (s, 1H), 2.76 (t, *J* = 6.3 Hz, 4H), 2.15 – 2.05 (m, 2H).

^{13}C {^{1}H} NMR (126 MHz, $CDCl_3$) δ 171.8, 159.5, 148.4, 147.6, 122.3, 113.8, 32.5, 17.5.

These data are consistent with the literature.[4]

1-octanoylpiperidine-2,6-dione (1k)

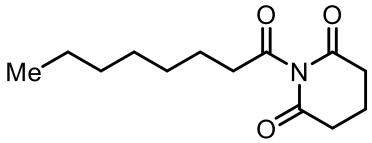

Prepared according to procedure A. Purified by FCC (50% EtOAc/PE) to give the title compound (1.58 g, 66%) as a white solid; m.p. 34 – 36 °C.

^{1}H NMR (500 MHz, $CDCl_3$) δ 2.66 – 2.56 (m, 6H), 1.99 – 1.93 (m, 2H), 1.67 – 1.60 (m, 2H), 1.34 – 1.18 (m, 8H), 0.83 (t, *J* = 7.0 Hz, 3H).

^{13}C {^{1}H} NMR (126 MHz, $CDCl_3$) δ 178.4, 171.7, 40.9, 32.2, 31.6, 28.89, 28.5, 23.4, 22.6, 17.3, 14.0.

These NMR data are consistent with the literature.[5]

1-pivaloylpiperidine-2,6-dione (1l)

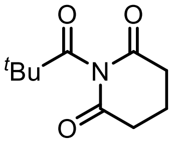

Prepared according to procedure A. The crude mixture was dissolved in the minimum amount of CH_2Cl_2 and added dropwise to petroleum ether. The white precipitate was then filtered under vacuum to give the title compound (1.47 g, 75%) as a white solid; m.p. 49 – 51 °C.

^{1}H NMR (500 MHz, $CDCl_3$) δ 2.65 (t, *J* = 6.5 Hz, 4H), 2.02 (p, *J* = 6.6 Hz, 2H), 1.26 (s, 9H).

^{13}C {^{1}H} NMR (126 MHz, $CDCl_3$) δ 185.7, 172.0, 43.9, 32.3, 27.3, 17.6.

These data are consistent with the literature.[3]

1-benzoylpyrrolidine-2,5-dione (1ac)

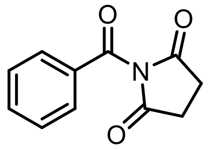

Prepared according to procedure A to give the title compound (1.81 g, 89%) as a white solid; m.p. 118 – 120 °C.

^{1}H NMR (500 MHz, $CDCl_3$) δ 7.87 – 7.83 (m, 2H), 7.67 (tt, *J* = 7.2, 1.2 Hz, 1H), 7.52 – 7.48 (m, 2H), 2.94 (s, 4H).

^{13}C {^{1}H} NMR (126 MHz, $CDCl_3$) δ 174.8, 167.8, 135.3, 131.4, 130.7, 129.1, 29.2.

These data are consistent with literature.[2]

***N,N*-Bis(*tert*-butoxycarbonyl)benzamide (1ab)**

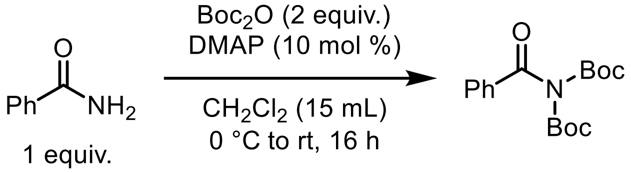

To a flame dried round-bottomed flask, equipped with a magnetic stirrer, was added benzamide (1.21 g, 10 mmol, 1 equiv.), 4-(dimethylamino)pyridine (0.12 g, 1 mmol, 10 mol %), and CH_2Cl_2 (15 mL). Di-tert-butyl dicarbonate (4.37 g, 20 mmol, 2 equiv.) was added in portions at 0 °C with vigorous stirring. The reaction was warmed to room temperature and stirred for 16 hours. After this time, the reaction mixture was diluted with CH_2Cl_2. The organic mixture was then washed with 1 M HCl (20 mL), water (20 mL), and then brine (20 mL). The organic layer was then dried over magnesium sulfate, filtered and concentrated *in vacuo* to afford the crude product. The crude product was purified by FCC (10% EtOAc/hexane) to give the title compound (2.66 g, 83%) as a colourless oil.

^{1}H NMR (500 MHz, $CDCl_3$) δ 7.83 (dd, *J* = 8.4, 1.3 Hz, 2H), 7.62 – 7.58 (m, 1H), 7.50 – 7.45 (m, 2H), 1.37 (s, 18H).

^{13}C {^{1}H} NMR (126 MHz, $CDCl_3$) δ 169.5, 149.9, 134.3, 133.6, 129.2, 128.8, 84.4, 27.7.

These data are consistent with the literature.[2]

Phenyl(2,2,6,6-tetramethylpiperidin-1-yl)methanone (1ad)

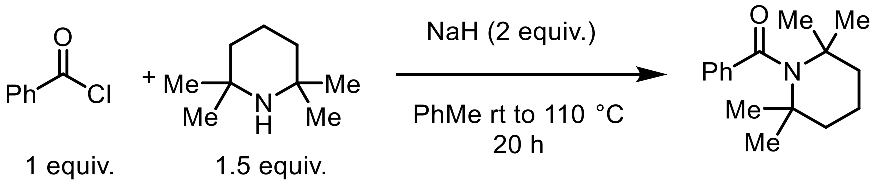

In a flame dried round-bottomed flask, equipped with a magnetic stirrer, 2,2,6,6-tetramethylpiperidine (2.53 mL, 15 mmol, 1.5 equiv.) was added to a suspension of sodium hydride (60% dispersion in paraffin oil, 0.89 g, 20 mmol, 2 equiv.) in toluene (20 mL). The mixture was stirred at room temperature for 10 minutes. Benzoyl chloride (1.16 mL, 10 mmol, 1 equiv.) was added dropwise, and the reaction was refluxed for 20 hours. The reaction was cooled to room temperature and EtOH (5 mL) was added to quench excess sodium hydride, followed by slow addition of water (10 mL). CH_2Cl_2 (25 mL) was added, and the layers were separated. The organic layer was washed with $NaHCO_3$ (2 x 20 mL) followed by brine (20 mL) and concentrated *in vacuo* to give the crude product. The crude product was purified by

FCC (10% EtOAc/hexane) to give the title compound (0.88 g, 38%) as a white crystalline solid; m.p. 79 – 82 °C.

^{1}H NMR (500 MHz, $CDCl_3$) δ 7.45 – 7.41 (m, 2H), 7.37 – 7.32 (m, 3H), 1.79 (s, 6H), 1.37 (s, 12H).

^{13}C {^{1}H} NMR (126 MHz, $CDCl_3$) δ 176.9, 143.4, 129.5, 128.0, 56.7, 37.1, 30.6, 15.1.

These data are consistent with literature.[1]

(1H-imidazol-1-yl)(phenyl)methanone (1af)

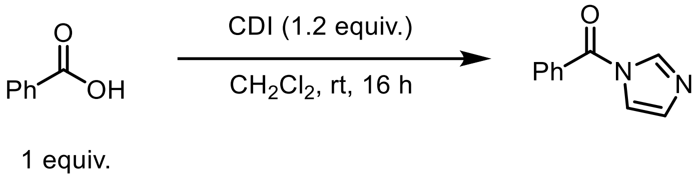

To a round-bottomed flask, equipped with a magnetic stirrer, containing a solution of benzoic acid (0.61 g, 5 mmol, 1 equiv.) in CH_2Cl_2 (20 mL) was added *N,N'*-carbonyldiimidazole (0.97 g, 6 mmol, 1.2 equiv.). The mixture was stirred at room temperature for 16 hours. Distilled water (25 mL) was added, and the layers were separated. The organic layer was washed with water (2 x 25 mL) and then brine (30 mL). The organic phase was then dried over magnesium sulfate, filtered, and concentrated *in vacuo* to afford the title compound (0.77 g, 90%) as a colourless oil.

^{1}H NMR (500 MHz, $CDCl_3$) δ 8.09 (s, 1H), 7.81 – 7.78 (m, 2H), 7.71 – 7.67 (m, 1H), 7.59 – 7.54 (m, 3H), 7.18 (s, 1H).

^{13}C {^{1}H} NMR (126 MHz, $CDCl_3$) δ 166.3, 138.4, 133.8, 132.0, 131.6, 129.9, 129.1, 118.2.

These data are consistent with literature.[6]

***N*-methoxy-*N*-methylbenzamide (1m)**

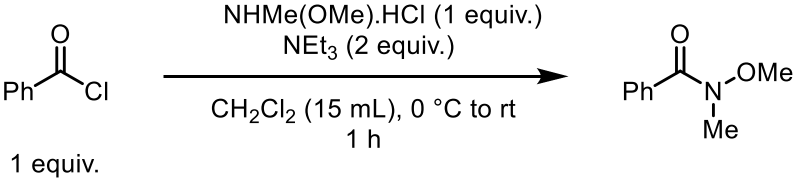

To a flame dried round-bottomed flask, equipped with a magnetic stirrer, was added *N*-methoxymethylamine hydrochloride (0.39 g, 5 mmol, 1 equiv.) and CH_2Cl_2 (15 mL). The mixture was cooled to 0 °C and triethylamine (1.4 mL, 10 mmol, 2 equiv.) was added slowly. Benzoyl chloride (0.58 mL, 5 mmol, 1 equiv.) was added dropwise. After 10 minutes of stirring at 0 °C, the mixture was warmed to room temperature and stirred for 1 hour. The reaction mixture was quenched with saturated aqueous $NaHCO_3$ (10 mL) and the layers separated. The organic phase was washed with 1M HCl (2 x 10 mL) and brine (10 mL). The organic phase was then dried over magnesium sulfate, filtered, and concentrated *in vacuo* to give the title compound (0.81 g, 98%) as a colourless oil.

^{1}H NMR (500 MHz, $CDCl_3$) δ 7.68 – 7.64 (m, 2H), 7.47 – 7.42 (m, 1H), 7.41 – 7.37 (m, 2H), 3.55 (s, 3H), 3.36 (s, 3H).

^{13}C {^{1}H} NMR (126 MHz, $CDCl_3$) δ 170.1, 134.2, 130.7, 128.3, 128.1, 61.2, 33.9.

These data are consistent with the literature.[7]

(3-bromobutyl)benzene (1o)

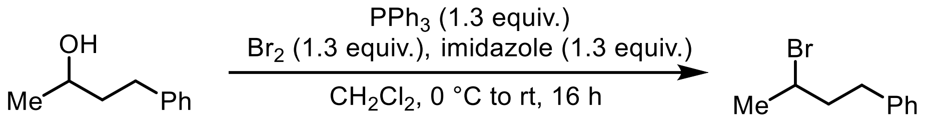

1 equiv.

To a round-bottomed flask, equipped with a magnetic stirrer, was added 4-phenylbutan-2-ol (0.62 mL, 4 mmol, 1 equiv.)), triphenylphosphine (1.36 g, 5.2 mmol, 1.3 equiv.), imidazole (0.35 g, 5.2 mmol, 1.3 equiv.) and CH_2Cl_2 (8 mL). Bromine (0.27 mL, 5.2 mmol, 1.3 equiv.) was added dropwise at 0 °C. The reaction was warmed to room temperature and stirred for 16 hours. The reaction mixture was quenched with saturated $NaHCO_3$ (10 mL). The layers were separated. The aqueous layer was extracted with CH_2Cl_2 (3 x 10 mL). The combined organic layers were washed with brine (25 mL), dried over magnesium sulfate, filtered, and concentrated *in vacuo*. The crude product was purified by FCC (100% PE) to give the title compound (0.77 g, 91%) as a colourless oil.

^{1}H NMR (500 MHz, $CDCl_3$) δ 7.33 – 7.27 (m, 2H), 7.23 – 7.19 (m, 3H), 4.13 – 4.05 (m, 1H), 2.90 – 2.84 (m, 1H), 2.79 – 2.72 (m, 1H), 2.19 – 2.10 (m, 1H), 2.10 – 2.00 (m, 1H), 1.74 (d, *J* = 6.7 Hz, 3H).

^{13}C {^{1}H} NMR (126 MHz, $CDCl_3$) δ 141.1, 128.7, 128.6, 126.2, 51.0, 42.8, 34.1, 26.7.

These data are consistent with the literature.[8]

2.2. Optimization of Model Reaction

Table S1. Optimization data for model ball-milled *N*-acyl imide XEC.

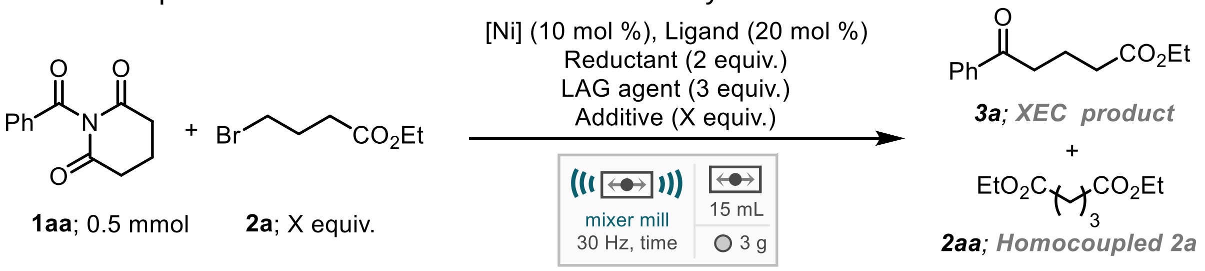

Entry	Equiv. of 2a	Catalyst	Ligand	Reductant (equiv.)	LAG Agent	Solid additive (equiv.)	Time (h)	Yield 3a (%)[a]	Yield 2aa (%)[a]
**1**	***1.5***	$NiCl_2.6H_2O$	**L2**	Zn (2)	DMA	-	2	10	42
**2**	***2***	$NiCl_2.6H_2O$	**L2**	Zn (2)	DMA	-	2	32	24
**3**	***2.5***	$NiCl_2.6H_2O$	**L2**	Zn (2)	DMA	-	2	31	30
**4**	***3***	$NiCl_2.6H_2O$	**L2**	Zn (2)	DMA	-	2	32	34
**5**	***3***	$NiCl_2.6H_2O$	**L2**	Zn (3)	DMA	-	2	30	28
**6**	2	$NiCl_2.6H_2O$	**L2**	Zn (2)	***DMA (5 equiv.)***	-	2	34	26
**7**	2	$NiCl_2.6H_2O$	**L2**	Zn (2)	***DMSO***	-	2	25	25
**8**	2	$NiCl_2.6H_2O$	**L2**	Zn (2)	***DMF***	-	2	8	17
**9**	2	$NiCl_2.6H_2O$	**L2**	Zn (2)	***NMP***	-	2	5	14
**10**	2	$NiCl_2.6H_2O$	**L2**	Zn (2)	***THF***	-	2	<2	<2
**11**	2	$NiCl_2.6H_2O$	**L2**	Zn (2)	***DCM***	-	2	<2	<2
**12**	2	$NiCl_2.6H_2O$	**L2**	Zn (2)	***MeCN***	-	2	<2	<2
**13**	2	$NiCl_2.6H_2O$	**L2**	Zn (2)	***PhMe***	-	2	<2	<2
**14**	2	$NiCl_2.6H_2O$	**L2**	Zn (2)	DMA	***LiBr (2)***	2	32	29
**15**	2	$NiCl_2.6H_2O$	**L2**	Zn (2)	DMA	***LiCl (2)***	2	19	<2
**16**	2	$NiCl_2.6H_2O$	**L2**	Zn (2)	DMA	***NaCl (2)***	2	55	32

**17**	2	$NiCl_2.6H_2O$	**L2**	Zn (2)	DMA	***NaBr (2)***	2	36	36
**18**	2	$NiCl_2.6H_2O$	**L2**	Zn (2)	DMA	***Na_2SO_4 (2)***	2	30	27
**19**	2	$NiCl_2.6H_2O$	**L2**	Zn (2)	DMA	***K_2SO_4 (2)***	2	29	28
**20**	2	$NiCl_2.6H_2O$	**L2**	Zn (2)	DMA	***NaCl (1)***	2	54	31
**21**	2	$NiCl_2.6H_2O$	**L2**	Zn (2)	DMA	***NaCl (0.5)***	2	43	28
**22**	2	$NiCl_2.6H_2O$	**L2**	***Zn (2)***	DMA	NaCl (0.5)	2	54	31
**23**	2	$NiCl_2.6H_2O$	**L2**	***Mn powder (2)***	DMA	NaCl (0.5)	2	54	2
**24**	2	$NiCl_2.6H_2O$	**L2**	***Mn pieces (2)***	DMA	NaCl (0.5)	2	48	3
**25**	2	$NiCl_2.6H_2O$	**L2**	***Mn powder (3)***	DMA	NaCl (0.5)	2	54	2
**26**	2	$NiCl_2.6H_2O$	***L2***	Mn powder (2)	DMA	NaCl (1)	2	54	<2
**27**	2	$NiCl_2.6H_2O$	***L3***	Mn powder (2)	DMA	NaCl (1)	2	46	<2
**28**	2	$NiCl_2.6H_2O$	***L4***	Mn powder (2)	DMA	NaCl (1)	2	62	<2
**29**	2	$NiCl_2.6H_2O$	***L5***	Mn powder (2)	DMA	NaCl (1)	2	76 (72)	<2
**30**	2	$NiCl_2.6H_2O$	***L6***	Mn powder (2)	DMA	NaCl (1)	2	70	<2
**31**	2	$NiCl_2.6H_2O$	***L7***	Mn powder (2)	DMA	NaCl (1)	2	14	<2
**32**	2	$NiCl_2.6H_2O$	***L8***	Mn powder (2)	DMA	NaCl (1)	2	<2	<2
**33**	2	$NiCl_2.6H_2O$	***-***	Mn powder (2)	DMA	NaCl (1)	2	<2	<2
**34**	2	***$NiCl_2$.dme***	L5	Mn powder (2)	DMA	NaCl (1)	2	24	<2
**35**	2	***$NiBr_2$.dme***	L5	Mn powder (2)	DMA	NaCl (1)	2	31	<2
**36**	2	***NiI_2***	L5	Mn powder (2)	DMA	NaCl (1)	2	42	<2
**37**	2	***$NiCl_2$ (anhyd.)***	L5	Mn powder (2)	DMA	NaCl (1)	2	16	<2
**38**	2	***-***	L5	Mn powder (2)	DMA	NaCl (1)	2	<2	<2
**39**	2	$NiCl_2.6H_2O$	L5	Mn powder (2)	DMA	NaCl (1)	***1***	59	<2
**40**	2	$NiCl_2.6H_2O$	L5	Mn powder (2)	DMA	NaCl (1)	***3***	74	<2
**41**	2	$NiCl_2.6H_2O$	L5	Mn powder (2)	DMA	NaCl (1)	***4***	75	<2
**42**	2	$NiCl_2.6H_2O$	L5	***-***	DMA	NaCl (1)	2	7	<2
**43**	2	$NiCl_2.6H_2O$	L5	Mn powder (2)	***-***	NaCl (1)	2	8	<2

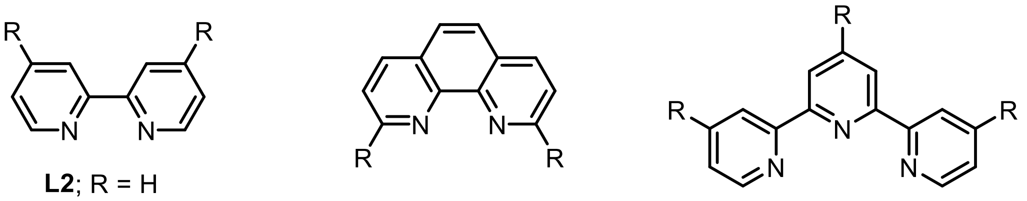

L2; R = H
L3; R = OMe
L4; R = *t*Bu

L5; R = H
L6; R = Me

L7; R = H
L8; R = Me

[a]Yield determined *via* analysis of the crude ^{1}H NMR spectra, using mesitylene as an internal standard. Isolated yields in parentheses.

2.3. General Procedure C – Mechanochemical XEC of *N*-Acyl Glutarimides with Alkyl Halides

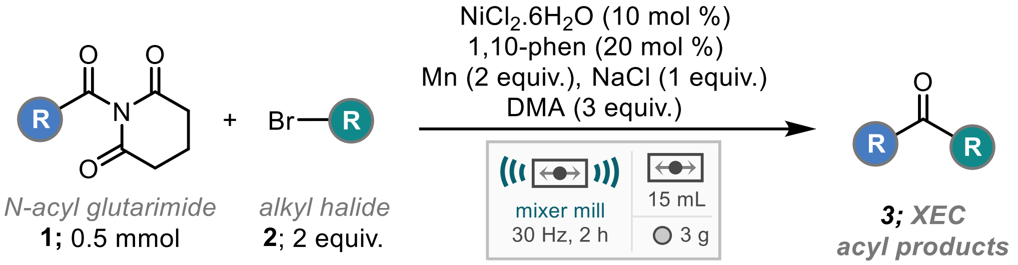

To a 15 mL stainless steel jar, equipped with a 3 g, 9 mm stainless steel ball, was charged *N*-Acyl glutarimide (0.5 mmol, 1 equiv.), alkyl bromide (1 mmol, 2 equiv.), nickel chloride hexahydrate (11.9 mg, 0.05 mmol, 10 mol %), 1,10-phenanthroline (18.0 mg, 0.1 mmol, 20 mol %), manganese powder (54.9 mg, 1 mmol, 2 equiv.) sodium chloride (29.2 mg, 0.5 mmol, 1 equiv.) and *N,N*-dimethylacetamide (139.0 µL, 1.5 mmol, 3 equiv.). The jar was closed and placed on the mixer mill to be milled at 30 Hz for 2 hours. Upon completion, the reaction mixture was transferred to a conical flask using EtOAc (10 mL). 1 M HCl (15 mL) was added, and the mixture was stirred for 3 minutes to quench metal salts. The mixture was then transferred to a separating funnel and the layers separated. The aqueous layer was extracted with EtOAc (2 x 15 mL). The combined organic layers were washed with brine (30 mL), dried over magnesium sulfate, filtered, and concentrated *in vacuo* to afford the crude product. The crude product was purified by FCC in the stated solvent system to afford the pure ketone product.

N.B. Unless stated otherwise, alkyl bromide used as alkyl halide coupling partner.

1-phenylnonan-1-one (3b)

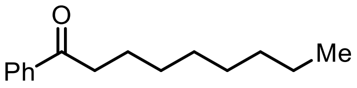

Prepared according to general procedure C using 1-iodoctane as the alkyl halide. Purified by FCC (5% Et_2O/hexane) to give the title compound (88 mg, 81%) as a colourless oil.

^{1}H NMR (500 MHz, $CDCl_3$) δ 7.97 – 7.94 (m, 2H), 7.57 – 7.53 (m, 1H), 7.48 – 7.44 (m, 2H), 2.98 – 2.93 (m, 2H), 1.79 – 1.69 (m, 2H), 1.41 – 1.24 (m, 10H), 0.88 (t, *J* = 7.0 Hz, 3H).

^{13}C {^{1}H} NMR (126 MHz, $CDCl_3$) δ 200.80, 137.26, 133.00, 128.69, 128.21, 38.80, 31.99, 29.60, 29.54, 29.32, 24.54, 22.81, 14.25.

These data are consistent with the literature.[9]

1,4-diphenylbutan-1-one (3c)

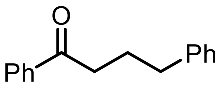

Prepared according to general procedure C. Purified by FCC (5 – 10% EtOAc/hexane) to give the title compound (87 mg, 78%) as a colourless oil.

^{1}H NMR (500 MHz, $CDCl_3$) δ 7.94 – 7.90 (m, 2H), 7.57 – 7.53 (m, 1H), 7.48 – 7.42 (m, 2H), 7.32 – 7.27 (m, 2H), 7.23 – 7.15 (m, 3H), 2.98 (t, *J* = 7.3 Hz, 2H), 2.76 – 2.66 (m, 2H), 2.12 – 2.05 (m, 2H).

^{13}C {^{1}H} NMR (126 MHz, $CDCl_3$) δ 200.3, 141.8, 137.2, 133.1, 128.7, 128.7, 128.6, 128.2, 126.1, 37.8, 35.3, 25.8.

These data are consistent with the literature.[6]

Ethyl 5-oxo-5-phenylpentanoate (3a)

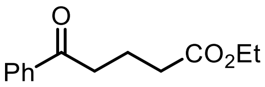

Prepared according to general procedure C. Purified by FCC (10 – 20% EtOAc/hexane) to give the title compound (79 mg, 72%) as a colourless oil.

^{1}H NMR (500 MHz, $CDCl_3$) δ 7.97 – 7.94 (m, 2H), 7.57 – 7.53 (m, 1H), 7.47 – 7.43 (m, 2H), 4.13 (q, *J* = 7.1 Hz, 2H), 3.05 (t, *J* = 7.2 Hz, 2H), 2.42 (t, *J* = 7.2 Hz, 2H), 2.06 (app p, *J* = 7.2 Hz, 2H), 1.24 (t, *J* = 7.1 Hz, 2H).

^{13}C {^{1}H} NMR (126 MHz, $CDCl_3$) δ 199.6, 173.4, 136.9, 133.2, 128.7, 128.1, 60.5, 37.6, 33.5, 19.5, 14.3.

These data are consistent with the literature.[10]

3-(4-fluorophenyl)-1-phenylpropan-1-one (3f)

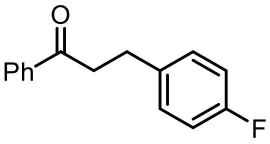

Prepared according to general procedure C. Purified by FCC (0 – 5% EtOAc/PE) to give the title compound (80 mg, 70%) as a white solid; m.p. 63 – 64 °C.

^{1}H NMR (500 MHz, $CDCl_3$) δ 7.97 – 7.93 (m, 2H), 7.59 – 7.54 (m, 1H), 7.48 – 7.43 (m, 2H), 7.23 – 7.18 (m, 2H), 7.00 – 6.94 (m, 2H), 3.28 (t, *J* = 7.6 Hz, 2H), 3.05 (t, *J* = 7.6 Hz, 2H).

^{13}C {^{1}H} NMR (126 MHz, $CDCl_3$) δ 199.2, 161.5 (d, *J* = 243.8 Hz), 137.0 (d, *J* = 3.2 Hz), 136.9, 133.3, 130.0 (d, *J* = 7.8 Hz), 128.8, 128.2, 115.4 (d, *J* = 21.1 Hz), 40.6, 29.4.

^{19}F NMR (471 MHz, $CDCl_3$) δ -117.31 – -117.23 (m).

These data are consistent with the literature.[11]

8-oxo-8-phenyloctanenitrile (3d)

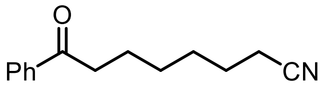

Prepared according to general procedure C. Purified by FCC (5 – 10% EtOAc/PE) to give the title compound (87 mg, 81%) as a colourless oil.

^{1}H NMR (500 MHz, $CDCl_3$) δ 7.97 – 7.94 (m, 2H), 7.59 – 7.54 (m, 1H), 7.49 – 7.44 (m, 2H), 2.98 (t, *J* = 7.2 Hz, 2H), 2.35 (t, *J* = 7.1 Hz, 2H), 1.76 (m, 2H), 1.69 (m, 2H), 1.55 – 1.47 (m, 2H), 1.48 – 1.38 (m, 2H).

^{13}C {^{1}H} NMR (126 MHz, $CDCl_3$) δ 200.3, 137.1, 133.2, 128.7, 128.2, 119.9, 38.4, 28.6, 28.6, 25.3, 23.9, 17.2.

These data are consistent with the literature.[10]

2-(4-oxo-4-phenylbutyl)isoindoline-1,3-dione (3e)

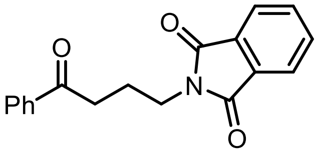

Prepared according to general procedure C. Purified by FCC (10 – 30% EtOAc/PE) to give the title compound (89 mg, 61%) as a white solid; m.p. 126 – 128 °C

^{1}H NMR (500 MHz, $CDCl_3$) δ 7.94 – 7.90 (m, 2H), 7.84 (dd, *J* = 5.4, 3.0 Hz, 2H), 7.71 (dd, *J* = 5.5, 3.0 Hz, 2H), 7.56 – 7.51 (m, 1H), 7.46 – 7.41 (m, 2H), 3.82 (t, *J* = 6.8 Hz, 2H), 3.06 (t, *J* = 7.3 Hz, 2H), 2.15 (app p, *J* = 7.0 Hz, 2H).

^{13}C {^{1}H} NMR (126 MHz, $CDCl_3$) δ 199.0, 168.6, 136.9, 134.1, 133.2, 132.2, 128.7, 128.1, 123.4, 37.6, 35.9, 23.3.

These data are consistent with the literature.[12]

Cyclohexyl(phenyl)methanone (3g)

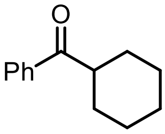

Prepared according to general procedure C. Prepared according to general procedure C with a 3 hour reaction time. Purified by FCC (0 – 5% EtOAc/PE) to give the title compound (45 mg, 48%) as a white solid; m.p. 53 – 56 °C.

^{1}H NMR (500 MHz, $CDCl_3$) δ 7.96 – 7.92 (m, 2H), 7.56 – 7.52 (m, 1H), 7.49 – 7.43 (m, 2H), 3.26 (tt, *J* = 11.5, 3.3 Hz, 1H), 1.93 – 1.81 (m, 3H), 1.78 – 1.70 (m, 1H), 1.56 – 1.25 (m, 5H).

^{13}C {^{1}H} NMR (126 MHz, $CDCl_3$) δ 204.1, 136.5, 132.9, 128.7, 128.4, 45.8, 29.6, 26.1, 26.0.

These data are consistent with the literature.[6]

(*S*)-4,8-dimethyl-1-phenylnon-7-en-1-one (3h)

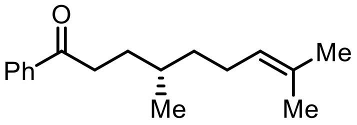

Prepared according to general procedure C. Purified by FCC (0 – 5% EtOA/PE) to give the title compound (68 mg, 56%) as a colourless oil.

^{1}H NMR (500 MHz, $CDCl_3$) δ 7.98 – 7.95 (m, 2H), 7.57 – 7.53 (m, 1H), 7.49 – 7.44 (m, 2H), 5.13 – 5.07 (m, 1H), 3.04 – 2.88 (m, 2H), 2.06 – 1.93 (m, 2H), 1.81 – 1.74 (m, 1H), 1.68 (d, *J* = 1.1 Hz, 3H), 1.60 (s, 3H), 1.59 – 1.51 (m, 2H), 1.43 – 1.35 (m, 1H), 1.25 – 1.16 (m, 1H), 0.94 (d, *J* = 6.5 Hz, 3H).

^{13}C {^{1}H} NMR (126 MHz, $CDCl_3$) δ 201.0, 137.2, 133.0, 131.5, 128.7, 128.2, 124.8, 37.0, 36.5, 32.4, 31.5, 25.9, 25.7, 19.6, 17.8.

These data are consistent with the literature.[13]

Oxetan-3-yl(phenyl)methanone (3i)

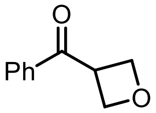

Prepared according to general procedure C with a 3 hour reaction time. Purified by FCC (10 – 30% EtOAc/PE) to give the title compound (68 mg, 84%) as a colourless oil.

^{1}H NMR (500 MHz, $CDCl_3$) δ 7.80 – 7.77 (m, 2H), 7.62 – 7.57 (m, 1H), 7.50 – 7.45 (m, 2H), 4.97 (d, *J* = 7.8 Hz, 4H), 4.64 (p, *J* = 7.8 Hz, 1H).

^{13}C {^{1}H} NMR (126 MHz, $CDCl_3$) δ 197.2, 135.0, 133.8, 129.1, 128.3, 72.9, 42.3.

HRMS (ES) m/z: $[M+H]^+$ Calcd. for $C_{10}H_{11}O$ 163.0759, found: 163.0753.

FTIR (film) v_{max} (cm^{-1}): 2914, 2854, 1672, 1583, 1212, 1080, 801.

***Tert*-butyl 4-benzoylpiperidine-1-carboxylate (3j)**

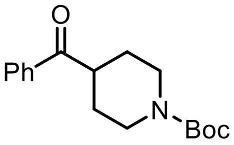

Prepared according to general procedure C with a 3 hour reaction time. Purified by FCC (5 – 20% EtOAc/PE) to give the title compound (93 mg, 64%) as a white solid; m.p. 93 – 95 °C.

^{1}H NMR (500 MHz, $CDCl_3$) δ 7.96 – 7.92 (m, 2H), 7.59 – 7.55 (m, 1H), 7.50 – 7.45 (m, 2H), 4.20 – 4.13 (m, 2H), 3.44 – 3.37 (m, 1H), 2.93 – 2.86 (m, 2H), 1.87 – 1.81 (m, 2H), 1.74 – 1.64 (m, 2H), 1.46 (s, 9H).

^{13}C {^{1}H} NMR (126 MHz, $CDCl_3$) δ 202.2, 154.6, 136.0, 133.3, 128.9, 128.4, 79.8, 43.6, 43.4, 28.6, 28.5.

These data are consistent with the literature.[10]

Ethyl 5-oxo-5-(*p*-tolyl)pentanoate (3l)

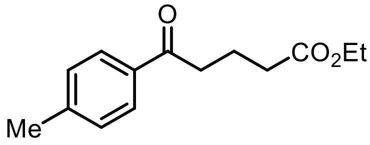

Prepared according to general procedure C. Purified by FCC (2 – 5% EtOAc/PE) to give the title compound (98 mg, 84%) as a white solid; m.p 37 – 38 °C.

^{1}H NMR (500 MHz, $CDCl_3$) δ 7.88 – 7.82 (m, 2H), 7.26 – 7.22 (m, 2H), 4.13 (q, *J* = 7.1 Hz, 2H), 3.02 (t, *J* = 7.2 Hz, 2H), 2.43 – 2.39 (m, 5H), 2.06 (app p, *J* = 7.2 Hz, 2H), 1.25 (t, *J* = 7.1 Hz, 3H).

^{13}C {^{1}H} NMR (126 MHz, $CDCl_3$) δ 199.2, 173.4, 144.0, 134.5, 129.4, 128.3, 60.5, 37.5, 33.6, 21.8, 19.6, 14.4.

These data are consistent with the literature.[14]

Ethyl 5-(4-methoxyphenyl)-5-oxopentanoate (3m)

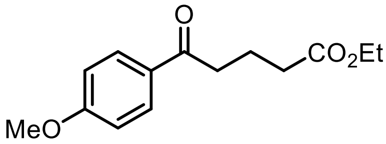

Prepared according to general procedure C. Purified by FCC (5 – 20% EtOAc/PE) to give the title compound (111 mg, 89%) as a white solid; m.p. 54 – 56 °C.

^{1}H NMR (500 MHz, $CDCl_3$) δ 7.97 – 7.91 (m, 2H), 6.94 – 6.90 (m, 2H), 4.13 (q, *J* = 7.1 Hz, 2H), 3.86 (s, 3H), 2.99 (t, *J* = 7.2 Hz, 2H), 2.42 (t, *J* = 7.2 Hz, 2H), 2.05 (p, *J* = 7.2 Hz, 2H), 1.25 (t, *J* = 7.1 Hz, 3H).

^{13}C {^{1}H} NMR (126 MHz, $CDCl_3$) δ 198.2, 173.5, 163.6, 130.4, 130.1, 113.8, 60.5, 55.6, 37.3, 33.6, 19.8, 14.4.

These data are consistent with the literature.[15]

Ethyl 5-(4-fluorophenyl)-5-oxopentanoate (3n)

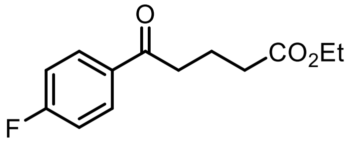

Prepared according to general procedure C. Purified by FCC (5 – 10% EtOAc/PE) to give the title compound (83 mg, 70%) as a colourless oil.

^{1}H NMR (500 MHz, $CDCl_3$) δ 8.02 – 7.95 (m, 2H), 7.15 – 7.10 (m, 2H), 4.14 (q, *J* = 7.1 Hz, 2H), 3.02 (t, *J* = 7.2 Hz, 2H), 2.43 (t, *J* = 7.1 Hz, 2H), 2.06 (p, *J* = 7.2 Hz, 2H), 1.25 (t, *J* = 7.1 Hz, 3H).

^{13}C {^{1}H} NMR (126 MHz, $CDCl_3$) δ 198.0, 173.4, 165.9 (d, *J* = 254.6 Hz), 133.4 (d, *J* = 3.0 Hz), 130.8 (d, *J* = 9.3 Hz), 115.8 (d, *J* = 21.8 Hz), 60.6, 37.5, 33.5, 19.5, 14.4.

^{19}F NMR (471 MHz, $CDCl_3$) δ -105.37 – -105.30 (m).

These data are consistent with the literature.[16]

Ethyl 5-(4-chlorophenyl)-5-oxopentanoate (3o)

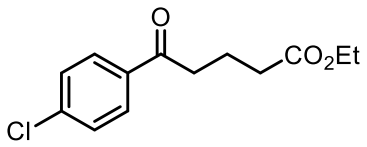

Prepared according to general procedure C. Purified by FCC (2 – 5% EtOAc/PE) to give the title compound (86 mg, 68%) as a white solid; m.p. 50 – 52 °C.

^{1}H NMR (500 MHz, $CDCl_3$) δ 7.92 – 7.88 (m, 2H), 7.45 – 7.41 (m, 2H), 4.14 (q, *J* = 7.1 Hz, 2H), 3.02 (t, *J* = 7.2 Hz, 2H), 2.43 (m, 2H), 2.06 (app p, *J* = 7.2 Hz, 2H), 1.25 (t, *J* = 7.2 Hz, 3H).

^{13}C {^{1}H} NMR (126 MHz, $CDCl_3$) δ 198.4, 173.4, 139.7, 135.3, 129.6, 129.1, 60.6, 37.6, 33.4, 19.4, 14.4.

These data are consistent with the literature.[17]

Methyl 4-(5-ethoxy-5-oxopentanoyl)benzoate (3q)

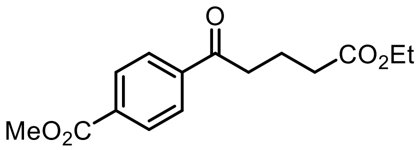

Prepared according to general procedure C. Purified by FCC (10 – 20% EtOAc/PE) to give the title compound (104 mg, 75%) as a white solid; m.p. 96 – 98 °C.

^{1}H NMR (500 MHz, $CDCl_3$) δ 8.13 – 8.10 (m, 2H), 8.02 – 7.99 (m, 2H), 4.14 (q, *J* = 7.1 Hz, 2H), 3.95 (s, 3H), 3.08 (t, *J* = 7.2 Hz, 2H), 2.44 (t, *J* = 7.1 Hz, 2H), 2.08 (app p, *J* = 7.2 Hz, 2H), 1.25 (t, *J* = 7.1 Hz, 3H).

^{13}C {^{1}H} NMR (126 MHz, $CDCl_3$) δ 199.1, 173.4, 166.4, 140.1, 134.0, 130.0, 128.1, 60.6, 52.6, 38.0, 33.4, 19.4, 14.4.

HRMS (ES) m/z: $[MH]^+$ Calcd for $C_{15}H_{19}O_5$ 279.1232, found: 279.1227.

FTIR (film) ν_{max} (cm^{-1}): 2930, 2842, 1731, 1680, 1262, 1196, 692.

Ethyl-5-oxo-5-(*o*-tolyl)pentanoate (3p)

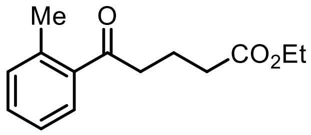

Prepared according to general procedure C. Purified by FCC (10 – 20% EtOAc/PE) to give the title compound (84 mg, 72%) as a colourless oil.

^{1}H NMR (500 MHz, $CDCl_3$) δ 7.63 (d, *J* = 7.8 Hz, 1H), 7.36 (td, *J* = 7.5, 1.3 Hz, 1H), 7.27 – 7.22 (m, 2H), 4.13 (q, *J* = 7.1 Hz, 2H), 2.97 (t, *J* = 7.2 Hz, 2H), 2.49 (s, 3H), 2.41 (t, *J* = 7.2 Hz, 2H), 2.04 (p, *J* = 7.2 Hz, 2H), 1.25 (t, *J* = 7.1 Hz, 3H).

^{13}C {^{1}H} NMR (126 MHz, $CDCl_3$) δ 203.7, 173.4, 138.2, 138.0, 132.1, 131.4, 128.6, 125.8, 60.5, 40.5, 33.6, 21.4, 19.7, 14.4.

These data are consistent with the literature.[17]

Ethyl 5-(naphthalen-2-yl)-5-oxopentanoate (3r)

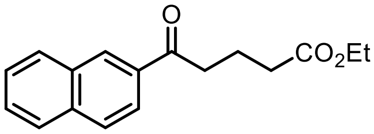

Prepared according to general procedure C. Purified by FCC (10 – 25% EtOAc/PE) to give the title compound (86 mg, 64%) as a white solid; m.p. 61 – 62 °C.

^{1}H NMR (500 MHz, $CDCl_3$) δ 8.48 (s, 1H), 8.03 (dd, *J* = 8.6, 1.7 Hz, 1H), 7.96 (d, *J* = 8.0 Hz, 1H), 7.91 – 7.86 (m, 2H), 7.63 – 7.53 (m, 2H), 4.15 (q, *J* = 7.1 Hz, 2H), 3.19 (t, *J* = 7.2 Hz, 2H), 2.48 (t, *J* = 7.2 Hz, 2H), 2.14 (p, *J* = 7.2 Hz, 2H), 1.26 (t, *J* = 7.1 Hz, 3H).

^{13}C {^{1}H} NMR (126 MHz, $CDCl_3$) δ 199.6, 173.5, 135.7, 134.3, 132.7, 129.9, 129.7, 128.6, 128.6, 127.9, 126.9, 124.0, 60.5, 37.7, 33.6, 19.7, 14.4.

These data are consistent with the literature.[18]

Ethyl 5-(furan-2-yl)-5-oxopentanoate (3s)

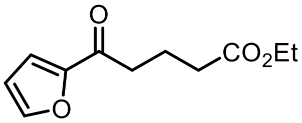

Prepared according to general procedure C. Purified by FCC (10% EtOAc/PE) to give the title compound (61 mg, 58%) as a colourless oil.

^{1}H NMR (500 MHz, $CDCl_3$) δ 7.59 – 7.56 (m, 1H), 7.20 (d, *J* = 3.6 Hz, 1H), 6.53 (dd, *J* = 3.5, 1.7 Hz, 1H), 4.13 (q, *J* = 7.1 Hz, 2H), 2.90 (t, *J* = 7.3 Hz, 2H), 2.41 (t, *J* = 7.2 Hz, 2H), 2.05 (p, *J* = 7.3 Hz, 2H), 1.25 (t, *J* = 7.1 Hz, 3H).

^{13}C {^{1}H} NMR (126 MHz, $CDCl_3$) δ 188.7, 173.2, 152.6, 146.3, 117.0, 112.2, 60.4, 37.3, 33.4, 19.3, 14.2.

These data are consistent with the literature.[17]

1-cyclohexylnonan-1-one (3u)

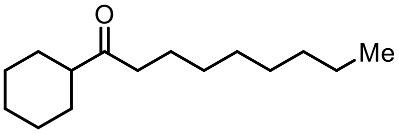

Prepared according to general procedure C. Purified by FCC (0 – 5% EtOAc/PE, PMA stain for TLC visualisation) to give the title compound (57 mg, 51%) as a colourless oil.

^{1}H NMR (500 MHz, $CDCl_3$) δ 2.43 – 2.29 (m, 3H), 1.86 – 1.73 (m, 3H), 1.70 – 1.63 (m, 1H), 1.57 – 1.51 (m, 2H), 1.37 – 1.16 (m, 15H), 0.87 (t, *J* = 6.9 Hz, 3H).

^{13}C {^{1}H} NMR (126 MHz, $CDCl_3$) δ 214.7, 51.0, 43.0, 40.8, 32.0, 29.6, 29.5, 29.3, 28.7, 26.0, 25.9, 23.9, 22.8, 14.2.

These data are consistent with the literature.[19]

1-cyclohexyl-3-(4-fluorophenyl)propan-1-one (3v)

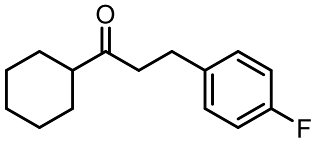

Prepared according to general procedure C. Purified by FCC (0 – 5% EtOAc/PE) to give the title compound (49 mg, 42%) as a colourless oil.

^{1}H NMR (500 MHz, $CDCl_3$) δ 7.14 – 7.10 (m, 2H), 6.98 – 6.92 (m, 2H), 2.85 (t, *J* = 7.5 Hz, 2H), 2.73 (t, *J* = 7.4 Hz, 2H), 2.33 – 2.25 (m, 1H), 1.82 – 1.72 (m, 3H), 1.68 – 1.58 (m, 2H), 1.35 – 1.15 (m, 5H).

^{13}C {^{1}H} NMR (126 MHz, $CDCl_3$) δ 213.1, 161.5 (d, *J* = 243.6 Hz), 137.2 (d, *J* = 3.2 Hz), 129.9 (d, *J* = 7.8 Hz), 115.3 (d, *J* = 21.1 Hz), 51.1, 42.4, 29.0, 28.5, 26.0, 25.8.

^{19}F NMR (471 MHz, $CDCl_3$) δ -117.50 – -117.42 (m).

These data are consistent with the literature.[20]

(*S*)-1-cyclohexyl-4,8-dimethylnon-7-en-1-one (3w)

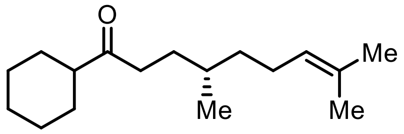

Prepared according to general procedure C. Purified by FCC (0 – 5% EtOAc/PE) to give the title compound (59 mg, 47%) as a colourless oil.

^{1}H NMR (500 MHz, $CDCl_3$) δ 5.11 – 5.05 (m, 1H), 2.49 – 2.30 (m, 3H), 2.04 – 1.89 (m, 2H), 1.86 – 1.73 (m, 3H), 1.70 – 1.54 (m, 8H), 1.44 – 1.11 (m, 10H), 0.87 (d, *J* = 6.2 Hz, 3H).

^{13}C {^{1}H} NMR (126 MHz, $CDCl_3$) δ 214.8, 131.4, 124.9, 51.0, 38.5, 37.0, 32.3, 30.8, 28.7, 28.7, 26.0, 25.9, 25.6, 19.5, 17.8.

HRMS (EI) m/z: $[M]^+$ Calcd for $C_{17}H_{39}O$ 250.2291, found: 250.2293.

FTIR (film) ν_{max} (cm^{-1}): 2926, 2854, 1706, 1450, 1377, 1145, 987, 822.

1-phenylundecan-4-one (3t)

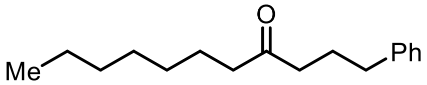

Prepared according to general procedure C. Purified by FCC (0 – 2% EtOAc/PE) to give the title compound (60 mg, 49%) as a colourless oil.

^{1}H NMR (500 MHz, $CDCl_3$) δ 7.30 – 7.27 (m, 2H), 7.21 – 7.15 (m, 3H), 2.64 – 2.59 (m, 2H), 2.41 (t, *J* = 7.4 Hz, 2H), 2.36 (t, *J* = 7.5 Hz, 2H), 1.96 – 1.87 (m, 2H), 1.59 – 1.50 (m, 2H), 1.32 – 1.22 (m, 8H), 0.88 (t, *J* = 7.0 Hz, 3H).

^{13}C {^{1}H} NMR (126 MHz, $CDCl_3$) δ 211.3, 141.8, 128.6, 128.5, 126.0, 43.0, 42.0, 35.3, 31.8, 29.3, 29.2, 25.4, 24.0, 22.7, 14.2.

These data are consistent with the literature.[21]

2,2-dimethyl-6-phenylhexan-3-one (3x)

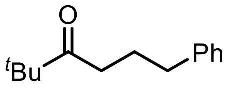

Prepared according to general procedure C. Purified by FCC (0 – 3% EtOAc/PE) to give the title compound (33 mg, 32%) as a colourless oil.

^{1}H NMR (500 MHz, $CDCl_3$) δ 7.30 – 7.26 (m, 2H), 7.21 – 7.16 (m, 3H), 2.61 (t, *J* = 7.4 Hz, 2H), 2.50 (t, *J* = 7.2 Hz, 2H), 1.90 (p, *J* = 7.4 Hz, 2H), 1.12 (s, 9H).

^{13}C {^{1}H} NMR (126 MHz, $CDCl_3$) δ 215.9, 142.0, 128.6, 128.5, 126.0, 44.26, 35.8, 35.3, 26.6, 25.5.

These data are consistent with the literature.[22]

2-methyl-1,4-diphenylbutan-1-one (3k)

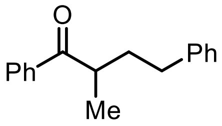

Prepared according to general procedure C with a reaction time of 3 hours. Purified by FCC (0 – 5% EtOAc/PE) to give the title compound (61 mg, 51%) as a colourless oil.

^{1}H NMR (500 MHz, $CDCl_3$) δ 7.89 – 7.85 (m, 2H), 7.59 – 7.51 (m, 1H), 7.47 – 7.41 (m, 2H), 7.29 – 7.25 (m, 2H), 7.22 – 7.17 (m, 1H), 7.17 – 7.13 (m, 2H), 3.47 (app h, *J* = 6.8 Hz, 1H), 2.69 – 2.60 (m, 2H), 2.21 – 2.13 (m, 1H), 1.79 – 1.70 (m, 1H), 1.24 (d, *J* = 6.9 Hz, 3H).

^{13}C {^{1}H} NMR (126 MHz, $CDCl_3$) δ 204.3, 141.9, 136.6, 133.1, 128.8, 128.6, 128.5, 128.4, 126.1, 39.8, 35.3, 33.6, 17.4.

These data are consistent with the literature.[23]

2.4. Scale-Up Experiment

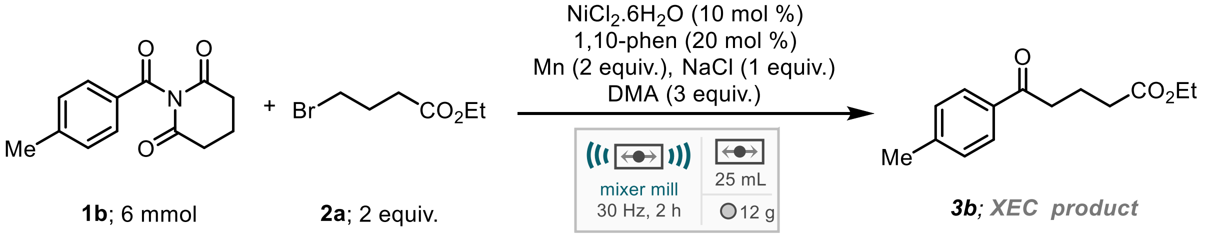

To a 25 mL stainless steel jar, equipped with a 12 g, 14 mm stainless steel ball, was charged nickel chloride hexahydrate (143 mg, 0.6 mmol, 10 mol %), 1,10-phenanthroline (216 mg, 1.2 mmol, 20 mol %), manganese powder (659 mg, 12 mmol, 2 equiv.), sodium chloride (351 mg, 12 mmol, 1 equiv.), and 1-(4-methylbenzoyl)piperidine-2,6-dione (1.41 g, 6 mmol, 1 equiv.). Followed by ethyl 4-bromobutyrate (1.72 mL, 12 mmol, 2 equiv.) and *N*,*N*-dimethylacetamide (1.67 mL, 18 mmol, 3 equiv.). The jar was closed and placed on the mixer mill, to be milled at 30 Hz for 2 hours. Upon completion, the reaction mixture was transferred to a conical flask using EtOAc (50 mL). 1M HCl (50 mL) was added, and the mixture was stirred for 3 minutes to quench metal salts. The mixture was then transferred to a separating funnel and the layers separated. The aqueous layer was extracted with EtOAc (2 x 50 mL). The combined organic layers were washed with brine (50 mL), dried over magnesium sulfate, filtered, and concentrated *in vacuo* to afford the crude product. The crude product was purified by FCC (2 – 5% EtOAc/PE) to give **ethyl 5-oxo-5-(*p*-tolyl)pentanoate** (1.06 g, 75%) as a white solid.

2.5. Mechanistic Studies

2.5.1. Radical Clock Experiment

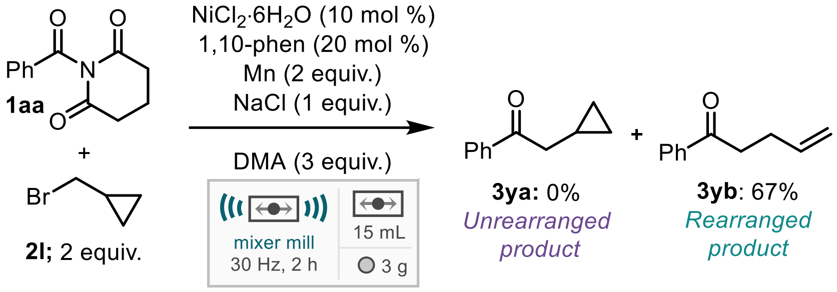

To a 15 mL stainless steel jar, equipped with a 3 g, 9 mm stainless steel ball, was charged 1-benzoylpiperidine-2,6-dione (109 mg, 0.5 mmol, 1 equiv.), (bromomethyl)cyclopropane (97.0 μL, 1 mmol, 2 equiv.), nickel chloride hexahydrate (11.9 mg, 0.05 mmol, 10 mol %), 1,10-phenanthroline (18.0 mg, 0.1 mmol, 20 mol %), manganese powder (54.9 mg, 1 mmol, 2 equiv.) sodium chloride (29.2 mg, 0.5 mmol, 1 equiv.) and *N,N*-dimethylacetamide (139 μL, 1.5 mmol, 3 equiv.). The jar was closed and placed on the mixer mill to be milled at 30 Hz for 2 hours. Upon completion, the mixture was washed from the jar with CH_2Cl_2 (20 mL). 1 M HCl (20 mL) was added, and the layers separated. The aqueous layer was extracted with CH_2Cl_2 (2 x 20 mL). The combined organic layers were washed with brine, dried over magnesium sulfate and filtered. The mixture was concentrated *in vacuo* to give the crude product. The crude product was purified using FCC (0 – 5% EtOAc/PE) to give the rearranged product **3yb** (54 mg, 67%) as a colourless oil.

^{1}H NMR (500 MHz, $CDCl_3$) δ 7.99 – 7.94 (m, 2H), 7.58 – 7.53 (m, 1H), 7.49 – 7.44 (m, 2H), 5.91 (ddt, *J* = 16.8, 10.2, 6.5 Hz, 1H), 5.13 – 4.98 (m, 2H), 3.11 – 3.06 (m, 2H), 2.53 – 2.48 (m, 2H).

^{13}C {^{1}H} NMR (126 MHz, $CDCl_3$) δ 199.60, 137.45, 137.07, 133.16, 128.74, 128.17, 115.43, 37.89, 28.29.

These data are consistent with the literature.[24]

2.5.2. Radical Trapping Experiment

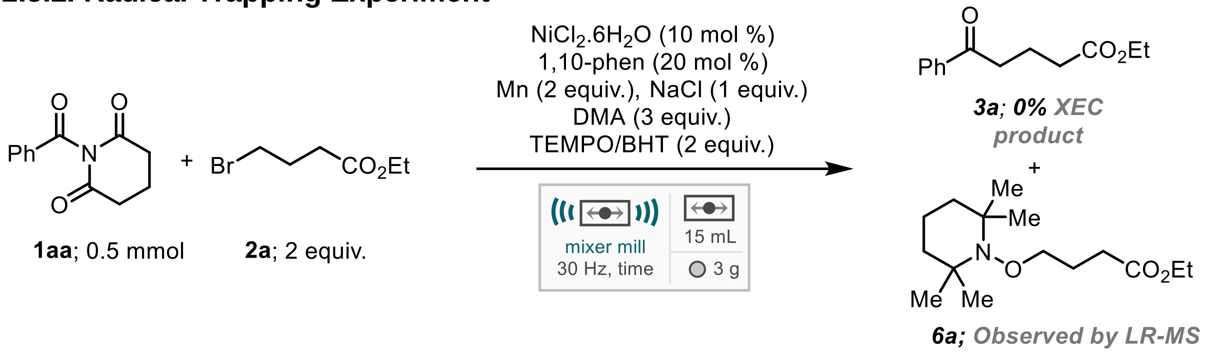

To a 15 mL stainless steel jar, equipped with a 3 g, 9 mm stainless steel ball, was charged 1-benzoylpiperidine-2,6-dione (109 mg, 0.5 mmol, 1 equiv.), ethyl 4-bromobutyrate (143 µL, 1 mmol, 2 equiv.), nickel chloride hexahydrate (11.9 mg, 0.05 mmol, 10 mol %), 1,10-phenanthroline (18.0 mg, 0.1 mmol, 20 mol %), manganese powder (54.9 mg, 1 mmol, 2 equiv.)), sodium chloride (29.2 g, 0.5 mmol, 1 equiv.), *N,N*-dimethylacetamide (139 µL, 1.5 mmol, 3 equiv.) and either TEMPO (156 mg, 1 mmol, 2 equiv.) or BHT (220.4 mg, 1 mmol, 2 equiv.). The jar was closed and placed on the mixer mill to be milled at 30 Hz for 2 hours. Upon completion, the mixture was washed from the jar with CH_2Cl_2 (20 mL) and filtered through a short pad of Celite. After concentrating *in vacuo*, the crude material was analysed *via* ^{1}H NMR and LR-MS to determine the presence of product (**3a**) and/or TEMPO adduct(s).

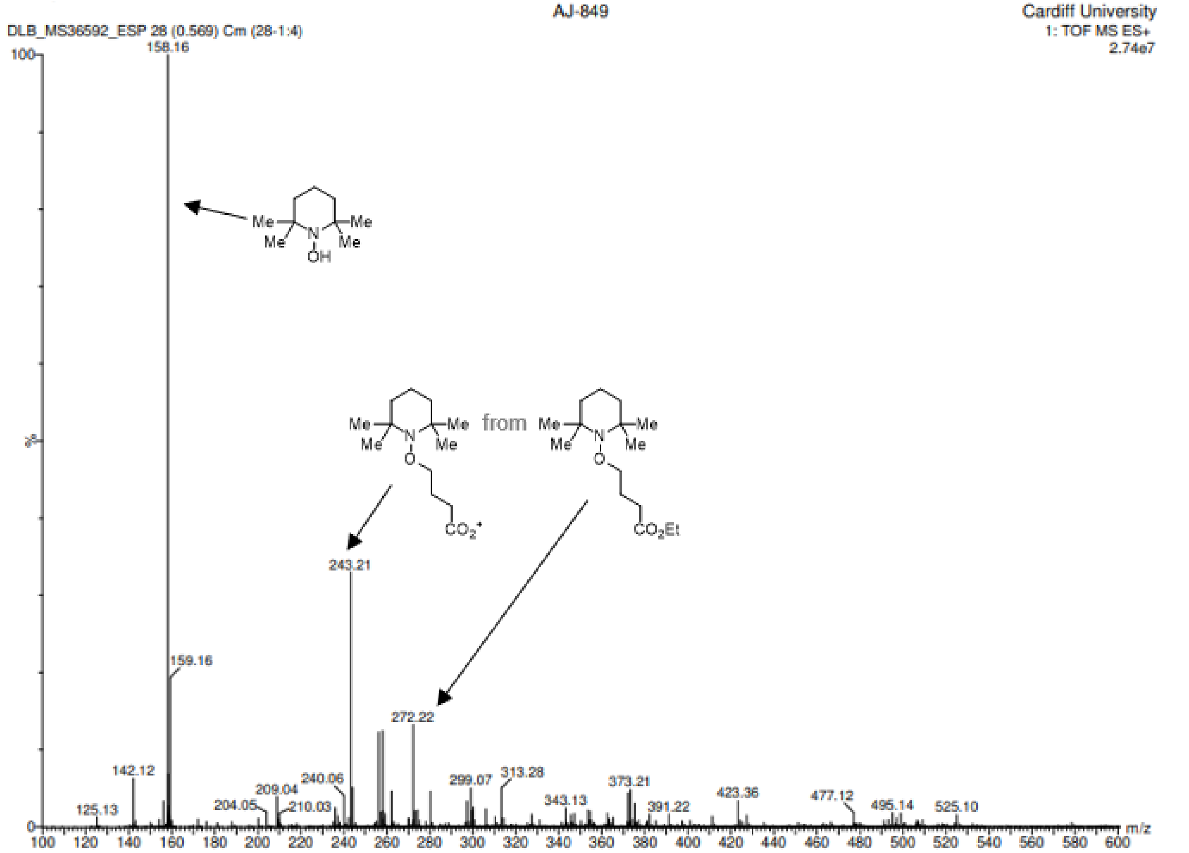

3.1. Mechanochemical XEC of Heteroaryl Halides

3.1.1. General Procedure D – Synthesis of Amidine Ligands

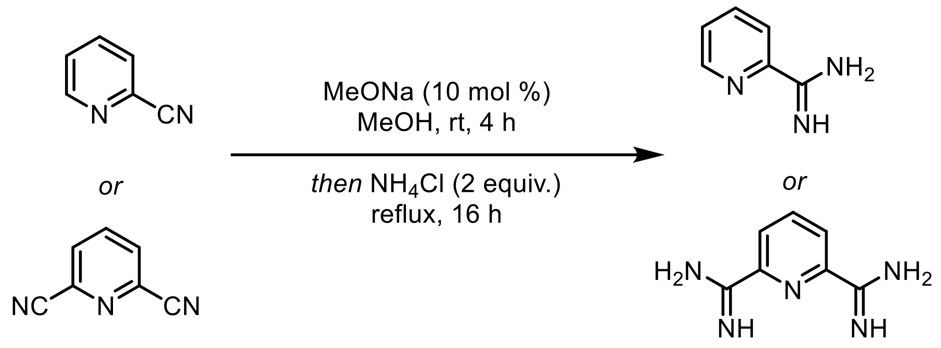

1 equiv.

To a round-bottomed flask, equipped with a magnetic stirrer, was charged 2-pyridinecarbonitrile or 2,6-pyridinecarbonitrile (1 equiv.) and methanol (0.5 M), under a nitrogen atmosphere. Sodium methoxide (0.5 M solution in methanol, 0.1 equiv.) was added, and the resulting mixture was stirred at room temperature for 4 hours. Ammonium chloride (2 equiv.) was then added in one portion and the reaction mixture was heated to reflux and stirred for 16 hours. After cooling to room temperature, the formed precipitate was filtered under suction and concentrated *in vacuo*. The resulting crude compound was heated in ethanol (1 mL/mmol), then filtered while hot to yield the product.

Picolinimidamide hydrogen chloride (L1)

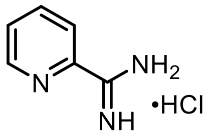

Prepared according to general procedure D, on a 20 mmol scale, to give the title compound (3.14 g, 99%) as a white solid; m.p. 90 – 92 °C.

^{1}H NMR (500 MHz, $(CD_3)_2SO$) δ 8.82 (d, J = 4.2 Hz, 1H), 8.51 (br s, 4H), 8.43 (d, J = 7.9 Hz, 1H), 8.16 (td, J = 7.8, 1.4 Hz, 1H), 7.79 (dd, J = 7.3, 4.8 Hz, 1H).

^{13}C {^{1}H} APT NMR (126 MHz, $(CD_3)_2SO$) δ 162.2, 150.0, 144.0, 138.4, 128.6, 123.6.

These data are consistent with the literature.[25]

Pyridine-2,6-bis(carboximidamide) dihydrogen chloride (L9)

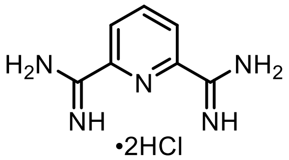

Prepared according to general procedure D, on a 7.7 mmol scale, to give the title compound (1.83 g, >98%) as a pale-yellow solid; m.p. >300 °C (decomp.).

^{1}H NMR (500 MHz, $(CD_3)_2SO$) δ 9.79 (s, 8H), 8.81 (d, *J* = 8.0 Hz, 2H), 8.53 (t, *J* = 8.0 Hz, 1H).

^{13}C {^{1}H} APT NMR (126 MHz, $(CD_3)_2SO$) δ 160.4, 144.1, 140.8, 127.3.

These data are consistent with the literature.[25]

3.1.2. Optimisation of Model Reaction

Table S2. Variation of Catalyst/Ligand Ratio and Alkyl Halide Equivalents.

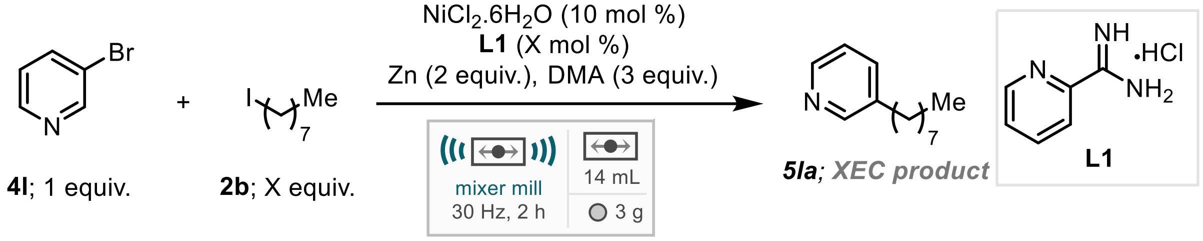

Entry	Ni:L1 ratio	2b (X equiv.)	4l consumed (%)[a]	5la (%)[a]
**1**	*1:1*	2	74	41
**2**	*1:2*	2	91	58
**3**	*1:4*	2	83	36
**4**	1:2	*1.1*	92	31
**5**	1:2	*1.5*	91	45
**6**	1:2	*3*	80	48

[a]Yield determined *via* analysis of the crude ^{1}H NMR spectra, using mesitylene as an internal standard.

Table S3. Optimization data for model ball-mill XEC of heteroaryl halides.

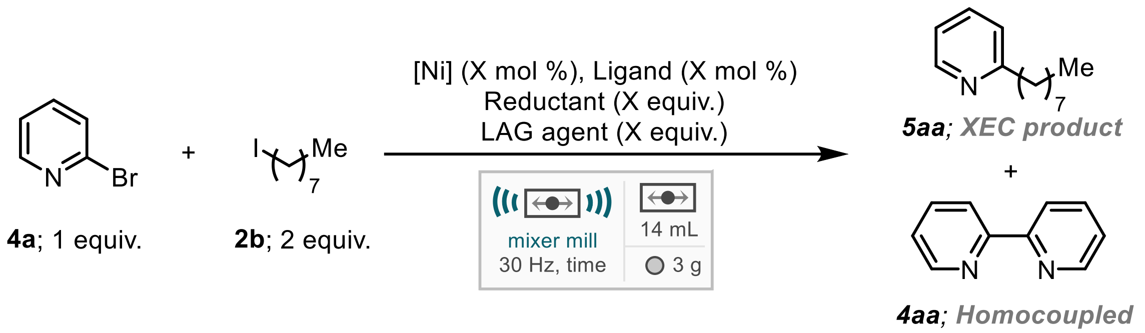

Entry	[Ni] (mol %)	Ligand (mol %)	Reductant (equiv.)	LAG (equiv.)	Time (h)	5aa (%)[a]	4aa (%)[a]
**1**	NiCl$_2$•6H$_2$O (10)	**L1** (20)	***Zn (1.2)***	DMA (3)	2	26	<2
**2**	NiCl$_2$•6H$_2$O (10)	**L1** (20)	***Zn (2.0)***	DMA (3)	2	65	<2
**3**	NiCl$_2$•6H$_2$O (10)	**L1** (20)	***Zn (2.5)***	DMA (3)	2	79 (72)	<2
**4**	NiCl$_2$•6H$_2$O (10)	**L1** (20)	***Zn (3.0)***	DMA (3)	2	72	<2
**5**	NiCl$_2$•6H$_2$O (10)	***L2 (20)***	Zn (2.5)	DMA (3)	2	38	10
**6**	NiCl$_2$•6H$_2$O (10)	***L5 (20)***	Zn (2.5)	DMA (3)	2	34	3
**7**	NiCl$_2$•6H$_2$O (10)	***L7 (20)***	Zn (2.5)	DMA (3)	2	27	5
**8**	NiCl$_2$•6H$_2$O (10)	***L9 (20)***	Zn (2.5)	DMA (3)	2	63	<2
**9**	***NiCl$_2$(DME) (10)***	**L1** (20)	Zn (2.5)	DMA (3)	2	69	4
**10**	***NiBr$_2$(DME) (10)***	**L1** (20)	Zn (2.5)	DMA (3)	2	70	3
**11**	NiCl$_2$•6H$_2$O (10)	**L1** (20)	***Mn (2.5)***	DMA (3)	2	52	11
**12**	NiCl$_2$•6H$_2$O (10)	**L1** (20)	***TDAE (2.5)***	DMA (3)	2	<2	<2
**13**	NiCl$_2$•6H$_2$O (10)	**L1** (20)	Zn (2.5)	***NMP (3)***	2	64	5
**14**	NiCl$_2$•6H$_2$O (10)	**L1** (20)	Zn (2.5)	***DMF (3)***	2	62	5
**15**	NiCl$_2$•6H$_2$O (10)	**L1** (20)	Zn (2.5)	***-***	2	7	6
**16**	NiCl$_2$•6H$_2$O (10)	**L1** (20)	Zn (2.5)	***DMA (1)***	2	27	7
**17**	NiCl$_2$•6H$_2$O (10)	**L1** (20)	Zn (2.5)	***DMA (4)***	2	77	<2
**18**	NiCl$_2$•6H$_2$O (10)	**L1** (20)	Zn (2.5)	DMA (3)	***1***	52	7
**19**	NiCl$_2$•6H$_2$O (5)	**L1** (10)	Zn (2.5)	DMA (3)	2	65	5
**20**	-	**L1** (20)	Zn (2.5)	DMA (3)	2	<2	<2
**21**	NiCl$_2$•6H$_2$O (10)	**-**	Zn (2.5)	DMA (3)	2	10	7
**22**	NiCl$_2$•6H$_2$O (10)	**L1** (20)	-	DMA (3)	2	<2	<2

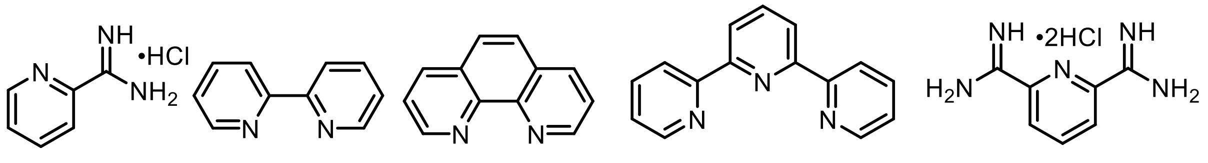

L1 **L2** **L5** **L7** **L9**

[a]Yield determined *via* analysis of the crude ^{1}H NMR spectra, using mesitylene as an internal standard. Isolated yields in parentheses.

3.1.3. Determination of Optimal Sodium Iodide Equivalents for Use of Alkyl Bromide Coupling Partners

Table S4. Determination of Optimal Sodium Iodide equivalents.

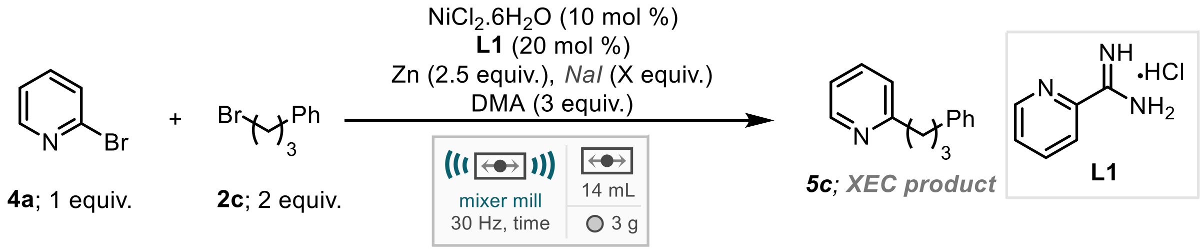

Entry	Sodium iodide (equiv.)	4a consumed (%)[a]	5c (%)[a]
**1**	-	>98	18
**2**	0.25	>98	39
**3**	0.50	77	36
**4**	1.0	79	43
**5**	2.0	84	59 (56)
**6**	3.0	>98	55

[a]Yield determined *via* analysis of the crude ^1H NMR spectra, using mesitylene as an internal standard. Isolated yields in parentheses.

3.1.4. General Procedure E – Mechanochemical XEC between Heteroaryl Bromides and Alkyl Halides

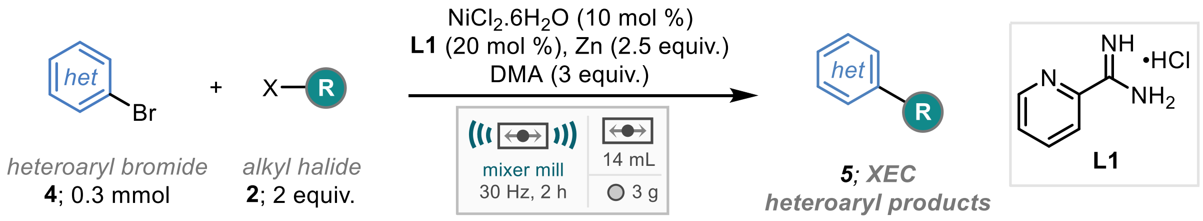

To a 14 mL stainless steel jar, equipped with a 3 g, 9 mm stainless steel ball, was charged nickel chloride hexahydrate (7.1 mg, 0.03 mmol, 10 mol %), amidine ligand **L1** (9.5 mg, 0.06 mmol, 20 mol %), and zinc granular 20 – 30 mesh (49 mg, 0.75 mmol, 2.5 equiv.). Followed by heteroaryl bromide (0.3 mmol, 1 equiv.), alkyl halide (0.6 mmol, 2 equiv.) and *N*,*N*-dimethylacetamide (84 µL, 0.9 mmol, 3 equiv.). The jar was closed and placed on the mixer mill, to be milled at 30 Hz for 2 hours. Upon completion. the reaction mixture was transferred to a separating funnel using ethyl acetate (25 mL) and water (25 mL), and the organic phase was washed twice with a 5% solution of ammonium hydroxide (2 x 20 mL). The aqueous phase was extracted once more with ethyl acetate (25 mL) and the combined organic phases were washed with brine (~20 mL), then dried over magnesium sulfate and filtered. After concentrating *in vacuo*, the crude material was purified by FCC in the stated solvent system.

N.B. For alkyl bromide coupling partners, sodium iodide (2 equiv.) was used as an additive.

2-octylpyridine (5aa)

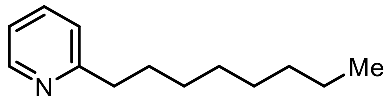

Prepared according to general procedure E. Purified by FCC (5% EtOAc/hexane) to give the title compound as a pale-yellow oil (41 mg, 72%).

^{1}H NMR (500 MHz, $CDCl_3$) δ 8.53 – 8.50 (m, 1H), 7.57 (td, *J* = 7.6, 1.8 Hz, 1H), 7.13 (d, *J* = 7.8 Hz, 1H), 7.11 – 7.05 (m, 1H), 2.80 – 2.73 (m, 2H), 1.74 – 1.67 (m, 2H), 1.39 – 1.22 (m, 10H), 0.86 (t, *J* = 7.0 Hz, 3H).

^{13}C {^{1}H} APT NMR (126 MHz, $CDCl_3$) δ 162.9, 149.32, 136.4, 122.8, 121.0, 38.6, 32.0, 30.1, 29.6, 29.6, 29.4, 22.8, 14.3.

These data are consistent with the literature.[27]

5-methyl-2-octylpyridine (5ab)

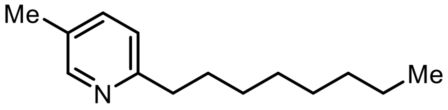

Prepared according to general procedure E. Purified by FCC (5% EtOAc/hexane) to give the title compound (40 mg, 65%) as a colourless oil.

^{1}H NMR (500 MHz, $CDCl_3$) δ 8.34 (s, 1H), 7.38 (dd, *J* = 7.9, 1.8 Hz, 1H), 7.02 (d, *J* = 7.9 Hz, 1H), 2.76 – 2.68 (m, 2H), 2.28 (s, 3H), 1.72 – 1.65 (m, 2H), 1.38 – 1.18 (m, 10H), 0.86 (t, *J* = 7.0 Hz, 3H).

^{13}C {^{1}H} APT NMR (126 MHz, $CDCl_3$) δ 159.7, 149.6, 137.0, 130.1, 122.2, 38.1, 32.0, 30.2, 29.6, 29.6, 29.4, 22.8, 18.2, 14.3.

HRMS (CI) m/z: $[M+H]^+$ Calcd for $C_{14}H_{24}N$ 206.1903, found: 206.1904.

FTIR (film) ν_{max} (cm^{-1}): 2922, 2853, 1603, 1568, 1487, 1381, 1030, 824, 723, 646, 559, 407.

5-fluoro-2-octylpyridine (5ac)

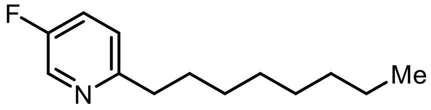

Prepared according to general procedure E. Purified by FCC (3% EtOAc/hexane) to give the title compound oil (43 mg, 68%) as a colourless oil.

^{1}H NMR (400 MHz, $CDCl_3$) δ 8.37 (d, *J* = 2.9 Hz, 1H), 7.29 (td, *J* = 8.5, 3.0 Hz, 1H), 7.12 (dd, *J* = 8.6, 4.4 Hz, 1H), 2.81 – 2.71 (m, 2H), 1.69 (p, *J* = 7.6 Hz, 2H), 1.39 – 1.18 (m, 10H), 0.87 (t, *J* = 6.9 Hz, 3H).

^{13}C {^{1}H} APT NMR (126 MHz, $CDCl_3$) δ 158.6 (d, *J* = 3.8 Hz), 158.1 (d, *J* = 25.3 Hz), 137.2 (d, *J* = 23.9 Hz), 123.4 (d, *J* = 3.8 Hz), 123.1 (d, *J* = 17.6 Hz), 37.7 (d, *J* = 1.3 Hz), 32.0, 30.1, 30.0, 29.4, 29.4, 22.8, 14.2.

^{19}F {^{1}H} NMR (376 MHz, $CDCl_3$) δ -131.94.

HRMS (CI) m/z: $[M+H]^+$ Calcd for $C_{13}H_{21}NF$ 210.1653, found: 210.1651.

FTIR (film) ν_{max} (cm^{-1}): 2924, 2855, 1585, 1483, 1468, 1339, 1225, 1020, 908, 829.

6-octylnicotinonitrile (5ad)

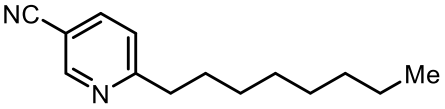

Prepared according to general procedure E. Purified by FCC (3% EtOAc/hexane) to give the title compound (29 mg, 46%) as a colourless, viscous oil.

^{1}H NMR (500 MHz, $CDCl_3$) δ 8.80 (dd, *J* = 2.1, 0.8 Hz, 1H), 7.85 (dd, *J* = 8.1, 2.2 Hz, 1H), 7.28 – 7.26 (m, 1H), 2.89 – 2.80 (m, 2H), 1.78 – 1.66 (m, 2H), 1.39 – 1.19 (m, 10H), 0.87 (t, *J* = 7.0 Hz, 3H).

^{13}C {^{1}H} APT NMR (126 MHz, $CDCl_3$) δ 167.4, 152.3, 139.4, 122.9, 117.2, 107.2, 38.9, 32.0, 29.6, 29.5, 29.4, 29.3, 22.8, 14.2.

HRMS (CI) m/z: $[M+H]^+$ Calcd for $C_{14}H_{21}N_2$ 217.1699, found: 217.1699.

FTIR (film) v_{max} (cm^{-1}): 2924, 2855, 2232, 1593, 1553, 1481, 1452, 1379, 1092, 1024, 833.

2-octyl-5-(trifluoromethyl)pyridine (5ae)

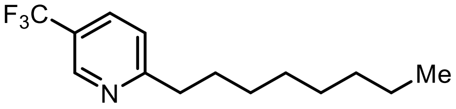

Prepared according to general procedure E. Purified by FCC (5% EtOAc/hexane) to give the title compound (39 mg, 50%) as a colourless oil.

^{1}H NMR (400 MHz, $CDCl_3$) δ 8.78 (s, 1H), 7.81 (dd, *J* = 8.2, 2.2 Hz, 1H), 7.26 (d, *J* = 8.2 Hz, 1H), 2.91 – 2.78 (m, 2H), 1.80 – 1.67 (m, 2H), 1.40 – 1.19 (m, 10H), 0.87 (t, *J* = 6.9 Hz, 3H).

^{13}C {^{1}H} APT NMR (126 MHz, $CDCl_3$) δ 166.8, 146.3 (q. *J* = 3.8 Hz), 133.4 (q, *J* = 3.8 Hz), 123.9 (q, *J* = 272.2 Hz), 124.1 (q, *J* = 32.8 Hz), 122.5, 38.6, 32.0, 29.8, 29.5, 29.5, 29.3, 22.8, 14.2.

^{19}F {^{1}H} NMR (376 MHz, $CDCl_3$) δ -62.22.

HRMS (CI) m/z: $[M+H]^+$ Calcd for $C_{14}H_{21}NF_3$ 260.1621, found: 260.1625.

FTIR (film) v_{max} (cm^{-1}): 2926, 2857, 1609, 1574, 1325, 1161, 1126, 1078, 1016.

5-Chloro-2-octylpyridine (5af)

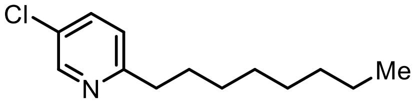

Prepared according to general procedure E. Purified by FCC (5% EtOAc/hexane) to give the title compound (42 mg, 62%) as a colourless oil.

^{1}H NMR (500 MHz, $CDCl_3$) δ 8.47 (dd, *J* = 2.5, 0.5 Hz, 1H), 7.55 (dd, *J* = 8.3, 2.5 Hz, 1H), 7.09 (d, *J* = 8.3 Hz, 1H), 2.79 – 2.71 (m, 2H), 1.72 – 1.65 (m, 2H), 1.38 – 1.19 (m, 10H), 0.87 (t, *J* = 7.0 Hz, 3H).

^{13}C {^{1}H} APT NMR (126 MHz, $CDCl_3$) δ 160.9, 148.1, 136.1, 129.3, 123.6, 37.9, 32.0, 30.0, 29.6, 29.4, 29.4, 22.8, 14.2.

HRMS (ES) m/z: $[M+H]^+$ Calcd for $C_{13}H_{21}NCl$ 226.1356, found: 226.1363.

FTIR (film) v_{max} (cm^{-1}): 2923, 2855, 1580, 1560, 1468, 1376, 1112, 1013.

6-octylpyridin-3-ol (5ag)

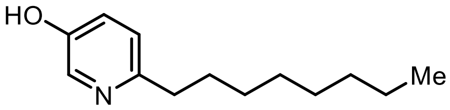

Prepared according to general procedure E. Purified by FCC (25% EtOAc/hexane) to give the title compound (21 mg, 34%) as a pale-yellow oil.

^{1}H NMR (500 MHz, $CDCl_3$) δ 8.18 (d, *J* = 2.1 Hz, 1H), 7.24 (dd, *J* = 8.5, 2.6 Hz, 1H), 7.10 (d, *J* = 8.5 Hz, 1H), 2.79 – 2.67 (m, 2H), 1.69 – 1.57 (m, 2H), 1.36 – 1.13 (m, 10H), 0.86 (t, *J* = 7.0 Hz, 3H).

^{13}C {^{1}H} APT NMR (126 MHz, $CDCl_3$) δ 153.2, 152.8, 135.7, 125.8, 124.3, 36.5, 32.0, 30.5, 29.5, 29.4, 29.4, 22.8, 14.3.

HRMS (ES) m/z: $[M+H]^+$ Calcd for $C_{13}H_{22}NO$ 208.1700, found: 208.1701.

FTIR (film) v_{max} (cm^{-1}): 2980, 2970, 2957, 2924, 2853, 2627 (broad), 1572, 1495, 1460, 1275, 833, 739, 654.

5-methoxy-2-octylpyridine (5ah)

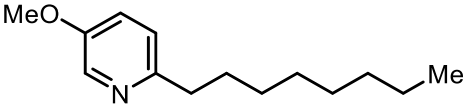

Prepared according to general procedure E. Purified by FCC (10% EtOAc/hexane) to give the title compound oil (36 mg, 56%) as a colourless oil.

^{1}H NMR (500 MHz, $CDCl_3$) δ 8.22 (d, *J* = 2.9 Hz, 1H), 7.12 (dd, *J* = 8.5, 3.0 Hz, 1H), 7.05 (d, *J* = 8.5 Hz, 1H), 3.83 (s, 3H), 2.74 – 2.68 (m, 2H), 1.72 – 1.61 (m, 2H), 1.38 – 1.15 (m, 10H), 0.87 (t, *J* = 7.0 Hz, 3H).

^{13}C {^{1}H} APT NMR (126 MHz, $CDCl_3$) δ 154.8, 153.9, 136.4, 122.8, 121.4, 55.7, 37.6, 32.0, 30.3, 29.6, 29.5, 29.4, 22.8, 14.3.

HRMS (CI) m/z: $[M+H]^+$ Calcd for $C_{14}H_{24}ON$ 222.1852, found: 222.1856.

FTIR (film) v_{max} (cm^{-1}): 2980, 2970, 2957, 2922, 2853, 1572, 1495, 1483, 1464, 1395, 1034, 824.

6-octylpyridin-3-amine (5ai)

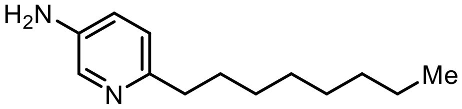

Prepared according to general procedure E. Purified by FCC (50 – 60% EtOAc/hexane) to give the title compound (25 mg, 40%) as an orange oil.

^{1}H NMR (500 MHz, $CDCl_3$) δ 8.04 (t, *J* = 1.7 Hz, 1H), 6.93 (d, *J* = 1.9 Hz, 2H), 3.58 (s, 2H), 2.72 – 2.61 (m, 2H), 1.71 – 1.60 (m, 2H), 1.37 – 1.15 (m, 10H), 0.87 (t, *J* = 7.0 Hz, 3H).

^{13}C {^{1}H} APT NMR (126 MHz, $CDCl_3$) δ 152.7, 140.2, 136.7, 122.9, 122.8, 37.4, 32.0, 30.4, 29.6, 29.5, 29.4, 22.8, 14.3.

HRMS (CI) m/z: $[M+H]^+$ Calcd for $C_{13}H_{23}N_2$ 207.1856, found: 207.1859.

FTIR (film) v_{max} (cm^{-1}): 3337, 3204, 2980, 2970, 2922, 2853, 1632, 1572, 1493, 1260, 1138, 829.

N-(6-octylpyridin-3-yl)acetamide (5aj)

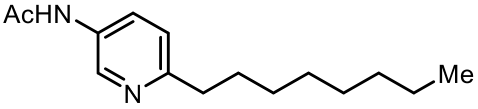

Prepared according to general procedure E. Purified by FCC (70% EtOAc/hexane) to give the title compound (34 mg, 46%) as an off-white solid; m.p. 77 – 79 °C.

^{1}H NMR (500 MHz, $CDCl_3$) δ 8.41 (d, *J* = 2.5 Hz, 1H), 8.06 (dd, *J* = 8.4, 2.6 Hz, 1H), 7.63 (s, 1H), 7.11 (d, *J* = 8.4 Hz, 1H), 2.77 – 2.69 (m, 2H), 2.19 (s, 3H), 1.72 – 1.62 (m, 2H), 1.38 – 1.16 (m, 10H), 0.86 (t, *J* = 7.0 Hz, 3H).

^{13}C {^{1}H} APT NMR (126 MHz, $CDCl_3$) δ 168.9, 158.4, 140.6, 132.5, 128.3, 122.8, 37.8, 32.0, 30.1, 29.6, 29.5, 29.4, 24.5, 22.8, 14.2.

HRMS (CI) m/z: $[M+H]^+$ Calcd for $C_{15}H_{25}ON_2$ 249.1961, found: 249.1964.

FTIR (film) ν_{max} (cm^{-1}): 3254, 2980, 2922, 2855, 1670, 1491, 1383, 1302, 750.

Ethyl 4-(pyridin-3-yl)butanoate (5l)

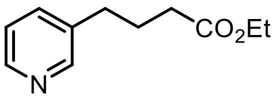

Prepared according to general procedure E, with sodium iodide (2 equiv.) as an additive. Purified by FCC (40% EtOAc/hexane) to give the title compound (29 mg, 49%) as a yellow oil.

^{1}H NMR (500 MHz, $CDCl_3$) δ 8.47 – 8.41 (m, 2H), 7.53 – 7.48 (m, 1H), 7.21 (ddd, *J* = 7.8, 4.8, 0.7 Hz, 1H), 4.12 (q, *J* = 7.1 Hz, 2H), 2.68 – 2.61 (m, 2H), 2.32 (t, *J* = 7.4 Hz, 2H), 1.99 – 1.90 (m, 2H), 1.25 (t, *J* = 7.1 Hz, 3H).

^{13}C {^{1}H} APT NMR (126 MHz, $CDCl_3$) δ 173.3, 150.0, 147.6, 136.9, 136.1, 123.5, 60.6, 33.6, 32.3, 26.3, 14.4.

These data are consistent with the literature.[28]

Ethyl 4-(pyridin-4-yl)butanoate (5m)

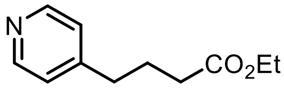

Prepared according to general procedure E, with sodium iodide (2 equiv.) as an additive. Purified by FCC (40% EtOAc/hexane) to give the title compound (15 mg, 25%) as a yellow oil.

^{1}H NMR (500 MHz, $CDCl_3$) δ 8.49 (dd, *J* = 4.4, 1.6 Hz, 2H), 7.11 (dd, *J* = 4.4, 1.6 Hz, 2H), 4.13 (q, *J* = 7.1 Hz, 2H), 2.68 – 2.61 (m, 2H), 2.32 (t, *J* = 7.4 Hz, 2H), 2.00 – 1.92 (m, 2H), 1.25 (t, *J* = 7.1 Hz, 3H).

^{13}C {^{1}H} APT NMR (126 MHz, $CDCl_3$) δ 173.2, 150.5, 149.9, 124.1, 60.6, 34.5, 33.6, 25.5, 14.4.

These data are consistent with the literature.[29]

Ethyl 4-(pyrimidin-5-yl)butanoate (5n)

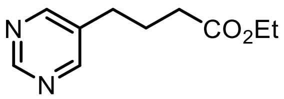

Prepared according to general procedure E, with sodium iodide (2 equiv.) as an additive. Purified by FCC (40% – 50% EtOAc/hexane) to give the title compound (39 mg, 67%) as a colourless oil.

^{1}H NMR (400 MHz, $CDCl_3$) δ 9.07 (s, 1H), 8.58 (s, 2H), 4.13 (q, *J* = 7.1 Hz, 2H), 2.71 – 2.61 (m, 2H), 2.35 (t, *J* = 7.2 Hz, 2H), 2.02 – 1.90 (m, 2H), 1.25 (t, *J* = 7.1 Hz, 3H).

^{13}C {^{1}H} APT NMR (101 MHz, $CDCl_3$) δ 172.9, 157.1, 156.9, 134.5, 60.9, 33.4, 29.8, 25.9, 14.4.

HRMS (CI) m/z: $[M+H]^+$ Calcd for $C_{10}H_{15}O_2N_2$ 195.1128, found: 195.1127.

FTIR (film) v_{max} (cm^{-1}): 2980, 1728, 1560, 1410, 1182, 727, 633.

Ethyl 4-(pyrimidin-2-yl)butanoate (5o)

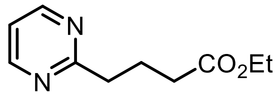

Prepared according to general procedure E, sodium iodide (2 equiv.) as an additive. Purified by FCC (50% EtOAc/hexane) to give the title compound (24 mg, 42%) as a yellow oil.

^{1}H NMR (500 MHz, $CDCl_3$) δ 8.66 (d, *J* = 4.9 Hz, 2H), 7.13 (t, *J* = 4.9 Hz, 1H), 4.12 (q, *J* = 7.1 Hz, 2H), 3.05 – 2.97 (m, 2H), 2.40 (t, *J* = 7.6 Hz, 2H), 2.21 – 2.13 (m, 2H), 1.24 (t, *J* = 7.1 Hz, 3H).

^{13}C {^{1}H} APT NMR (126 MHz, $CDCl_3$) δ 173.4, 170.7, 157.2, 118.7, 60.5, 38.7, 33.9, 23.7, 14.4.

HRMS (CI) m/z: $[M+H]^+$ Calcd for $C_{10}H_{15}O_2N_2$ 195.1128, found: 195.1129.

FTIR (film) v_{max} (cm^{-1}): 2980, 1728, 1560, 1423, 1239, 1163, 635.

Ethyl 4-(pyrazin-2-yl)butanoate (5p)

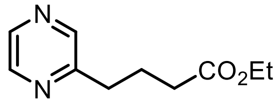

Prepared according to general procedure E, sodium iodide (2 equiv.) as an additive. Purified by FCC (40 – 50% EtOAc/hexane) to give the title compound (13 mg, 23%) as a yellow oil.

^{1}H NMR (400 MHz, $CDCl_3$) δ 8.49 (dd, *J* = 2.5, 1.6 Hz, 1H), 8.46 (d, *J* = 1.3 Hz, 1H), 8.41 (d, *J* = 2.5 Hz, 1H), 4.13 (q, *J* = 7.1 Hz, 2H), 2.90 – 2.83 (m, 2H), 2.38 (t, *J* = 7.4 Hz, 2H), 2.15 – 2.05 (m, 2H), 1.25 (t, *J* = 7.1 Hz, 3H).

^{13}C {^{1}H} APT NMR (101 MHz, $CDCl_3$) δ 173.2, 156.9, 144.8, 144.3, 142.5, 60.6, 34.6, 33.9, 24.5, 14.4.

HRMS (CI) m/z: $[M+H]^+$ Calcd for $C_{10}H_{15}O_2N_2$ 195.1128, found: 195.1128.

FTIR (film) v_{max} (cm^{-1}): 2980, 1728, 1402, 1375, 1161, 1016, 759.

Ethyl 4-(quinolin-3-yl)butanoate (5q)

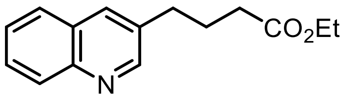

Prepared according to general procedure E, sodium iodide (2 equiv.) as an additive. Purified by FCC (30% EtOAc/hexane) to give the title compound (42 mg, 57%) as a yellow oil.

^{1}H NMR (500 MHz, $CDCl_3$) δ 8.78 (d, *J* = 2.2 Hz, 1H), 8.10 – 8.04 (m, 1H), 7.93 (d, *J* = 1.3 Hz, 1H), 7.79 – 7.74 (m, 1H), 7.66 (ddd, *J* = 8.4, 6.9, 1.5 Hz, 1H), 7.52 (ddd, *J* = 8.1, 6.9, 1.2 Hz, 1H), 4.13 (q, *J* = 7.1 Hz, 2H), 2.90 – 2.79 (m, 2H), 2.38 (t, *J* = 7.4 Hz, 2H), 2.11 – 2.00 (m, 2H), 1.25 (t, *J* = 7.1 Hz, 3H).

^{13}C {^{1}H} APT NMR (126 MHz, $CDCl_3$) δ 173.3, 152.0, 147.0, 134.6, 134.2, 129.3, 128.9, 128.2, 127.5, 126.8, 60.6, 33.6, 32.5, 26.3, 14.4.

These data are consistent with the literature.[30]

Ethyl 4-(1*H*-indol-7-yl)butanoate (5r)

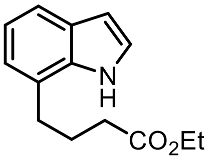

Prepared according to general procedure E, with sodium iodide (2 equiv.) as an additive. Purified by FCC (10% EtOAc/hexane) to give the title compound (20 mg, 28%) as a colourless oil.

^{1}H NMR (500 MHz, $CDCl_3$) δ 9.39 (s, 1H), 7.52 (d, *J* = 7.8 Hz, 1H), 7.29 – 7.26 (m, 1H), 7.04 (dd, *J* = 7.8, 7.1 Hz, 1H), 6.99 – 6.95 (m, 1H), 6.55 (dd, *J* = 3.1, 2.1 Hz, 1H), 4.23 (q, *J* = 7.1 Hz, 2H), 2.91 – 2.82 (m, 2H), 2.49 – 2.42 (m, 2H), 2.07 – 1.98 (m, 2H), 1.32 (t, *J* = 7.1 Hz, 3H).

^{13}C {^{1}H} APT NMR (126 MHz, $CDCl_3$) δ 175.1, 135.4, 127.9, 124.7, 124.4, 121.5, 119.8, 119.0, 102.6, 60.9, 33.2, 31.3, 25.0, 14.4.

HRMS (CI) m/z: $[M+H]^+$ Calcd for $C_{14}H_{17}O_2N$ 231.1254, found: 231.1255.

FTIR (film) v_{max} (cm^{-1}): 3393, 2980, 1711, 1213, 908, 723.

Ethyl 4-(pyridin-2-yl)butanoate (5b)

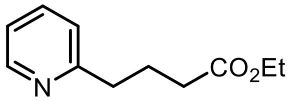

Prepared according to general procedure E, with sodium iodide (2 equiv.) as an additive. Purified by FCC (40% EtOAc/hexane) to give the title compound (42 mg, 72%) as a yellow oil.

^{1}H NMR (500 MHz, $CDCl_3$) δ 8.51 (ddd, *J* = 4.9, 1.8, 0.9 Hz, 1H), 7.58 (td, *J* = 7.7, 1.9 Hz, 1H), 7.14 (d, *J* = 7.8 Hz, 1H), 7.10 (ddd, *J* = 7.5, 4.9, 1.1 Hz, 1H), 4.11 (q, *J* = 7.1 Hz, 2H), 2.85 – 2.78 (m, 2H), 2.35 (t, *J* = 7.5 Hz, 2H), 2.10 – 2.02 (m, 2H), 1.24 (t, *J* = 7.1 Hz, 3H).

^{13}C {^{1}H} APT NMR (126 MHz, $CDCl_3$) δ 173.5, 161.3, 149.4, 136.5, 123.0, 121.3, 60.4, 37.5, 33.8, 25.0, 14.4.

These data are consistent with the literature.[29]

2-(3-phenylpropyl)pyridine (5c)

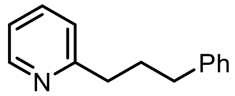

Prepared according to general procedure E, with sodium iodide (2 equiv.) as an additive. Purified by FCC (20% EtOAc/hexane) to give the title compound (33 mg, 56%) as a yellow oil.

^{1}H NMR (500 MHz, $CDCl_3$) δ 8.53 (ddd, *J* = 4.9, 1.8, 0.9 Hz, 1H), 7.58 (td, *J* = 7.7, 1.9 Hz, 1H), 7.31 – 7.26 (m, 2H), 7.22 – 7.16 (m, 3H), 7.15 – 7.12 (m, 1H), 7.10 (ddd, *J* = 7.5, 4.9, 1.1 Hz, 1H), 2.86 – 2.79 (m, 2H), 2.72 – 2.64 (m, 2H), 2.12 – 2.03 (m, 2H).

^{13}C {^{1}H} APT NMR (126 MHz, $CDCl_3$) δ 162.1, 149.4, 142.3, 136.4, 128.6, 128.4, 125.9, 122.9, 121.1, 38.0, 35.7, 31.6.

These data are consistent with the literature.[26]

2-(4-fluorophenethyl)pyridine (5d)

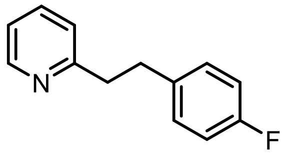

Prepared according to general procedure E, with sodium iodide (2 equiv.) as an additive. Purified by FCC (20% EtOAc/hexane) to give the title compound (33 mg, 54%) as a pale-yellow oil.

^{1}H NMR (500 MHz, $CDCl_3$) δ 8.56 (ddd, *J* = 4.9, 1.8, 0.9 Hz, 1H), 7.56 (td, *J* = 7.6, 1.9 Hz, 1H), 7.15 – 7.08 (m, 3H), 7.05 (dt, *J* = 7.8, 1.0 Hz, 1H), 6.97 – 6.91 (m, 2H), 3.09 – 2.99 (m, 4H).

^{13}C {^{1}H} APT NMR (126 MHz, $CDCl_3$) δ 162.7 (d, *J* = 171.4 Hz), 160.5, 149.5, 137.3 (d, *J* = 3.8 Hz), 136.4, 129.9 (d, *J* = 7.6 Hz), 123.2, 121.4, 115.2 (d, *J* = 21.4 Hz), 40.4, 35.3.

^{19}F {^{1}H} NMR (376 MHz, $CDCl_3$) δ -117.58

HRMS (EI) m/z: $[M-H]^+$ Calcd for $C_{13}H_{11}NF$ 200.0870, found: 200.0870.

FTIR (film) ν_{max} (cm^{-1}): 1590, 1569, 1508, 1475, 1435, 1276, 1218, 1157, 1051.

***N*-(6-(but-3-en-1-yl)pyridin-3-yl)acetamide (5e)**

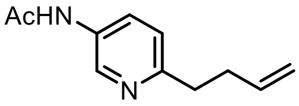

Prepared according to general procedure E, with sodium iodide (2 equiv.) as an additive. Purified by FCC (80% EtOAc/hexane) to give the title compound (13 mg, 22%) as an off-white solid; m.p. 102 – 104 °C.

^{1}H NMR (500 MHz, $CDCl_3$) δ 8.42 (d, *J* = 2.5 Hz, 1H), 8.08 (dd, *J* = 8.4, 2.6 Hz, 1H), 7.36 (s, 1H), 7.13 (d, *J* = 8.4 Hz, 1H), 5.85 (ddt, *J* = 16.9, 10.2, 6.6 Hz, 1H), 5.03 (ddd, *J* = 17.1, 3.4, 1.6 Hz, 1H), 5.00 – 4.94 (m, 1H), 2.88 – 2.82 (m, 2H), 2.50 – 2.42 (m, 2H), 2.20 (s, 3H).

^{13}C {^{1}H} APT NMR (126 MHz, $CDCl_3$) δ 168.8, 157.3, 140.6, 137.8, 132.6, 128.2, 122.9, 115.4, 37.1, 33.9, 24.6.

HRMS (ES) m/z: $[M+H]^+$ Calcd for $C_{11}H_{15}N_2O$ 191.1184, found: 191.1182.

FTIR (film) ν_{max} (cm^{-1}): 2939, 1689, 1641, 1611, 1587, 1539, 1486, 1373, 1286, 1242, 1100.

3-(oxetan-3-yl)pyridine (5f)

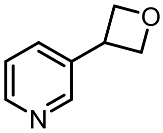

Prepared according to general procedure E, with sodium iodide (2 equiv.) as an additive. Purified by FCC (20% EtOAc/hexane) to give the title compound (13 mg, 32%) as a pale-yellow oil.

^{1}H NMR (500 MHz, $CDCl_3$) δ 8.63 – 8.50 (m, 2H), 7.88 – 7.82 (m, 1H), 7.34 (dd, *J* = 7.8, 4.7 Hz, 1H), 5.12 (dd, *J* = 8.3, 6.2 Hz, 2H), 4.75 (t, *J* = 6.4 Hz, 2H), 4.24 (tt, *J* = 8.3, 6.6 Hz, 1H).

^{13}C {^{1}H} APT NMR (126 MHz, $CDCl_3$) δ 148.8, 148.7, 137.1, 134.2, 142.0, 78.5, 38.0.

HRMS (ES) m/z: $[M+H]^+$ Calcd for $C_8H_{10}NO$ 136.0762, found: 136.0759.

FTIR (film) ν_{max} (cm^{-1}): 2349, 1275, 1267, 1262, 765, 750.

2-(pyridin-2-yl)ethyl benzoate (5g)

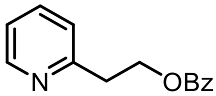

Prepared according to general procedure E, with sodium iodide (2 equiv.) as an additive. Purified by FCC (20 – 40% EtOAc/hexane) to give the title compound (18 mg, 27%) as a colourless oil.

^{1}H NMR (400 MHz, $CDCl_3$) δ 8.61 – 8.54 (m, 1H), 8.02 – 7.94 (m, 2H), 7.62 (td, *J* = 7.7, 1.8 Hz, 1H), 7.57 – 7.50 (m, 1H), 7.45 – 7.36 (m, 2H), 7.25 (d, *J* = 8.7 Hz, 1H), 7.16 (ddd, *J* = 7.4, 4.9, 0.9 Hz, 1H), 4.71 (t, *J* = 6.7 Hz, 2H), 3.26 (t, *J* = 6.7 Hz, 2H).

^{13}C {^{1}H} APT NMR (101 MHz, $CDCl_3$) δ 166.6, 158.3, 149.7, 136.6, 133.0, 130.4, 129.7, 128.5, 123.6, 121.8, 64.3, 37.7.

HRMS (ES) m/z: $[M+H]^+$ Calcd for $C_{14}H_{14}NO_2$ 228.1027, found: 228.1025.

FTIR (film) ν_{max} (cm^{-1}): 1714, 1591, 1570, 1475, 1452, 1437, 1315, 1270, 1177, 1150, 1112, 1071, 1050.

***Tert*-butyl (2-(pyridin-2-yl)ethyl)carbamate (5h)**

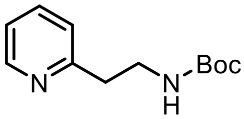

Prepared according to general procedure E, with sodium iodide (2 equiv.) as an additive. Purified by FCC (60% EtOAc/hexane) to give the title compound (16 mg, 24%) as a pale-yellow oil.

^{1}H NMR (500 MHz, $CDCl_3$) δ 8.53 (ddd, *J* = 4.9, 1.8, 0.9 Hz, 1H), 7.61 (td, *J* = 7.7, 1.8 Hz, 1H), 7.19 – 7.12 (m, 2H), 5.13 (s, 1H), 3.54 (dd, *J* = 12.4, 6.1 Hz, 2H), 2.98 (t, *J* = 6.4 Hz, 2H), 1.42 (s, 9H).

^{13}C {^{1}H} APT NMR (126 MHz, $CDCl_3$) δ 159.6, 156.1, 149.3, 136.8, 123.7, 121.7, 79.2, 40.1, 37.9, 28.6.

HRMS (ES) m/z: $[M+H]^+$ Calcd for $C_{12}H_{19}N_2O_2$ 223.1447, found: 223.1450.

FTIR (film) ν_{max} (cm^{-1}): 3337, 2977, 1693, 1594, 1569, 1476, 1436, 1392, 1365, 1275, 1267, 1166, 1051.

2-(3-(pyridin-2-yl)propyl)isoindoline-1,3-dione (5i)

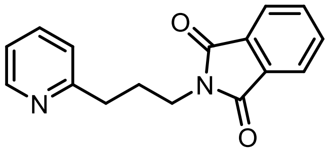

Prepared according to general procedure E. Purified by FCC (40% EtOAc/hexane) to give the title compound (51 mg, 63%) as a viscous, yellow oil.

^{1}H NMR (500 MHz, $CDCl_3$) δ 8.47 (ddd, *J* = 4.9, 1.6, 0.8 Hz, 1H), 7.86 – 7.77 (m, 2H), 7.74 – 7.66 (m, 2H), 7.56 (td, *J* = 7.7, 1.9 Hz, 1H), 7.17 (d, *J* = 7.8 Hz, 1H), 7.06 (ddd, *J* = 7.5, 4.9, 1.0 Hz, 1H), 3.78 (t, *J* = 7.0 Hz, 2H), 2.89 – 2.80 (m, 2H), 2.20 – 2.10 (m, 2H).

^{13}C {^{1}H} APT NMR (126 MHz, $CDCl_3$) δ 168.5, 160.9, 149.4, 136.5, 134.0, 132.3, 123.3, 123.0, 121.3, 37.8, 35.7, 28.5.

HRMS (CI) m/z: $[M+H]^+$ Calcd for $C_{16}H_{15}O_2N_2$ 267.1128, found: 267.1132.

FTIR (film) ν_{max} (cm^{-1}): 2980, 1707, 1395, 1265, 733, 718, 702.

2-cyclohexylpyridine (5j)

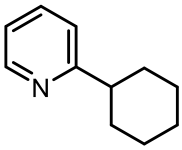

Prepared according to general procedure E. Purified by FCC (10 – 20% Et_2O/pentane) to give the title compound (14 mg, 30%) as a colourless oil.

^{1}H NMR (500 MHz, $CDCl_3$) δ 8.52 (ddd, *J* = 4.9, 1.8, 0.9 Hz, 1H), 7.59 (td, *J* = 7.7, 1.9 Hz, 1H), 7.16 – 7.11 (m, 1H), 7.08 (ddd, *J* = 7.5, 4.8, 1.0 Hz, 1H), 2.69 (tt, *J* = 11.9, 3.4 Hz, 1H), 1.98 – 1.90 (m, 2H), 1.89 – 1.81 (m, 2H), 1.79 – 1.70 (m, 1H), 1.58 – 1.47 (m, 2H), 1.46 – 1.35 (m, 2H), 1.33 – 1.22 (m, 1H).

^{13}C {^{1}H} APT NMR (126 MHz, $CDCl_3$) δ 166.7, 149.2, 136.5, 121.1 (2C), 46.7, 33.1, 26.7, 26.2.

These data are consistent with the literature.[27]

***N*-(6-neopentylpyridin-3-yl)acetamide (5k)**

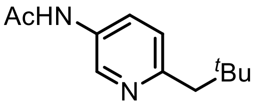

Prepared according to general procedure E. Purified by FCC (70 – 80% EtOAc/hexane) to give the title compound (26 mg, 42%) as a white solid; m.p. 86 – 88 °C.

^{1}H NMR (500 MHz, $CDCl_3$) δ 8.42 (d, *J* = 2.4 Hz, 1H), 8.09 (dd, *J* = 8.4, 2.6 Hz, 1H), 7.43 (s, 1H), 7.09 (d, *J* = 8.4 Hz, 1H), 2.65 (s, 2H), 2.20 (s, 3H), 0.93 (s, 9H).

^{13}C {^{1}H} APT NMR (126 MHz, $CDCl_3$) δ 168.9, 156.1, 140.0, 132.6, 127.5, 124.8, 51.4, 32.2, 29.6, 24.6.

HRMS (ES) m/z: $[M+H]^+$ Calcd for $C_{12}H_{19}N_2O$ 207.1497, found: 207.1498.

FTIR (film) ν_{max} (cm^{-1}): 3467, 2957, 2924, 1666, 1608, 1545, 1490, 1474, 1366, 1311, 1273, 1234, 1144, 1222, 1032.

3.2. Scale-Up Experiment

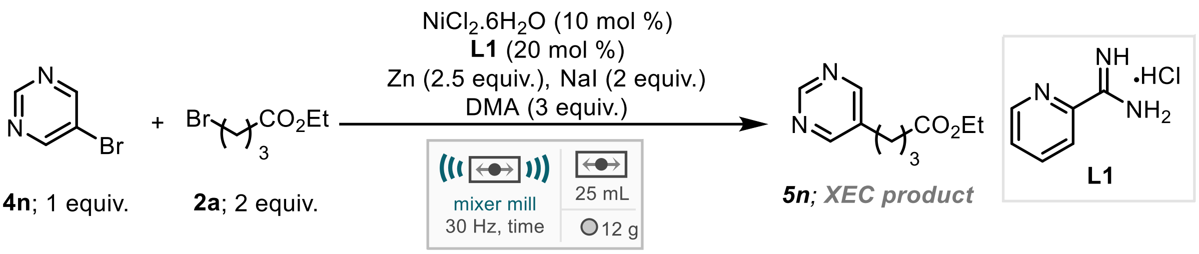

To a 25 mL stainless steel jar, equipped with a 12 g, 14 mm stainless steel ball, was charged nickel chloride hexahydrate (143 mg, 0.6 mmol, 10 mol %), amidine ligand **L1** (189 mg, 1.2 mmol, 20 mol %), zinc granular 20 – 30 mesh (981 mg, 15 mmol, 2.5 equiv.), sodium iodide (1.80 g, 12 mmol, 2 equiv.), and 5-bromopyrimidine (954 mg, 6 mmol, 1 equiv.). Followed by ethyl 4-bromobutyrate (1.72 mL, 12 mmol, 2 equiv.) and *N*,*N*-dimethylacetamide (1.67 mL, 18 mmol, 3 equiv.). The jar was closed and placed on the mixer mill, to be milled at 30 Hz for 2 hours. Upon completion, the reaction mixture was transferred to a separating funnel using ethyl acetate (50 mL) and water (50 mL), and the organic phase was washed twice with a 5% solution of ammonium hydroxide (2 x 20 mL). The aqueous phase was extracted once more with ethyl acetate (50 mL) and the combined organic phases were washed with brine (50 mL), then dried over magnesium sulfate and filtered. After concentrating *in vacuo*, the crude material was purified by FCC (5% EtOAc/hexane) to give **ethyl 4-(pyrimidin-5-yl)butanoate** (0.73 g, 62%) as a colourless oil.

3.3. Mechanistic Studies

3.3.1. Radical Clock Experiment

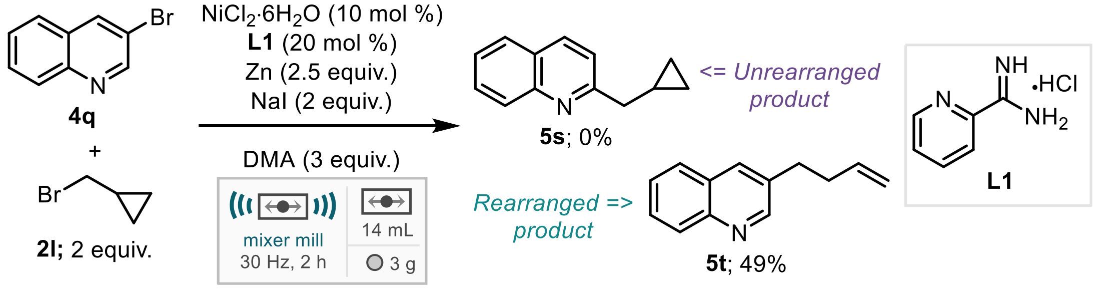

To a 14 mL stainless steel jar, equipped with a 3 g, 9 mm stainless steel ball, was charged nickel chloride hexahydrate (23.8 mg, 0.1 mmol, 10 mol %), amidine ligand **L1** (31.5 mg, 0.2 mmol, 20 mol %), zinc granular 20 – 30 mesh (164 mg, 2.5 mmol, 2.5 equiv.), and sodium iodide (300 mg, 2 mmol, 2 equiv.). Followed by 3-bromoquinoline (135.7 µL, 1 mmol, 1 equiv.), (bromomethyl)cyclopropane (194 µL, 2 mmol, 2 equiv.), and *N*,*N*-dimethylacetamide (278 µL, 3 mmol, 3 equiv.). The jar was closed and placed on the mixer mill, to be milled at 30 Hz for 2 hours. Upon completion, the reaction mixture was transferred to a separating funnel using ethyl acetate (50 mL) and water (50 mL), and the organic phase was washed twice with a 5% solution of ammonium hydroxide (2 x 20 mL). The aqueous phase was extracted once more with ethyl acetate (50 mL) and the combined organic phases were washed with brine (20 mL), then dried over magnesium sulfate and filtered. After concentrating *in vacuo*, the crude material was purified by FCC (10% EtOAc/hexane) to give the rearranged product (**5t**, 90.6 mg, 49%) as a yellow oil.

^{1}H NMR (500 MHz, $CDCl_3$) δ 8.78 (d, J = 2.2 Hz, 1H), 8.10 – 8.05 (m, 1H), 7.91 (d, *J* = 1.4 Hz, 1H), 7.76 (dd, *J* = 8.1, 1.2 Hz, 1H), 7.65 (ddd, *J* = 8.4, 6.9, 1.4 Hz, 1H), 7.51 (ddd, *J* = 8.1, 6.9, 1.2 Hz, 1H), 5.93 – 5.81 (m, 1H), 5.10 – 5.03 (m, 1H), 5.03 – 4.99 (m, 1H), 2.93 – 2.86 (m, 2H), 2.52 – 2.44 (m, 2H).

^{13}C {^{1}H} APT NMR (126 MHz, $CDCl_3$) δ 152.2, 147.0, 137.2, 134.5, 134.4, 129.3, 128.7, 128.2, 127.5, 126.7, 115.9, 35.2, 32.7.

HRMS (ES) m/z: $[M+H]^+$ Calcd for $C_{13}H_{14}N$ 184.1121, found: 184.1120.

FTIR (film) ν_{max} (cm^{-1}): 2926, 1639, 1570, 1495, 1439, 1420, 1325, 1125, 1016, 957, 912, 899, 860.

3.3.2. Radical Trapping Experiments

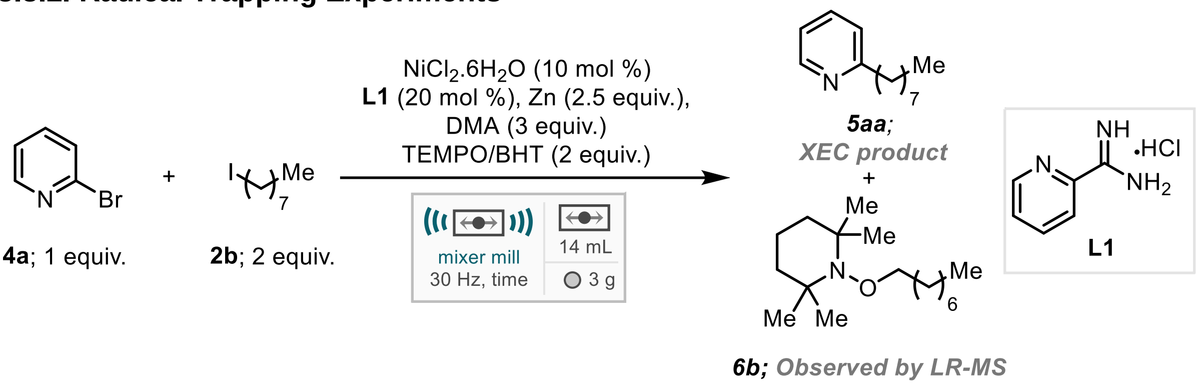

To a 14 mL stainless steel jar, equipped with a 3 g, 9 mm stainless steel ball, was charged nickel chloride hexahydrate (23.8 mg, 0.1 mmol, 10 mol %), amidine ligand **L1** (31.5 mg, 0.2 mmol, 20 mol %), zinc granular 20 – 30 mesh (164 mg, 2.5 mmol, 2.5 equiv.), and either TEMPO (313 mg, 2 mmol, 2 equiv.) or BHT (440.7 mg, 2 mmol, 2 equiv.). Followed by 2-bromopyridine (95.2 µL, 1 mmol, 1 equiv.), 1-iodooctane (361 µL, 2 mmol, 2 equiv.) and *N*,*N*-dimethylacetamide (278 µL, 3 mmol, 3 equiv.). The jar was closed and placed on the mixer mill, to be milled at 30 Hz for 2 hours. Upon completion, the reaction mixture was transferred to a separating funnel using ethyl acetate (50 mL) and water (50 mL), and the organic phase was washed twice with a 5% solution of ammonium hydroxide (2 x 20 mL). The aqueous phase was extracted once more with ethyl acetate (50 mL) and the combined organic phases were washed with brine (20 mL), then dried over magnesium sulfate and filtered. After concentrating *in vacuo*, the crude material was analysed *via* ^1H NMR and LR-MS to determine the presence of product (**5aa**) and/or TEMPO adduct(s).

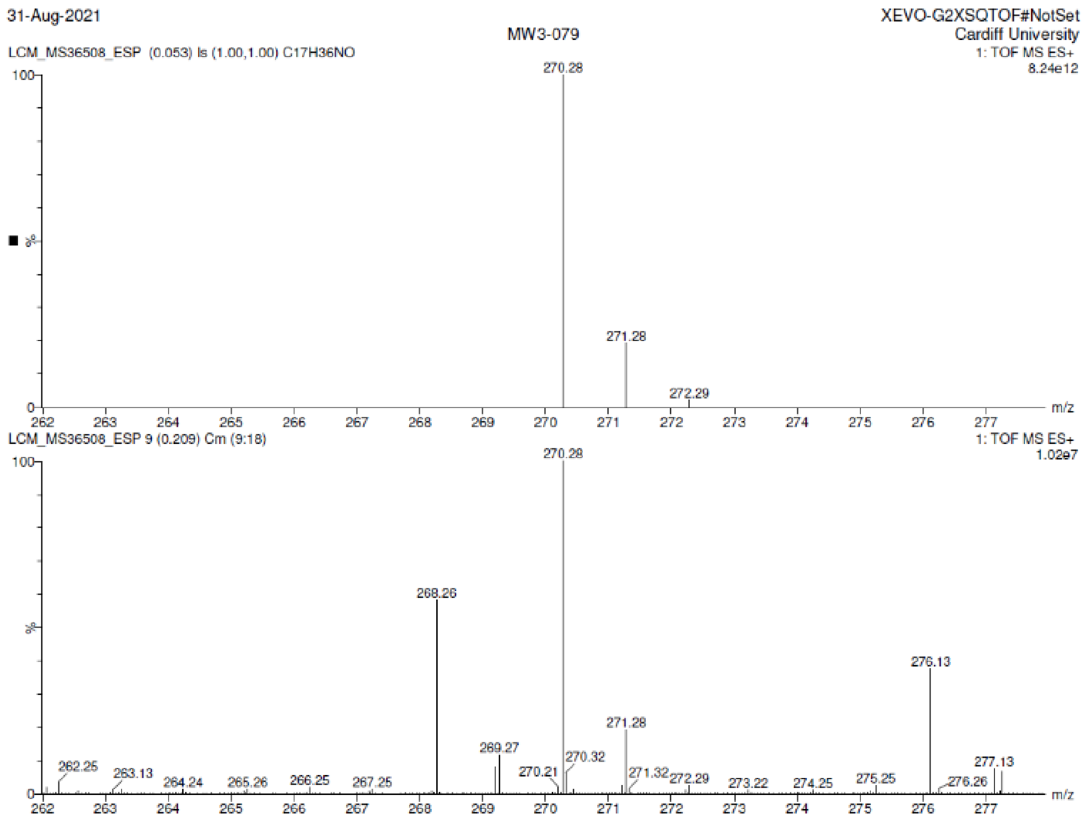

3.4. Solution-Phase Comparisons

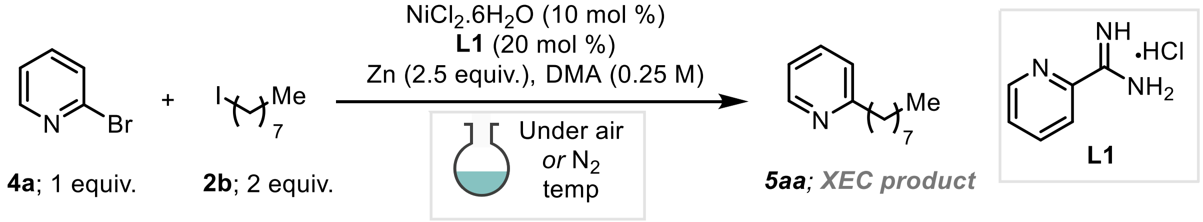

To a 5 mL microwave vial, equipped with a magnetic stirrer, was charged nickel chloride hexahydrate (7.1 mg, 0.03 mmol, 10 mol %), amidine ligand **L1** (9.5 mg, 0.06 mmol, 20 mol %), and zinc granular 20 – 30 mesh (49 mg, 0.75 mmol, 2.5 equiv.). The vial was capped and either left under an air atmosphere or a nitrogen inlet was introduced. Following this, 2-bromopyridine (28.6 µL, 0.3 mmol, 1 equiv.), 1-iodooctane (108.3 µL, 0.6 mmol, 2 equiv.), and *N*,*N*-dimethylacetamide (1.2 mL, 0.25 M) were added. The DMA used was either Winchester grade or dry. The reaction mixture was stirred at either room temperature or 60 °C for 16 hours. Upon completion, the reaction mixture was quenched with a small volume of 5% ammonium hydroxide solution (2 mL). Ethyl acetate (2 mL) and internal standard (mesitylene) were added and the mixture allowed to stir for a few minutes. The phases were allowed to separate, and an aliquot of the organic layer was taken, to be analysed by ^{1}H NMR, to determine the quantity of XEC product present.

3.5. Organomanganese Formation Studies

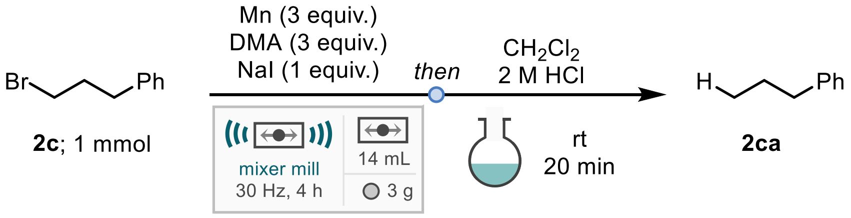

To a 14 mL stainless steel jar, equipped with a 3 g, 9 mm stainless steel ball, was charged 1-bromo-3-phenylpropane (152.0 µL, 1 mmol, 1 equiv.), manganese powder (164.8 mg, 3 mmol, 3 equiv.), sodium iodide (149.9 mg, 1 mmol, 1 equiv.), and *N*,*N*-dimethylacetamide (278.0 µL, 3 mmol, 3 equiv.). The jar was closed and placed on the mixer mill, to be milled at 30 Hz for 4 hours. After completion, the reaction mixture was transferred to a conical flask, equipped with a stirrer bar, using CH_2Cl_2 (~20 mL). 2 M HCl (~20 mL) was then added, and the resulting mixture was stirred at room temperature for 20 minutes to quench any organomanganese compounds. After this period, the mixture was transferred to a separating funnel and the phases separated. The aqeuous phase was extracted once more using CH_2Cl_2 (~20 mL) and the combined organic phases were washed with brine and dried over magnesium sulfate. After concentrating *in vacuo*, the crude compound was analysed by ^{1}H NMR, using mesitylene as an internal standard.

Table S5. Organomanganese formation studies.

Entry	Comments	2c consumed (%)[a]	2ca (%)[a]
**1**	Without NaI	55	31
**2**	With NaI	>98	60

[a]Yield determined *via* analysis of the crude ^{1}H NMR spectra, using mesitylene as an internal standard.

3.6. Heated Ball-Mill Studies

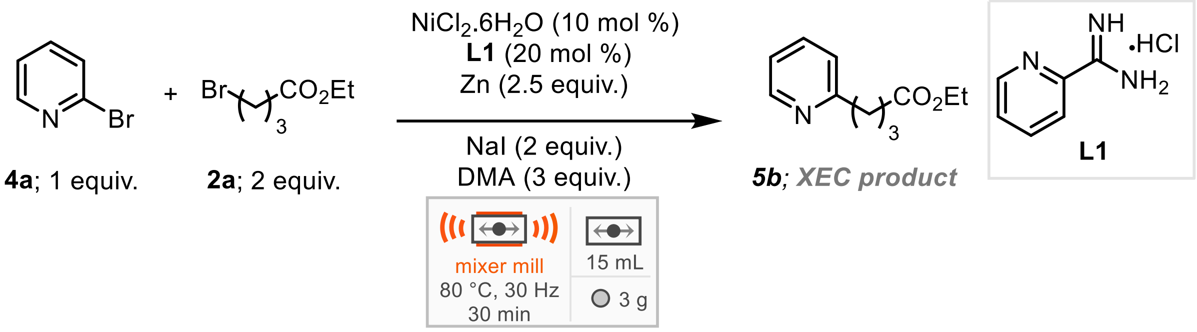

To a 15 mL stainless steel jar, equipped with a 3 g, 9 mm stainless steel ball, was charged nickel chloride hexahydrate (7.1 mg, 0.03 mmol, 10 mol %), amidine ligand **L1** (9.5 mg, 0.06 mmol, 20 mol %), zinc granular 20 – 30 mesh (49 mg, 0.75 mmol, 2.5 equiv.), and sodium iodide (89.9 mg, 0.6 mmol, 2 equiv.). Followed by 2-bromopyridine (28.6 µL, 0.3 mmol, 1 equiv.), ethyl 4-bromobutyrate (85.9 µL, 0.6 mmol, 2 equiv.) and *N*,*N*-dimethylacetamide (83.7 µL, 0.9 mmol, 3 equiv.). The jar was closed, placed on the mixer mill, and encased by a band heater. Heating to 80 °C and milling at 30 Hz were commenced simultaneously and continued for 30 minutes. Upon completion, the jar was allowed to cool, and the reaction mixture was transferred to a separating funnel using ethyl acetate (25 mL) and water (25 mL). The organic phase was washed twice with a 5% solution of ammonium hydroxide (2 x 20 mL). The aqueous phase was extracted once more with ethyl acetate (25 mL) and the combined organic phases were washed with brine (20 mL), then dried over magnesium sulfate and filtered. After concentrating *in vacuo*, the crude material was analysed by ^{1}H NMR using mesitylene as an internal standard.

3.7. Stainless Steel-Free Reactions in Planetary Ball-Mill

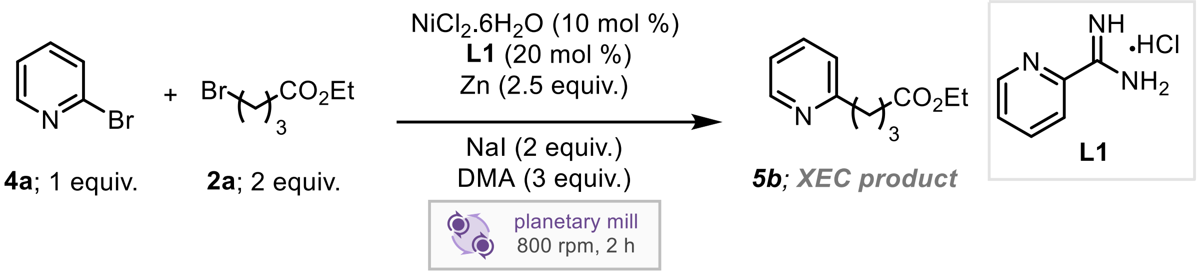

To a 12 mL zirconium oxide grinding bowl, equipped with 6, 3 g, 10 mm ceramic grinding balls, was charged nickel chloride hexahydrate (7.1 mg, 0.03 mmol, 10 mol %), amidine ligand **L1** (9.5 mg, 0.06 mmol, 20 mol %), zinc granular 20 – 30 mesh (49 mg, 0.75 mmol, 2.5 equiv.), and sodium iodide (89.9 mg, 0.6 mmol, 2 equiv.). Followed by 2-bromopyridine (28.6 µL, 0.3 mmol, 1 equiv.), ethyl 4-bromobutyrate (85.9 µL, 0.6 mmol, 2 equiv.) and *N*,*N*-dimethylacetamide (83.7 µL, 0.9 mmol, 3 equiv.). The lid was placed on the bowl and the bowl mounted on the central disc of the planetary mill. The reaction mixture was then ground at 800 rpm, for 2 hours (15 minute cycles, with reverse cycles and 1 minute pauses in between cycles). Upon completion, the reaction mixture was transferred to a separating funnel using ethyl acetate (25 mL) and water (25 mL). The organic phase was washed twice with a 5% solution of ammonium hydroxide (2 x 20 mL). The aqueous phase was extracted once more with ethyl acetate (25 mL) and the combined organic phases were washed with brine (20 mL), then dried over magnesium sulfate and filtered. After concentrating *in vacuo*, the crude material was analysed by ^{1}H NMR using mesitylene as an internal standard.

4. NMR Spectra

Figure S5. 1aa, ^{1}H NMR, 500 MHz, $CDCl_3$

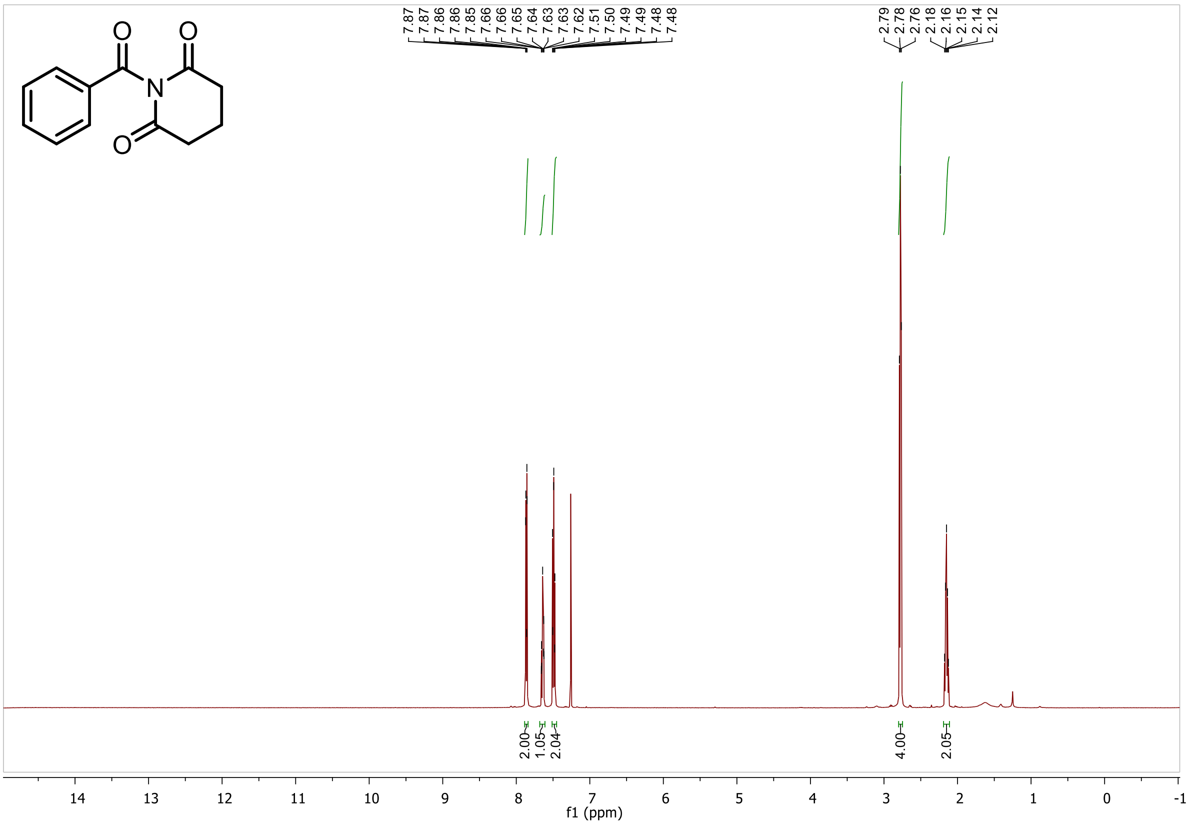

Figure S6. 1aa, ^{13}C {^{1}H} NMR, 126 MHz, $CDCl_3$

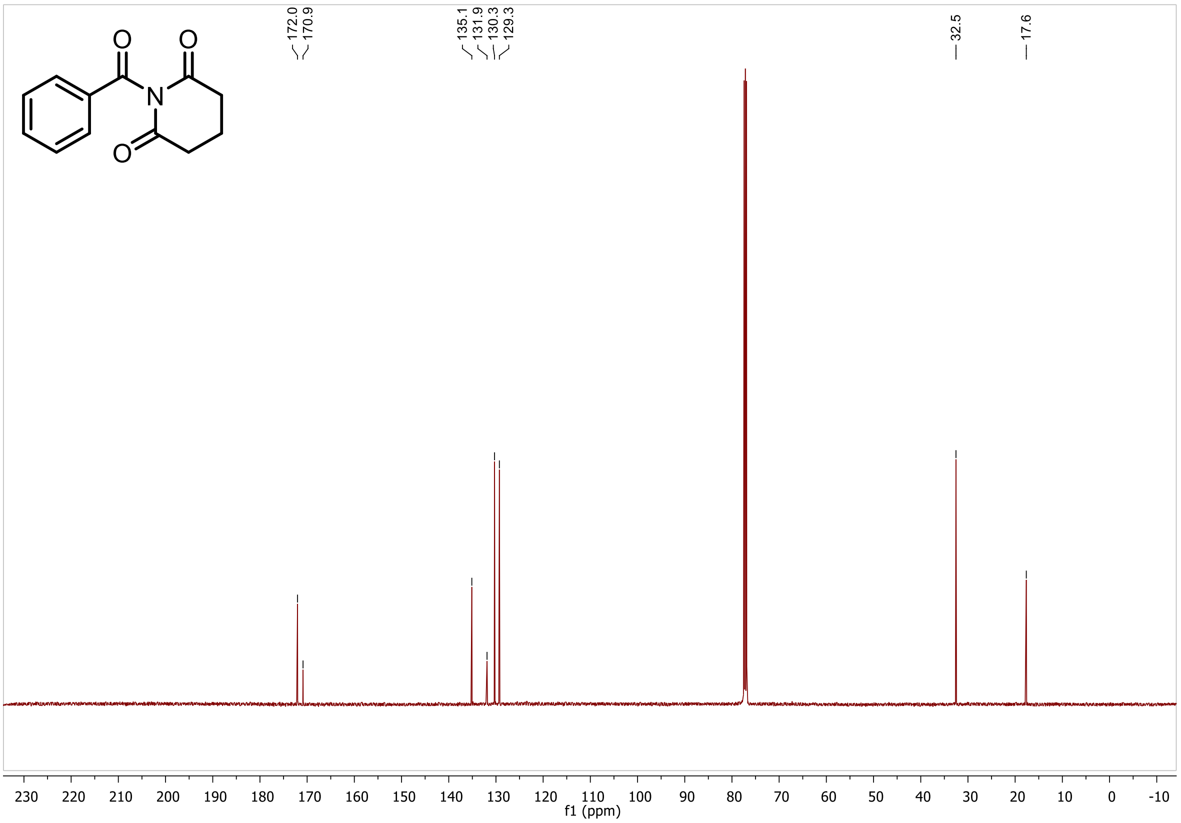

Figure S7. 1b, ^{1}H NMR, 500 MHz, $CDCl_3$

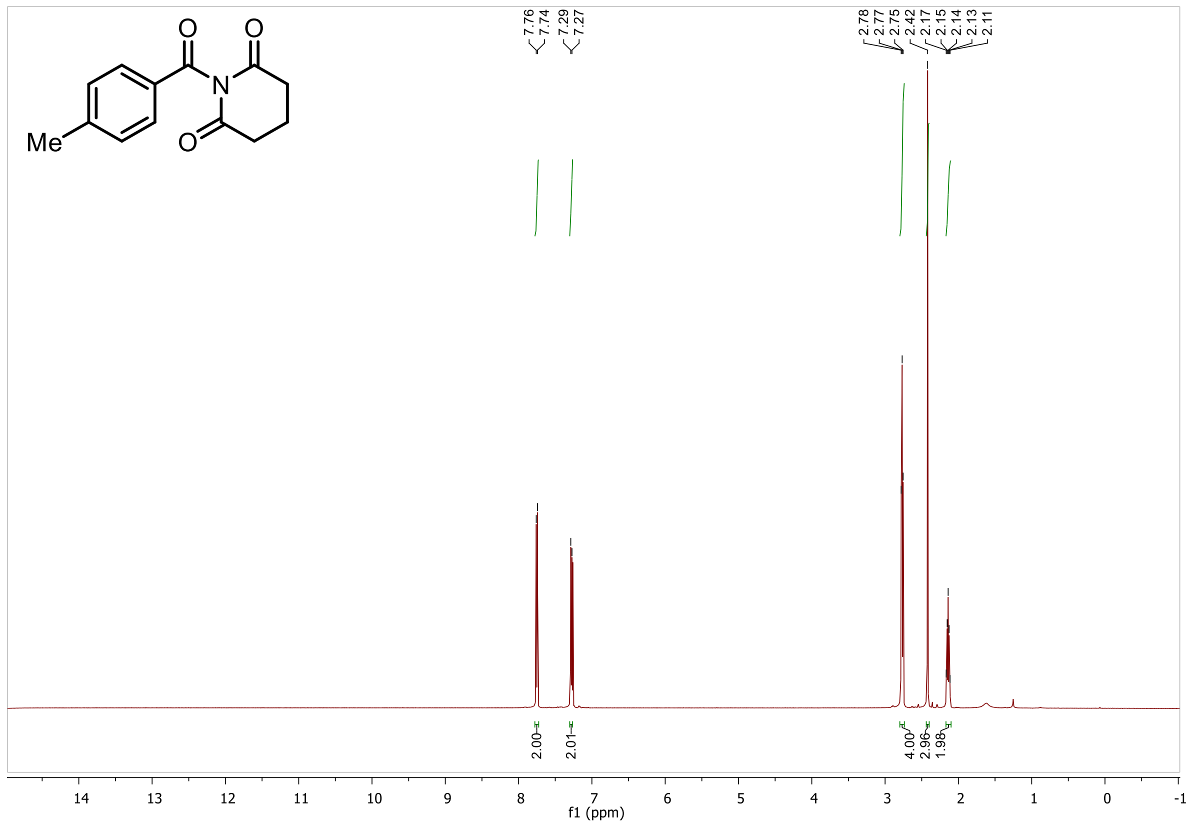

Figure S8. 1b, ^{13}C {^{1}H} NMR, 126 MHz, $CDCl_3$

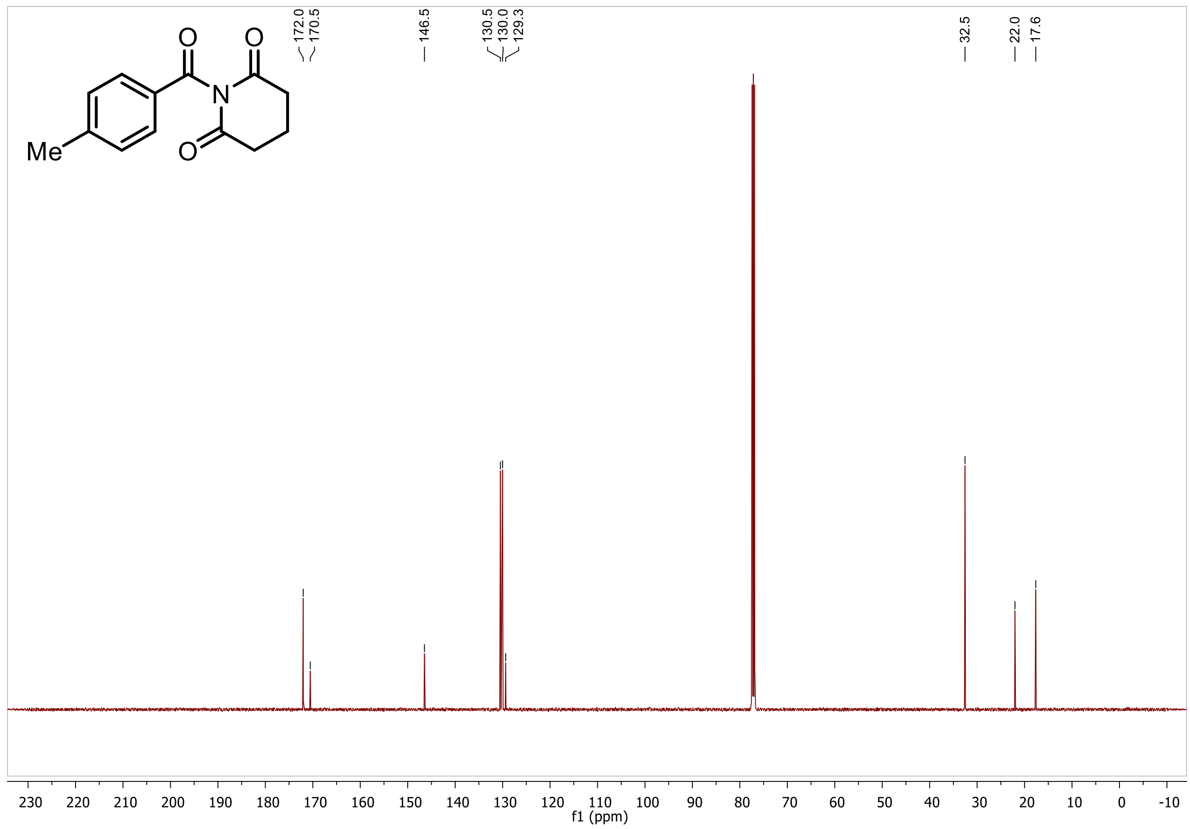

Figure S9. 1c, ^{1}H NMR, 500 MHz, $CDCl_3$

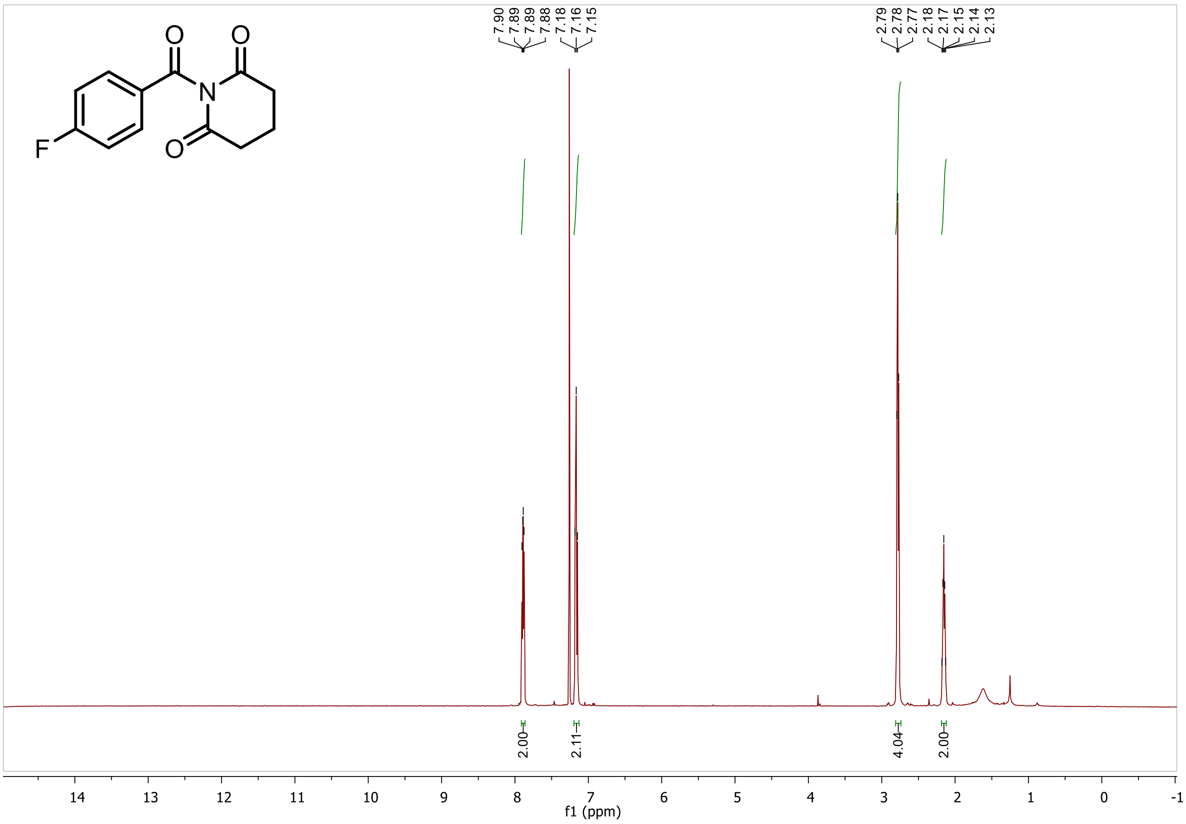

Figure S10. 1c, ^{13}C {^{1}H} NMR, 126 MHz, $CDCl_3$

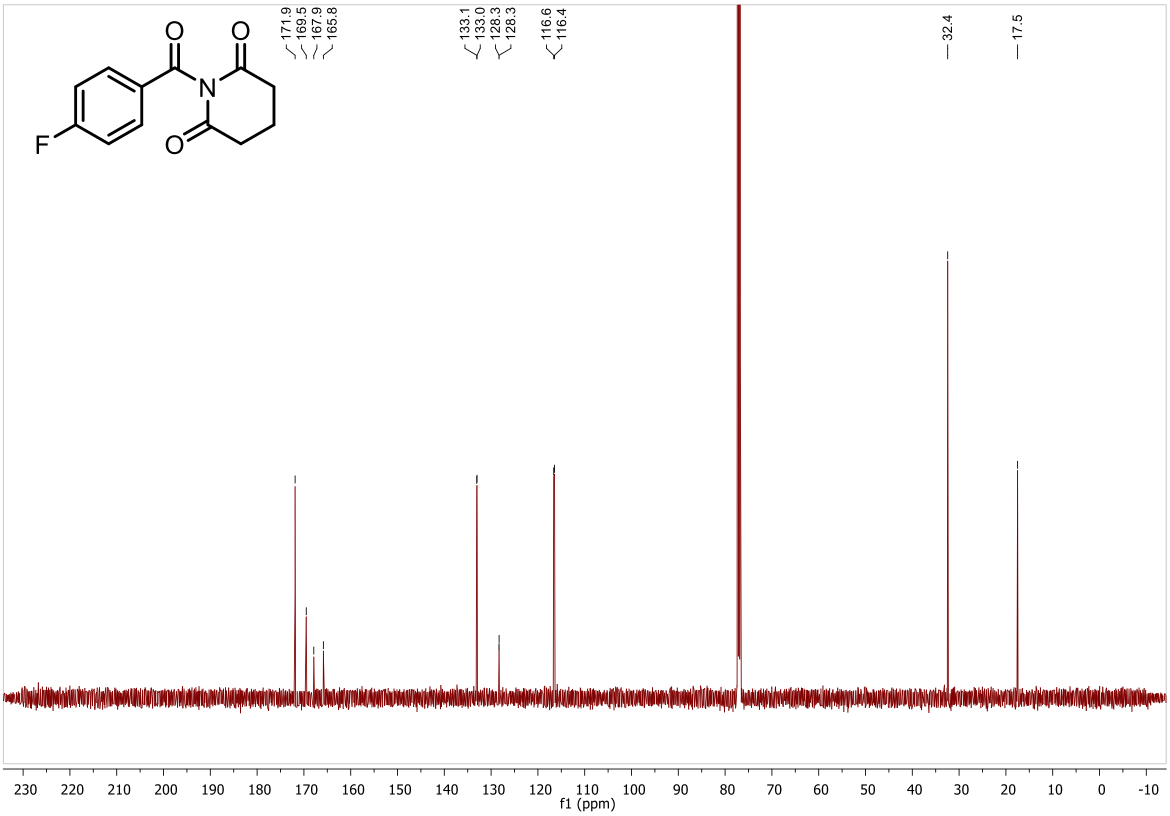

Figure S11. 1c, ^{19}F NMR, 471 MHz, $CDCl_3$

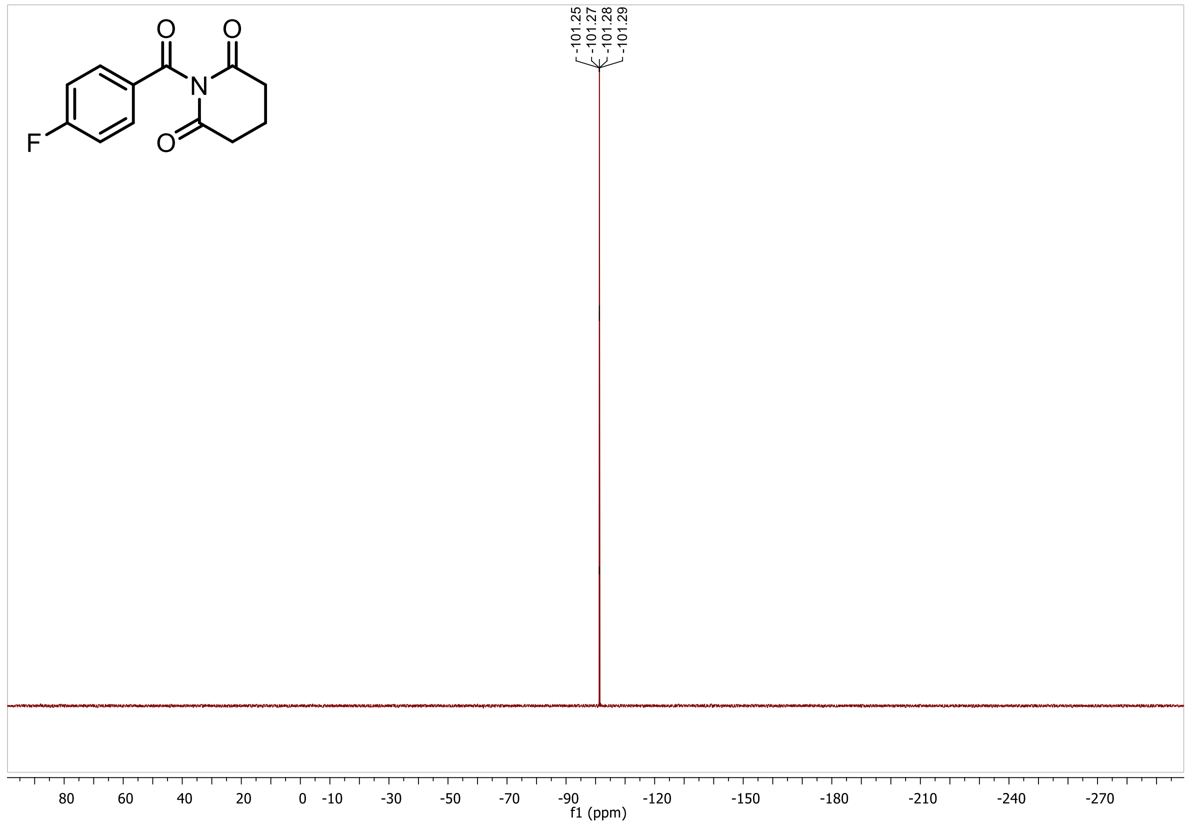

Figure S12. 1d, ^{1}H NMR, 500 MHz, $CDCl_3$

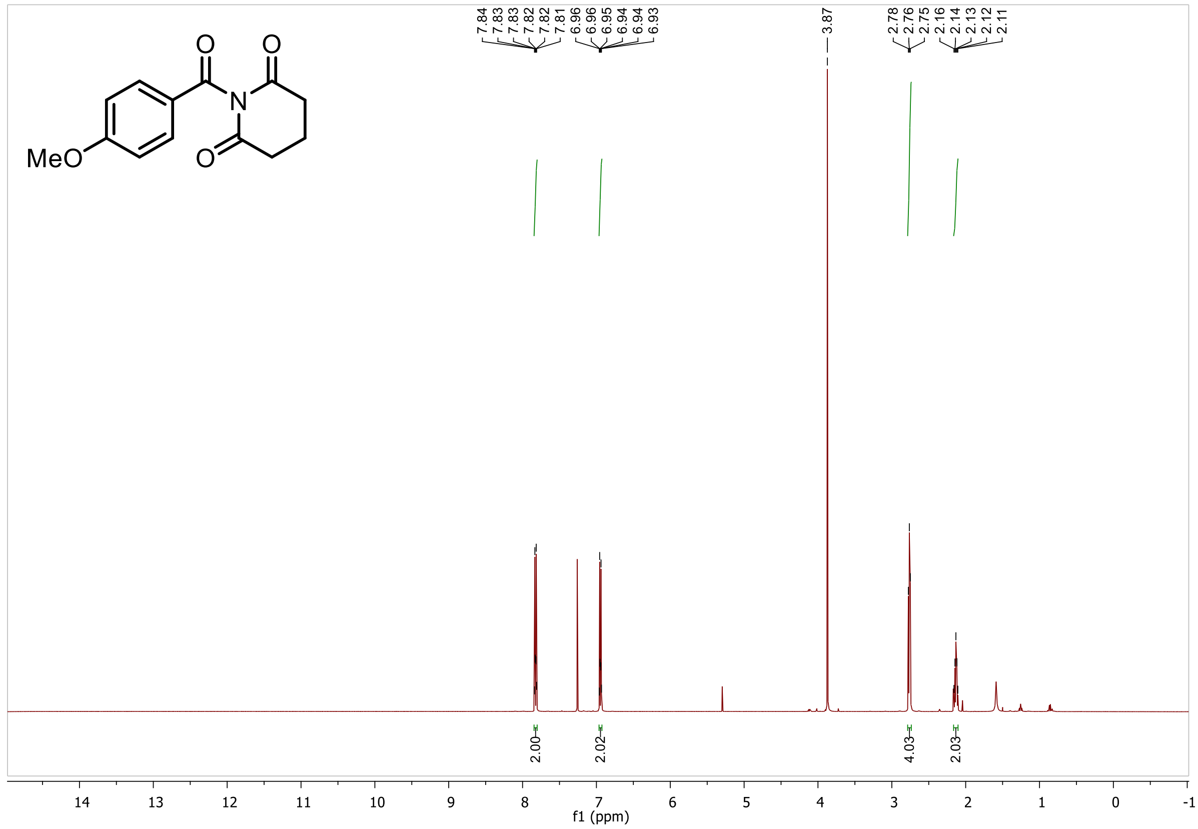

Figure S13. 1d, $^{13}C\{^1H\}$ NMR, 126 MHz, $CDCl_3$

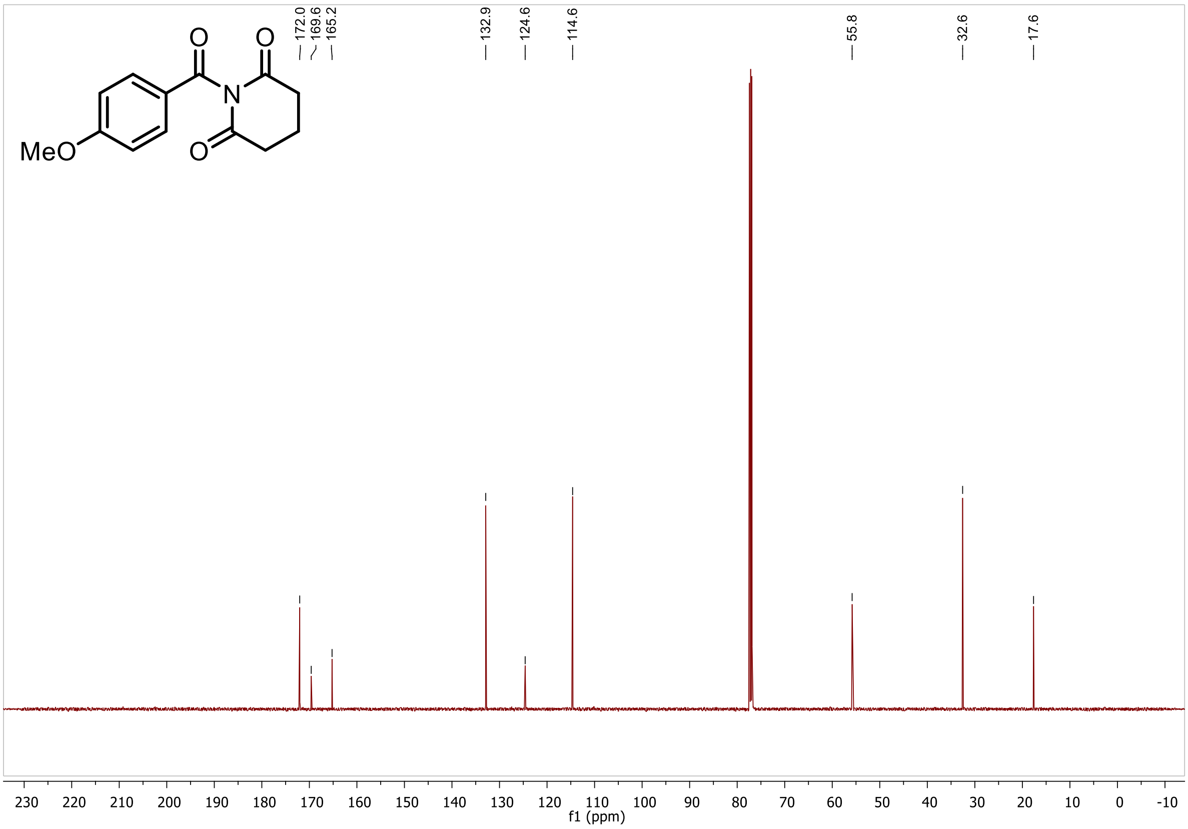

Figure S14. 1e, ^1H NMR, 500 MHz, $CDCl_3$

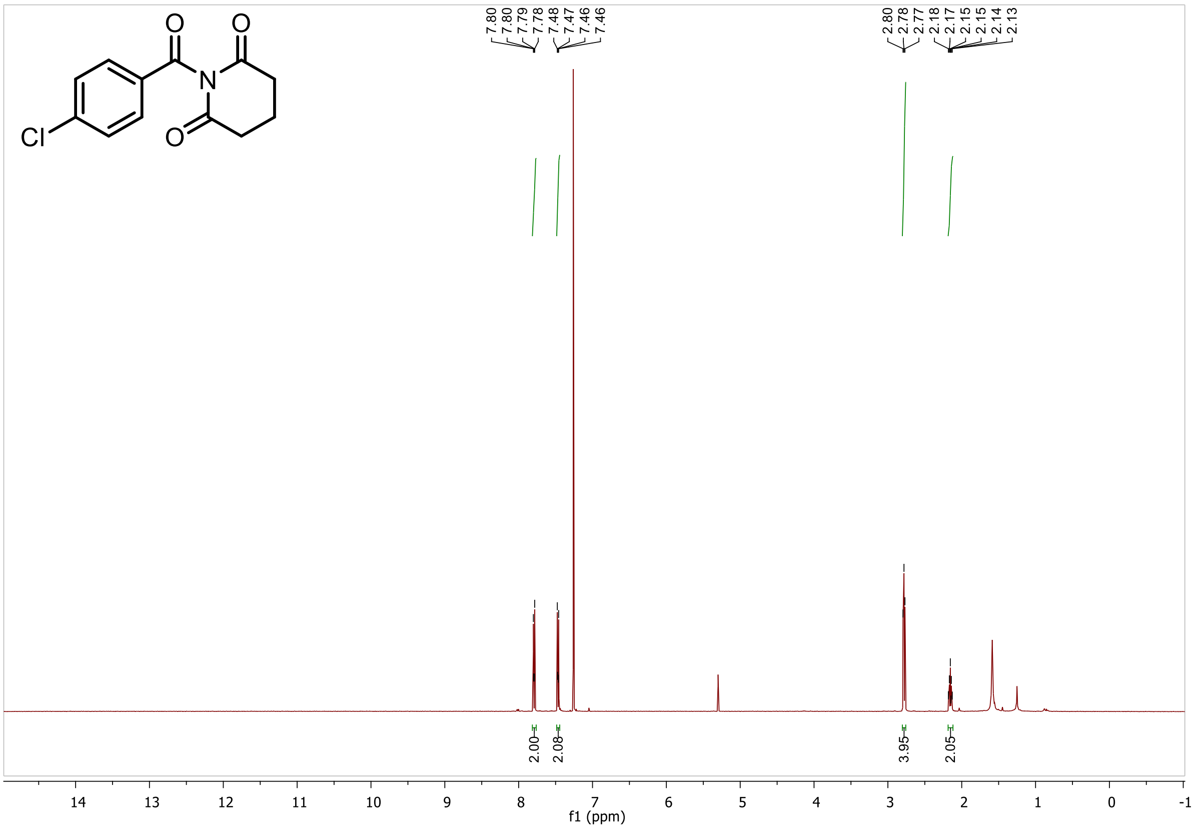

Figure S15. 1e, $^{13}C\{^{1}H\}$ NMR, 126 MHz, $CDCl_3$

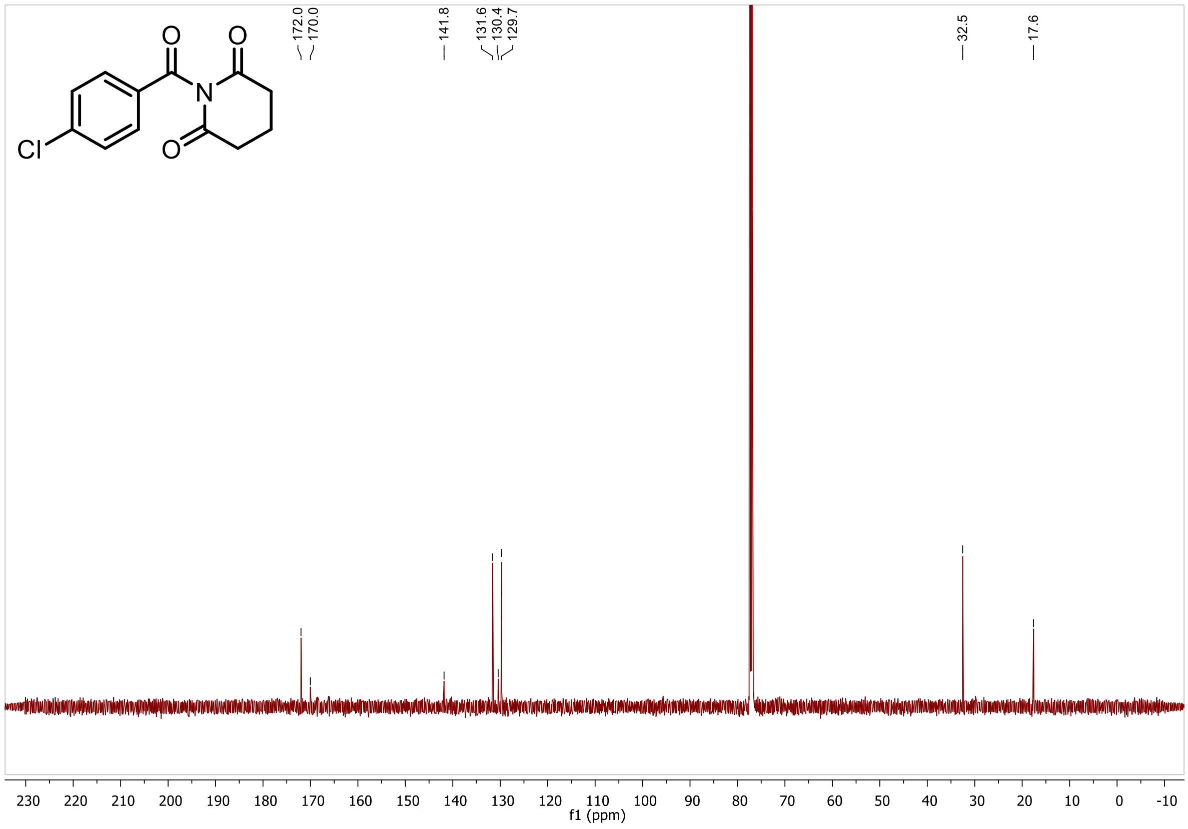

Figure S16. 1f, ^{1}H NMR, 500 MHz, $CDCl_3$

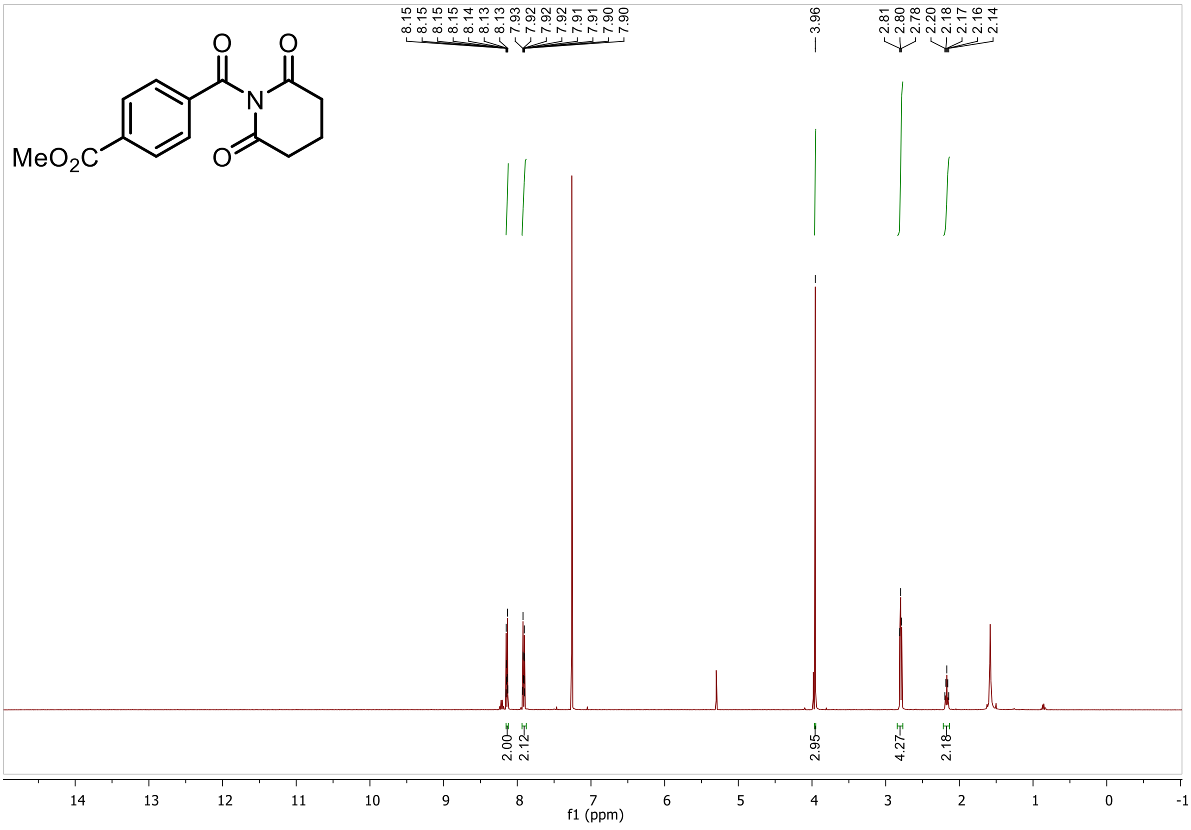

Figure S17. 1f, ^{13}C {^{1}H} NMR, 126 MHz, $CDCl_3$

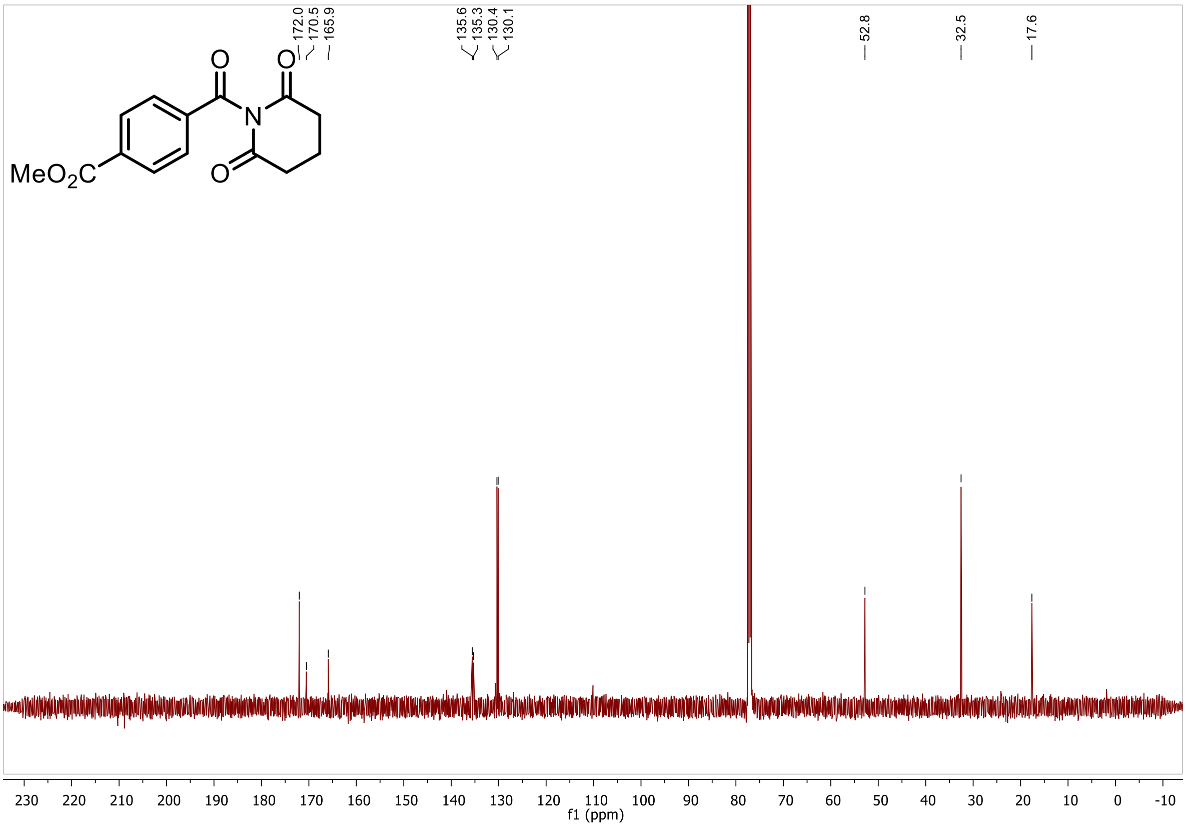

Figure S18. 1g, ^{1}H NMR, 500 MHz, $CDCl_3$

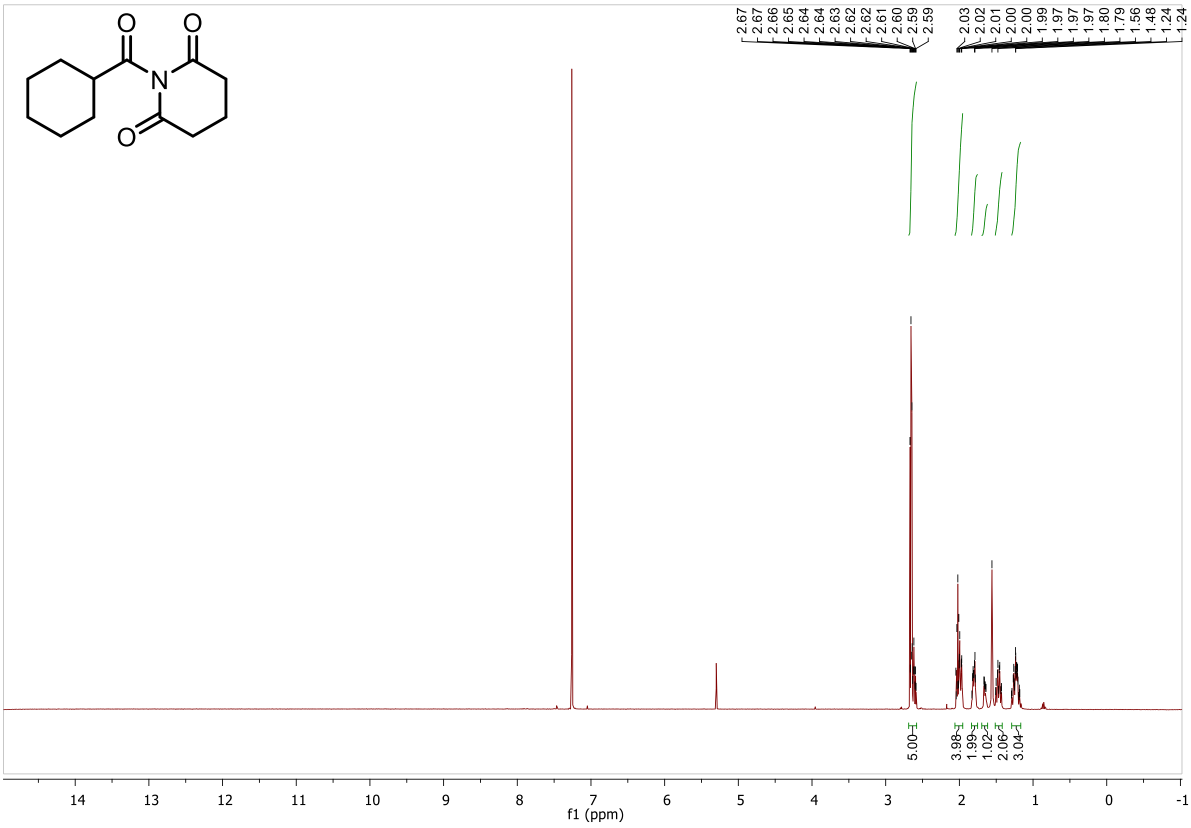

Figure S19. 1g, $^{13}C\{^1H\}$ NMR, 126 MHz, $CDCl_3$

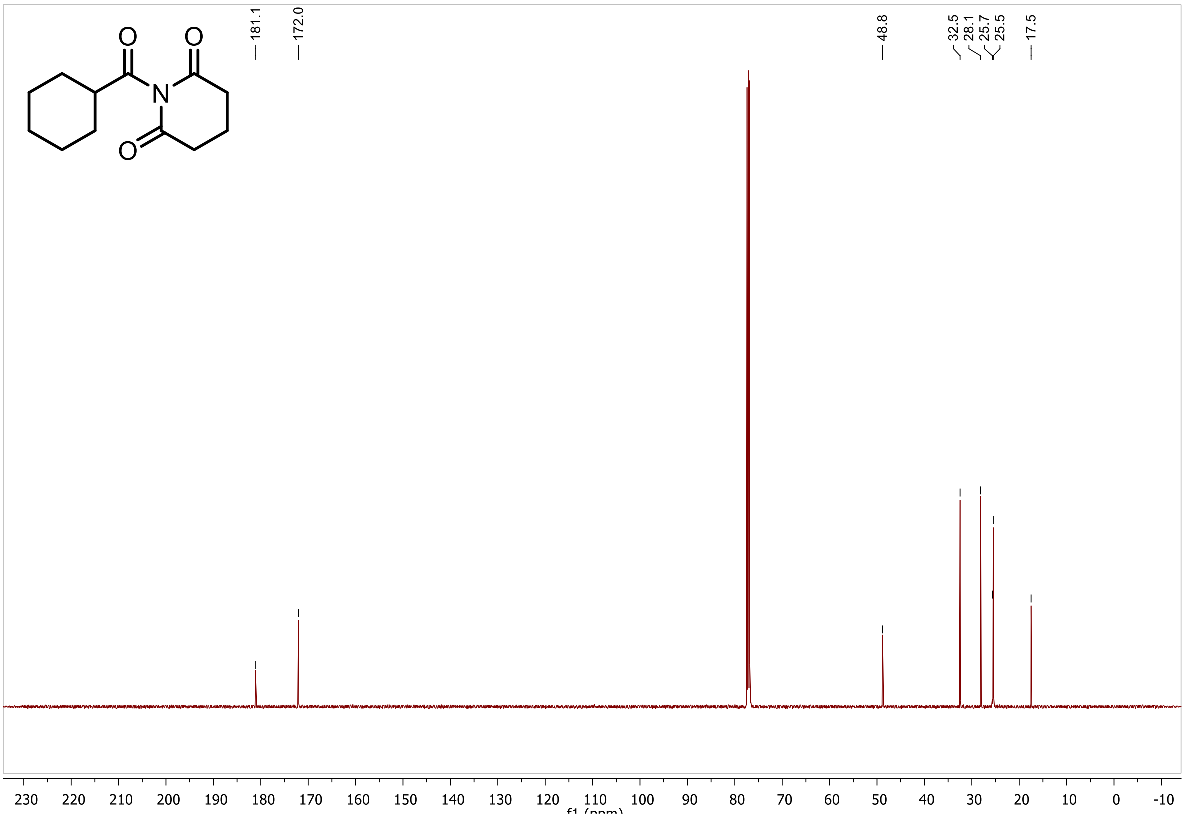

Figure S20. 1h, ^1H NMR, 500 MHz, $CDCl_3$

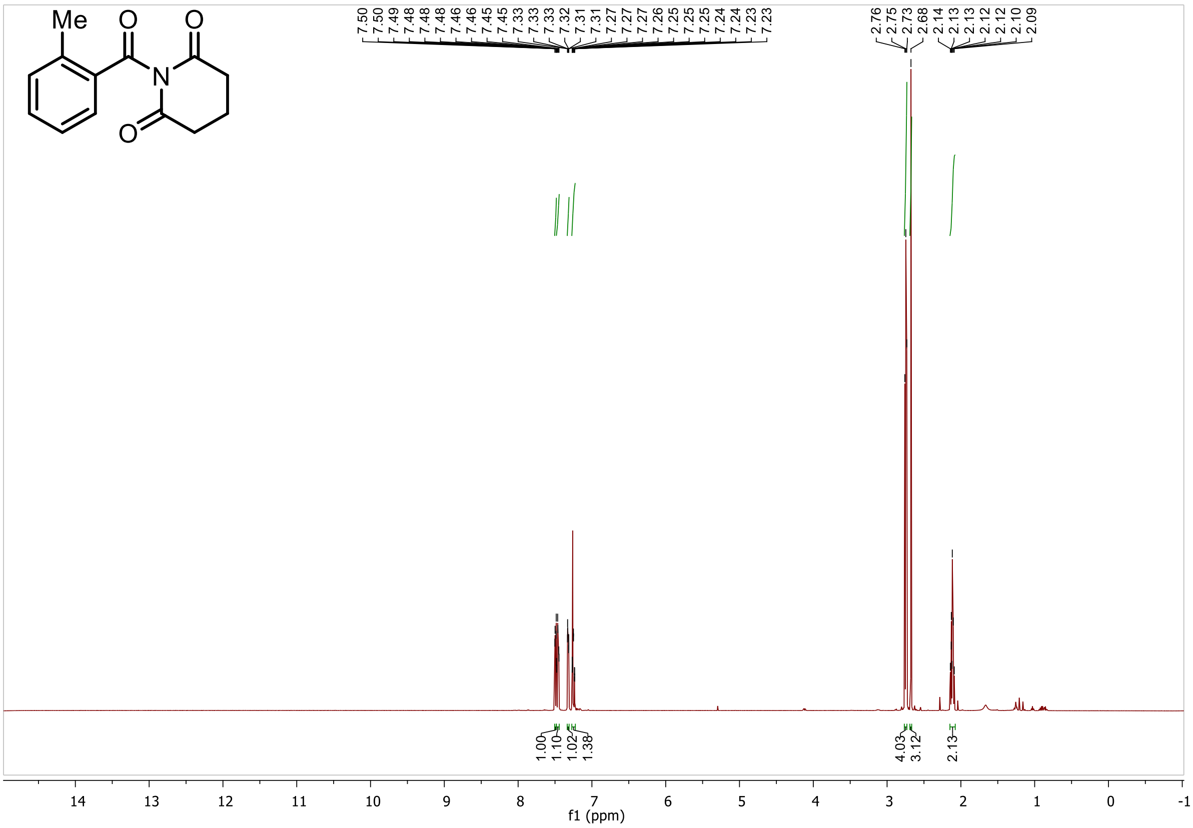

Figure S21. 1h, $^{13}C\ \{^1H\}$ NMR, 126 MHz, $CDCl_3$

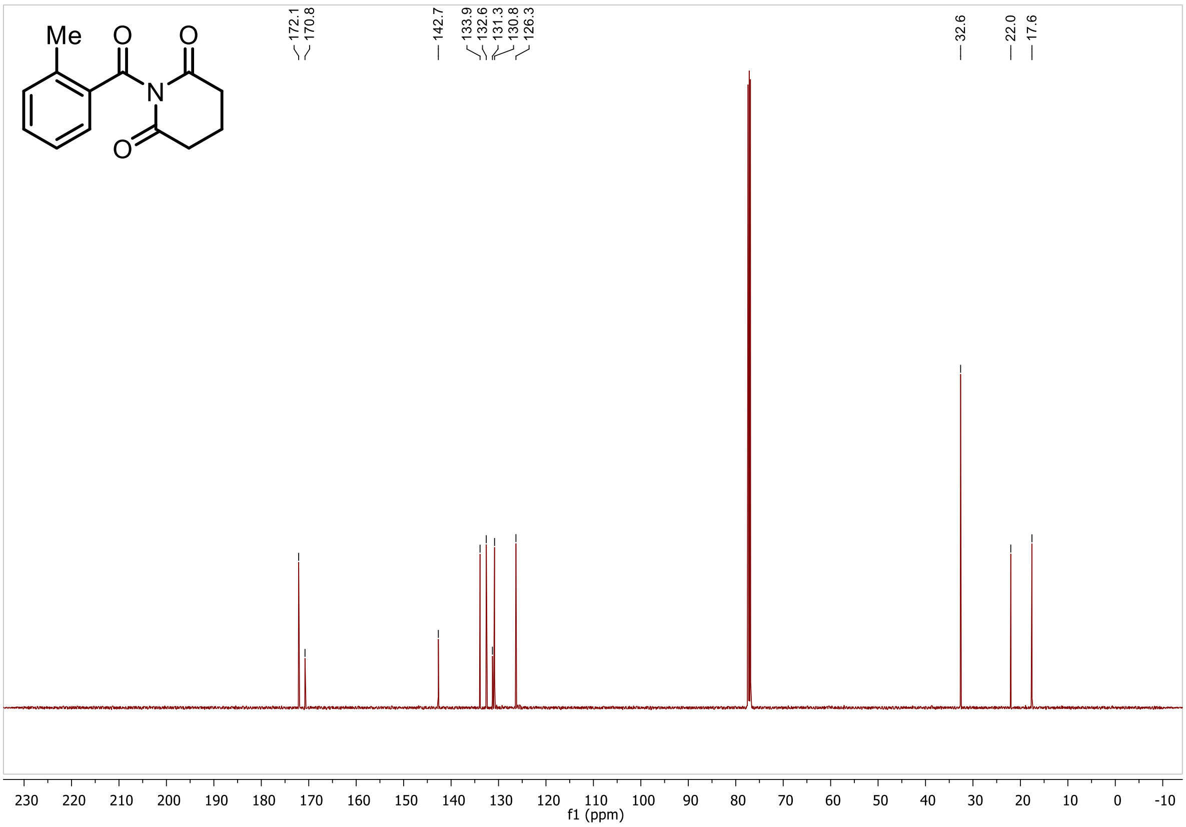

Figure S22. 1i, ^1H NMR, 500 MHz, $CDCl_3$

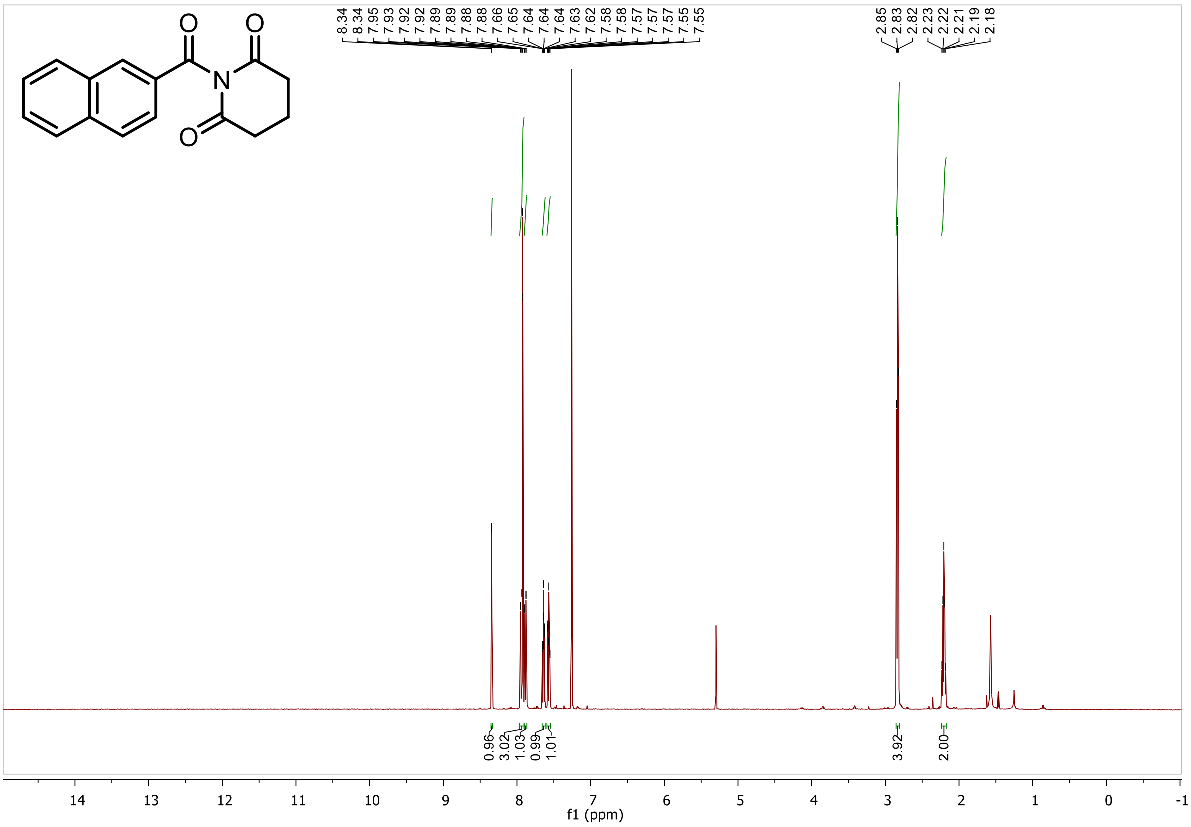

Figure S23. 1i, ^{13}C {^{1}H} NMR, 126 MHz, $CDCl_3$

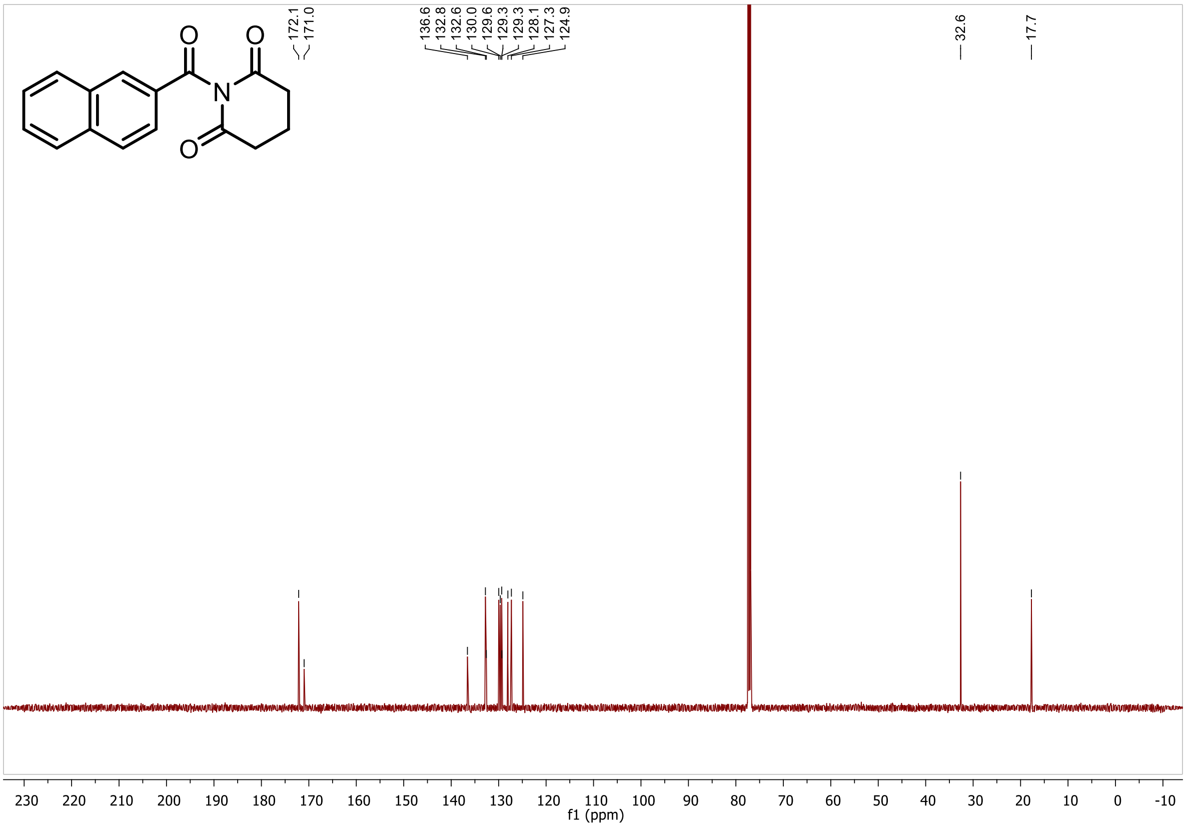

Figure S24. 1j, ^{1}H NMR, 500 MHz, $CDCl_3$

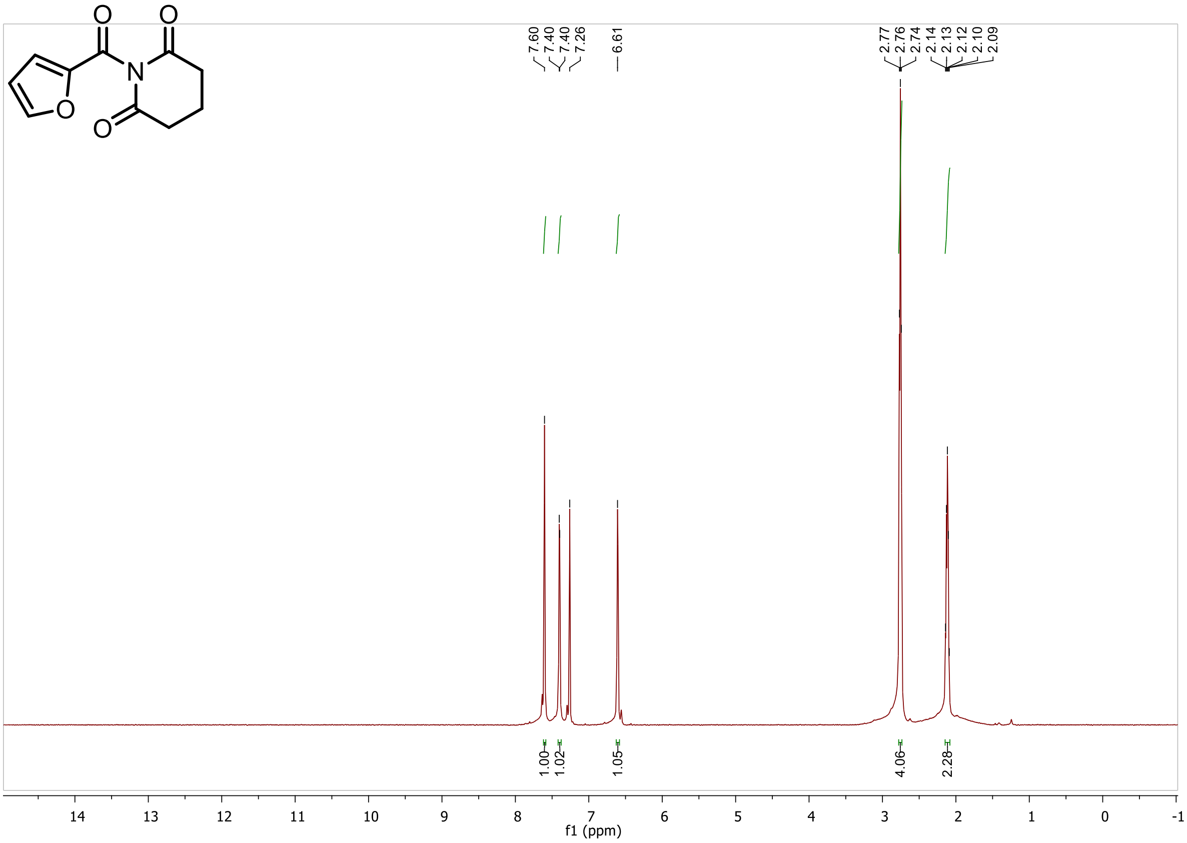

Figure S25. 1j, ^{13}C {^{1}H} NMR, 126 MHz, $CDCl_3$

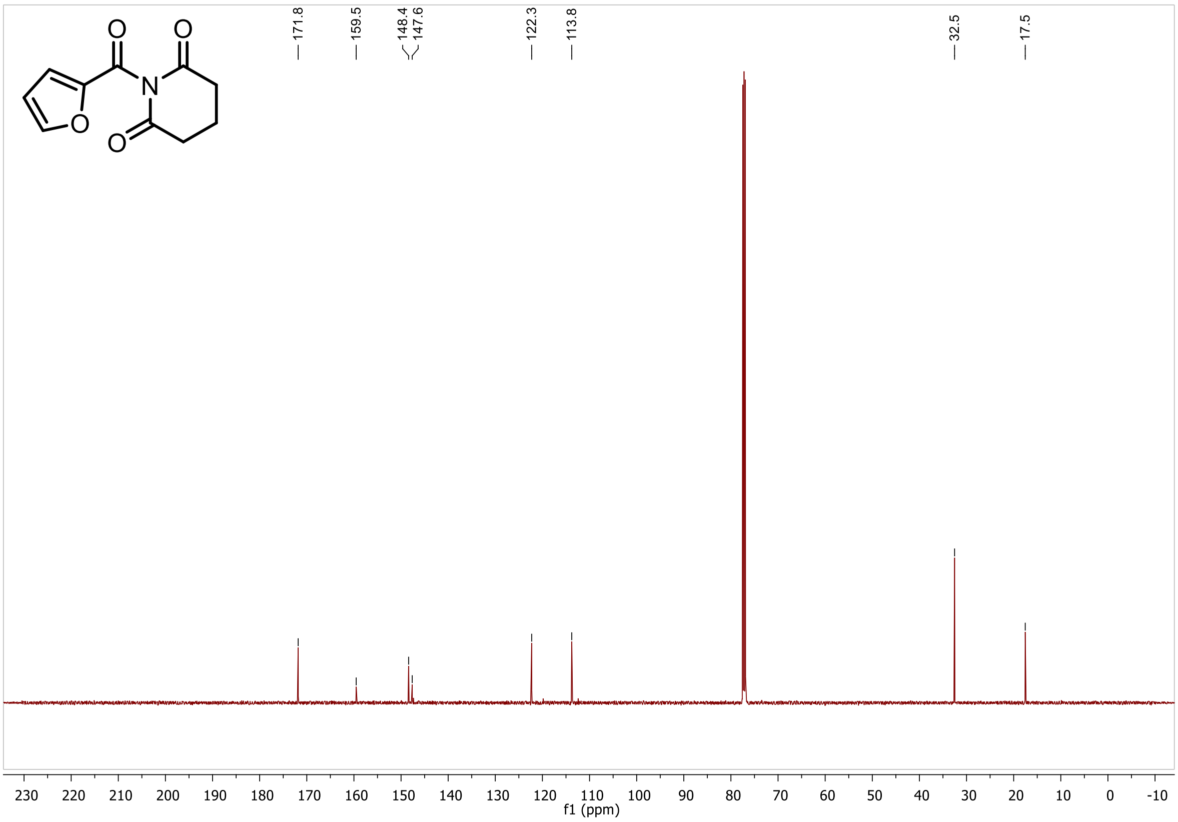

Figure S26. 1k, ^{1}H NMR, 500 MHz, $CDCl_3$

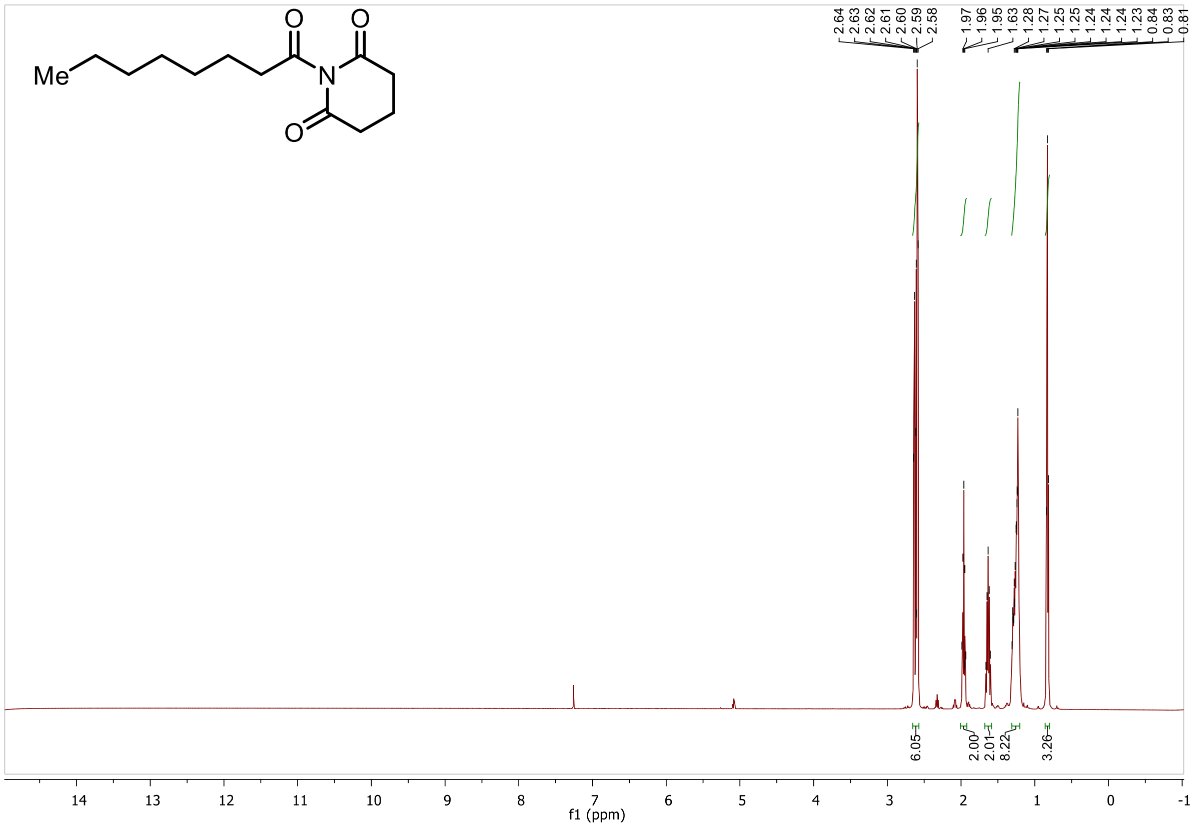

Figure S27. 1k, $^{13}C\ \{^1H\}$ NMR, 126 MHz, $CDCl_3$

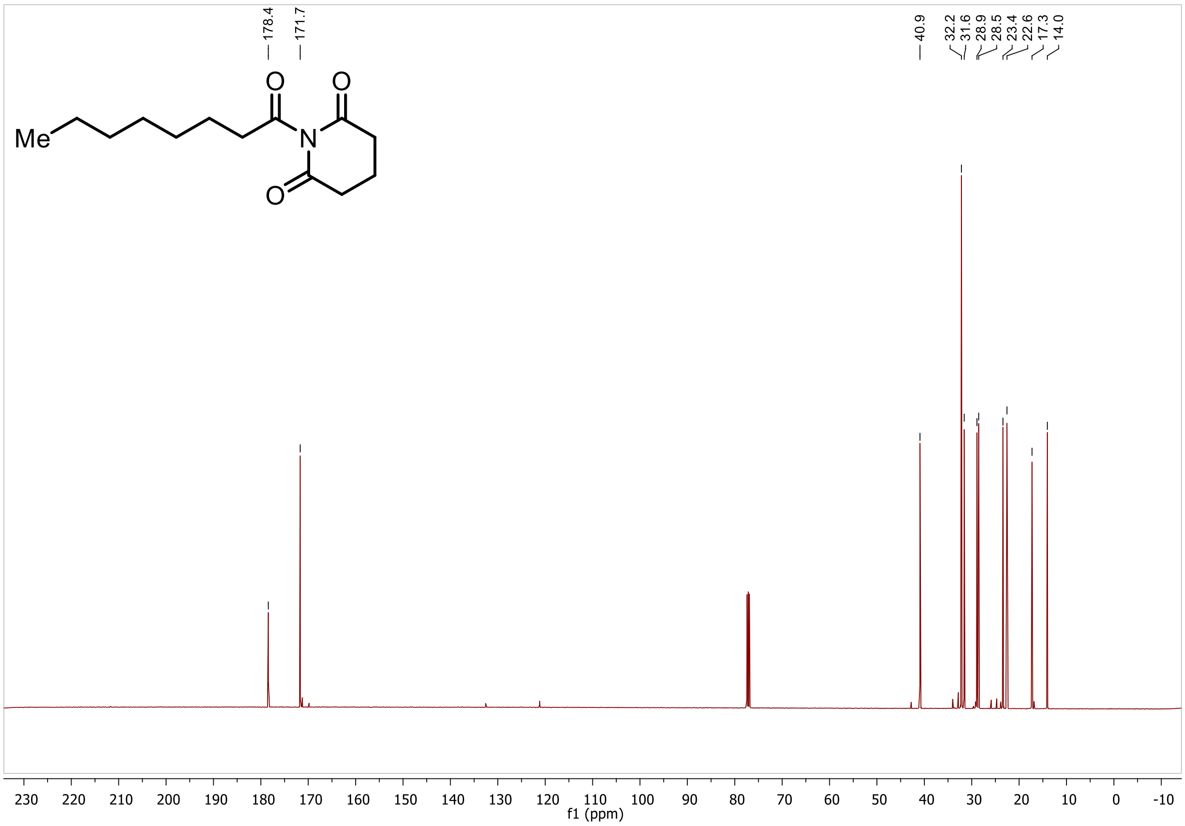

Figure S28. 1l, ^1H NMR, 500 MHz, $CDCl_3$

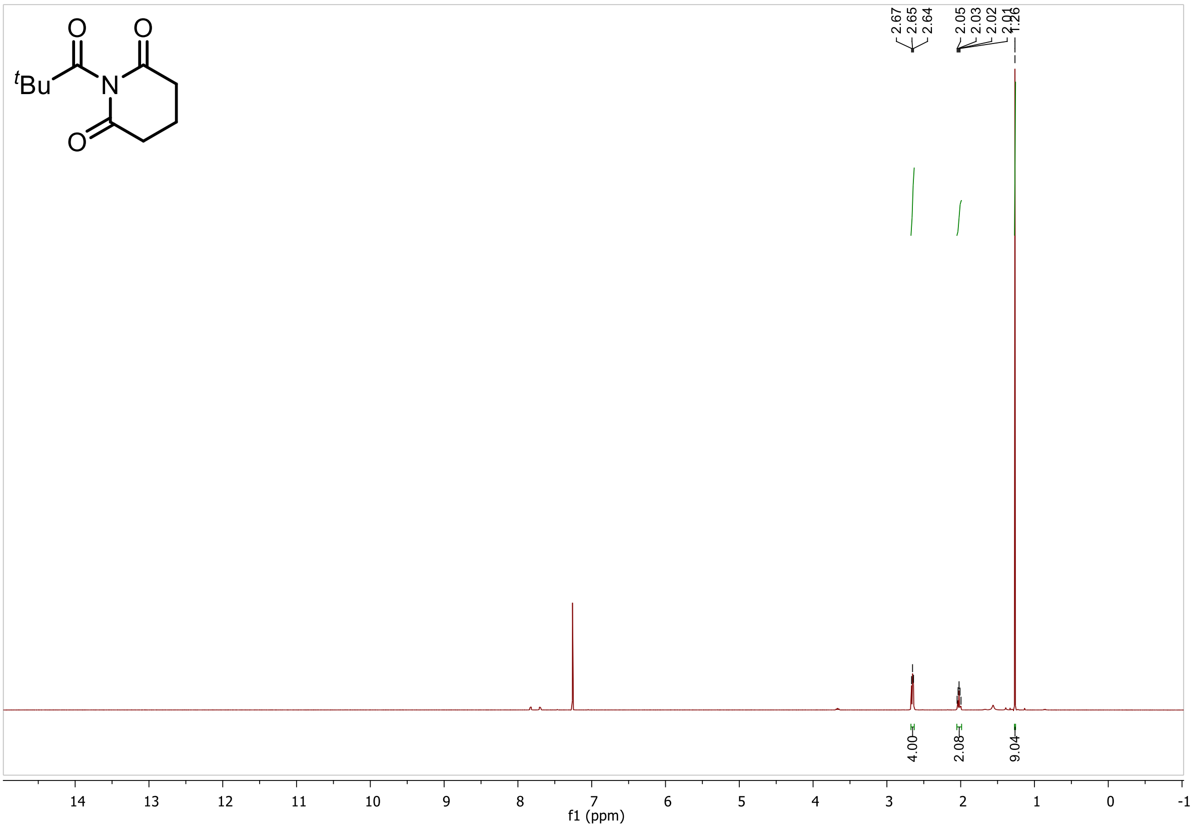

Figure S29. 1l, $^{13}C\ \{^1H\}$ NMR, 126 MHz, $CDCl_3$

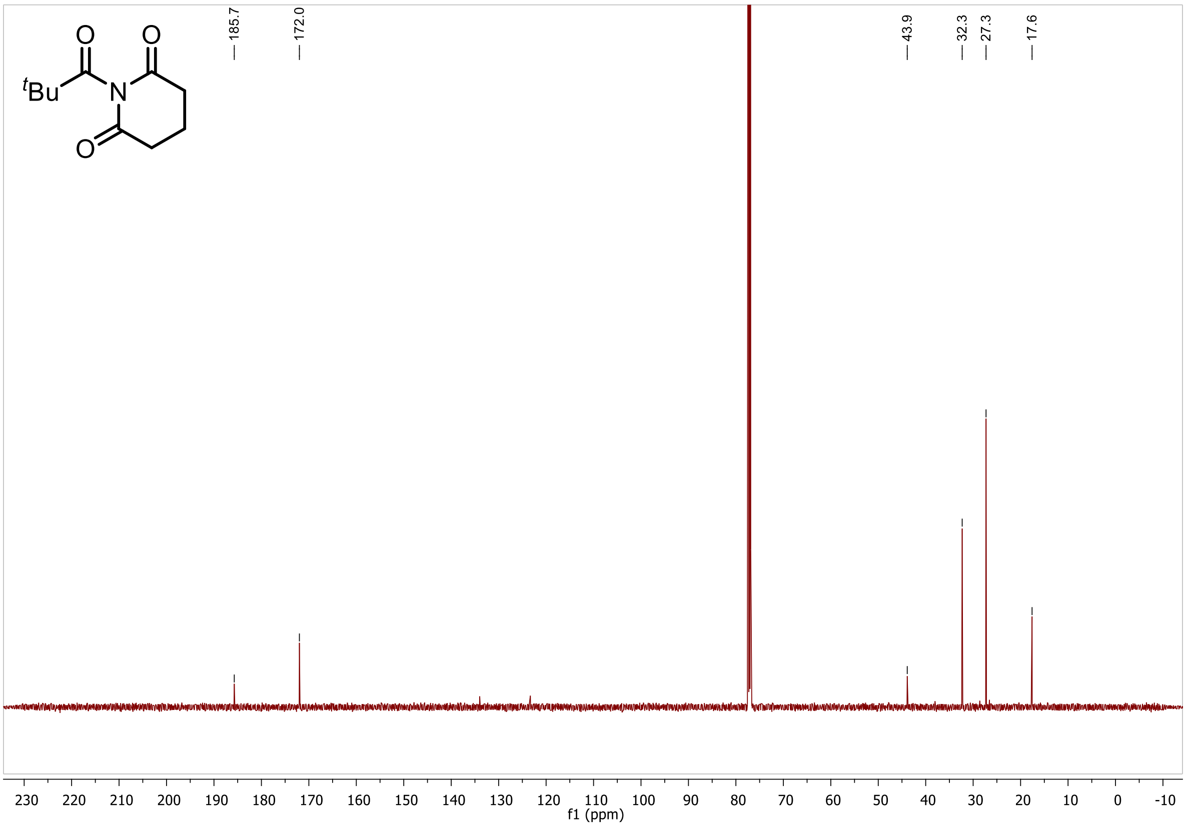

Figure S30. 1ac, ^1H NMR, 500 MHz, $CDCl_3$

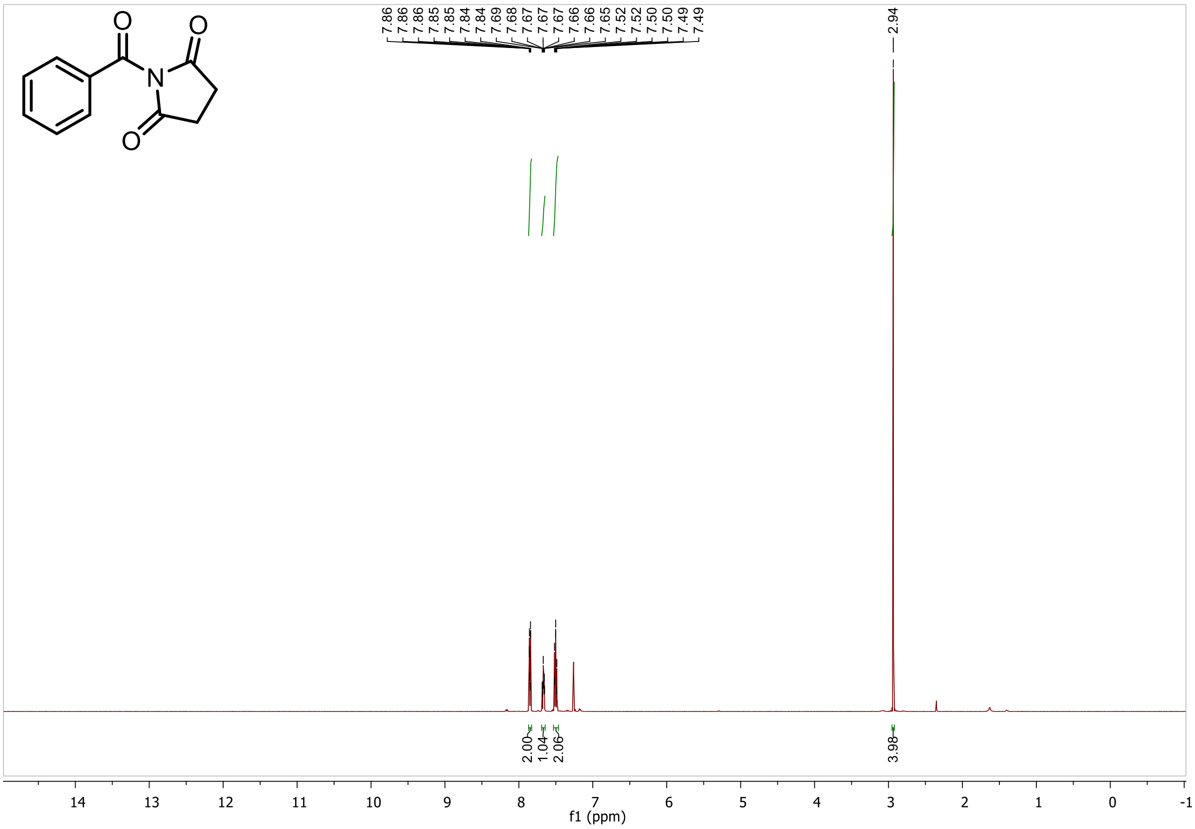

Figure S31. 1ac, $^{13}C\{^1H\}$ NMR, 126 MHz, $CDCl_3$

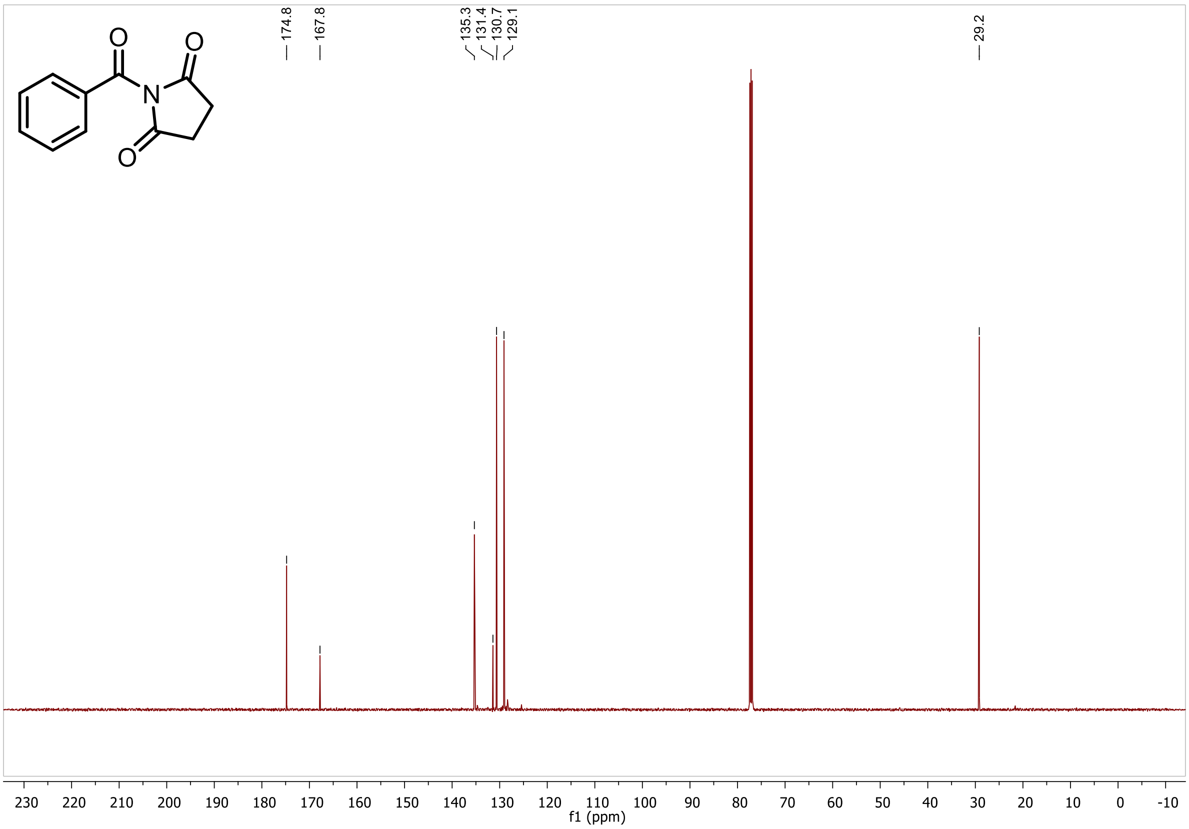

Figure S32. 1ab, ^1H NMR, 500 MHz, $CDCl_3$

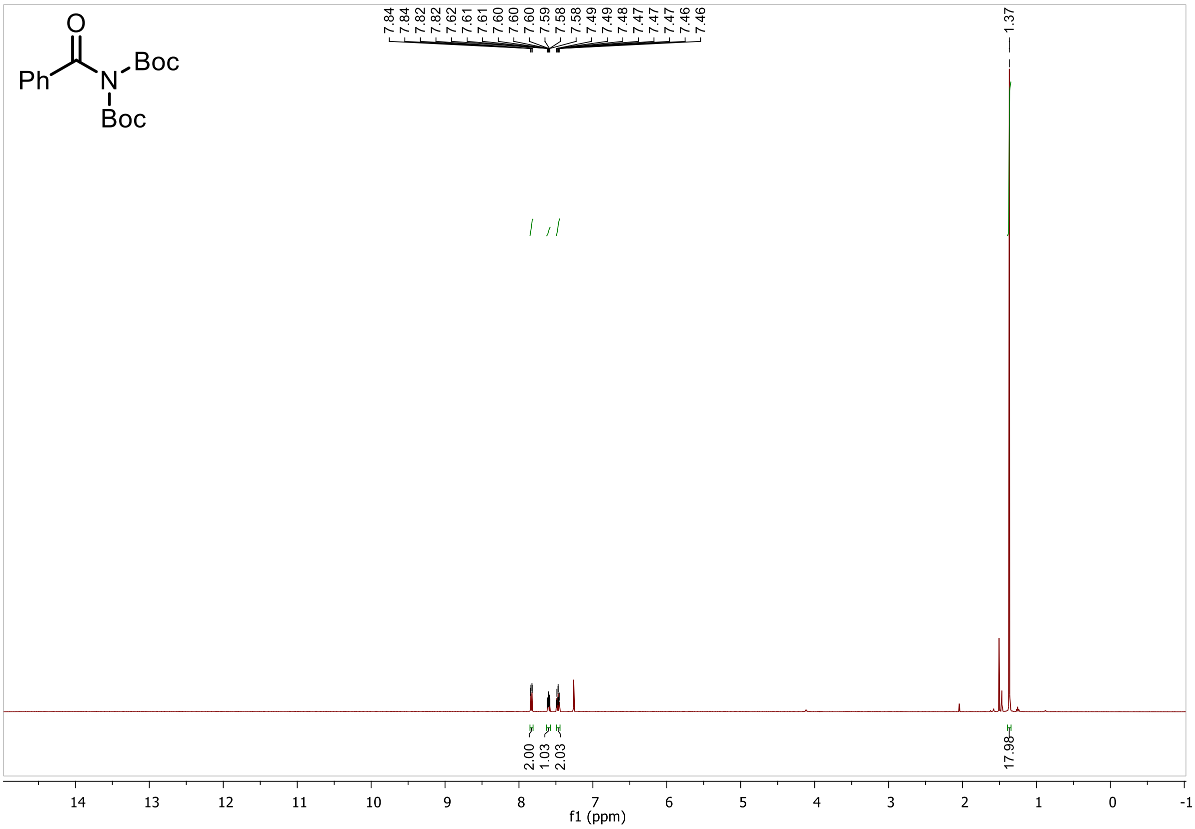

Figure S33.1ab, $^{13}C\{^1H\}$ NMR, 126 MHz, $CDCl_3$

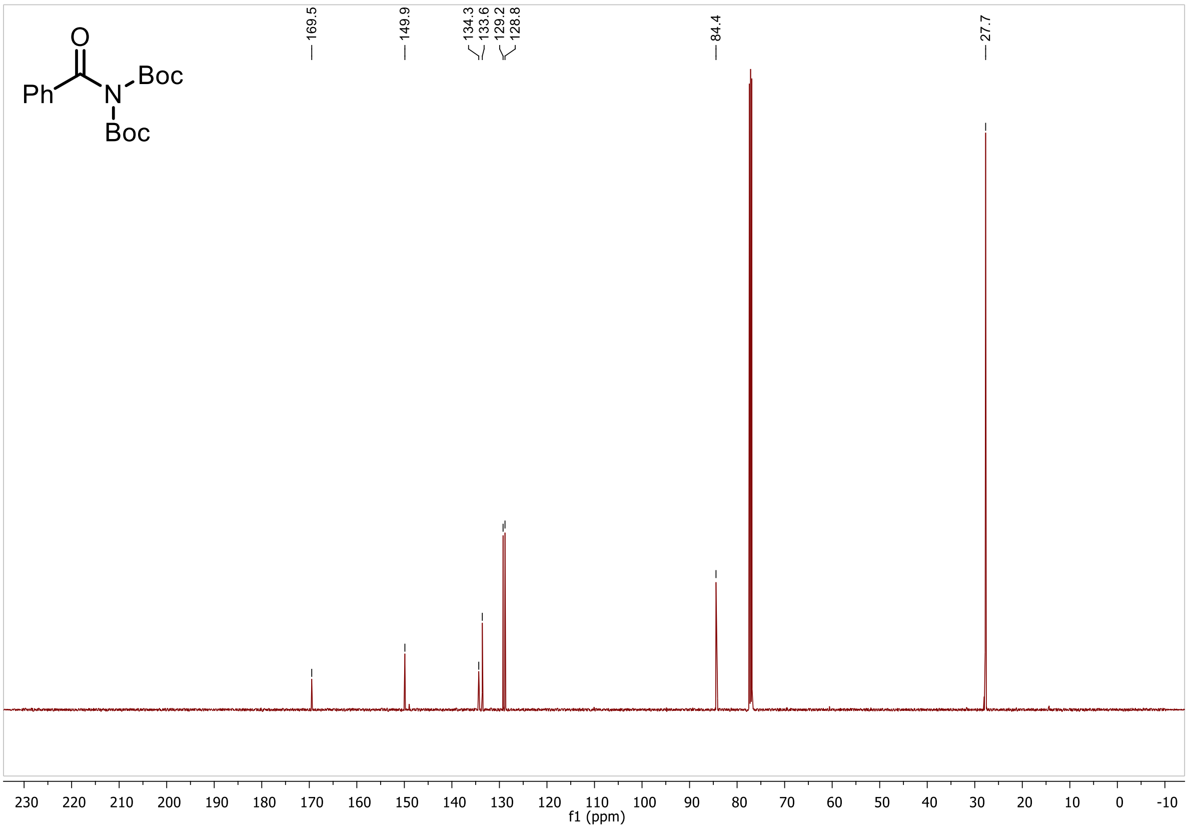

Figure S34. 1ad, ^1H NMR, 500 MHz, $CDCl_3$

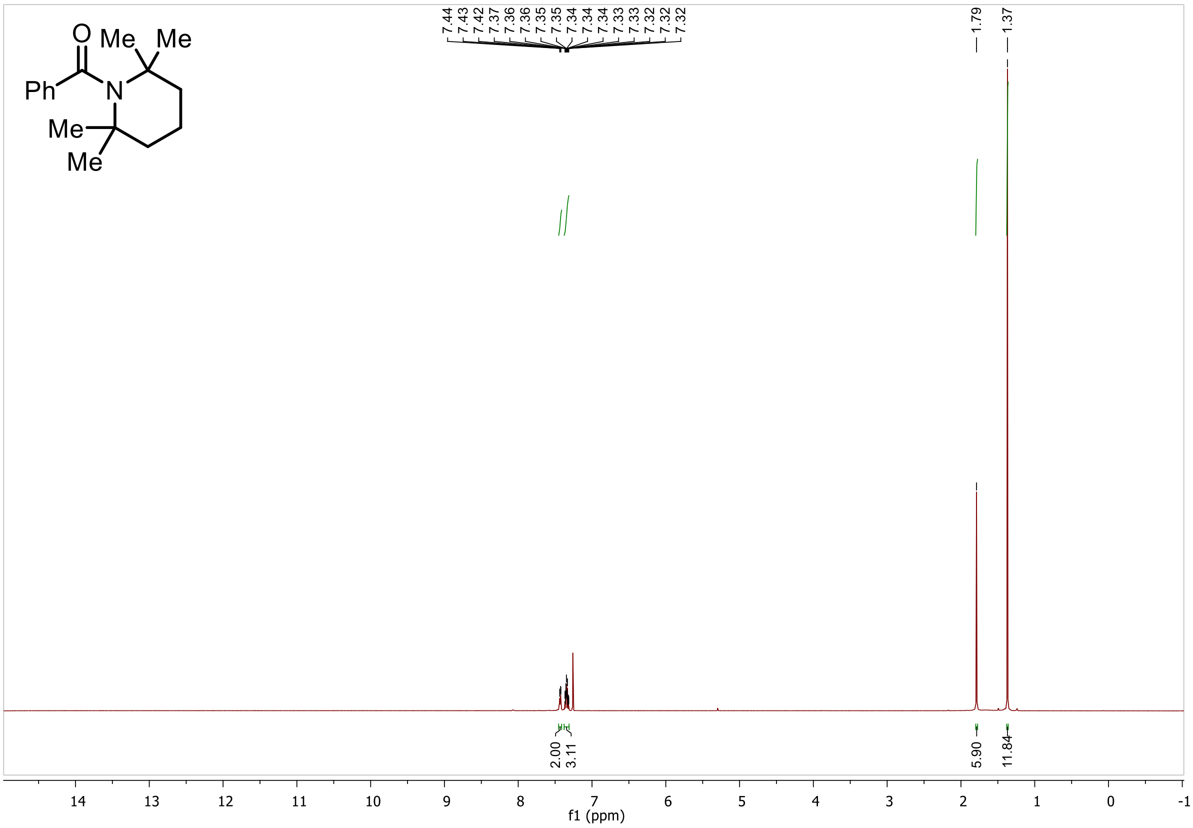

Figure S35. 1ad, $^{13}C\ \{^{1}H\}$ NMR, 126 MHz, $CDCl_3$

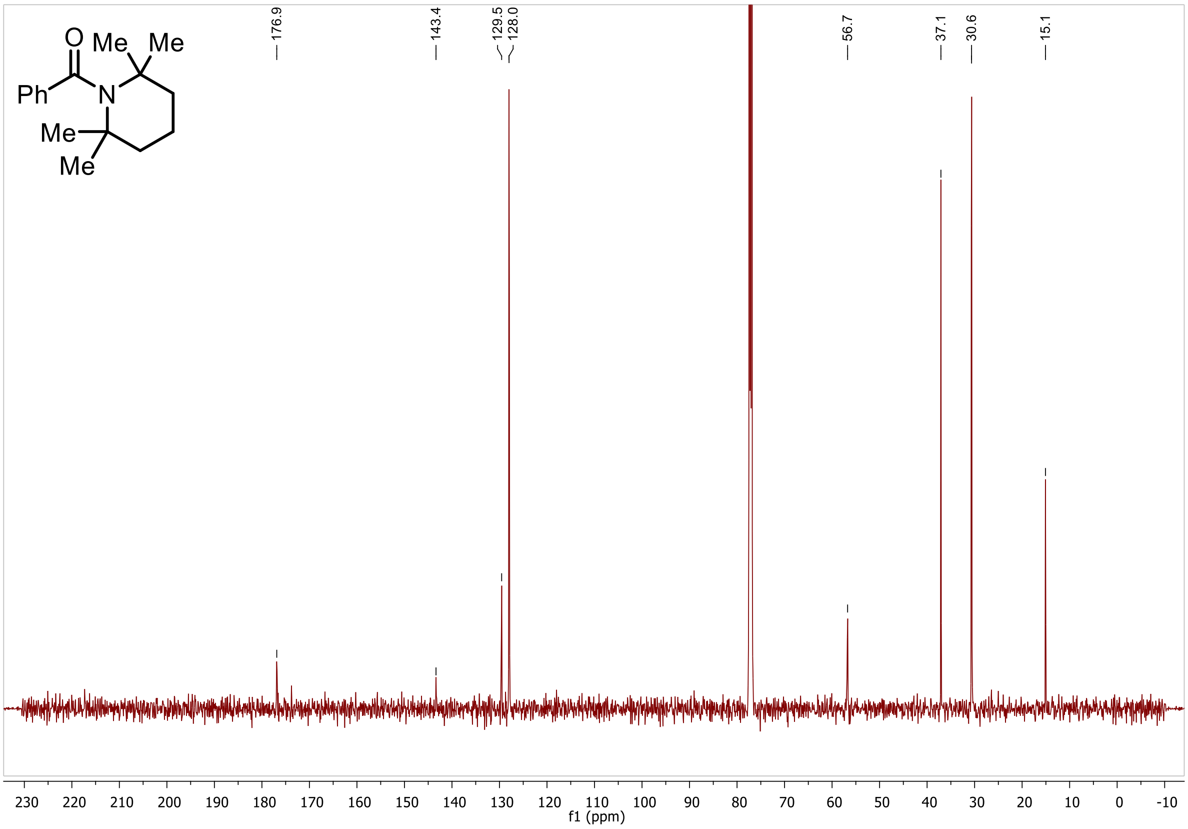

Figure S36. 1af, ^{1}H NMR, 500 MHz, $CDCl_3$

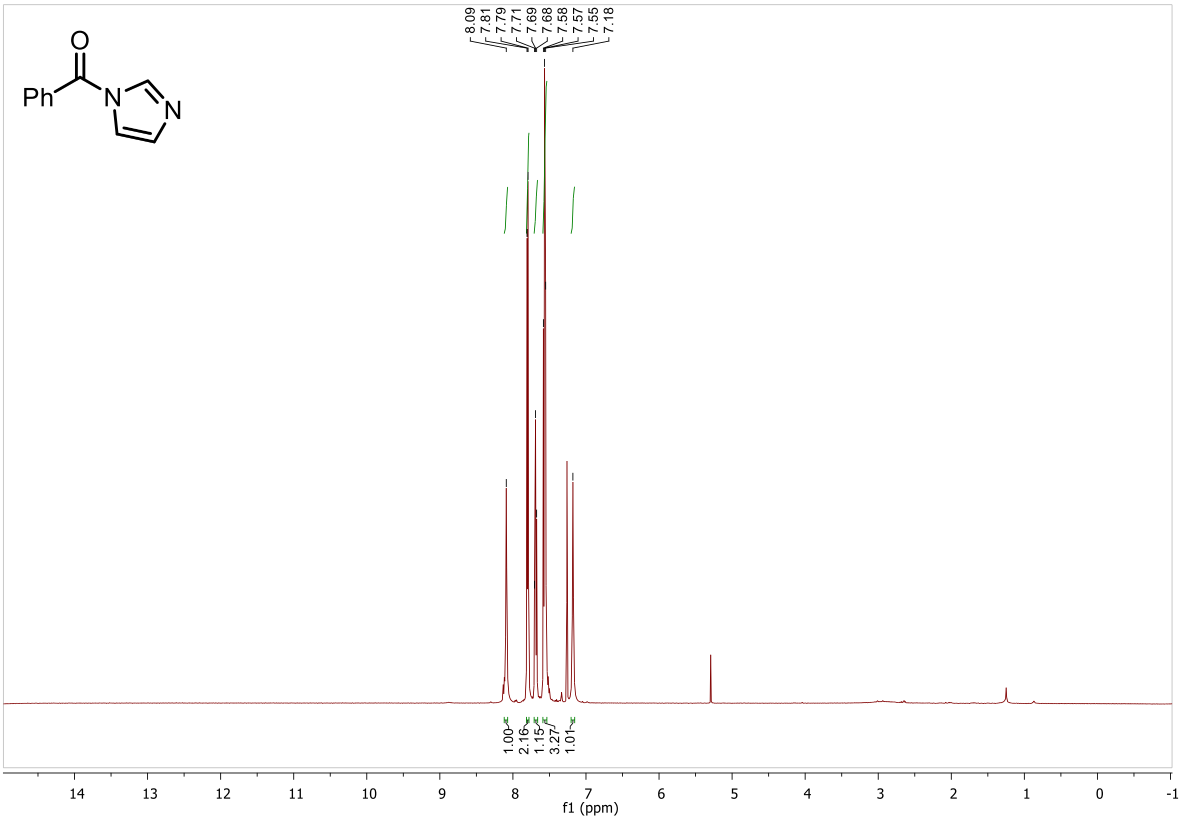

Figure S37. 1af, $^{13}C\ \{^{1}H\}$ NMR, 126 MHz, $CDCl_3$

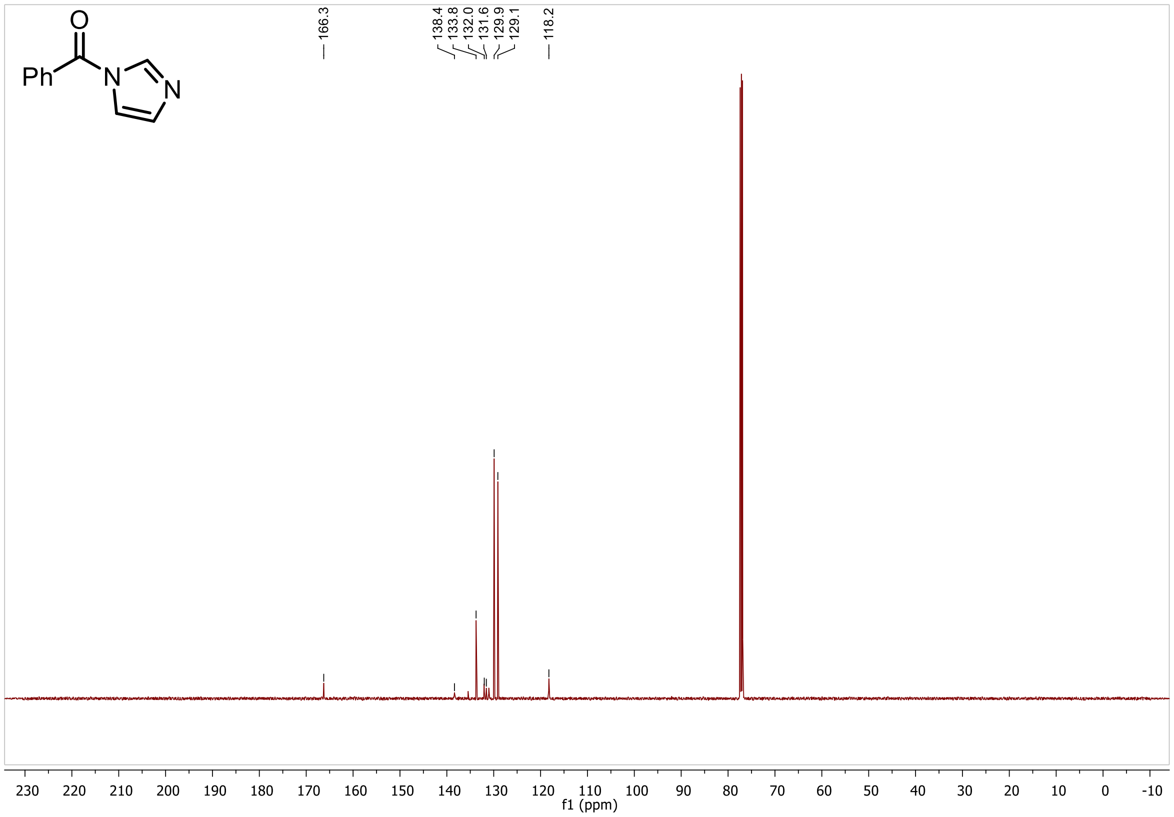

Figure S38. 1m, ^{1}H NMR, 500 MHz, $CDCl_3$

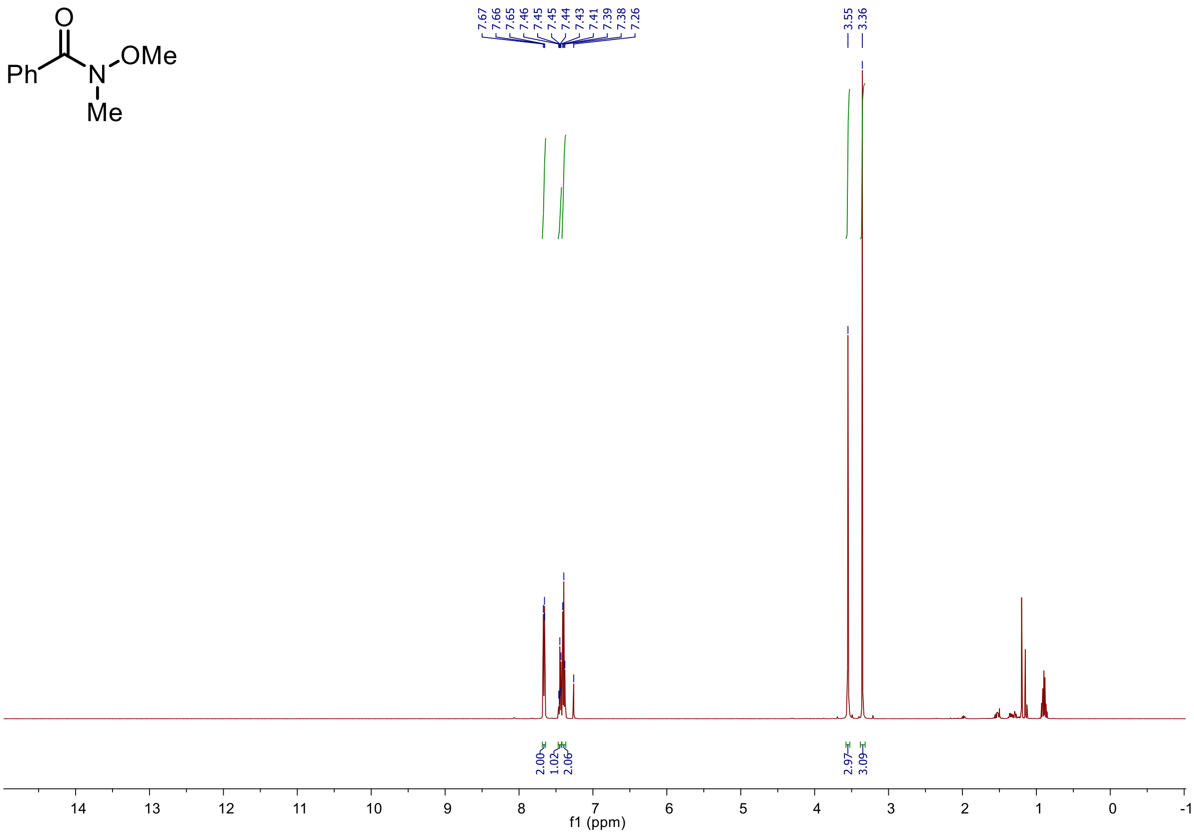

Figure S39. 1m, ^{13}C {^{1}H} NMR, 126 MHz, $CDCl_3$

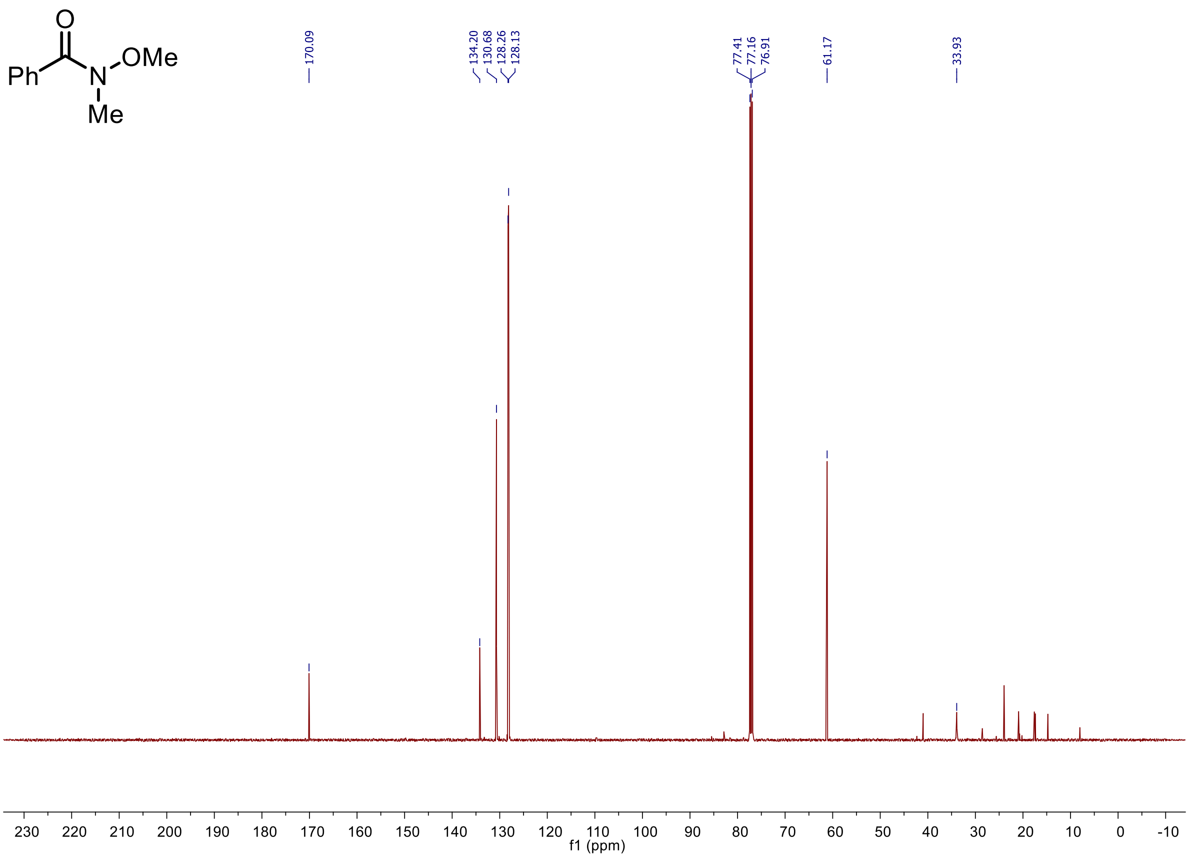

Figure S40. 1o, ^{1}H NMR, 500 MHz, $CDCl_3$

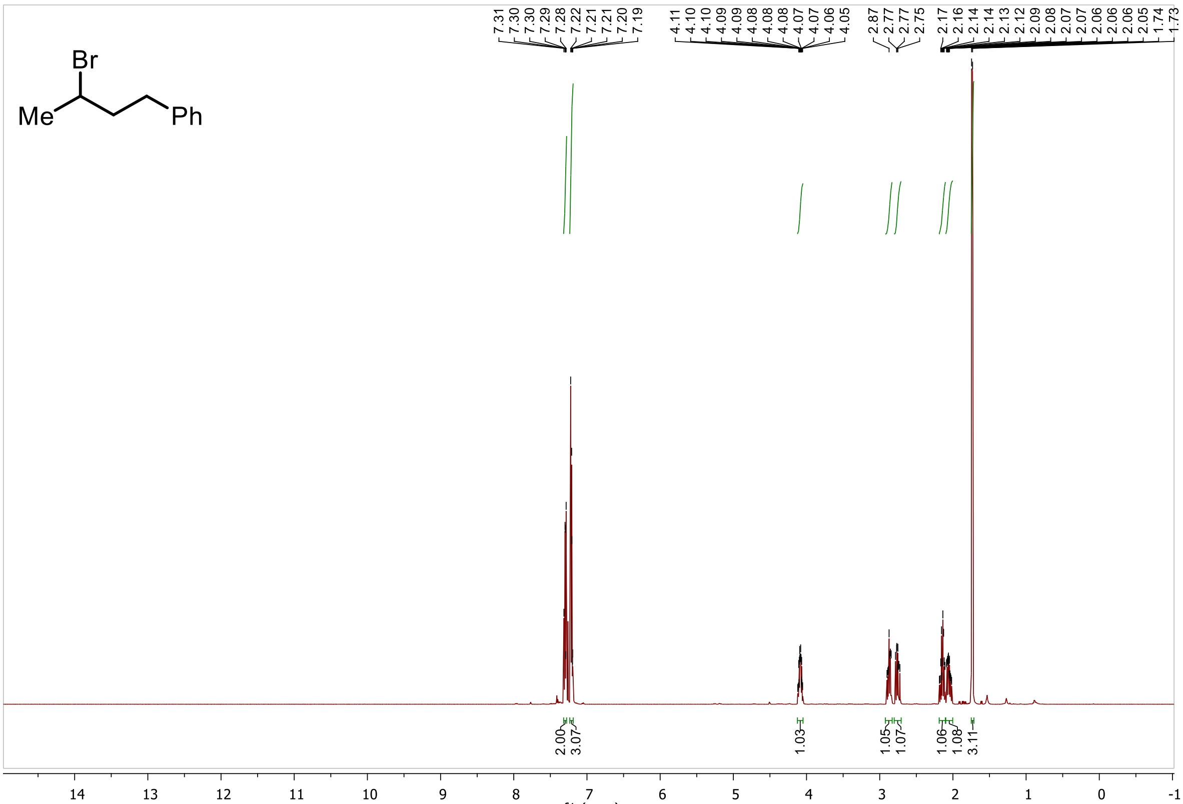

Figure S41. 1o, ^{13}C {^{1}H} NMR, 126 MHz, $CDCl_3$

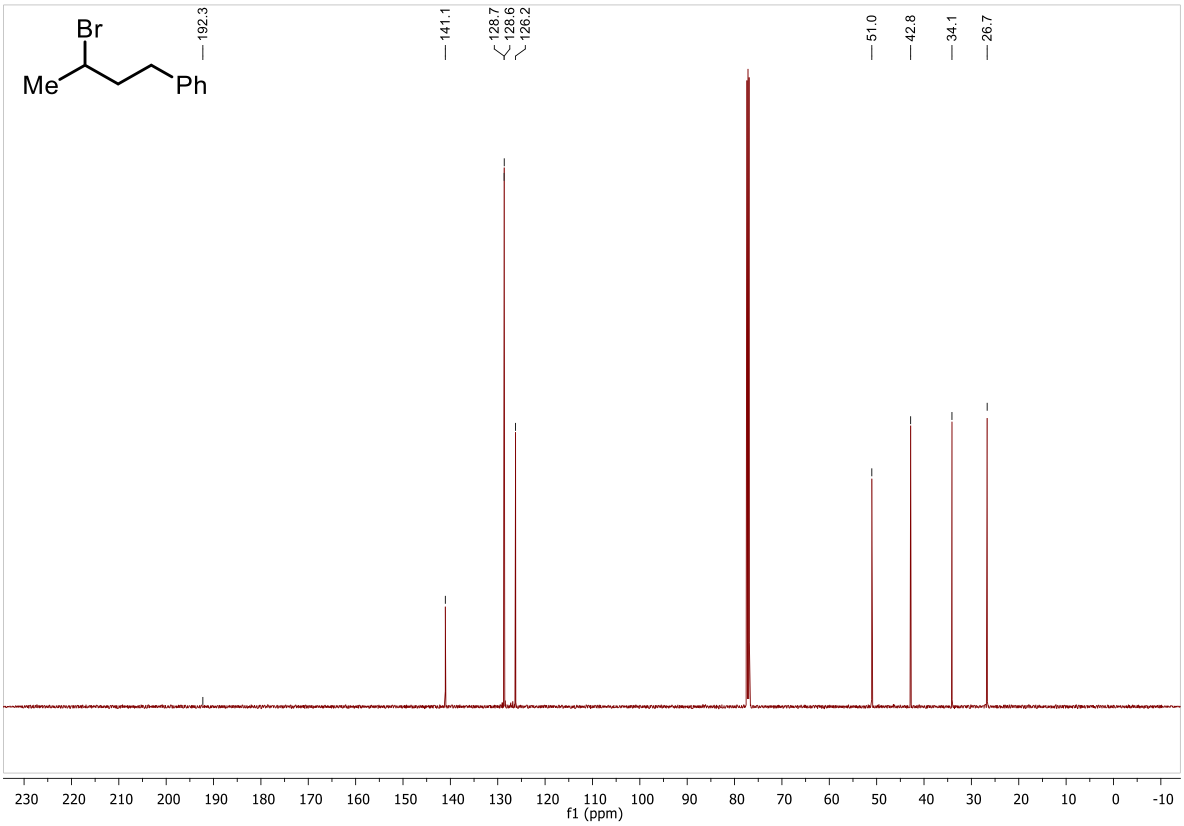

Figure S42. 3b, ^{1}H NMR, 500 MHz, $CDCl_3$

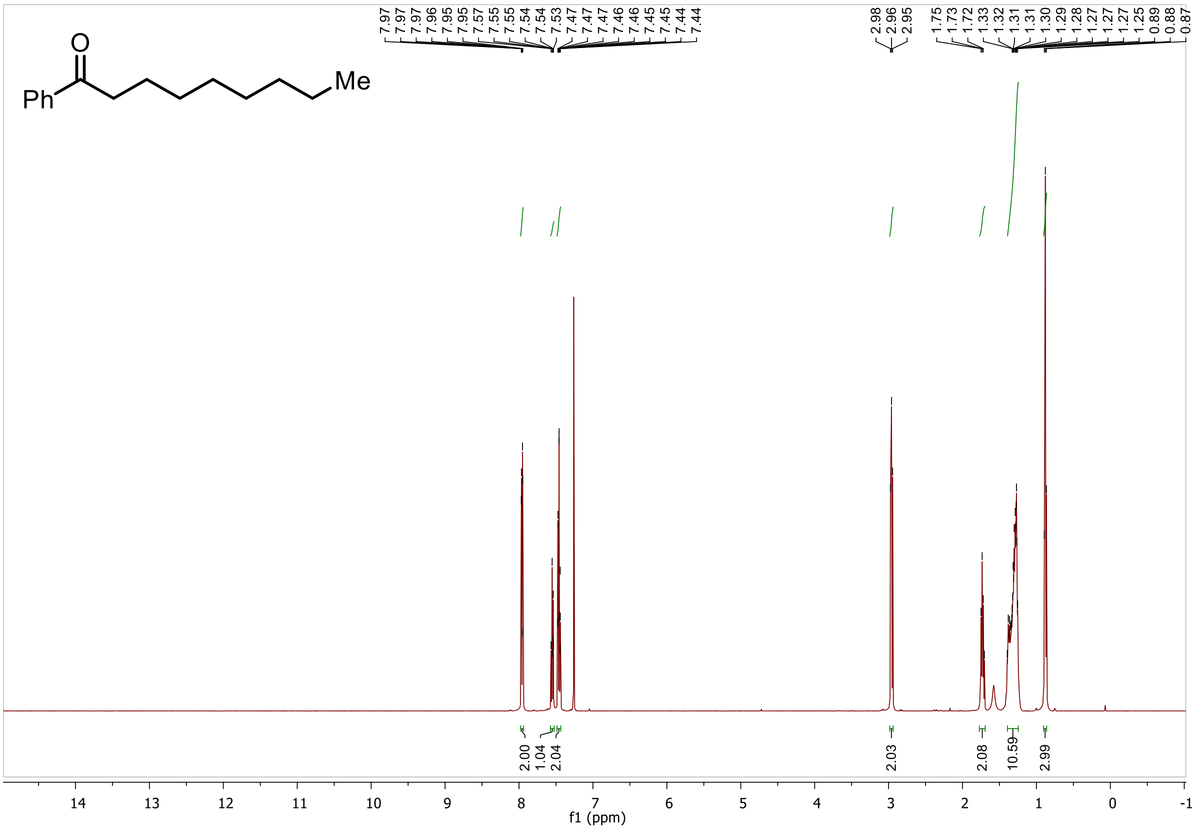

Figure S43. 3b, ^{13}C {^{1}H} NMR, 126 MHz, $CDCl_3$

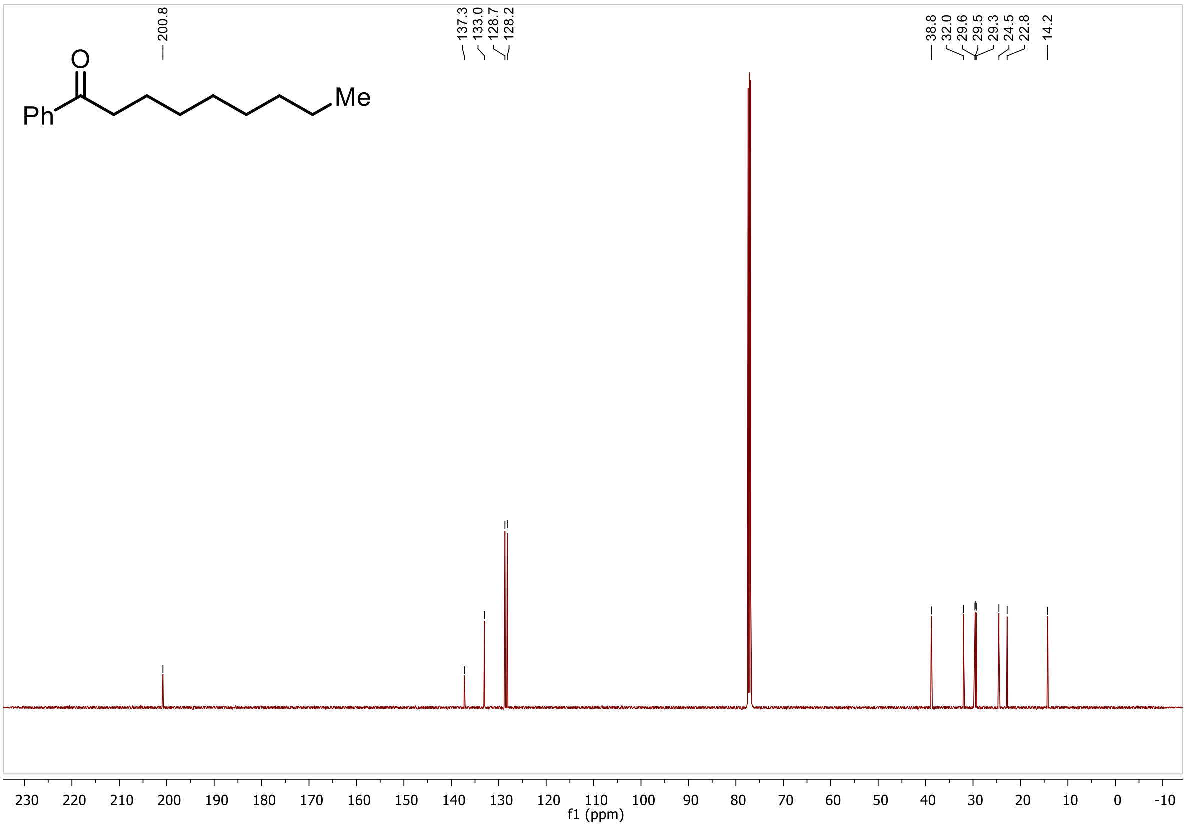

Figure S44. 3c, ^{1}H NMR, 500 MHz, $CDCl_3$

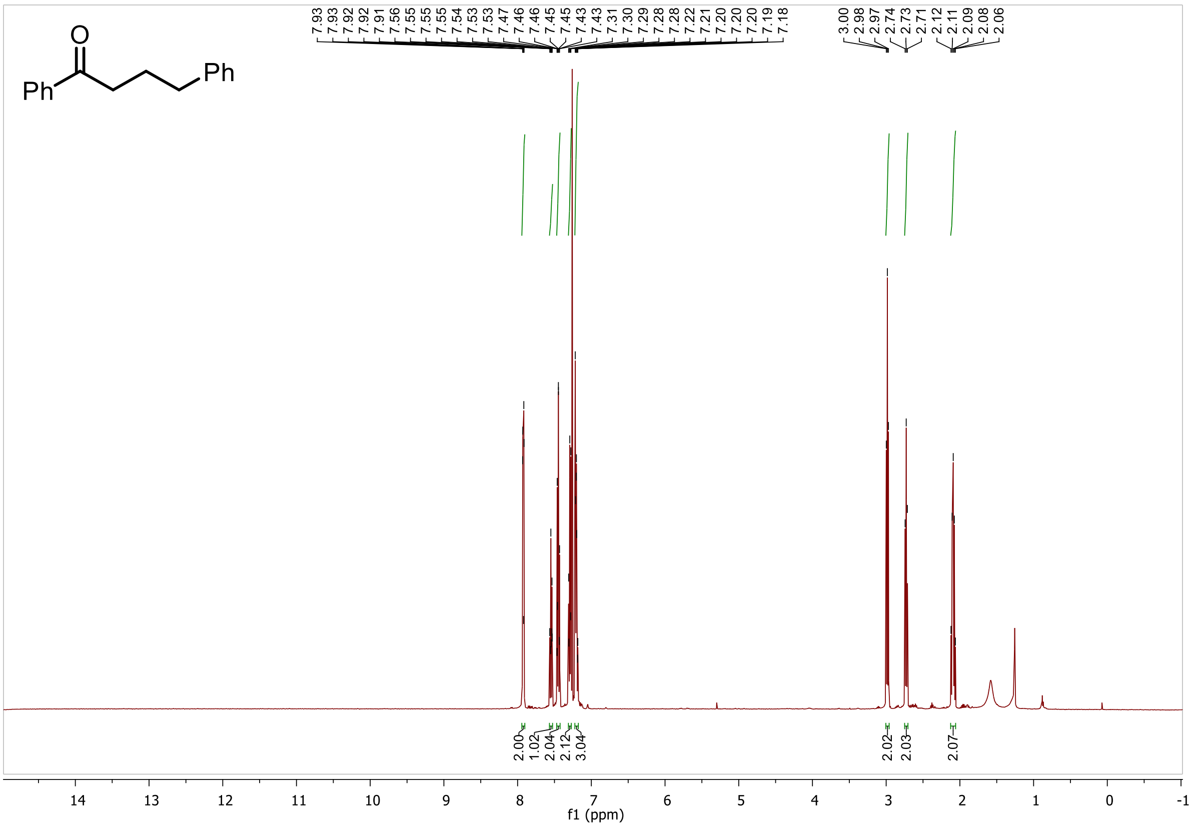

Figure S45. 3c, $^{13}C\ \{^{1}H\}$ NMR, 126 MHz, $CDCl_3$

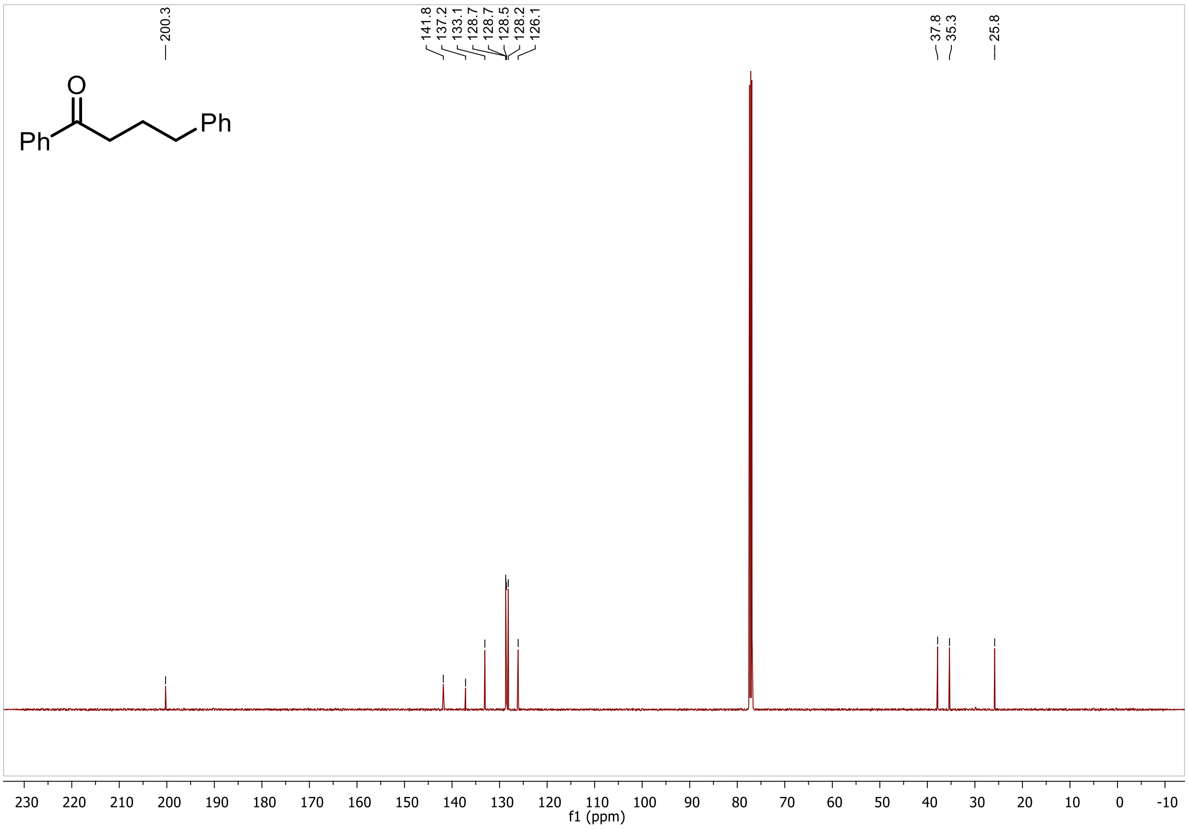

Figure S46. 3a, ^{1}H NMR, 500 MHz, $CDCl_3$

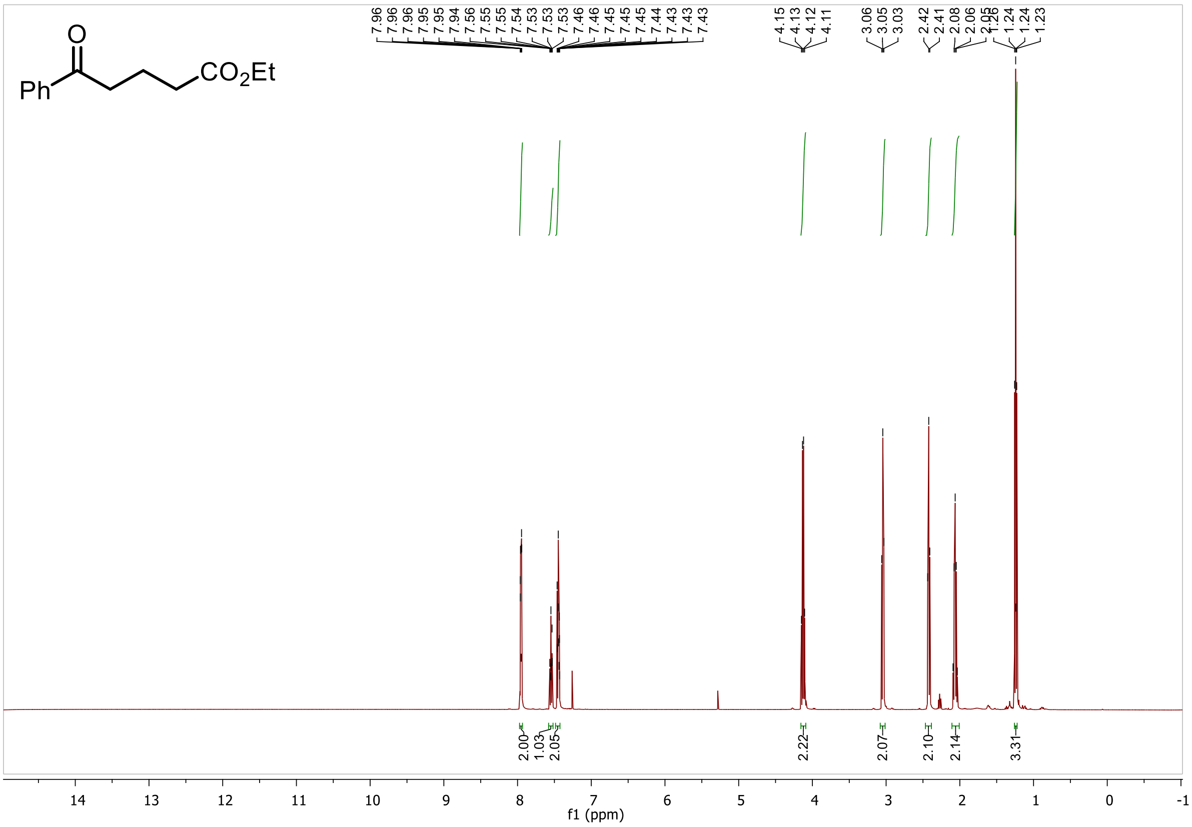

Figure S47. 3a, $^{13}C\{^{1}H\}$ NMR, 126 MHz, $CDCl_3$

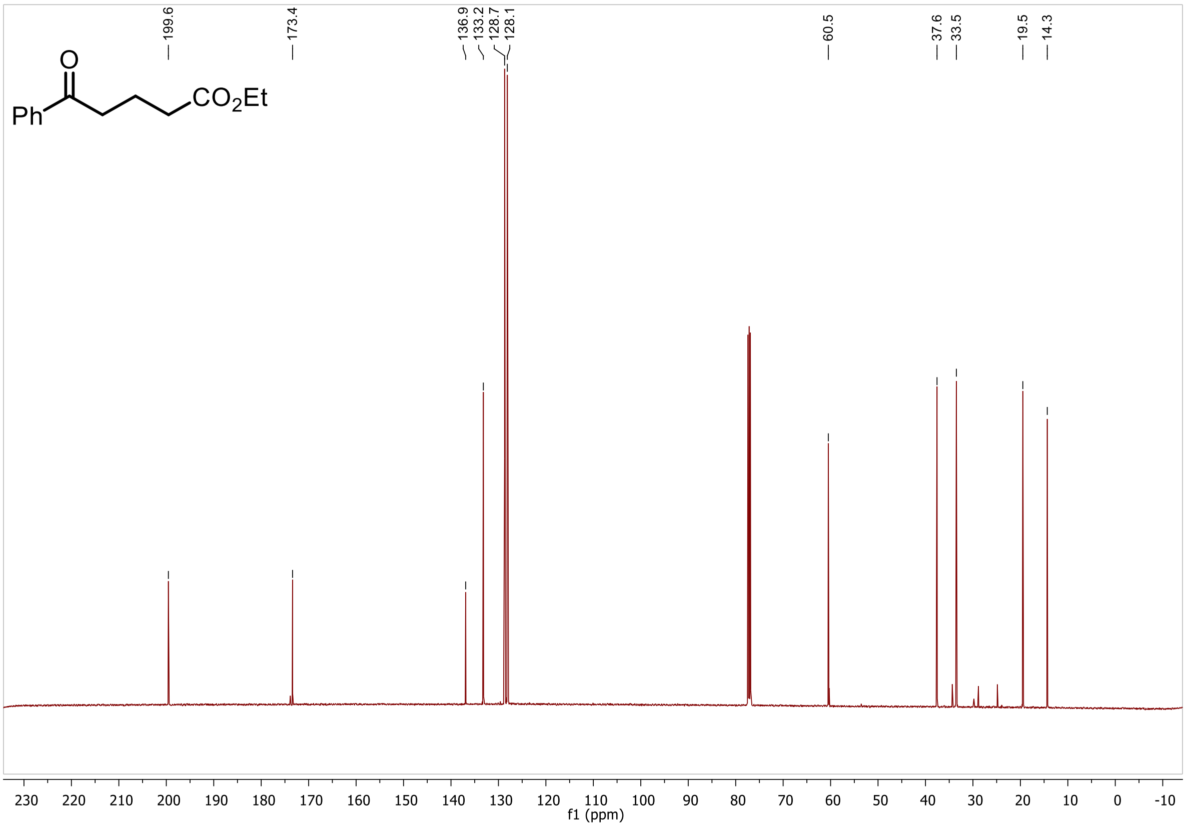

Figure S48. 3f, ^{1}H NMR, 500 MHz, $CDCl_3$

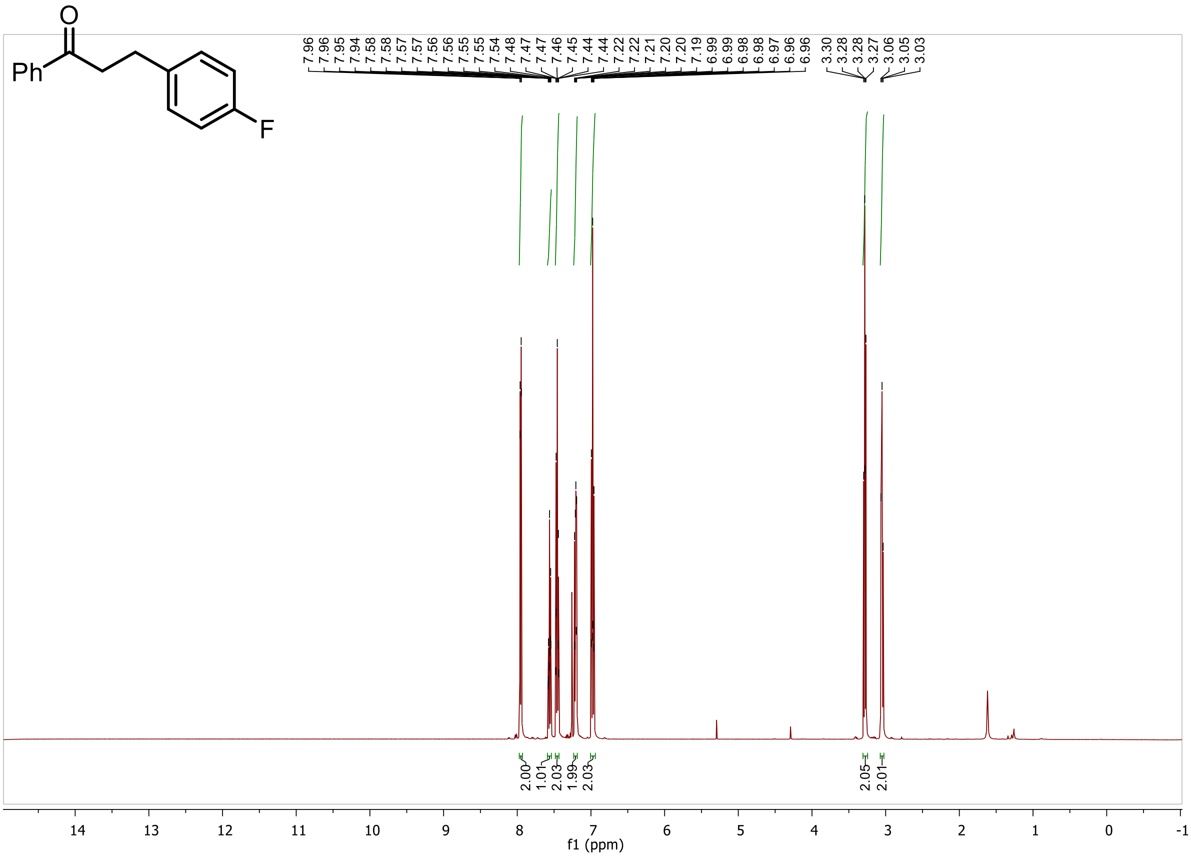

Figure S49. 3f, $^{13}C\{^{1}H\}$ NMR, 126 MHz, $CDCl_3$

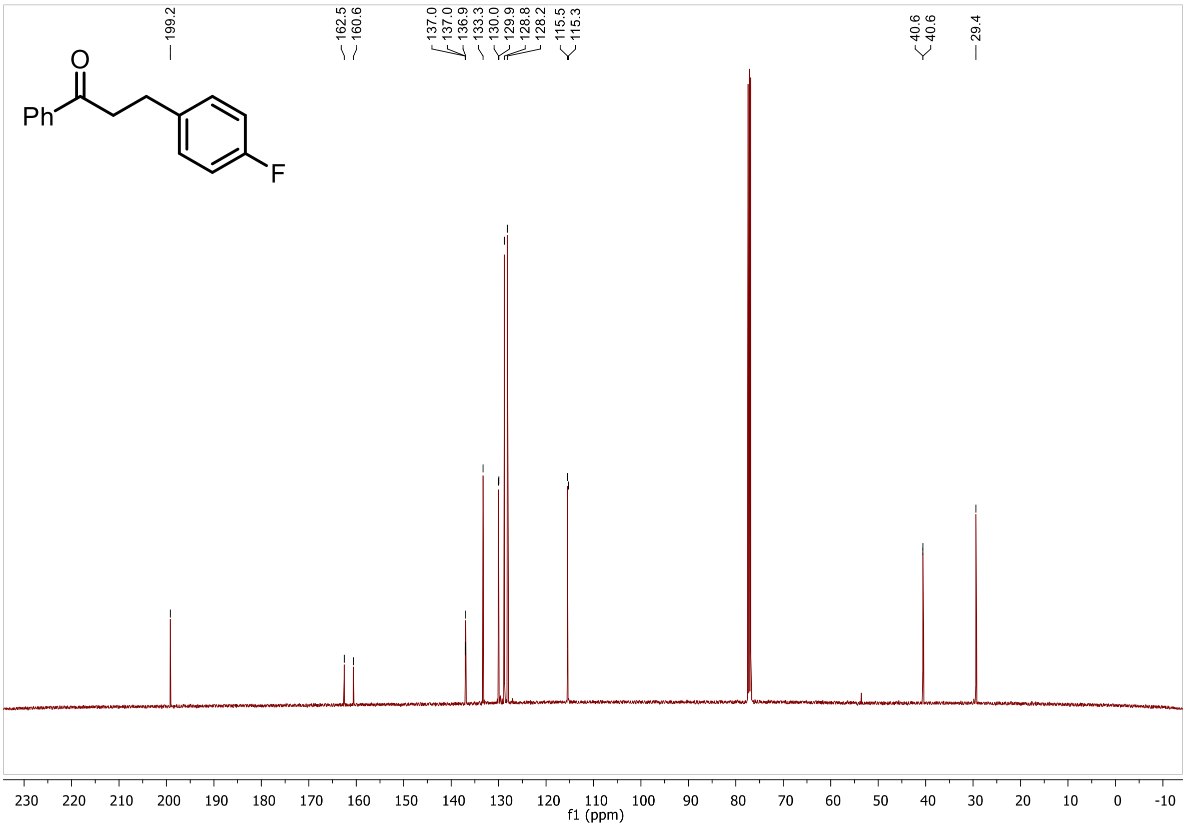

Figure S50. 3f, ^{19}F NMR, 471 MHz, $CDCl_3$

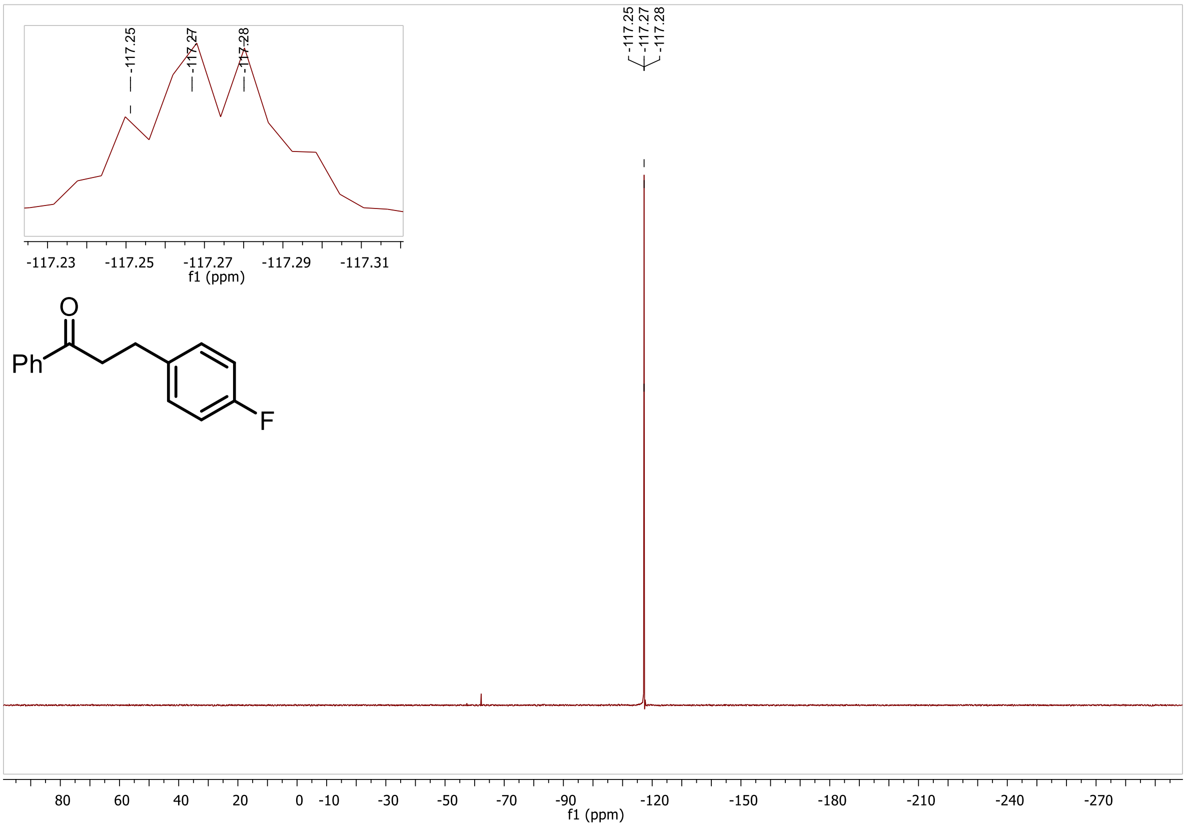

Figure S51. 3d, ^{1}H NMR, 500 MHz, $CDCl_3$

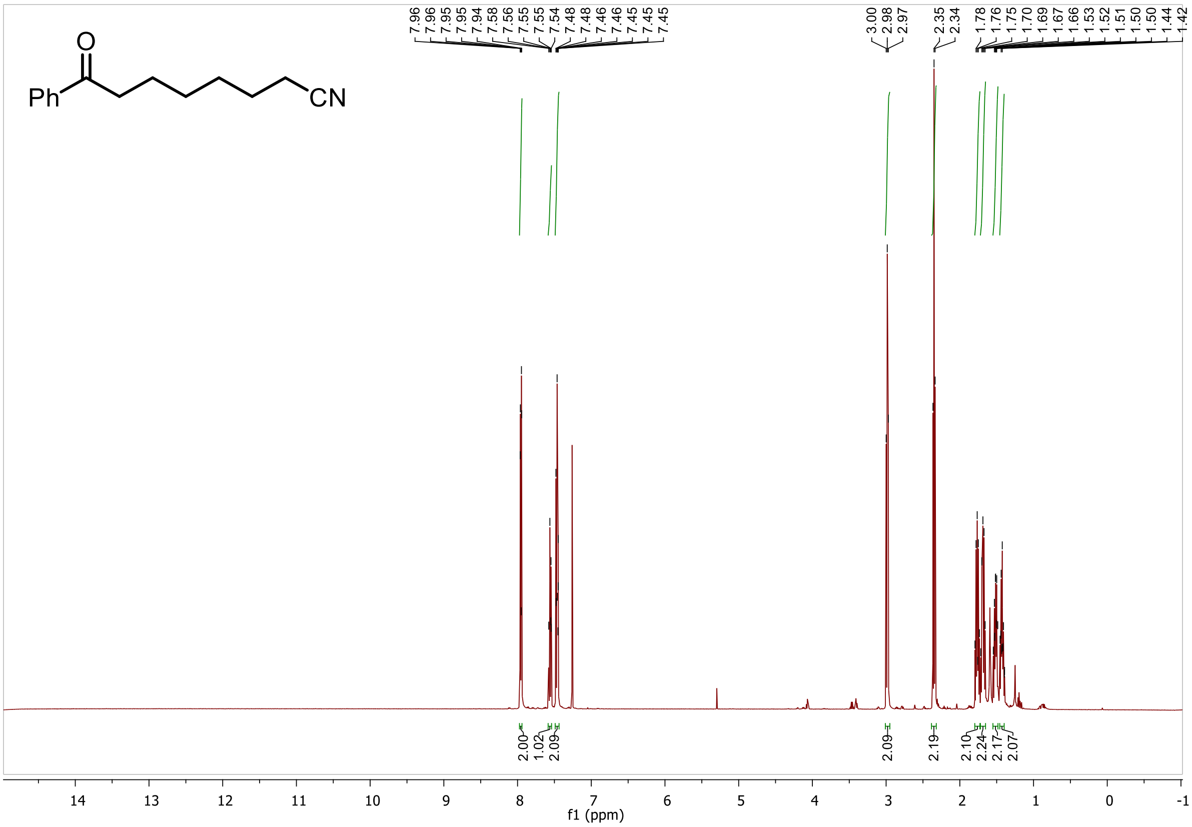

Figure S52. 3d, ^{13}C {^{1}H} NMR, 126 MHz, $CDCl_3$

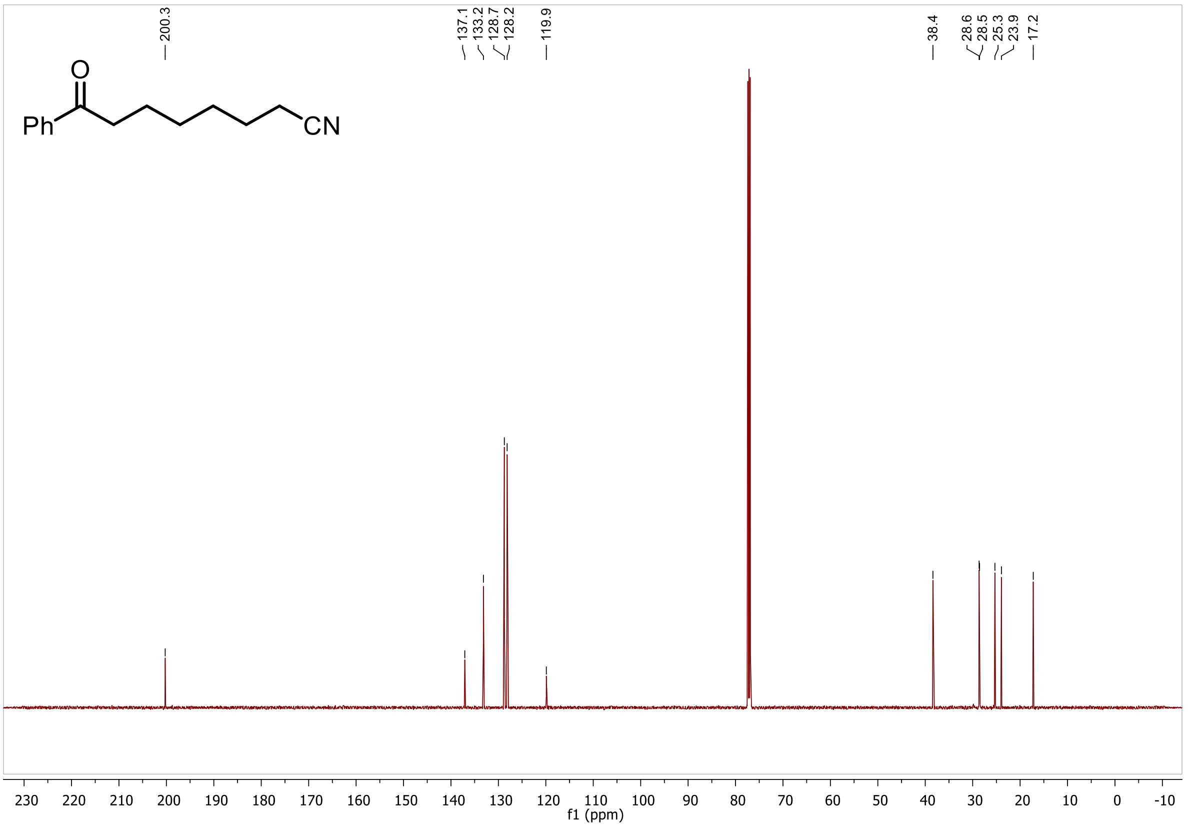

Figure S53. 3e, ^{1}H NMR, 500 MHz, $CDCl_3$

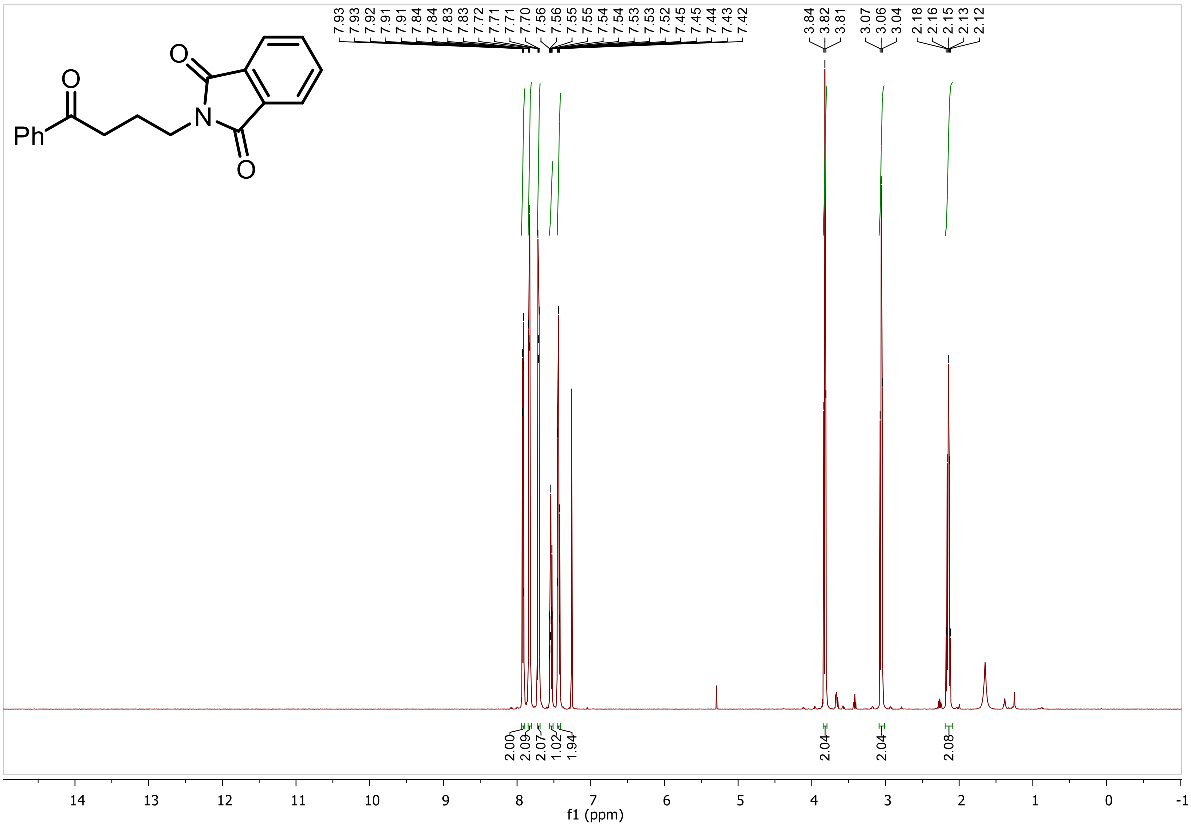

Figure S54. 3e, ^{13}C {^{1}H} NMR, 126 MHz, $CDCl_3$

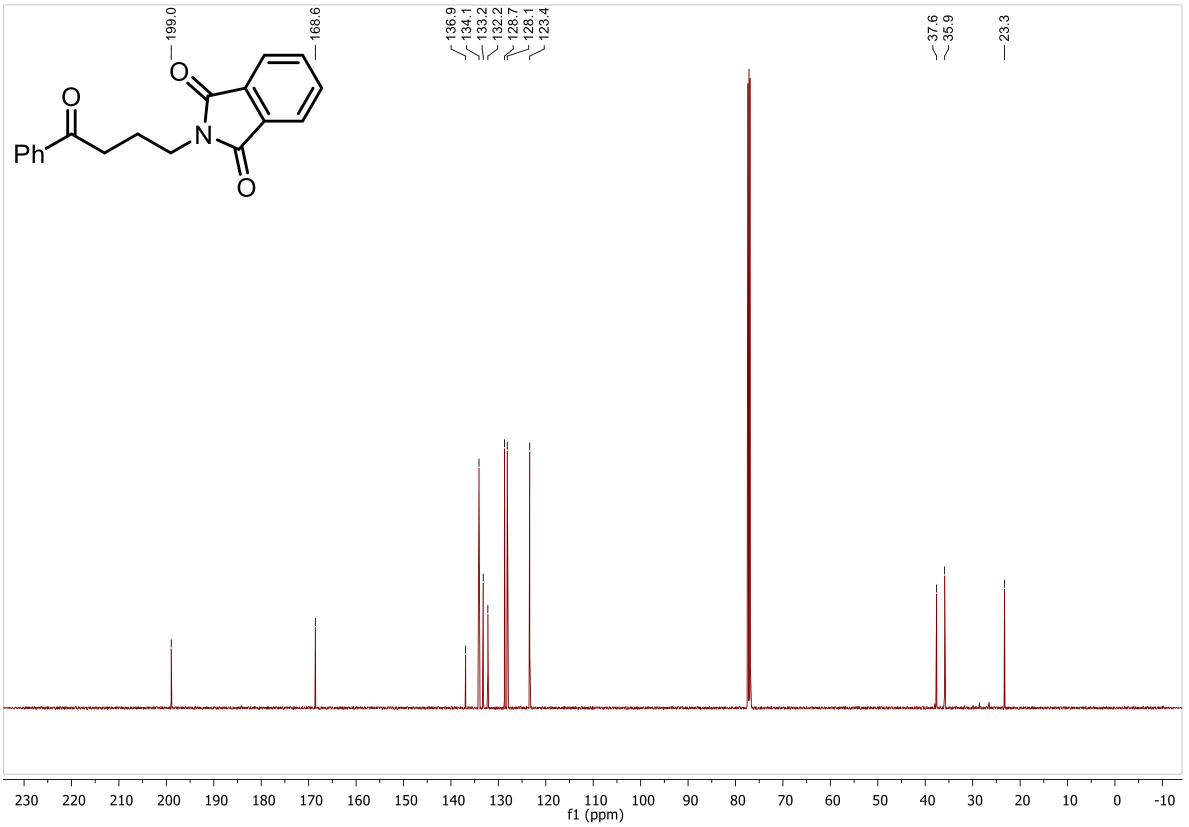

Figure S55. 3g, ^{1}H NMR, 500 MHz, $CDCl_3$

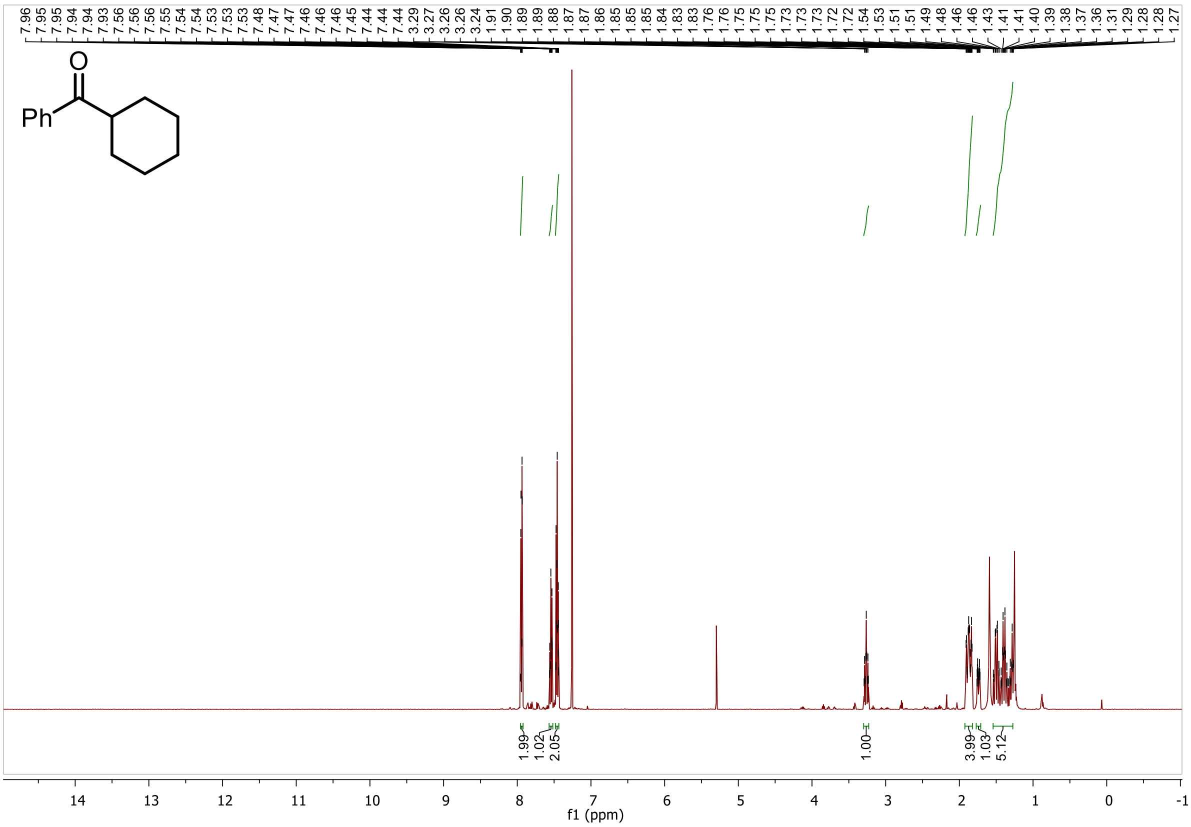

Figure S56. 3g, ^{13}C {^{1}H} NMR, 126 MHz, $CDCl_3$

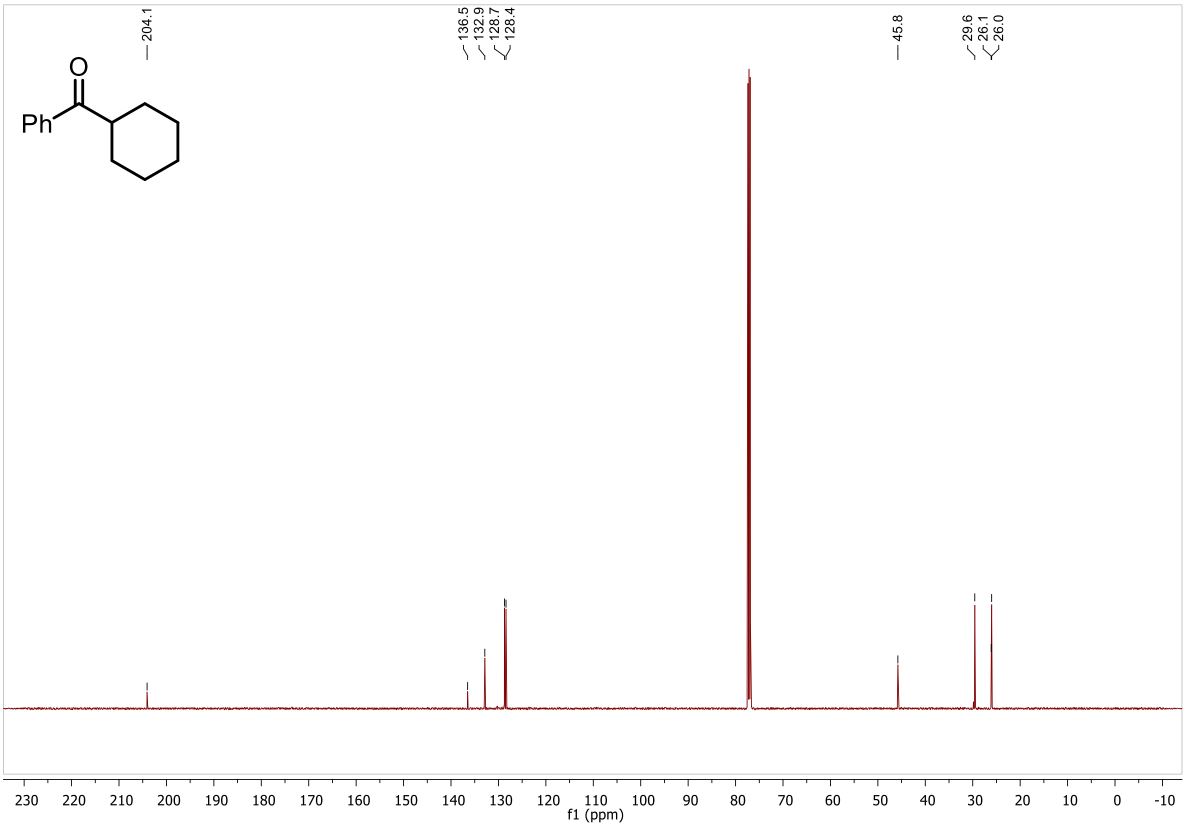

Figure S57. 3h, ^{1}H NMR, 500 MHz, $CDCl_3$

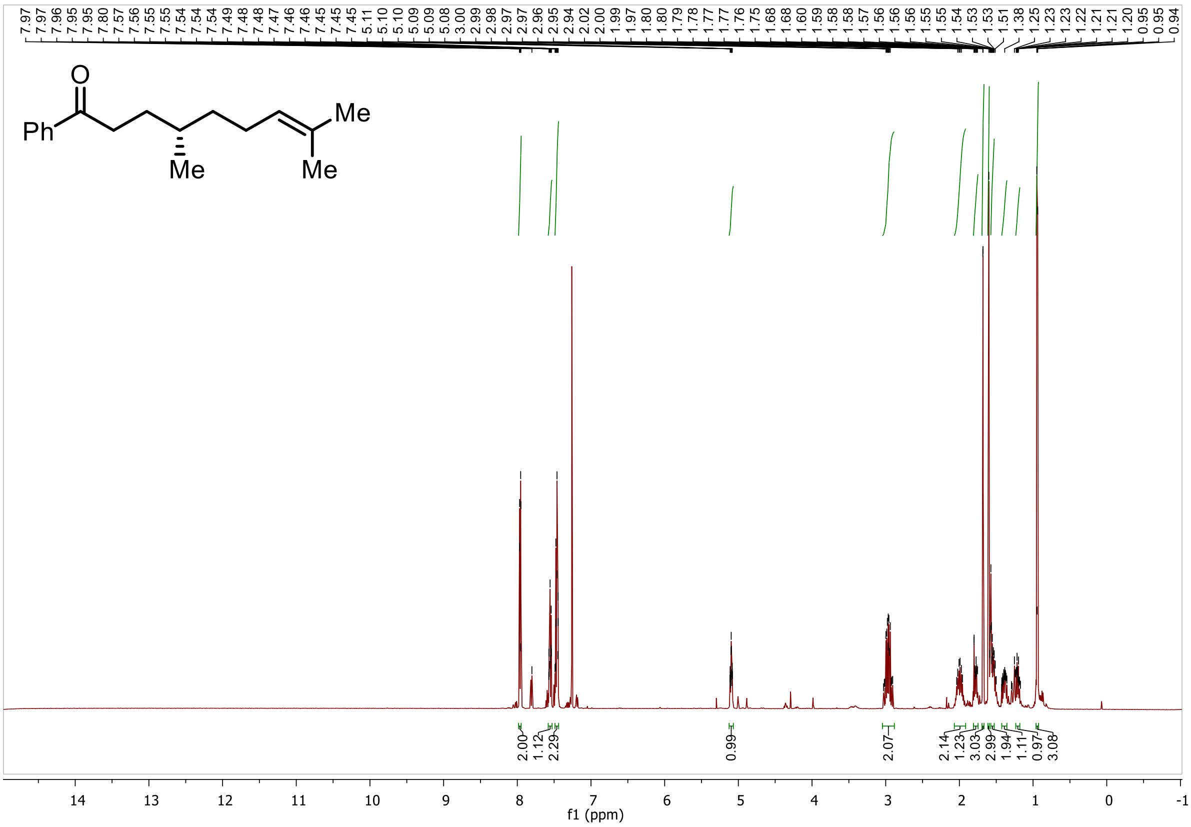

Figure S58. 3h, ^{13}C {^{1}H} NMR, 126 MHz, $CDCl_3$

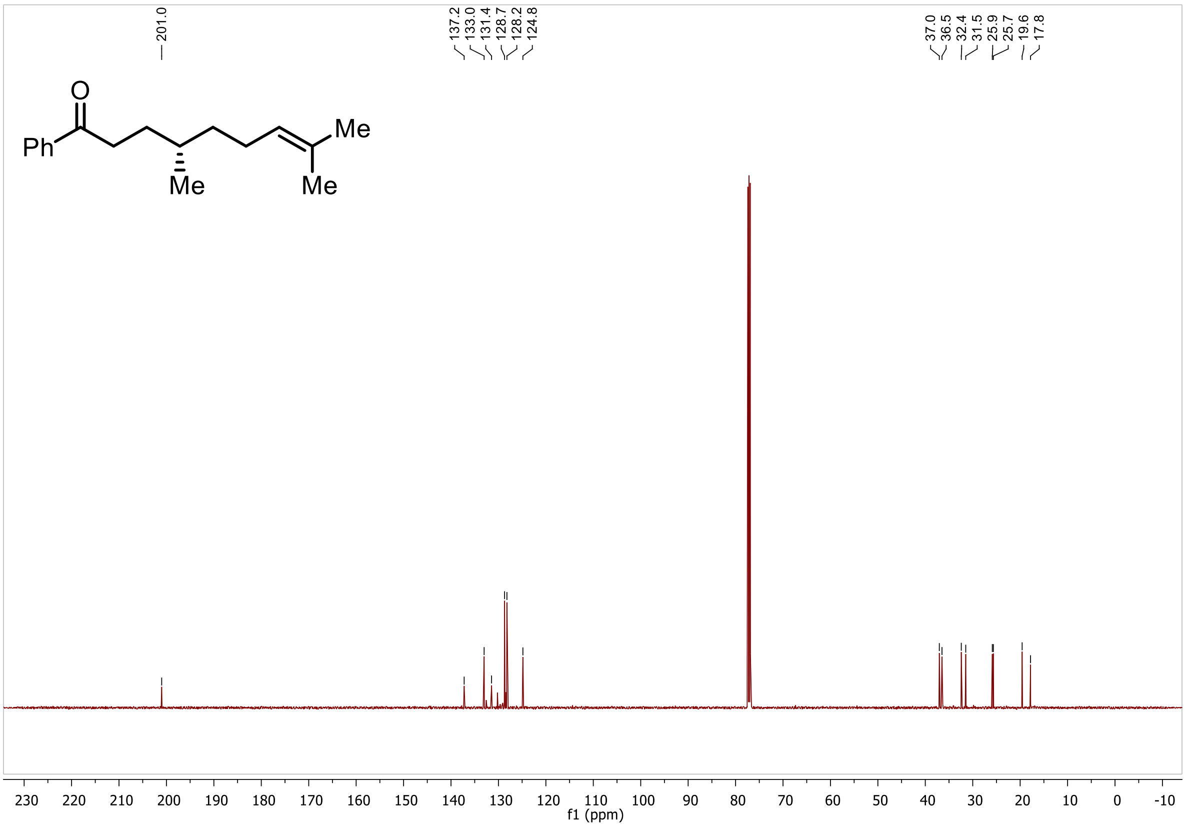

Figure S59. 3i, ^{1}H NMR, 500 MHz, $CDCl_3$

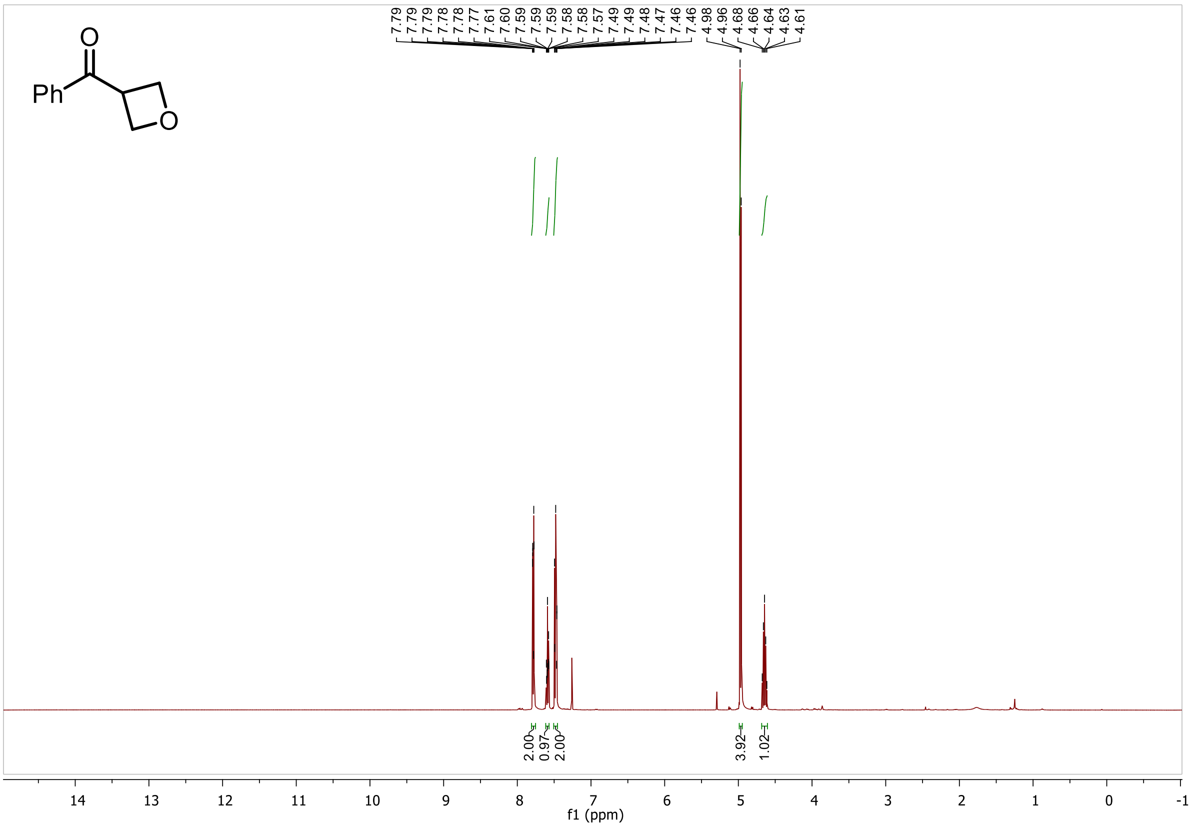

Figure S60. 3i, ^{13}C {^{1}H} NMR, 126 MHz, $CDCl_3$

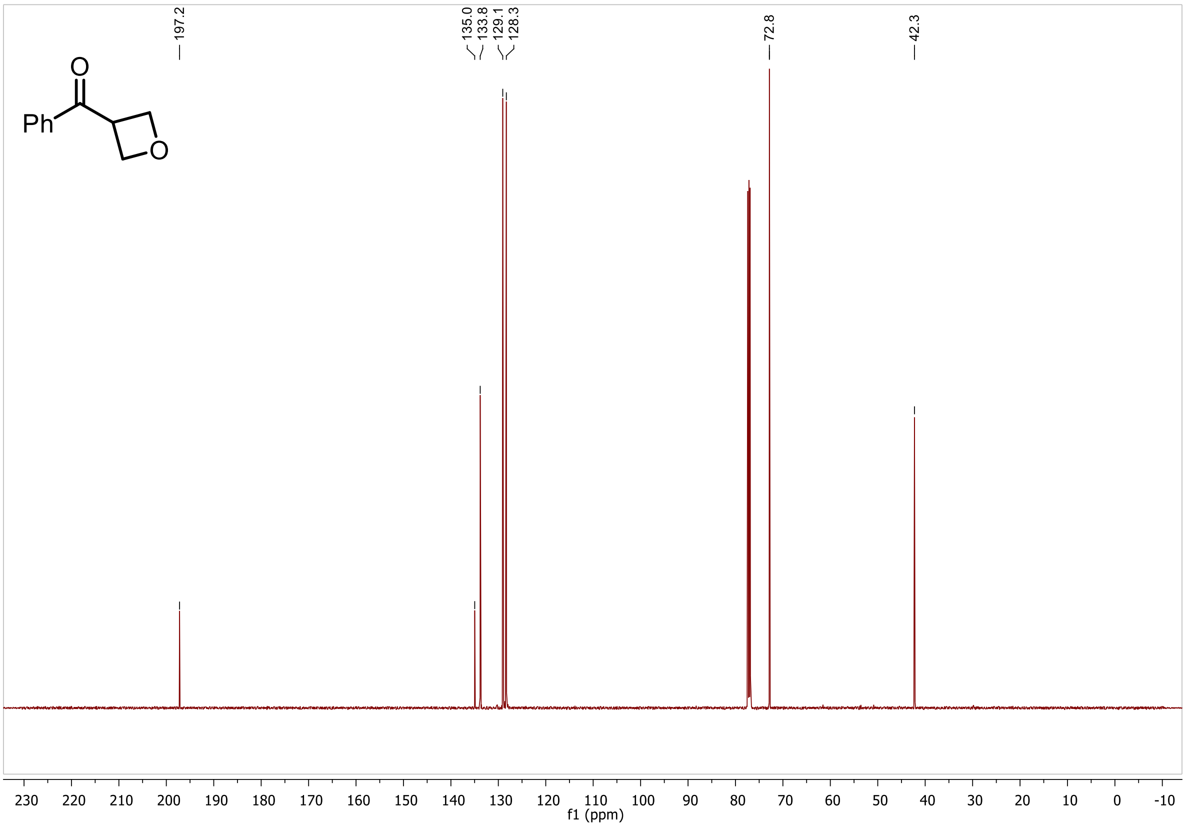

Figure S61. 3j, ^{1}H NMR, 500 MHz, $CDCl_3$

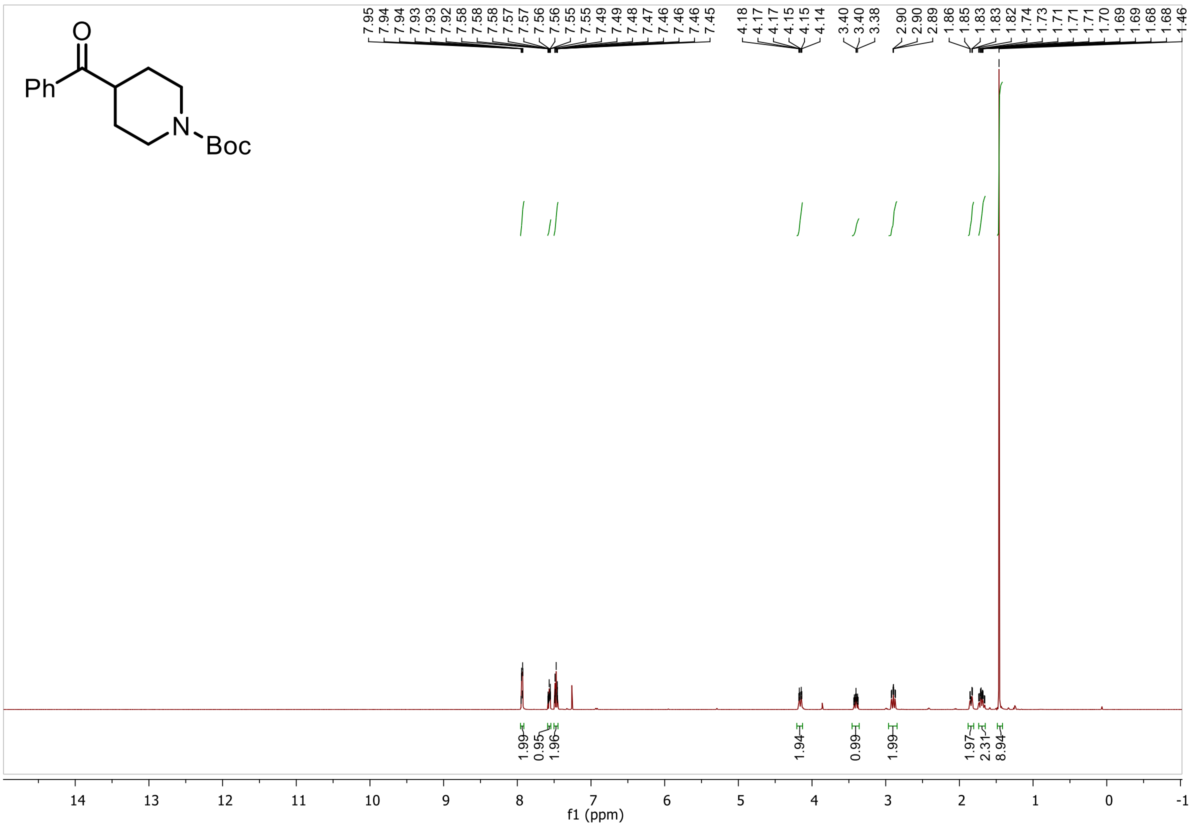

Figure S62. 3j, ^{13}C {^{1}H} NMR, 126 MHz, $CDCl_3$

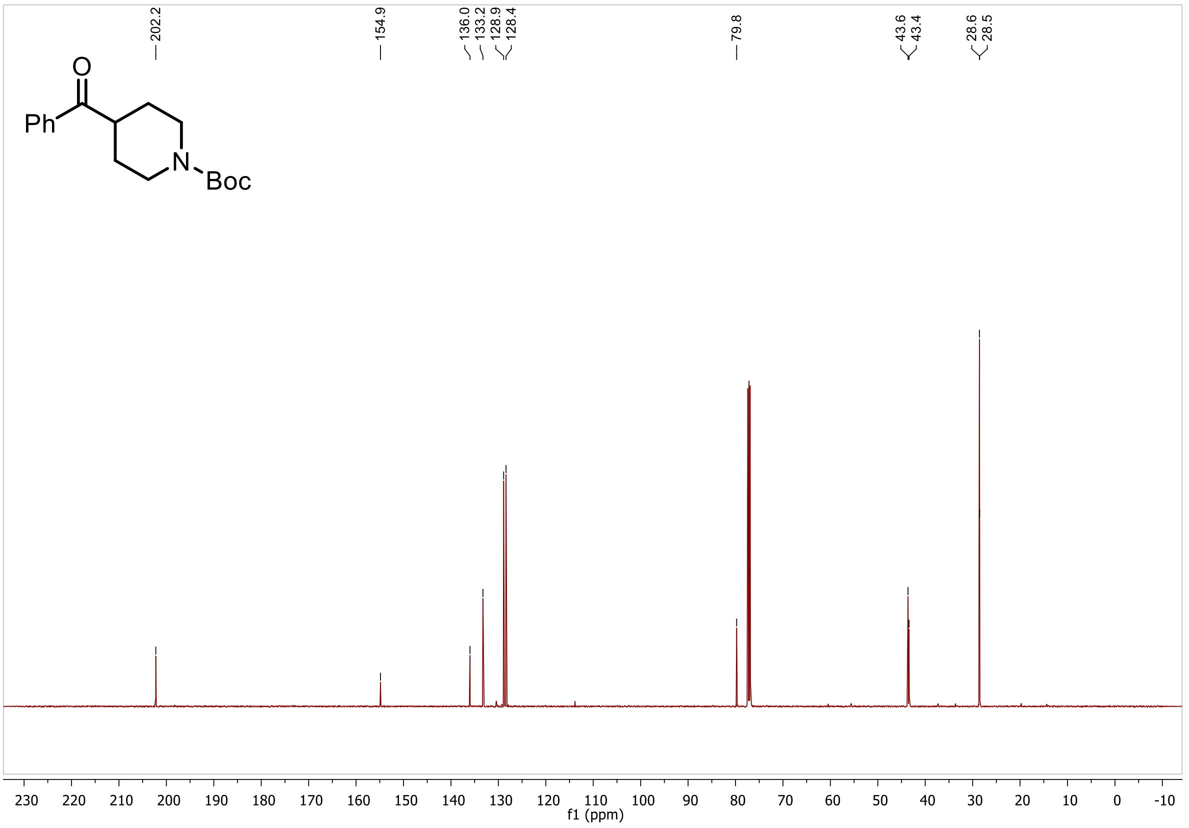

Figure S63. 3l, ^{1}H NMR, 500 MHz, $CDCl_3$

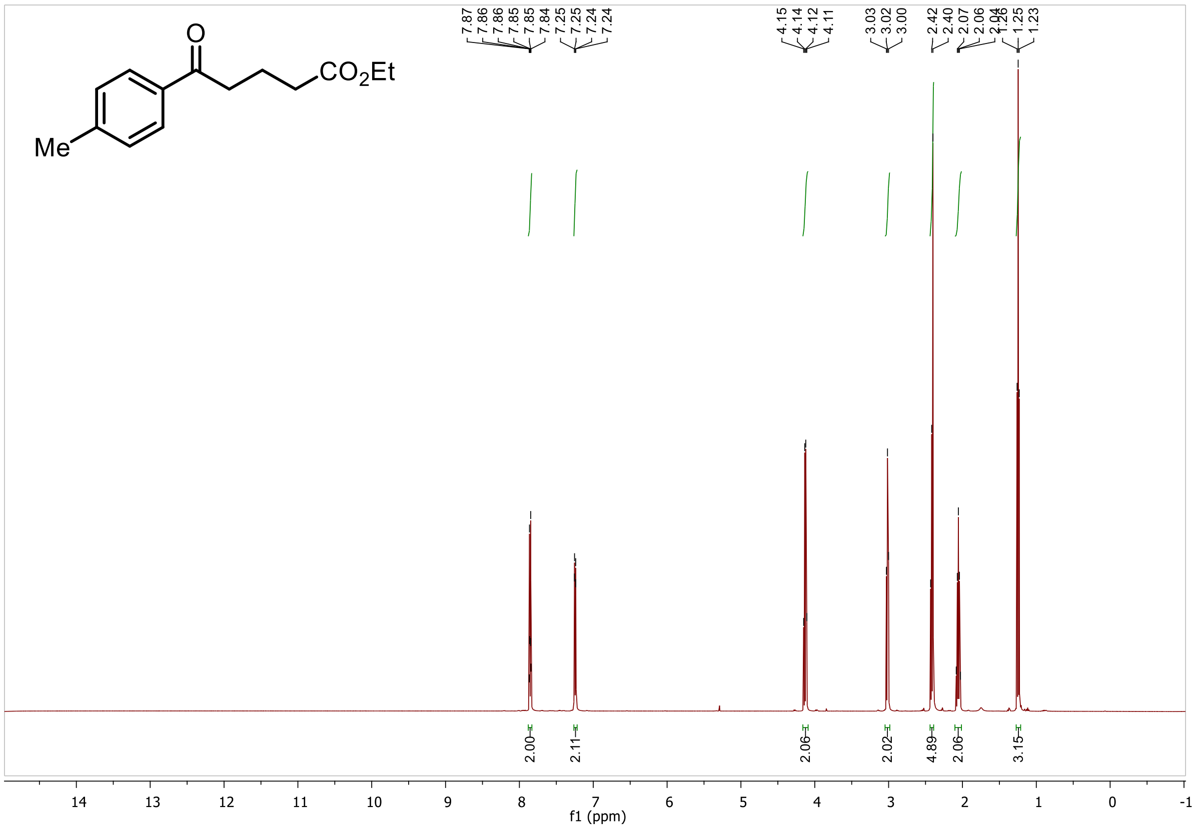

Figure S64. 3l, ^{13}C {^{1}H} NMR, 126 MHz, $CDCl_3$

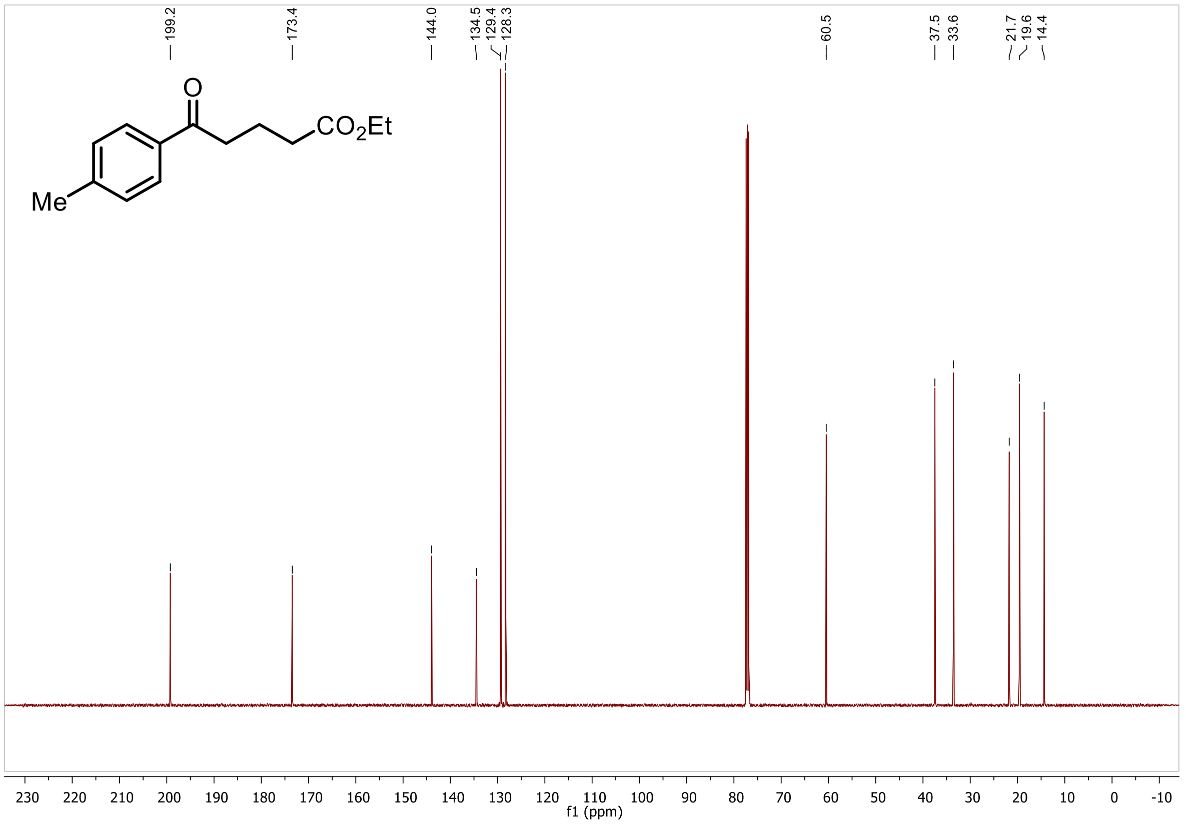

Figure S65. 3m, ^{1}H NMR, 500 MHz, $CDCl_3$

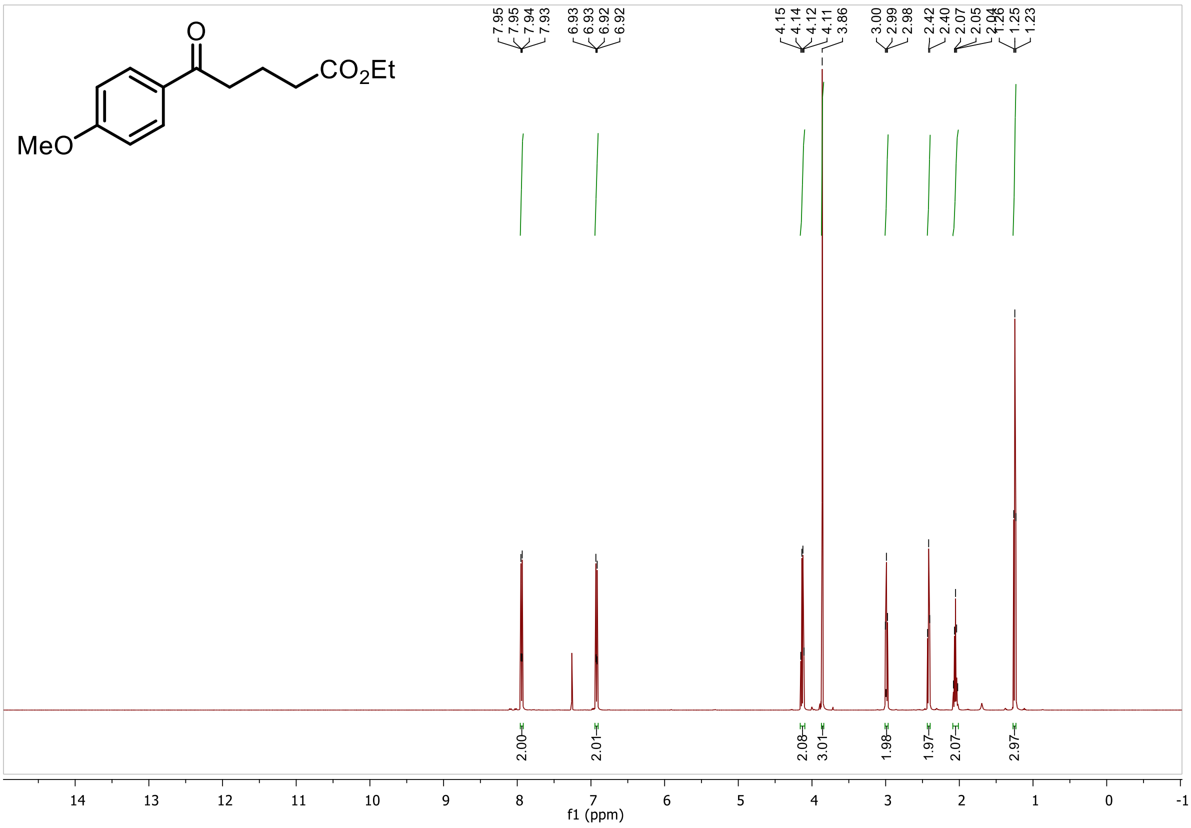

Figure S66. 3m, ^{13}C {^{1}H} NMR, 126 MHz, $CDCl_3$

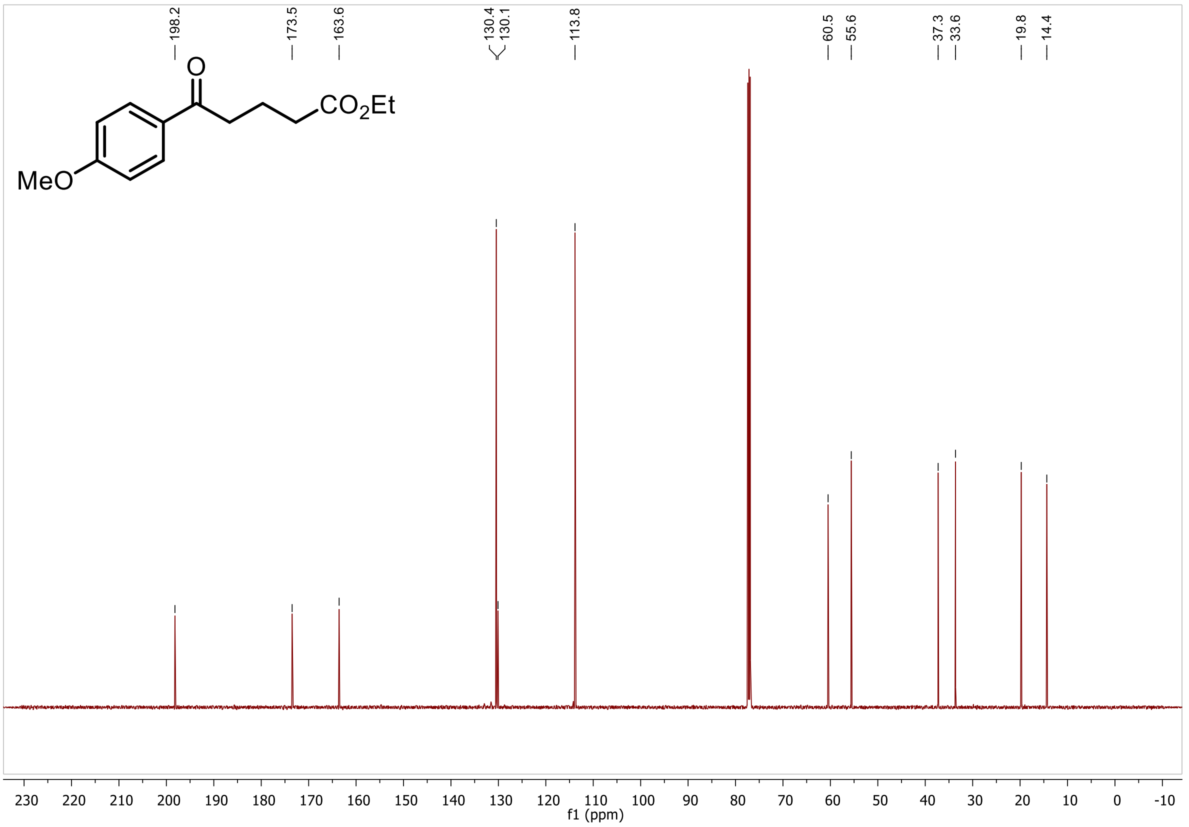

Figure S67. 3n, ^{1}H NMR, 500 MHz, $CDCl_3$

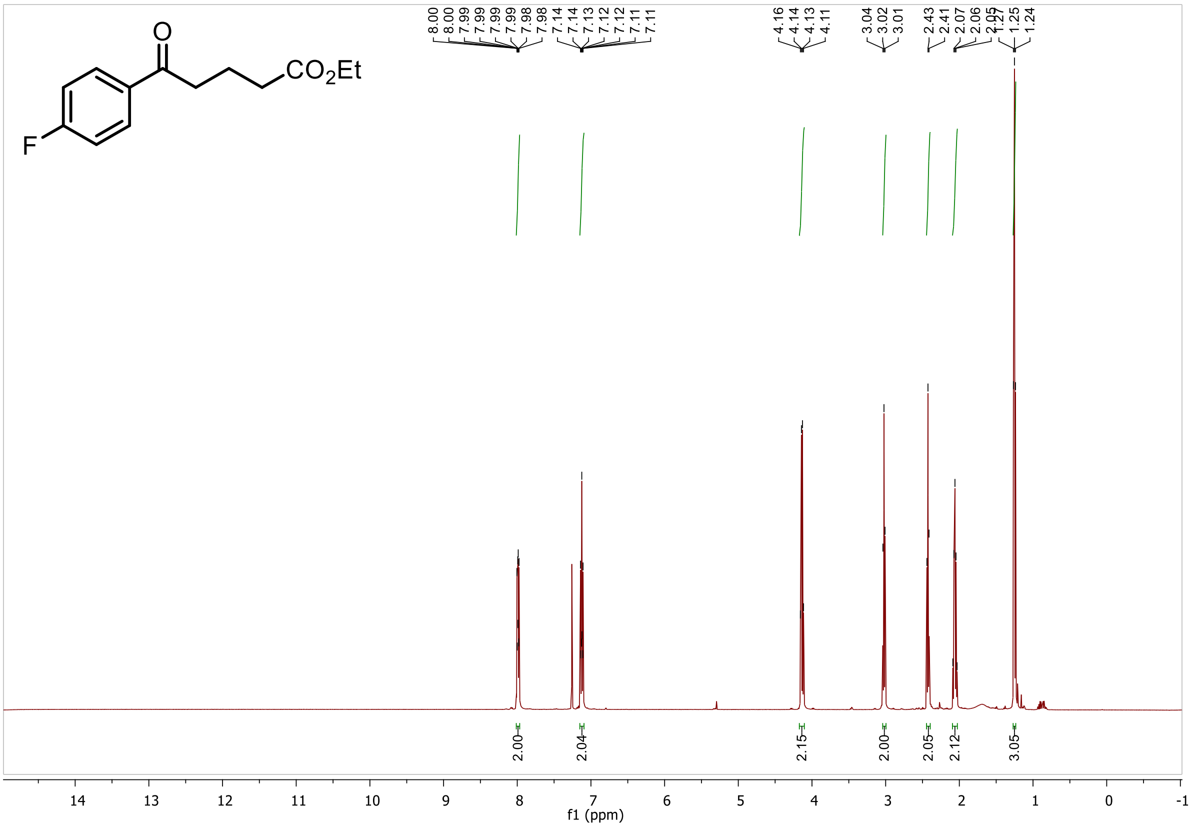

Figure S68. 3n, ^{13}C {^{1}H} NMR, 126 MHz, $CDCl_3$

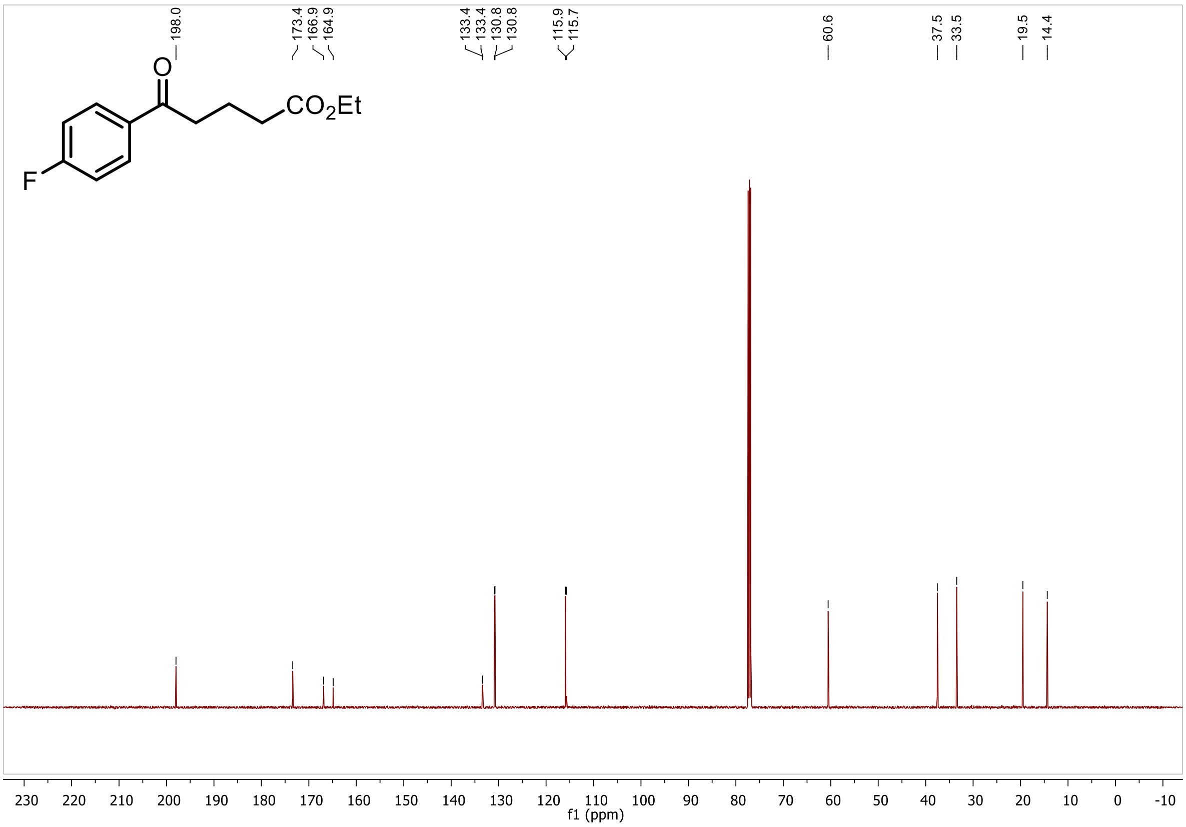

Figure S69. 3n, ^{19}F NMR, 471 MHz, $CDCl_3$

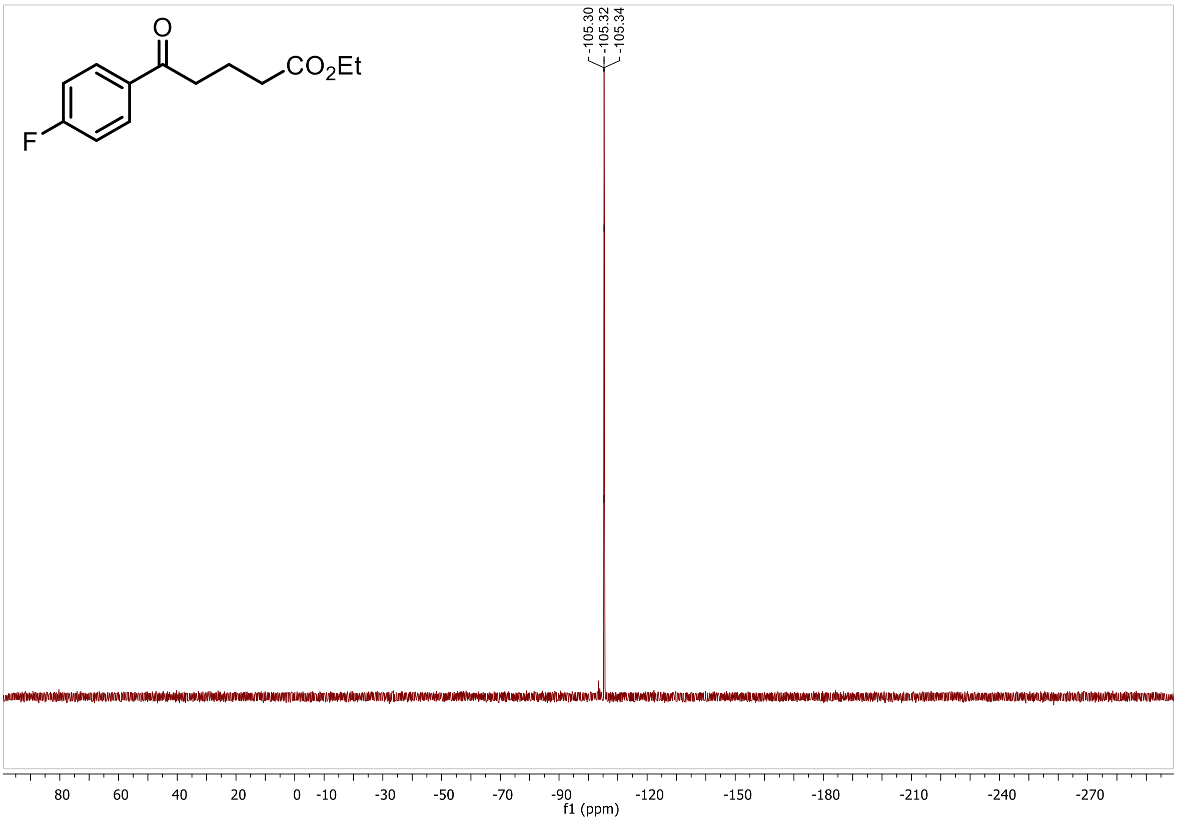

Figure S70. 3o, ^{1}H NMR, 500 MHz, $CDCl_3$

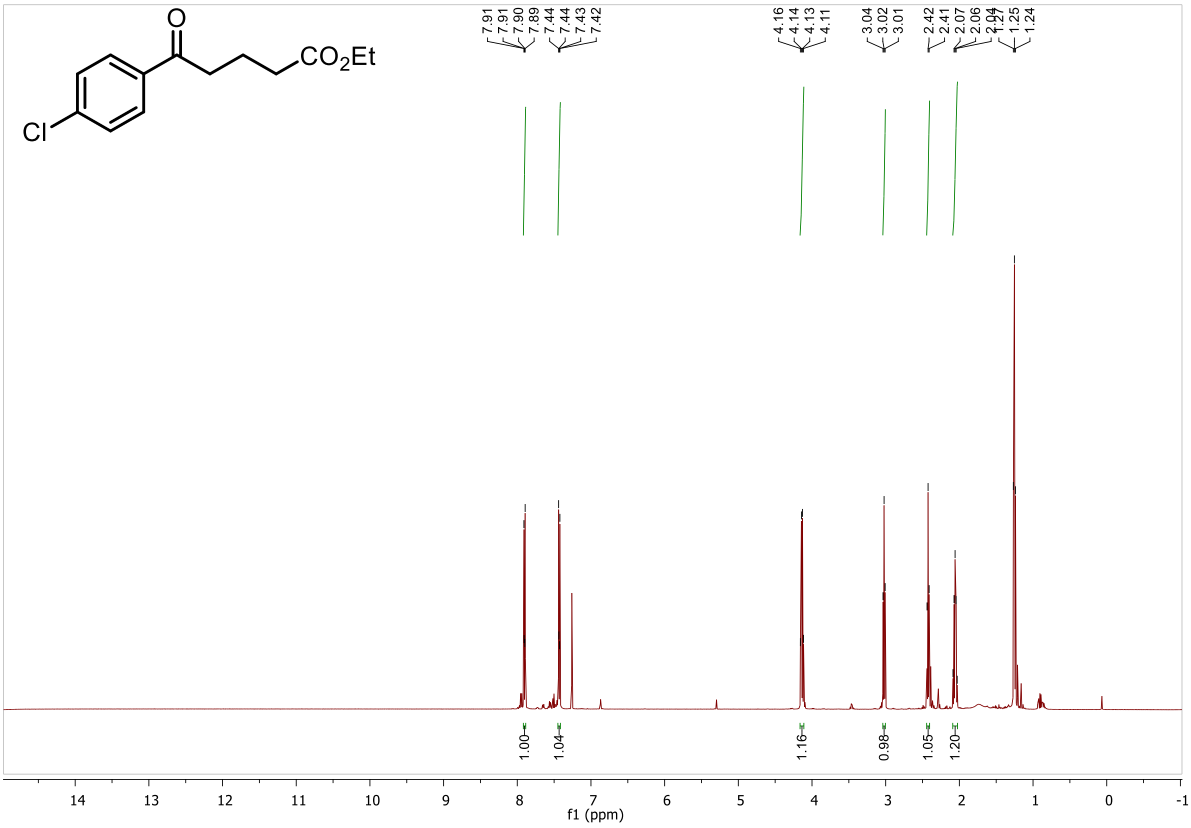

Figure S71. 3o, ^{13}C {^1H} NMR, 126 MHz, $CDCl_3$

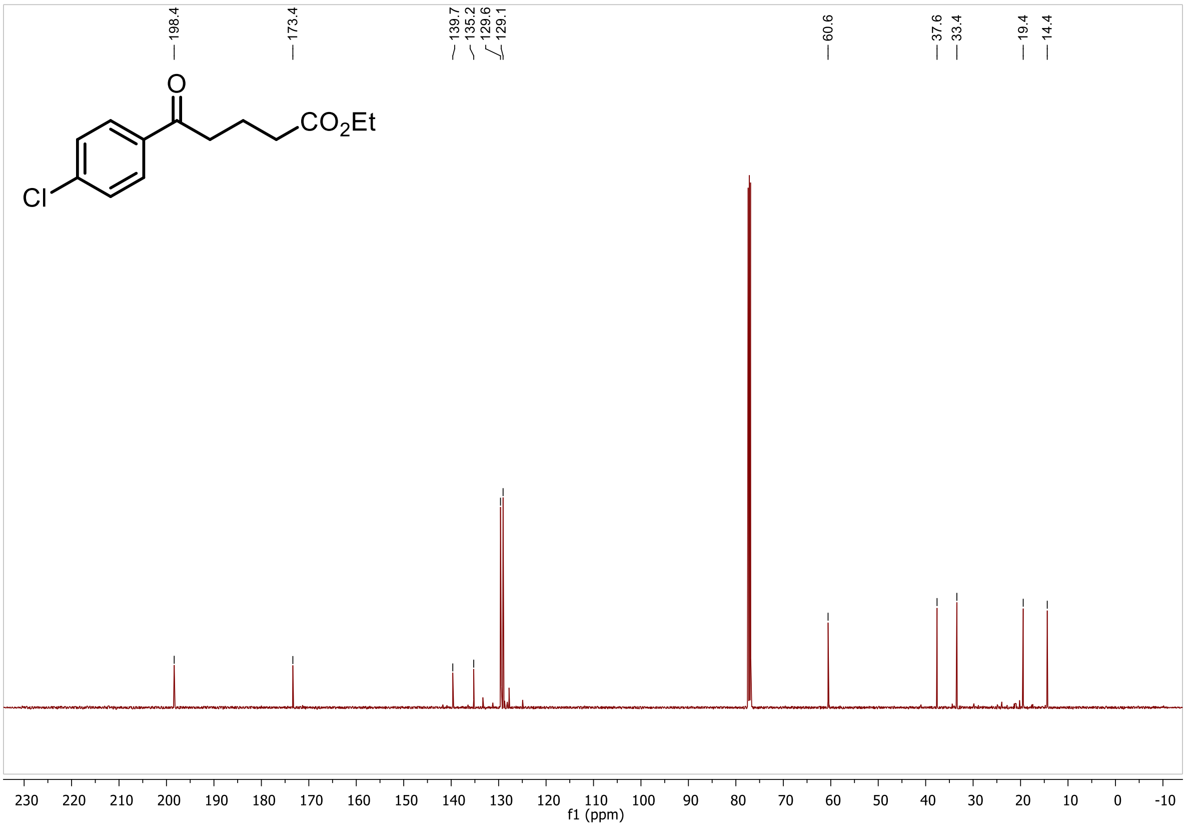

Figure S72. 3q, ^1H NMR, 500 MHz, $CDCl_3$

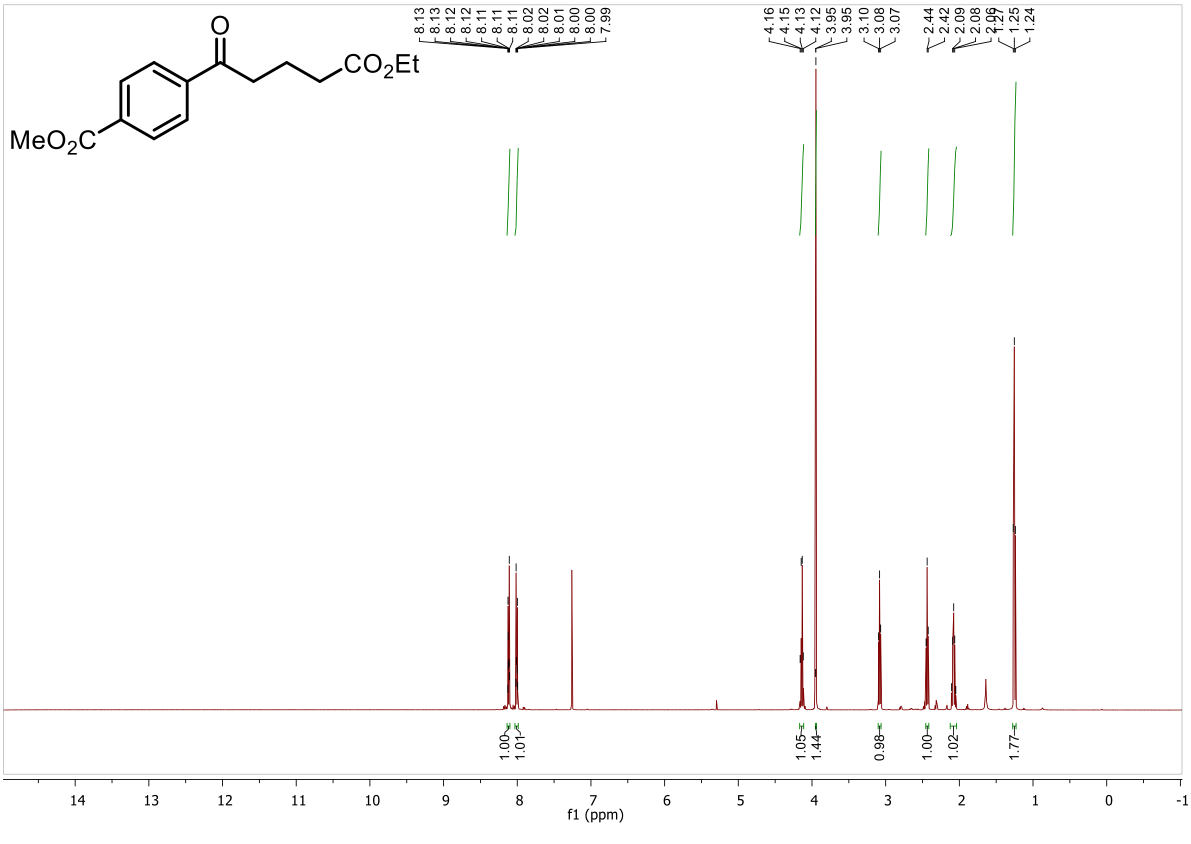

Figure S73. 3q, $^{13}C\ \{^1H\}$ NMR, 126 MHz, $CDCl_3$

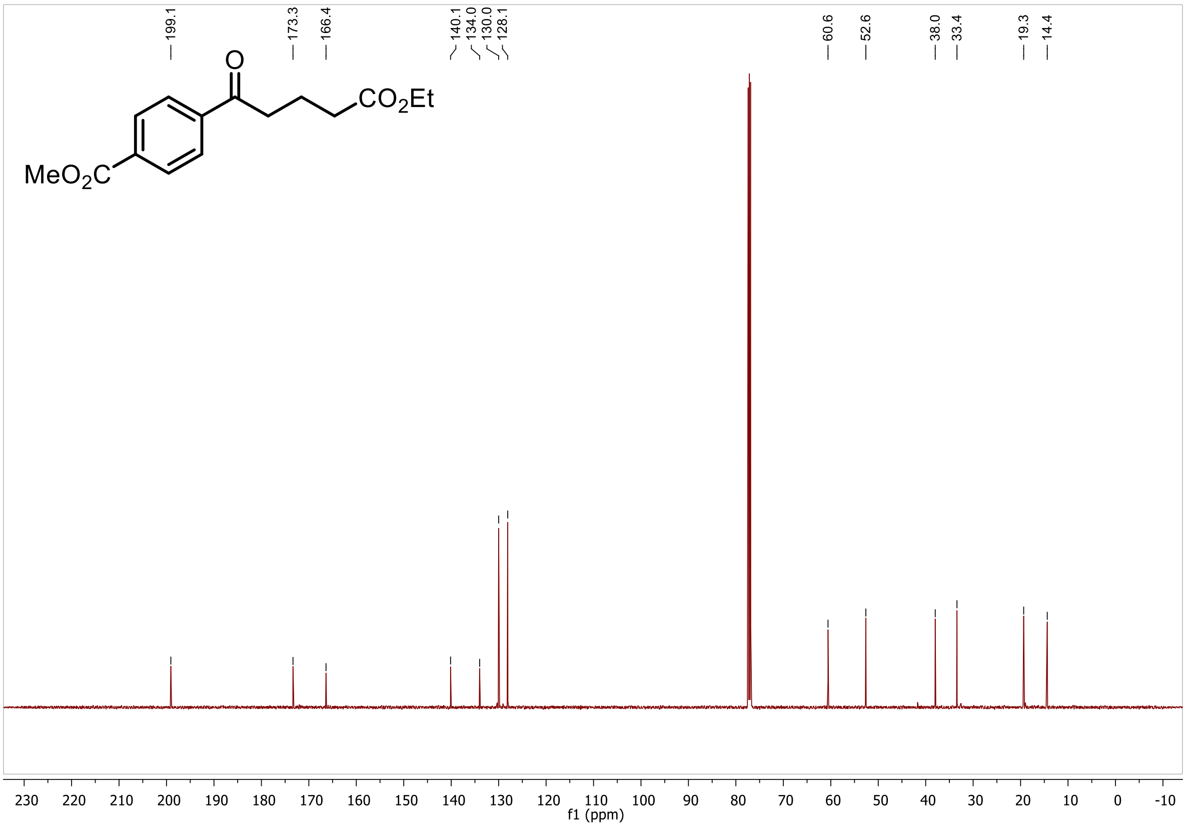

Figure S74. 3p, ^1H NMR, 500 MHz, $CDCl_3$

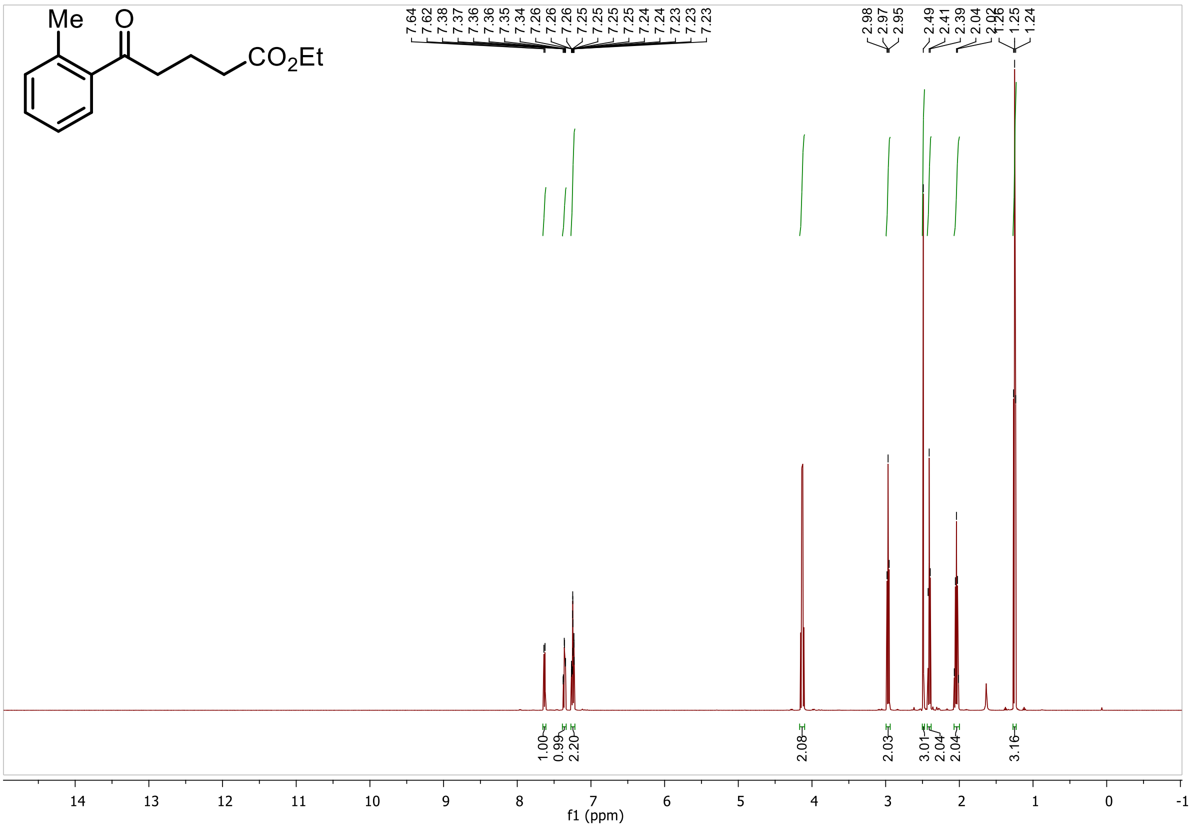

Figure S75. 3p, $^{13}C\{^1H\}$ NMR, 126 MHz, $CDCl_3$

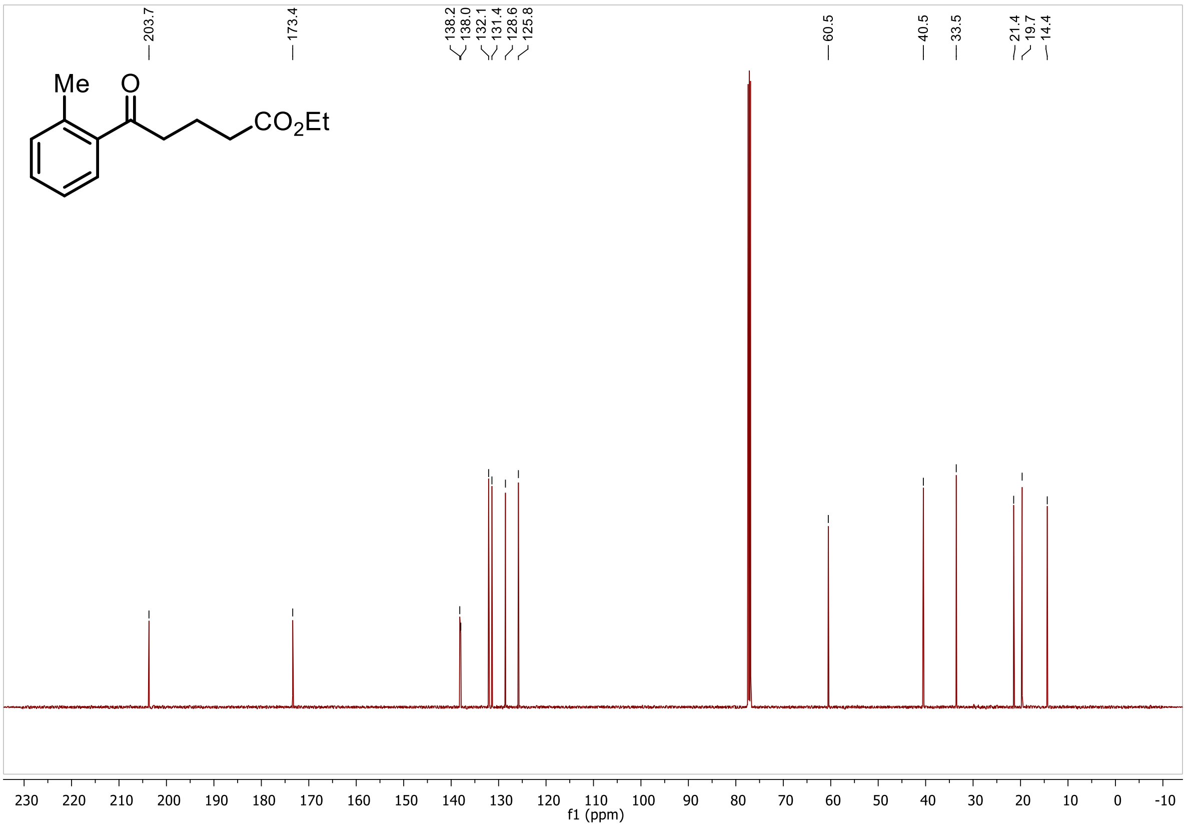

Figure S76. 3r, ^1H NMR, 500 MHz, $CDCl_3$

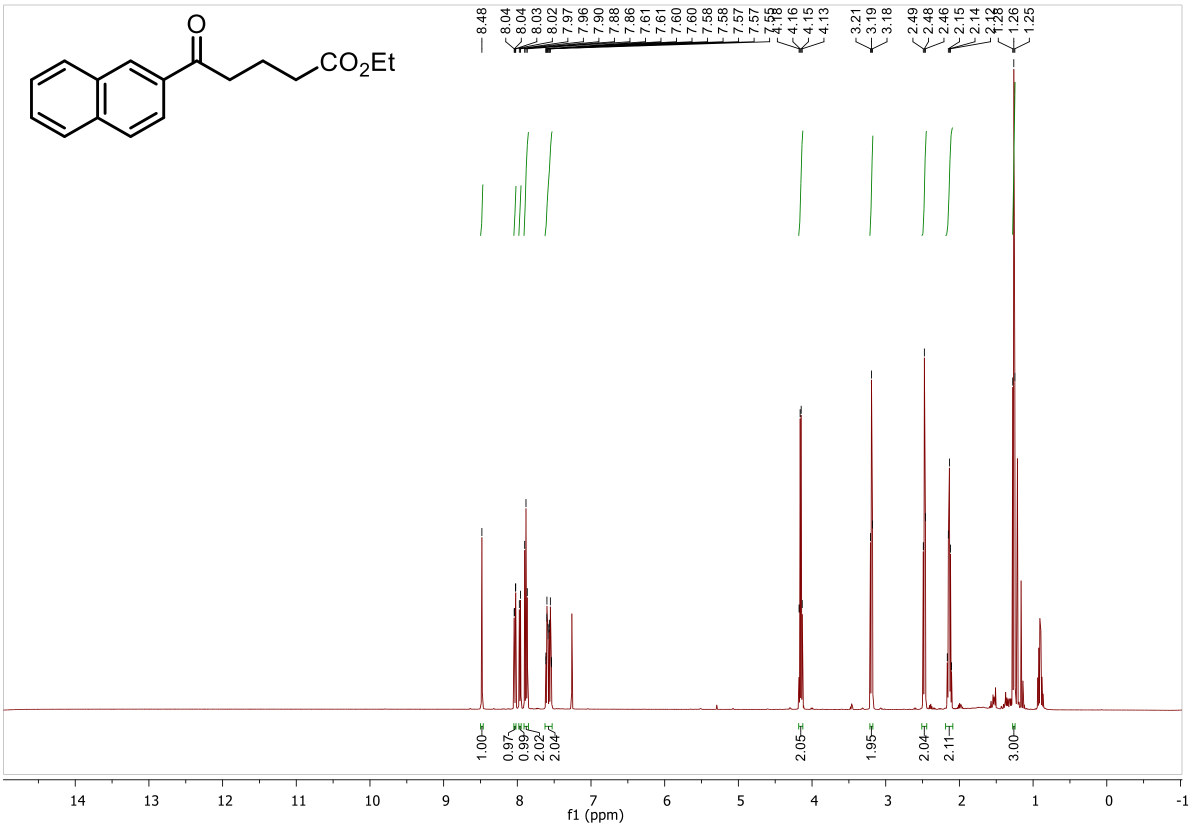

Figure S77. 3r, $^{13}C\ \{^{1}H\}$ NMR, 126 MHz, $CDCl_3$

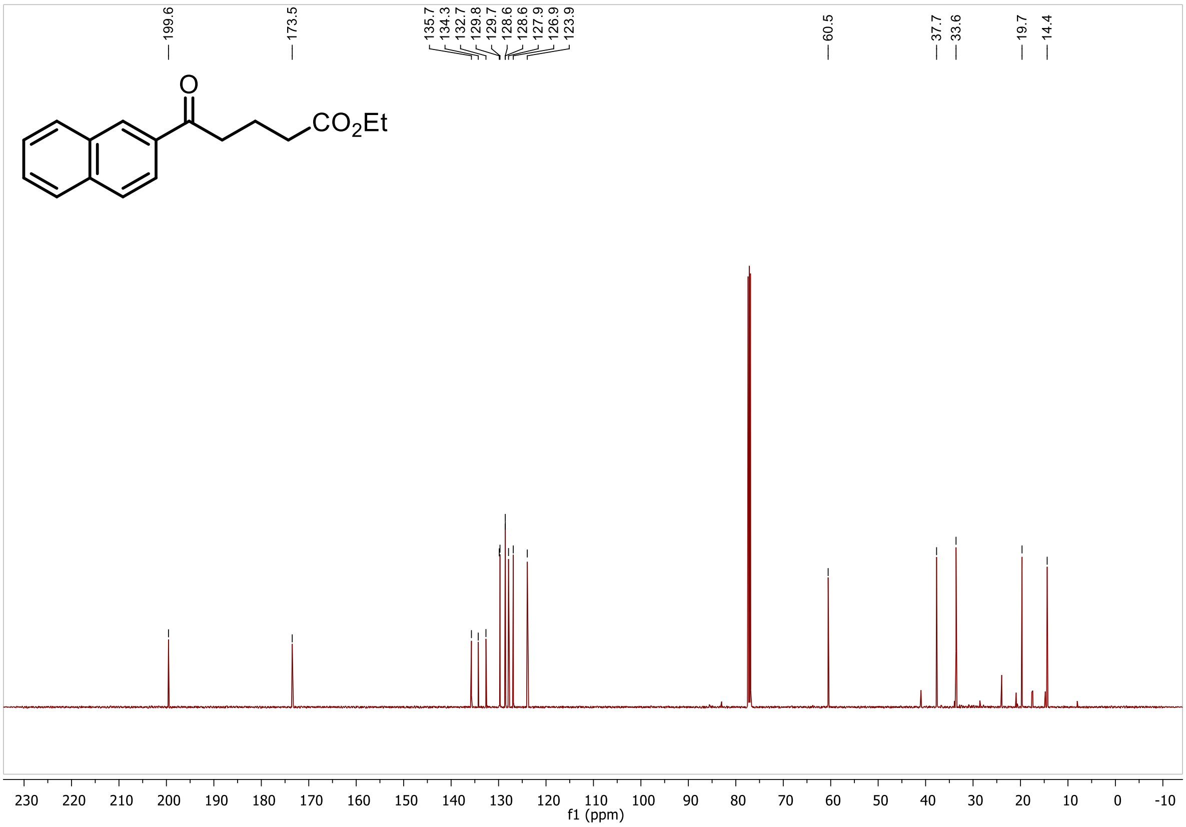

Figure S78. 3s, ^{1}H NMR, 500 MHz, $CDCl_3$

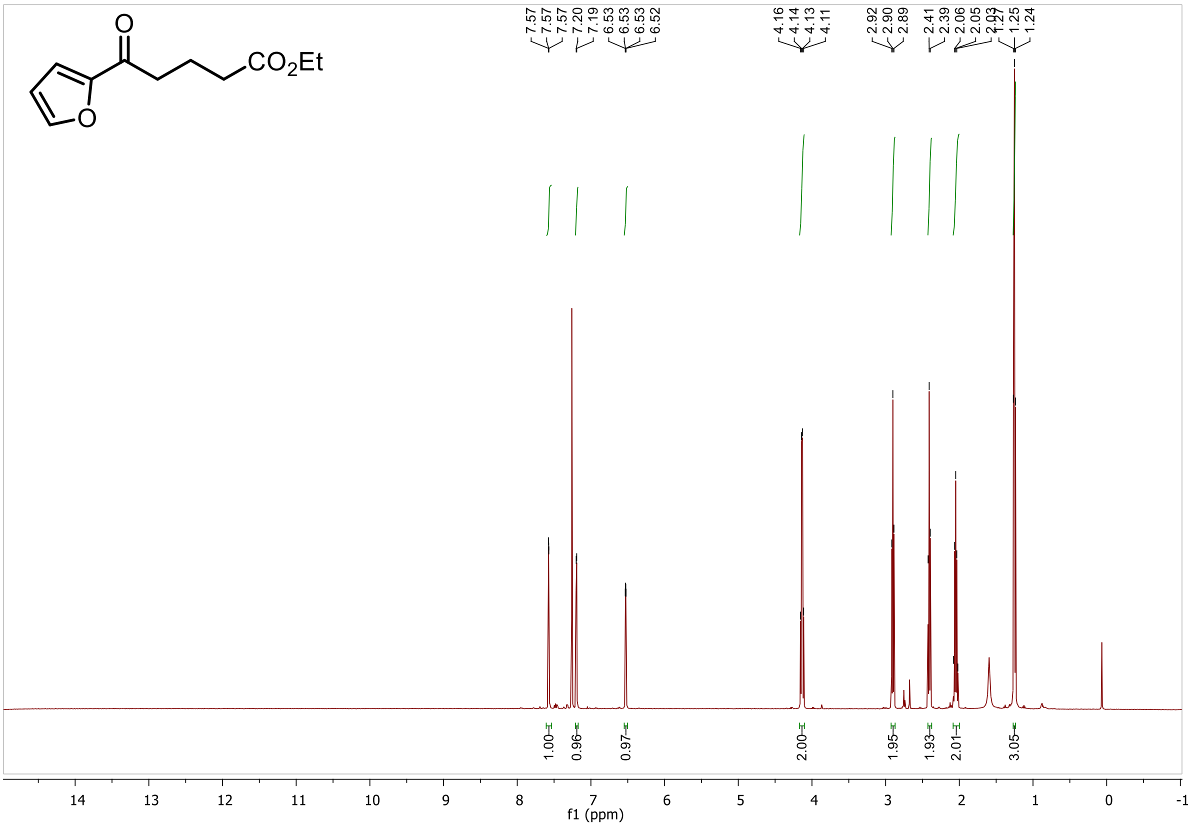

Figure S79. 3s, $^{13}C\{^{1}H\}$ NMR, 126 MHz, $CDCl_3$

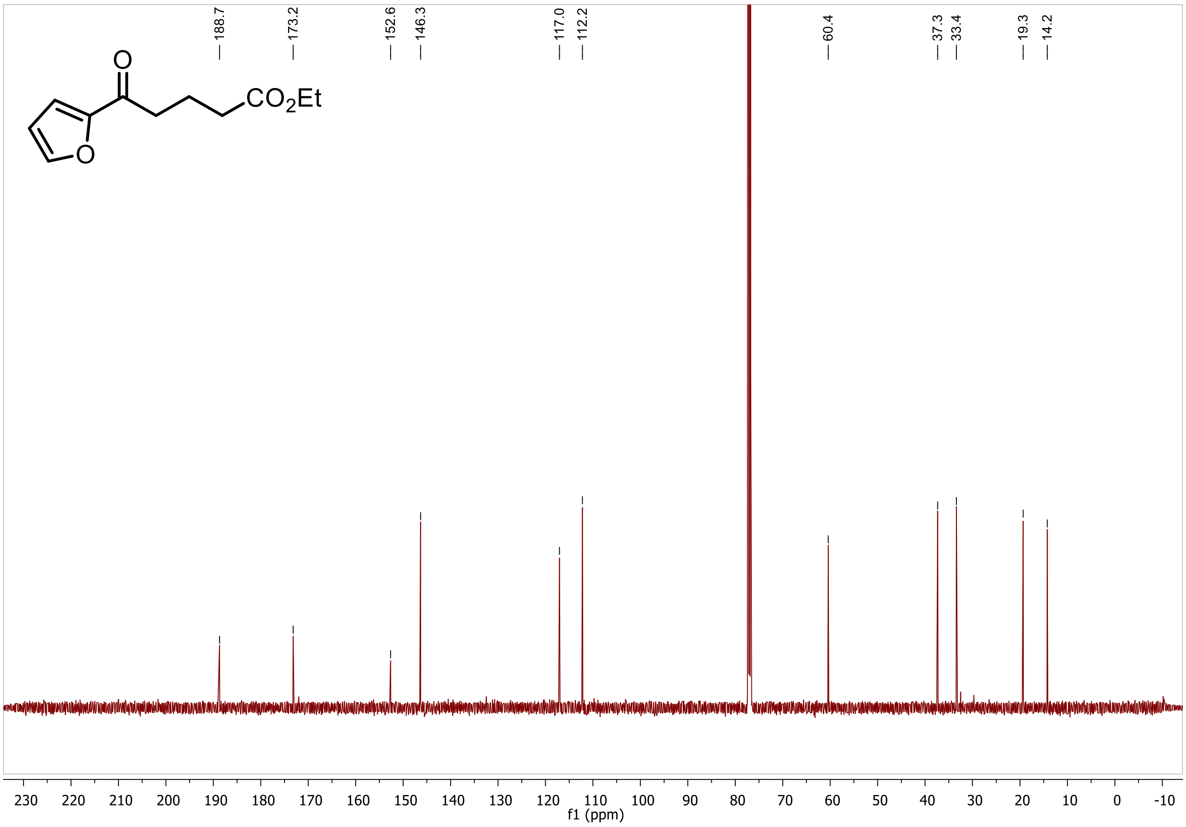

Figure S80. 3u, ^{1}H NMR, 500 MHz, $CDCl_3$

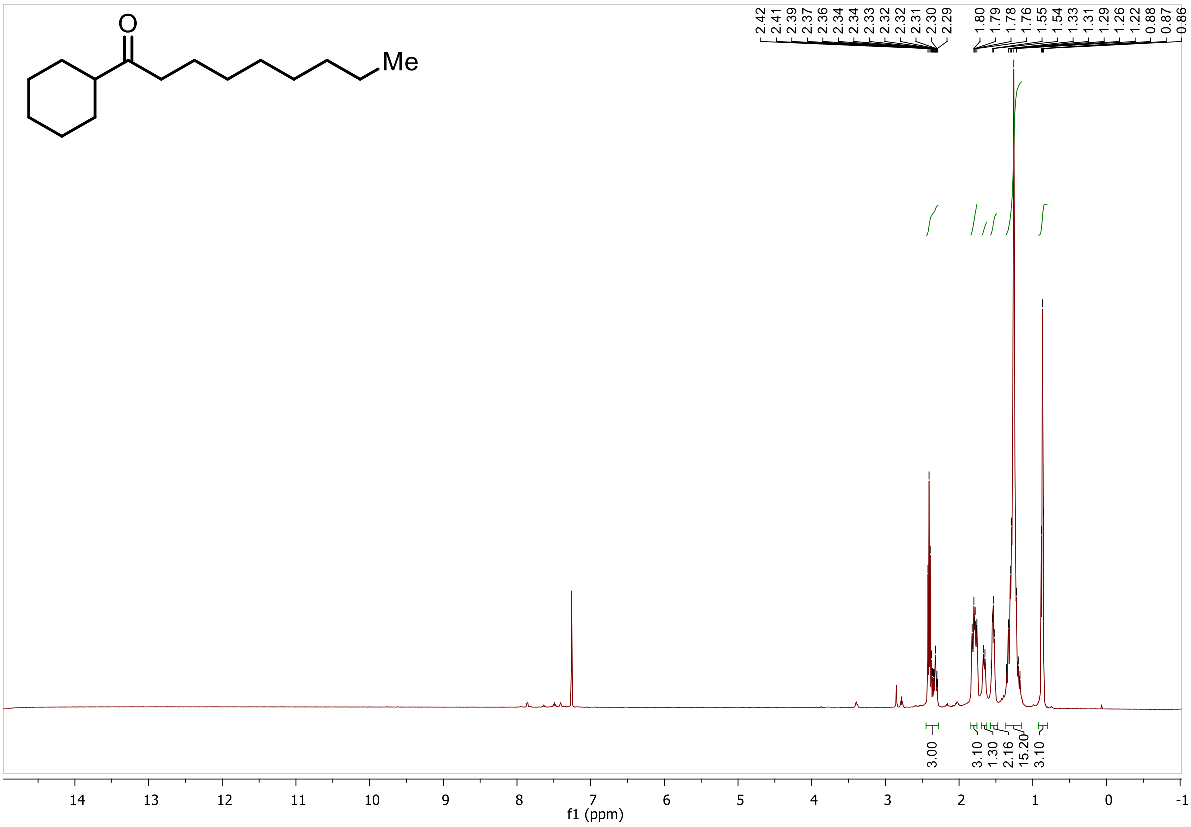

Figure S81. 3u, $^{13}C\ \{^{1}H\}$ NMR, 126 MHz, $CDCl_3$

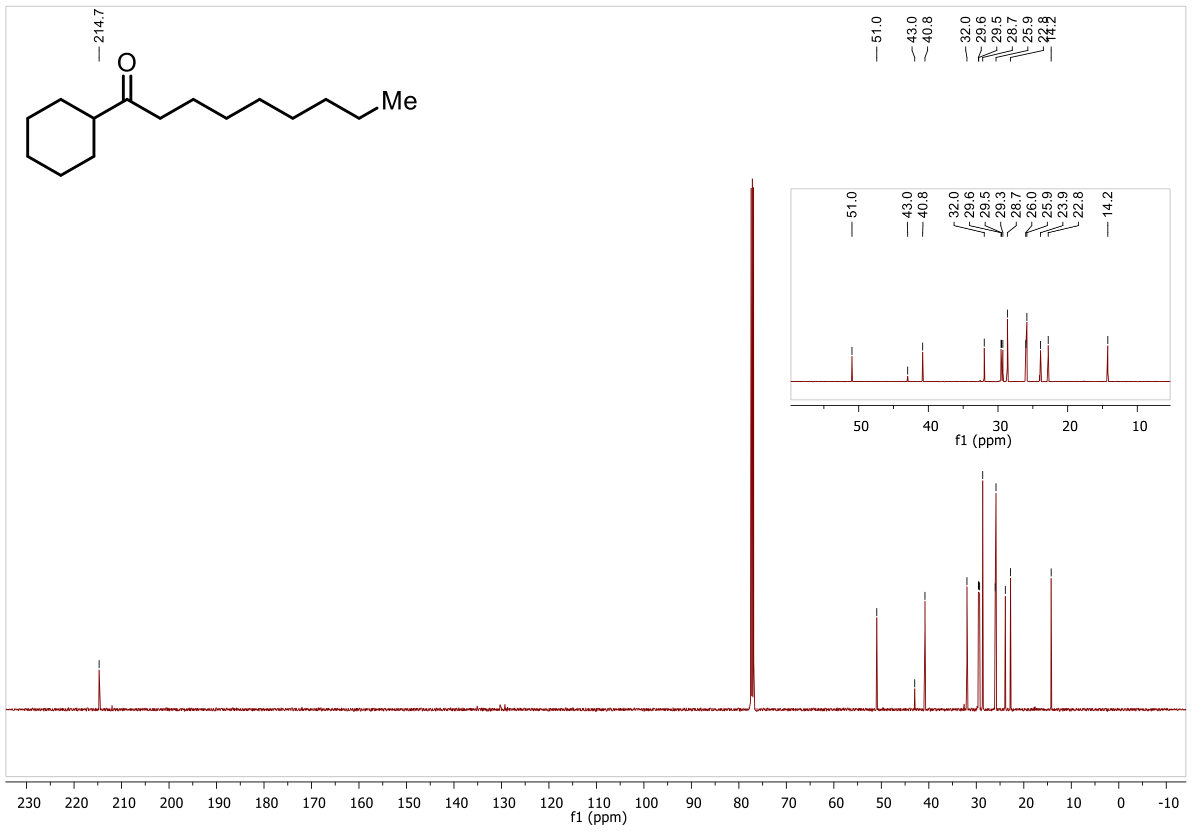

Figure S82. 3v, ^{1}H NMR, 500 MHz, $CDCl_3$

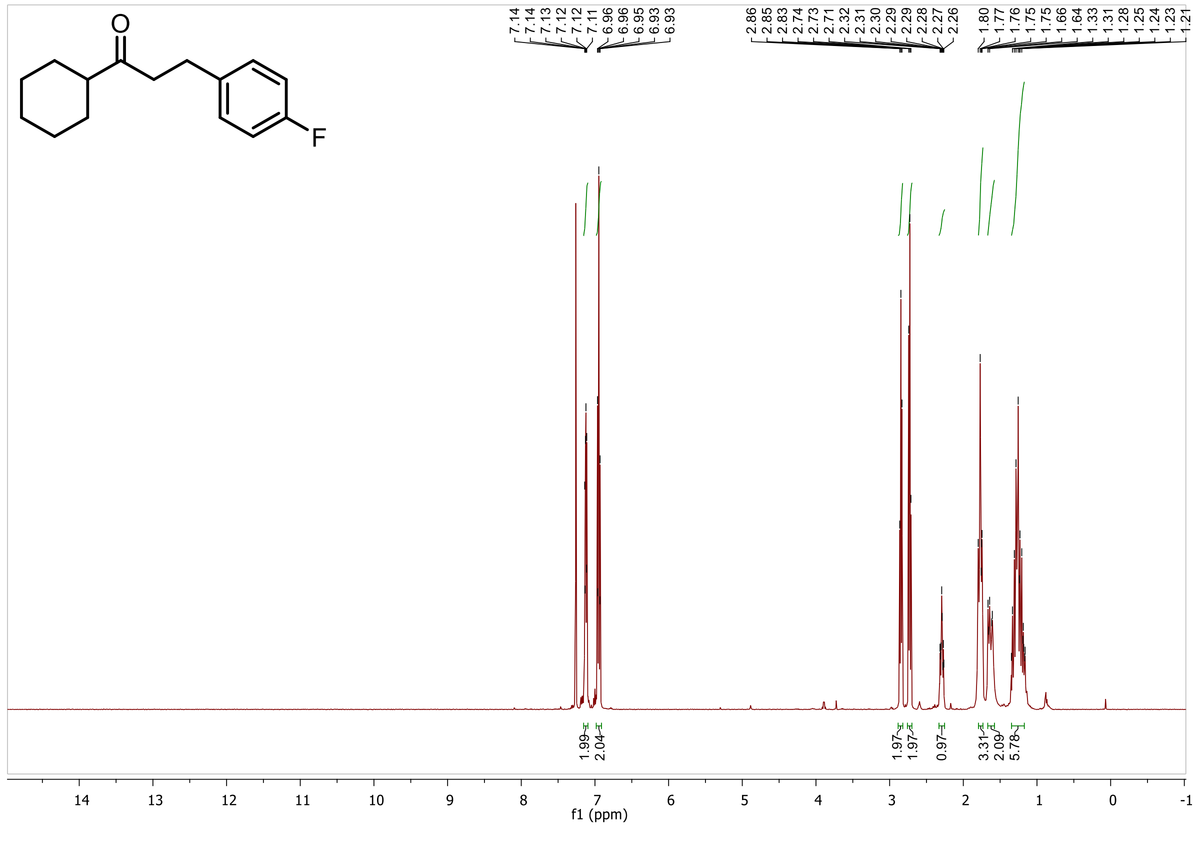

Figure S83. 3v, ^{13}C {^{1}H} NMR, 126 MHz, $CDCl_3$

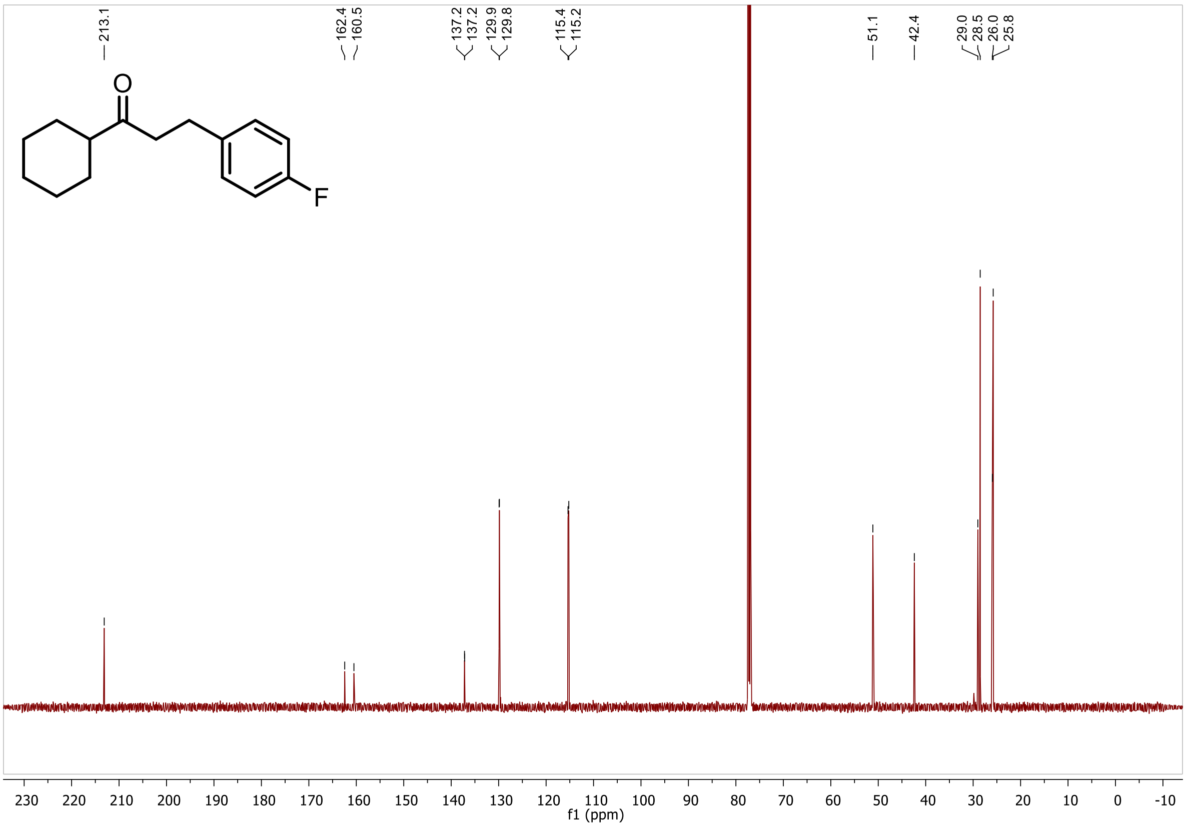

Figure S84. 3v, ^{19}F NMR, 471 MHz, $CDCl_3$

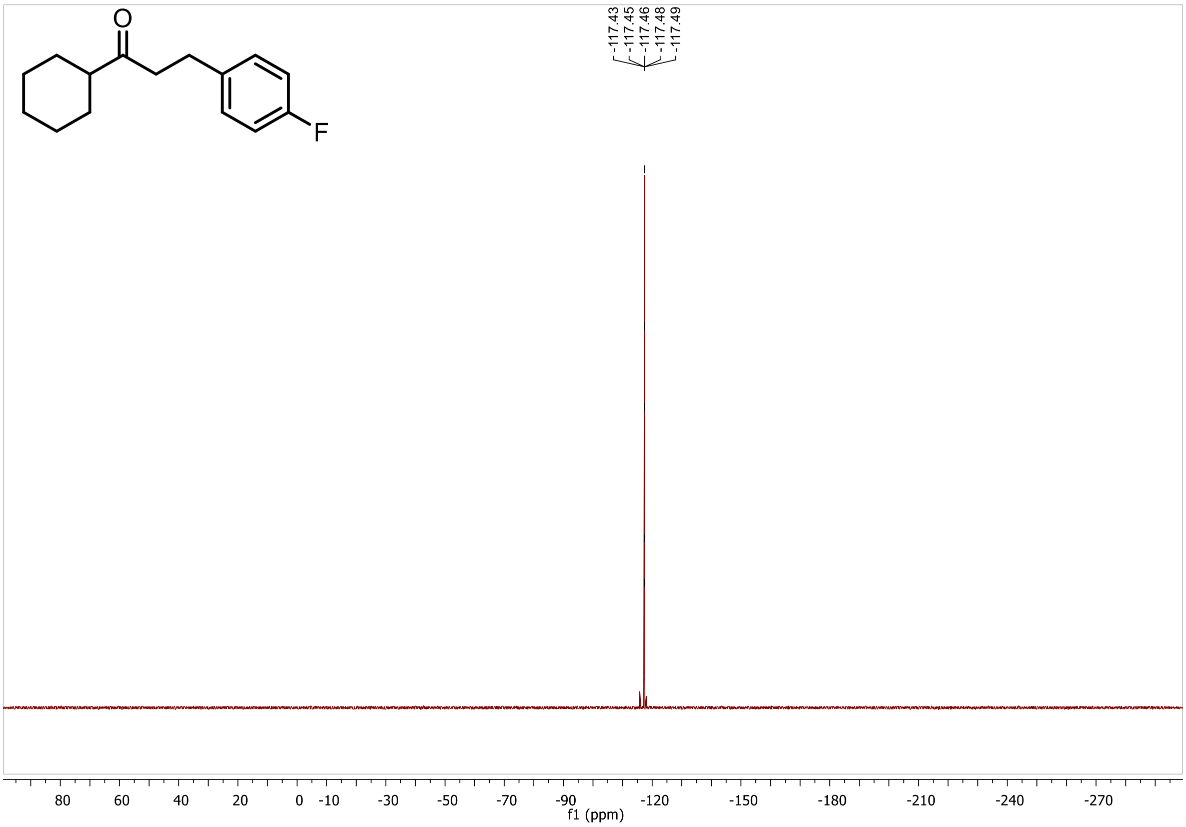

Figure S85. 3w, ^{1}H NMR, 500 MHz, $CDCl_3$

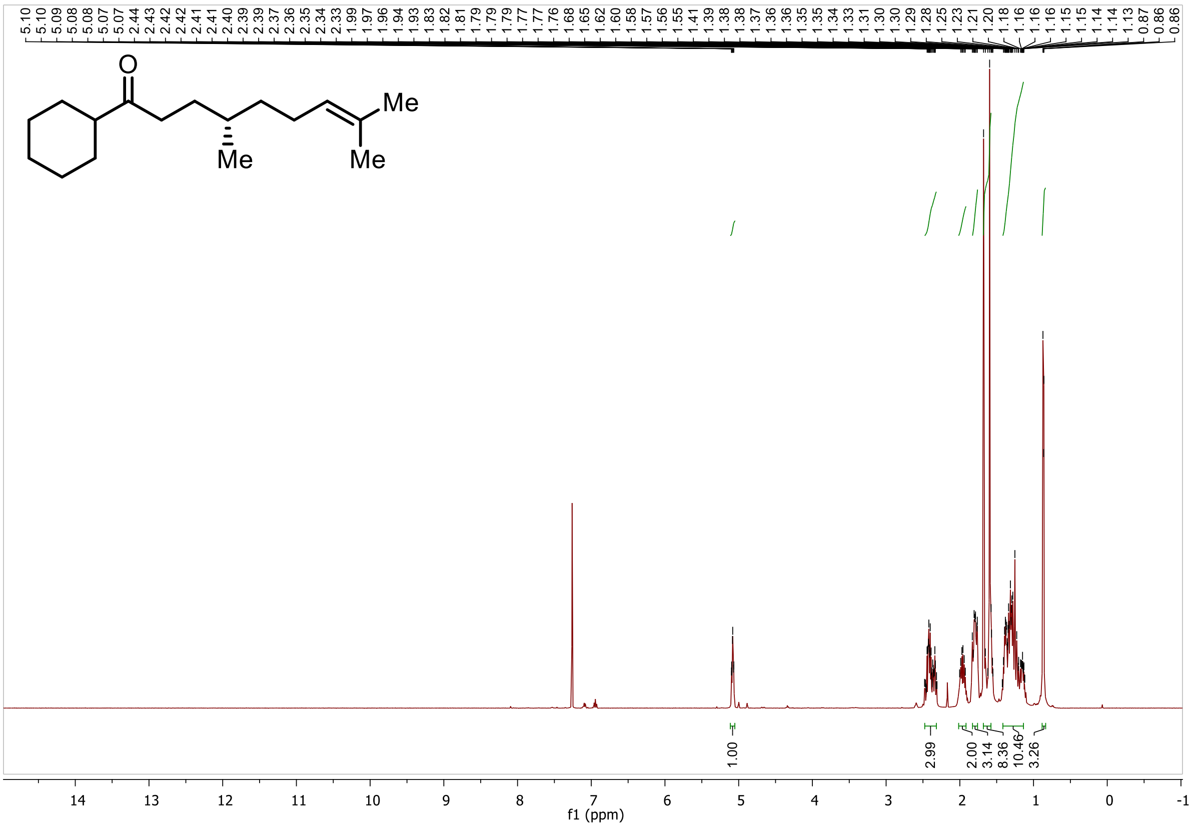

Figure S86. 3w, ^{13}C {^{1}H} NMR, 126 MHz, $CDCl_3$

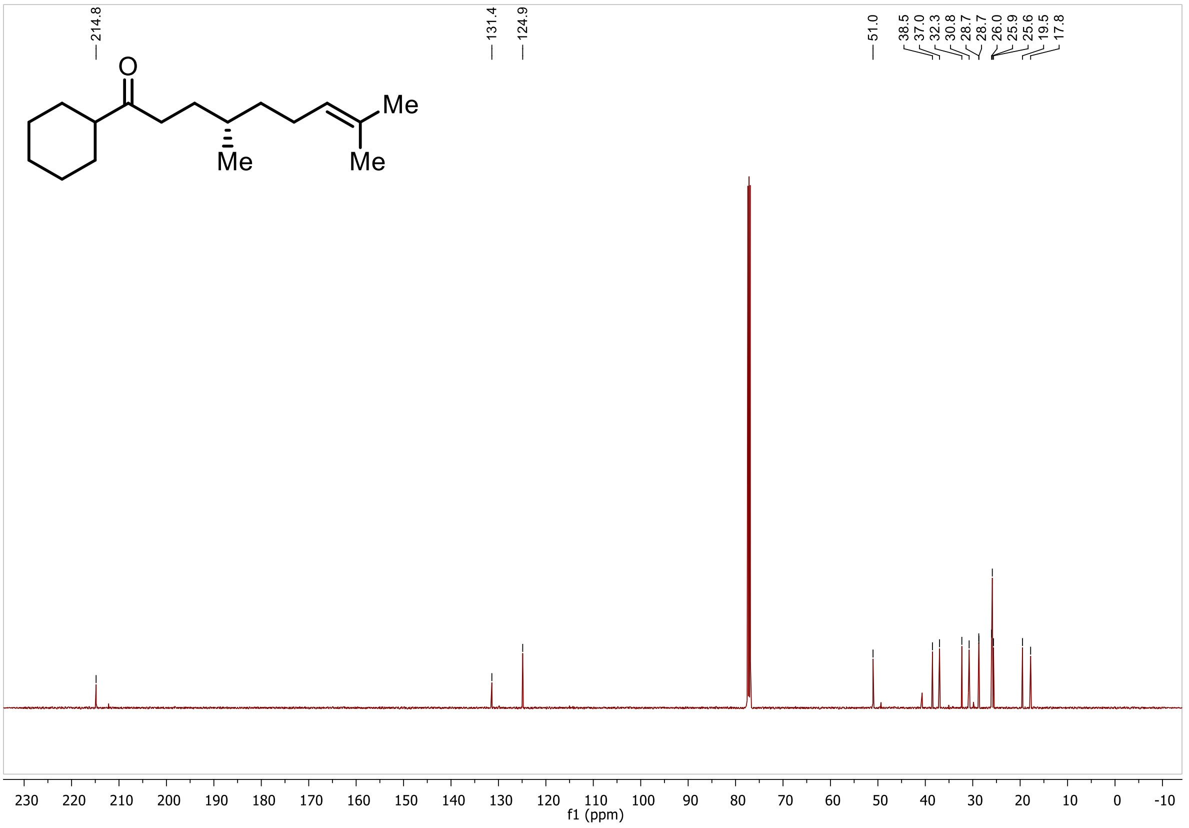

Figure S87. 3t, ^{1}H NMR, 500 MHz, $CDCl_3$

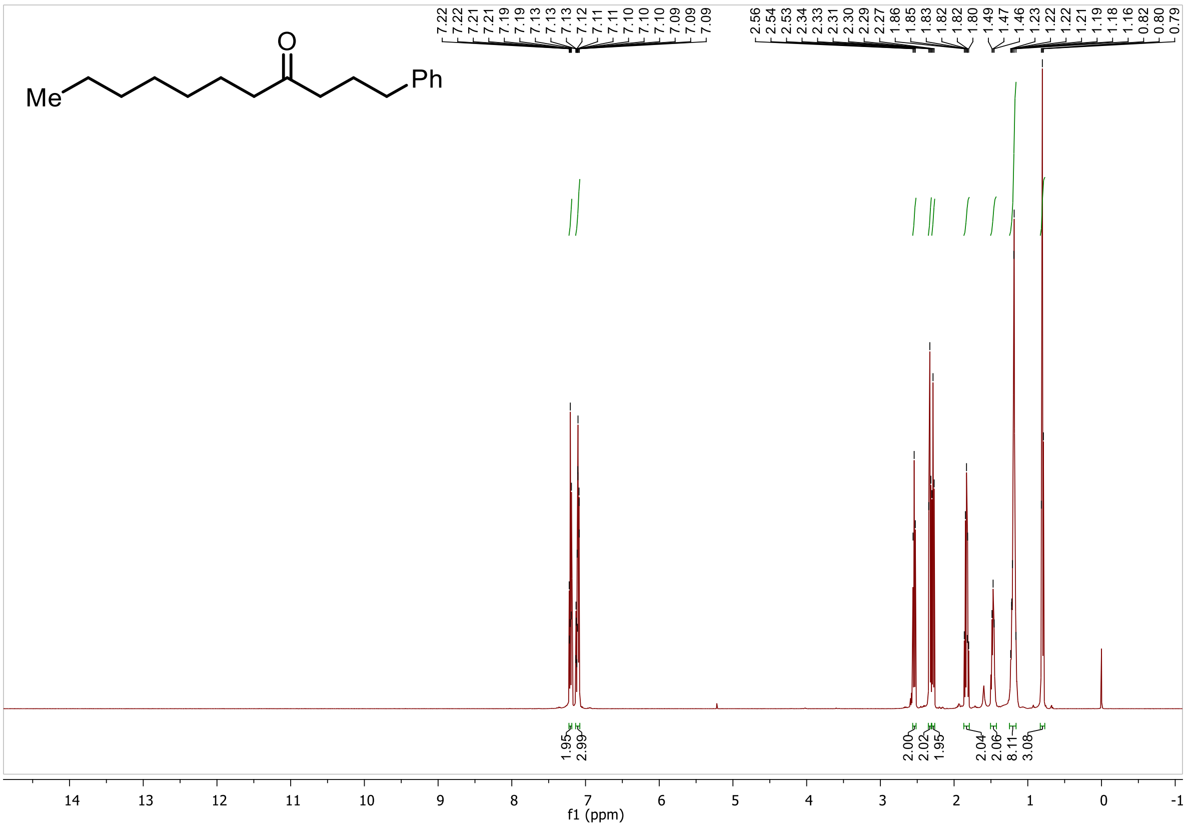

Figure S88. 3t, ^{13}C {^{1}H} NMR, 126 MHz, $CDCl_3$

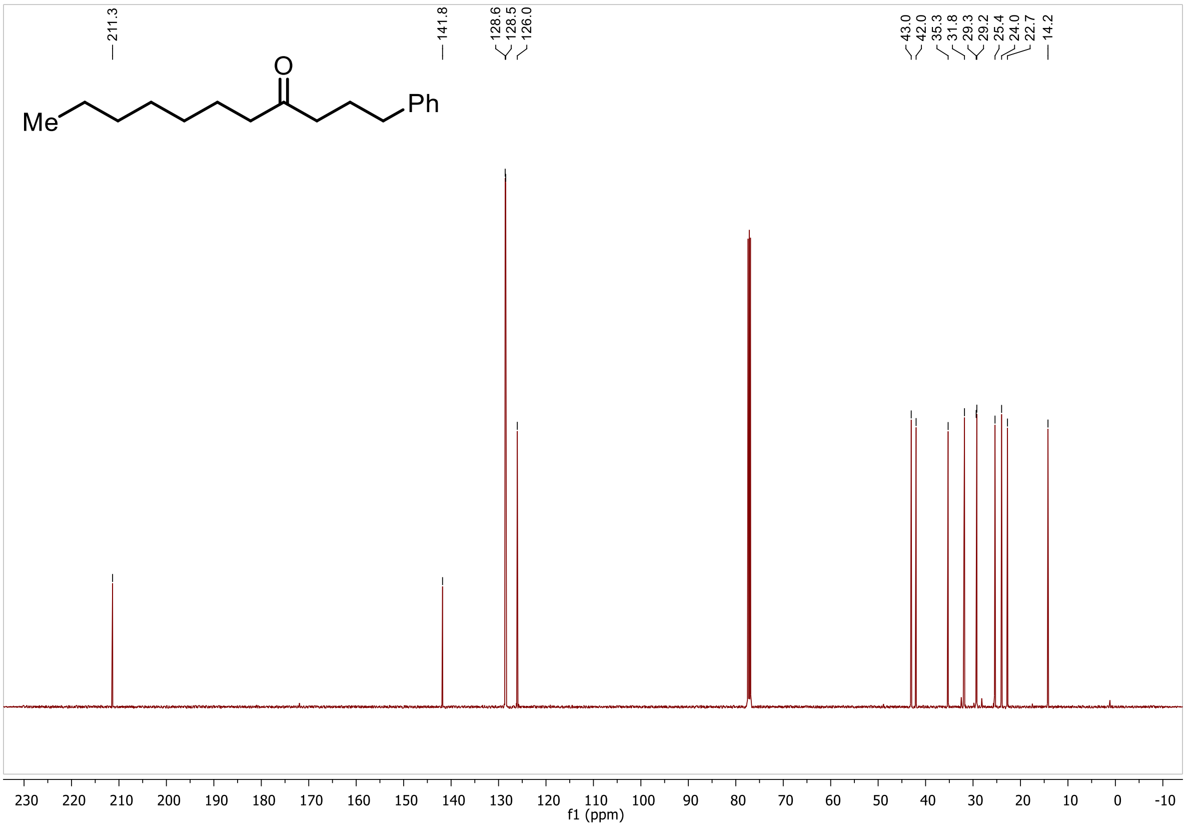

Figure S89. 3x, ^{1}H NMR, 500 MHz, $CDCl_3$

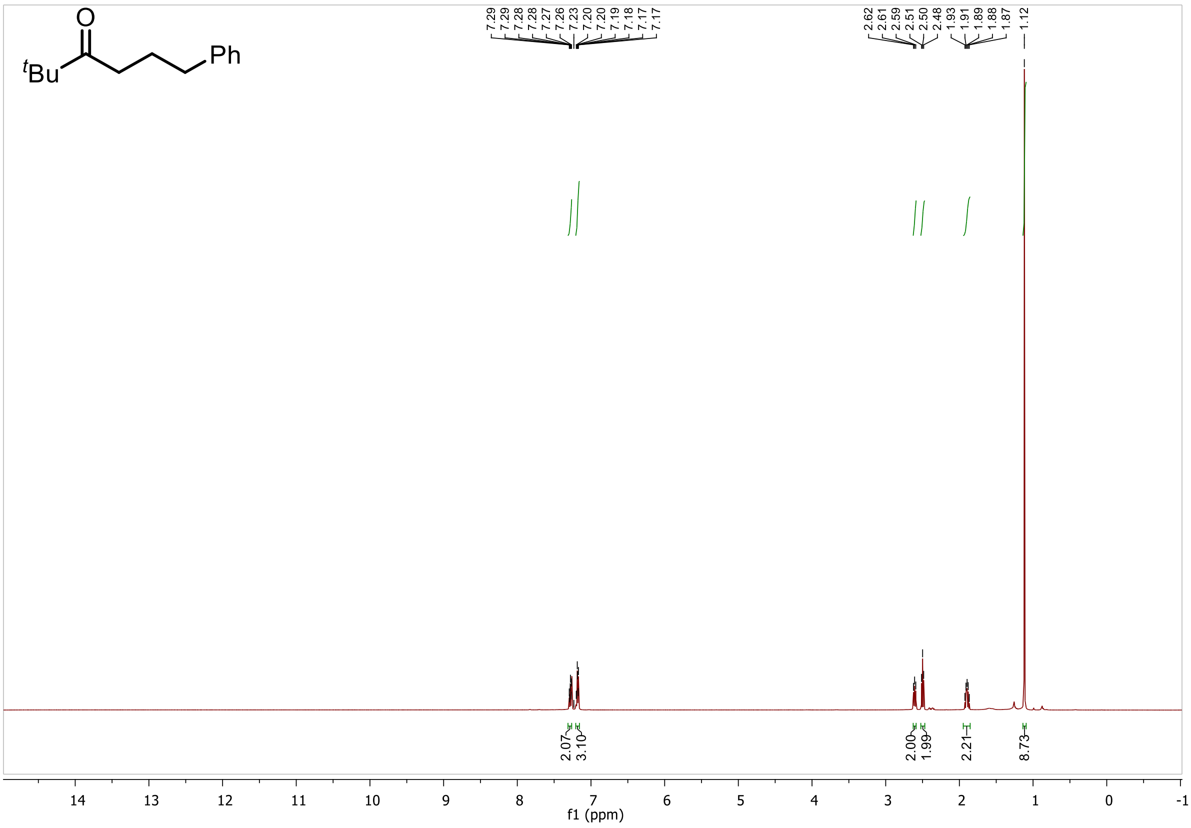

Figure S90. 3x, ^{13}C {^{1}H} NMR, 126 MHz, $CDCl_3$

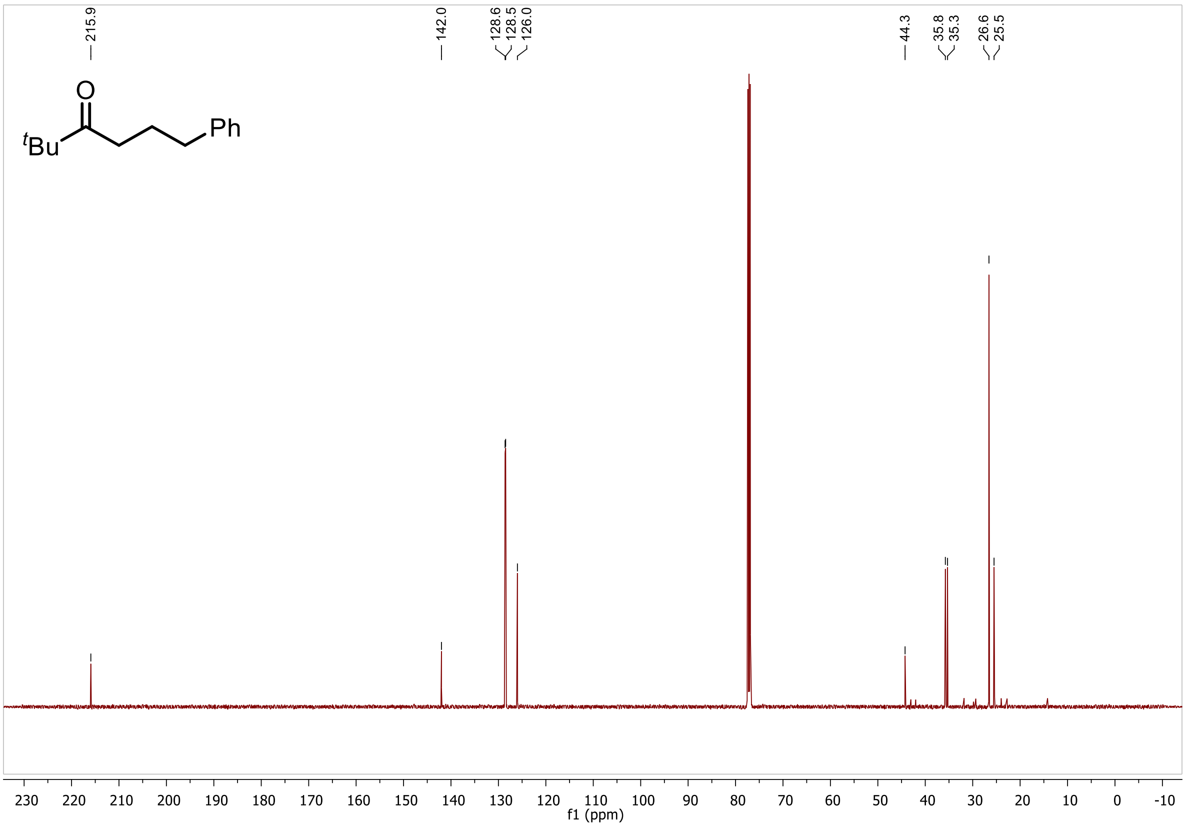

Figure S91. 3k, ^{1}H NMR, 500 MHz, $CDCl_3$

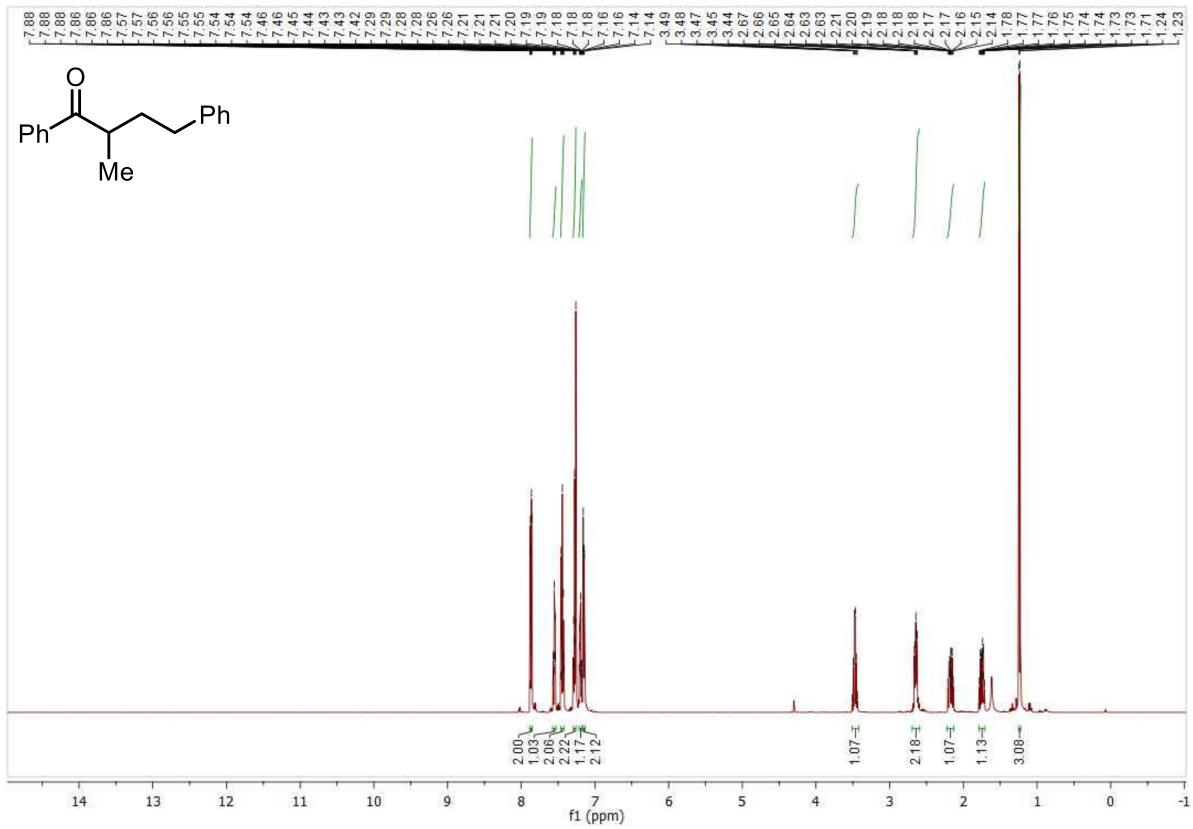

Figure S92. 3k, ^{13}C {^{1}H} NMR, 126 MHz, $CDCl_3$

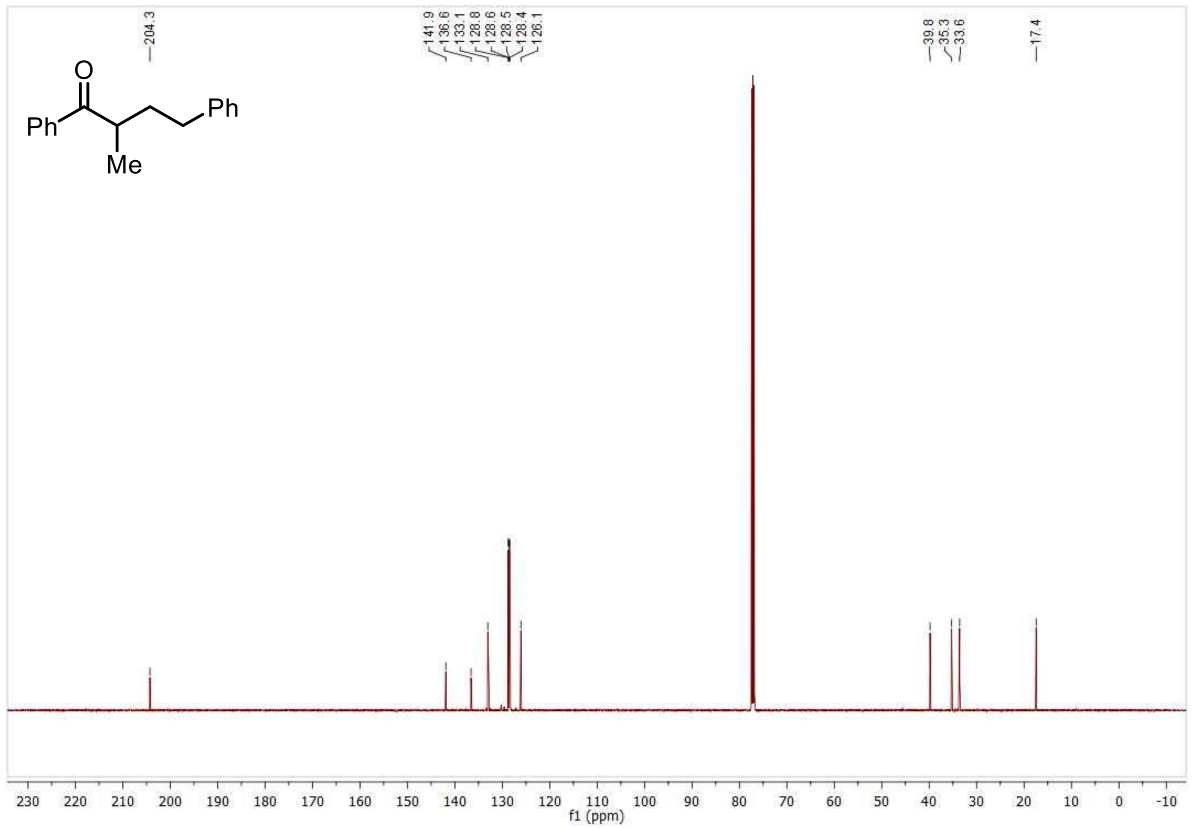

Figure S93. 3yb, ^{1}H NMR, 500 MHz, $CDCl_3$

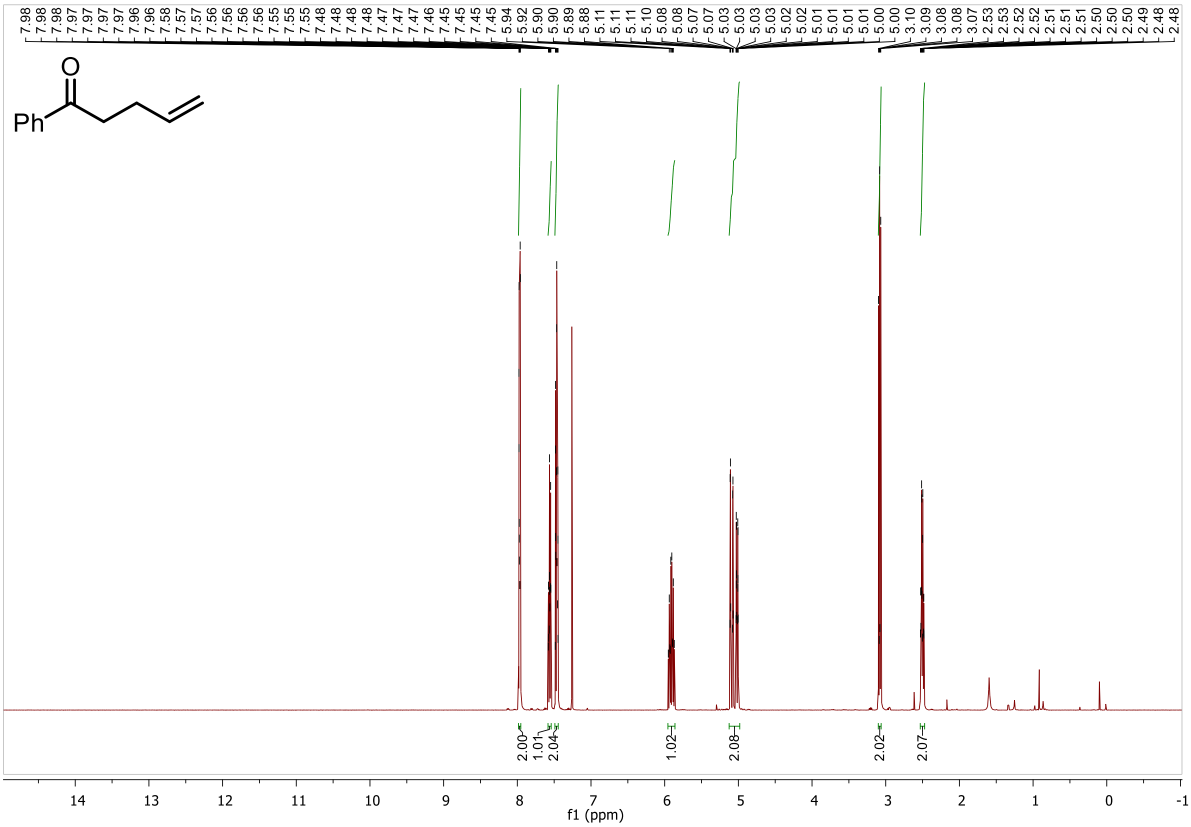

Figure S94. 3yb, ^{13}C {^{1}H} NMR, 126 MHz, $CDCl_3$

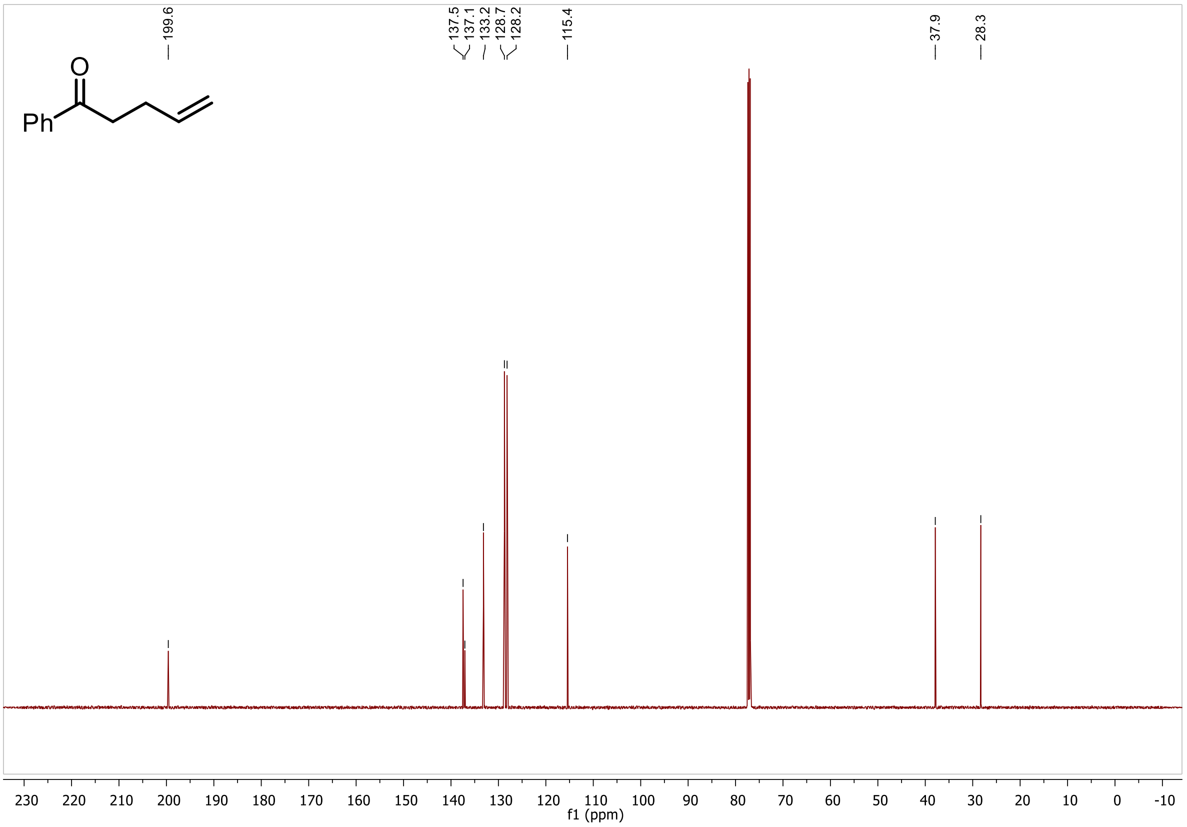

Figure S95. L1, ^{1}H NMR, 500 MHz, $(CD_3)SO$

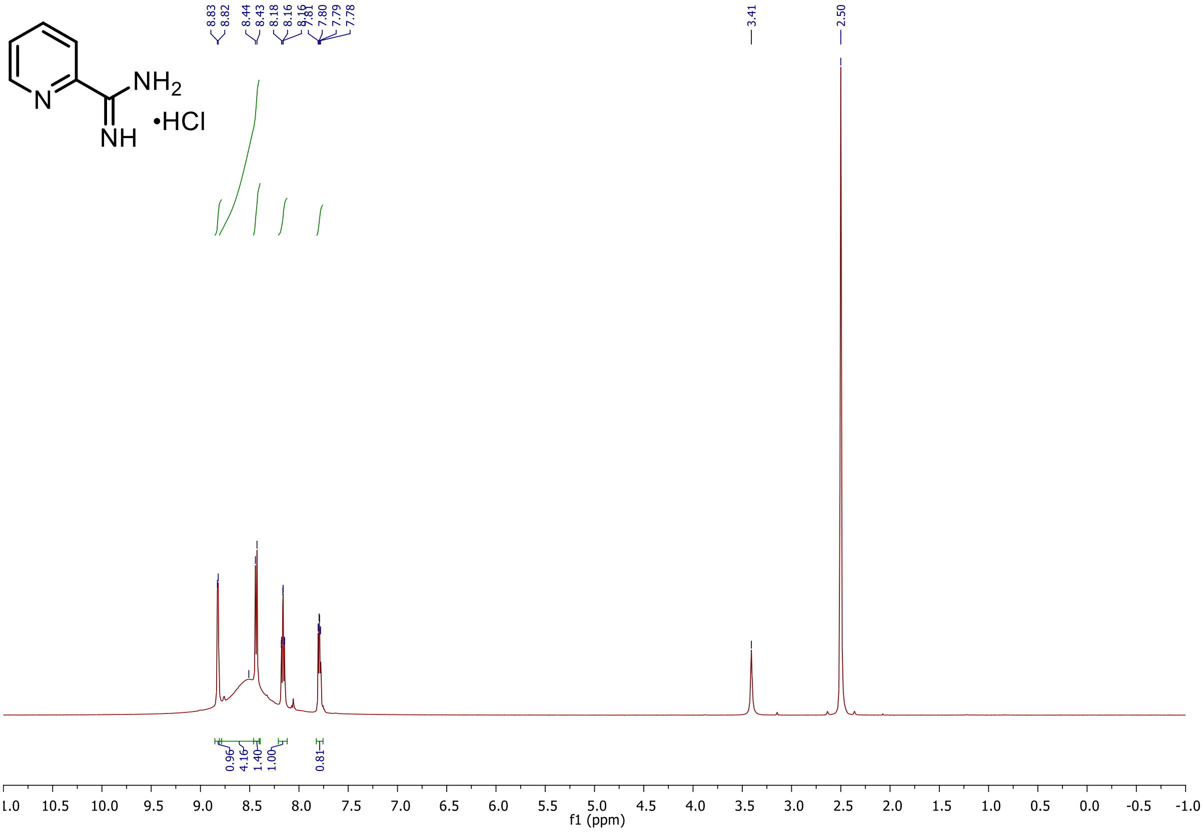

Figure S96. L1, ^{13}C {^{1}H} NMR, 126 MHz, $(CD_3)SO$

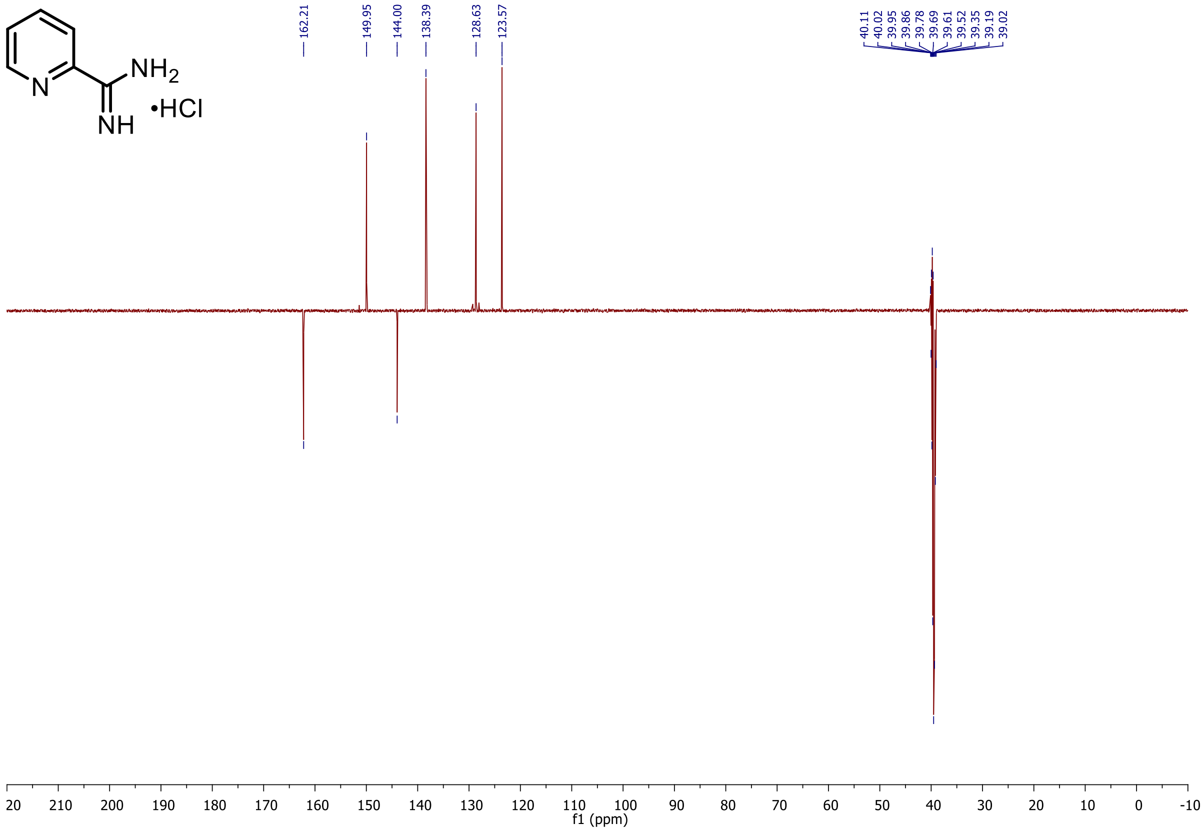

Figure S97. L9, ^{1}H NMR, 500 MHz, $(CD_3)SO$

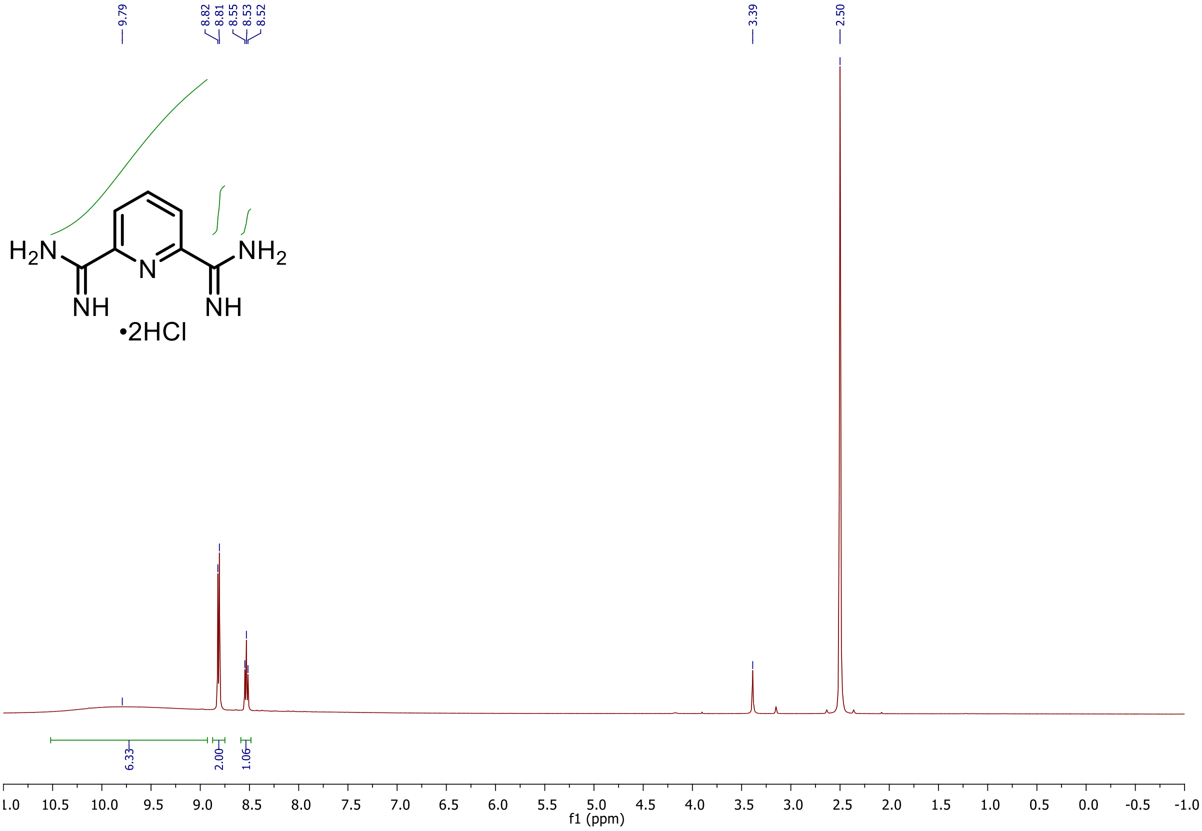

Figure S98. L9, ^{13}C {^{1}H} NMR, 126 MHz, $(CD_3)SO$

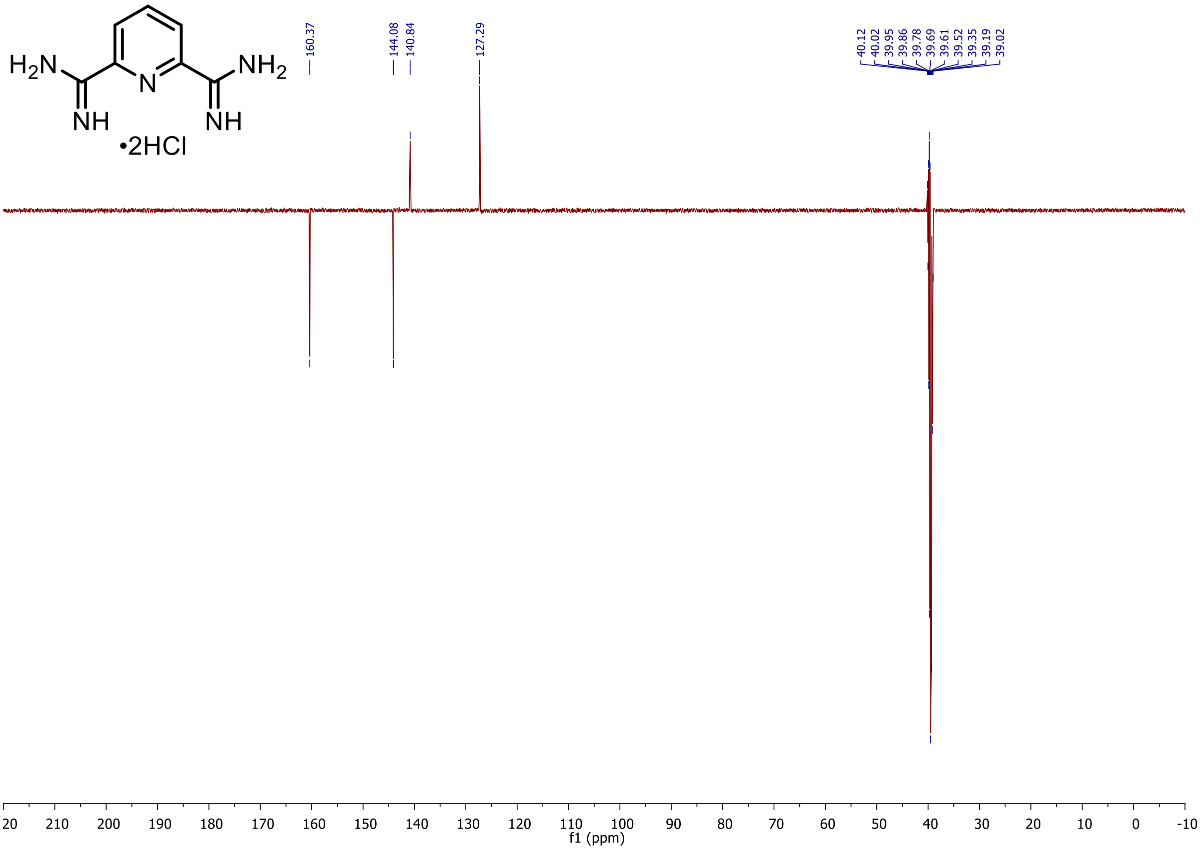

Figure S99. 5aa, ^{1}H NMR, 500 MHz, $CDCl_3$

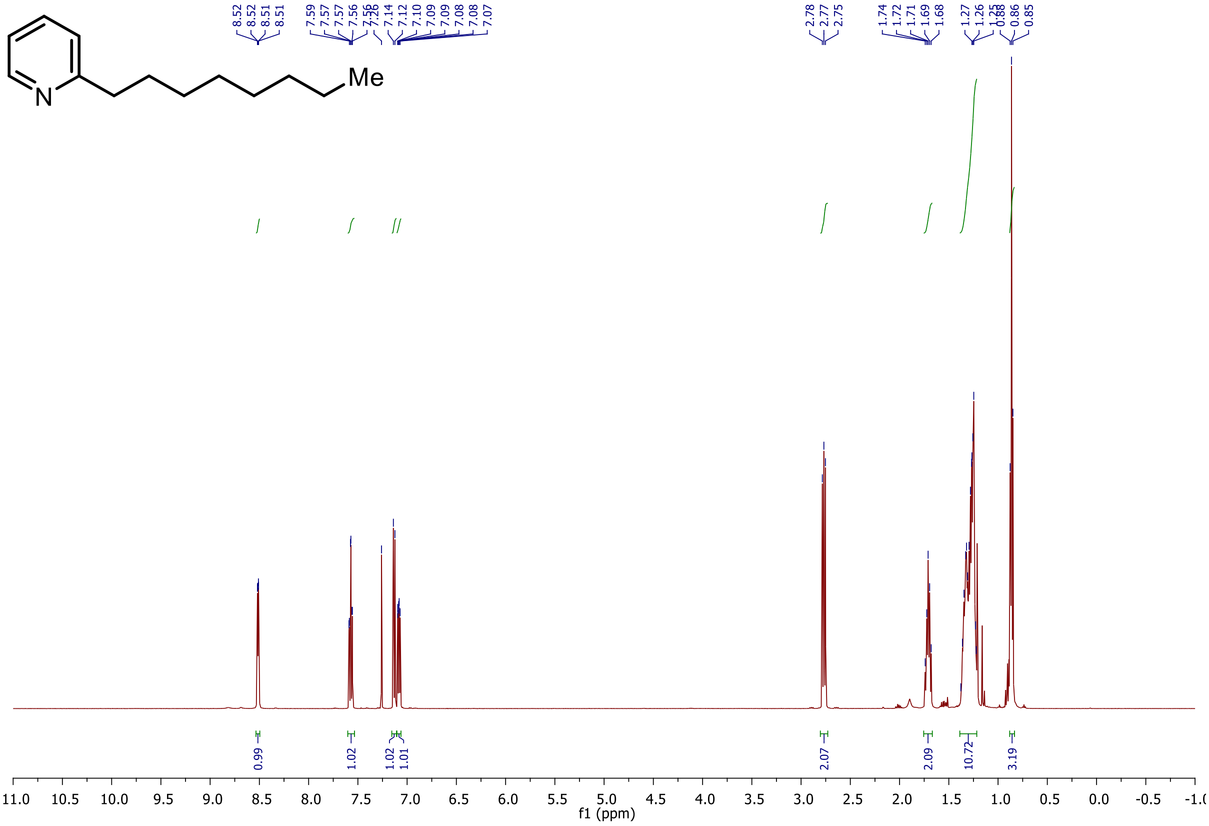

Figure S100. 5aa, ^{13}C {^{1}H} NMR, 126 MHz, $CDCl_3$

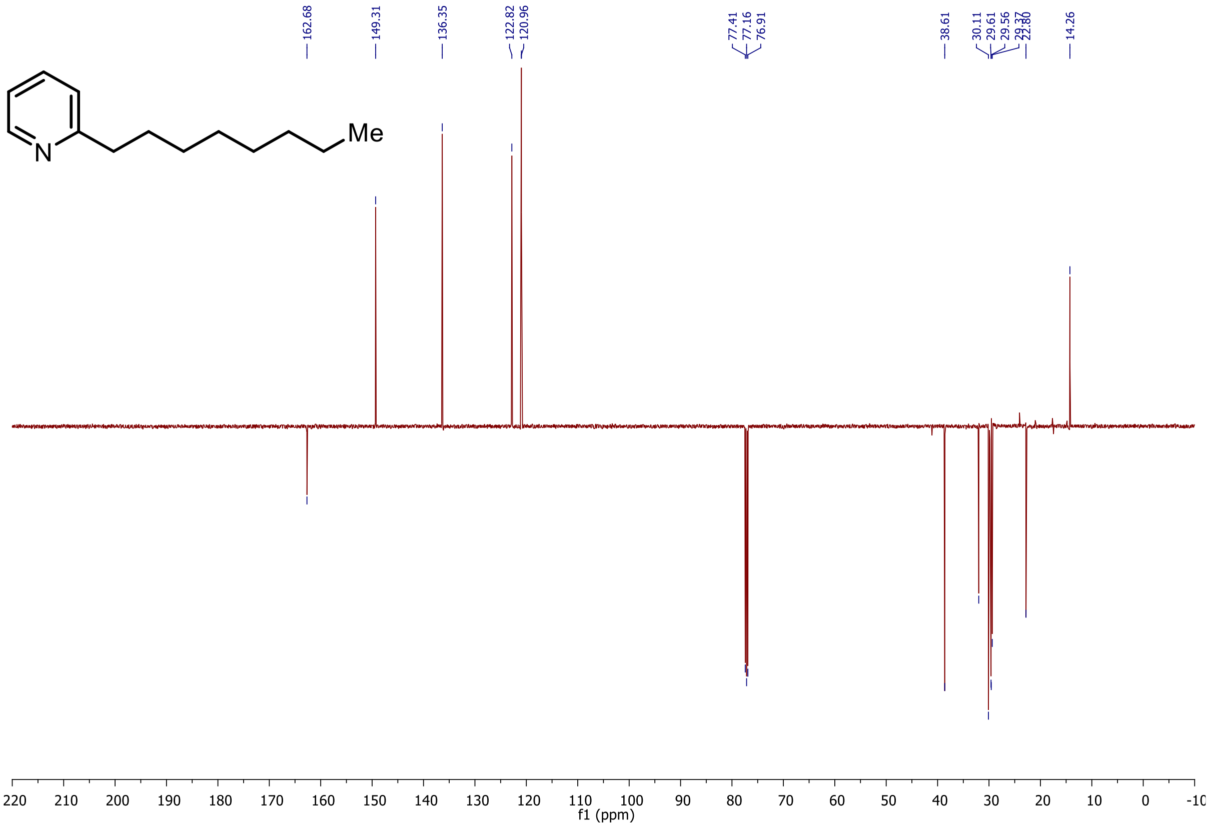

Figure S101. 5ab, ^{1}H NMR, 500 MHz, $CDCl_3$

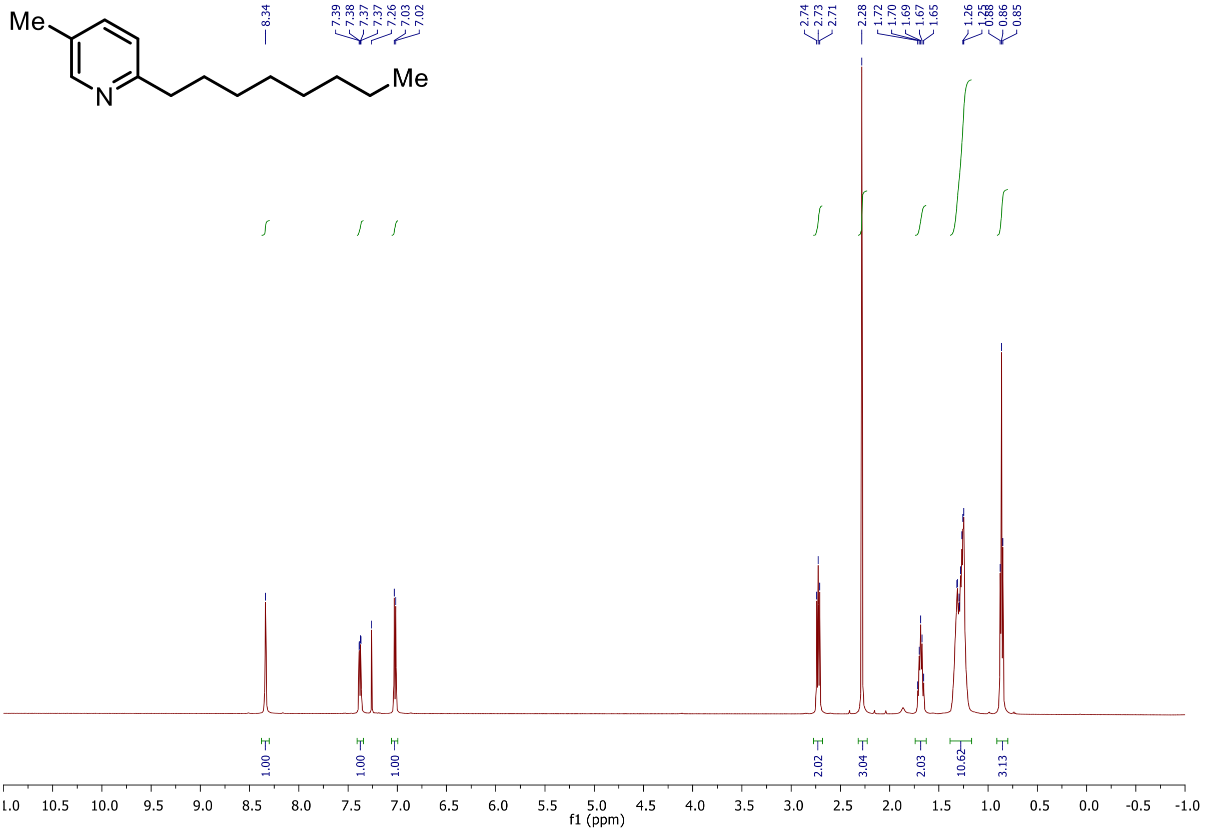

Figure S102. 5ab, ^{13}C {^{1}H} NMR, 126 MHz, $CDCl_3$

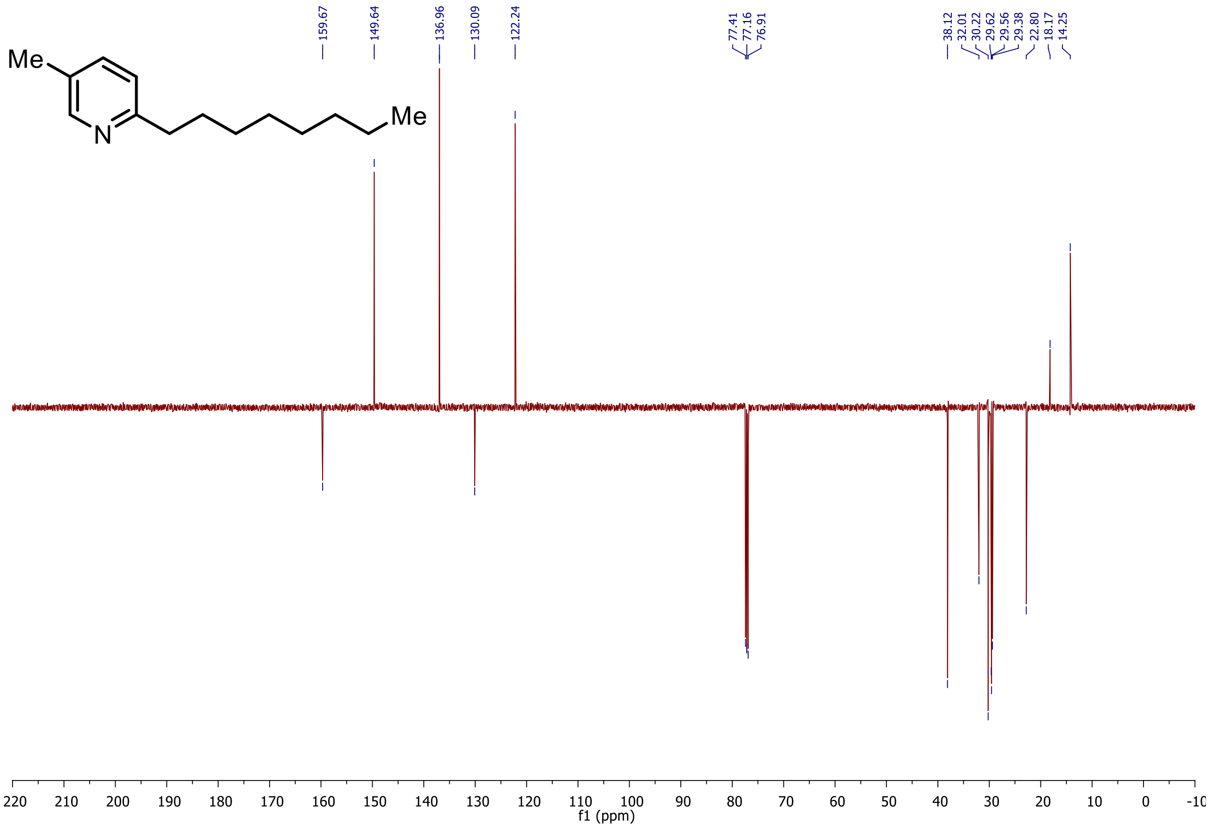

Figure S103. 5ac, ^{1}H NMR, 400 MHz, $CDCl_3$

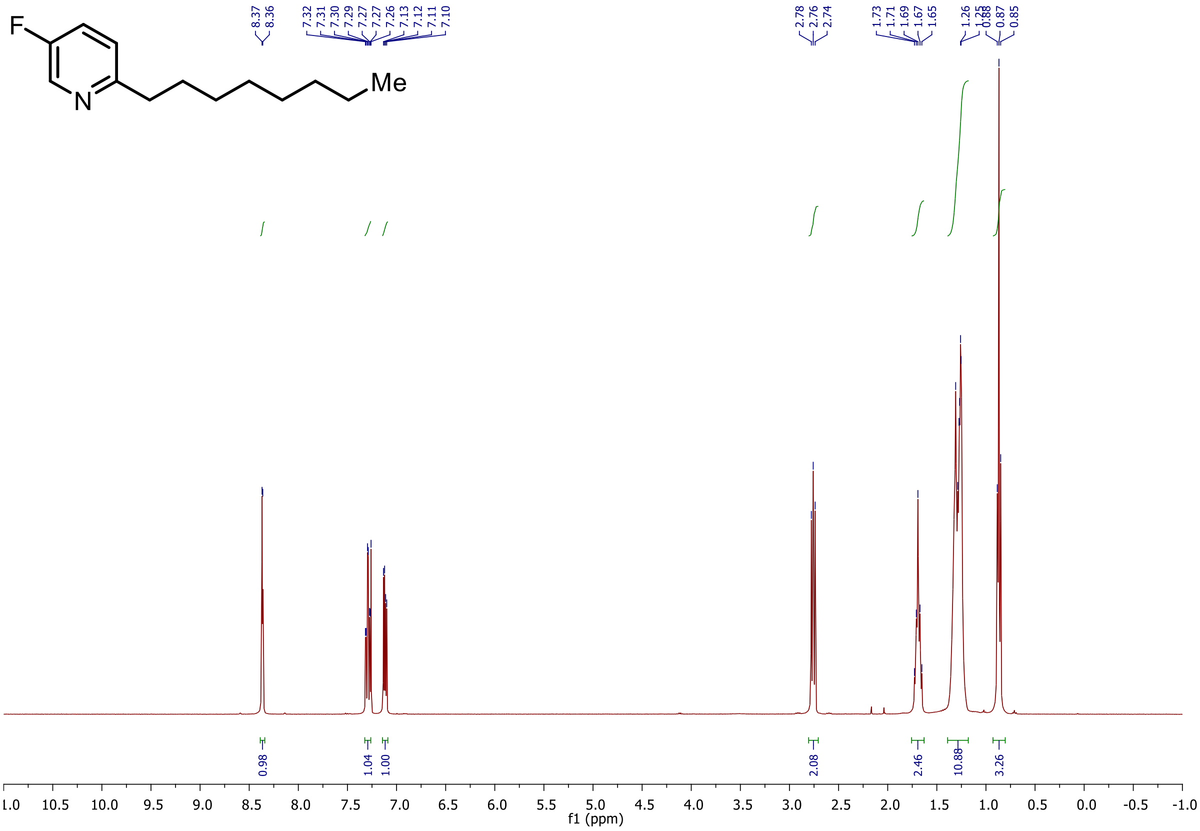

Figure S104. 5ac, ^{13}C {^{1}H} NMR, 101 MHz, $CDCl_3$

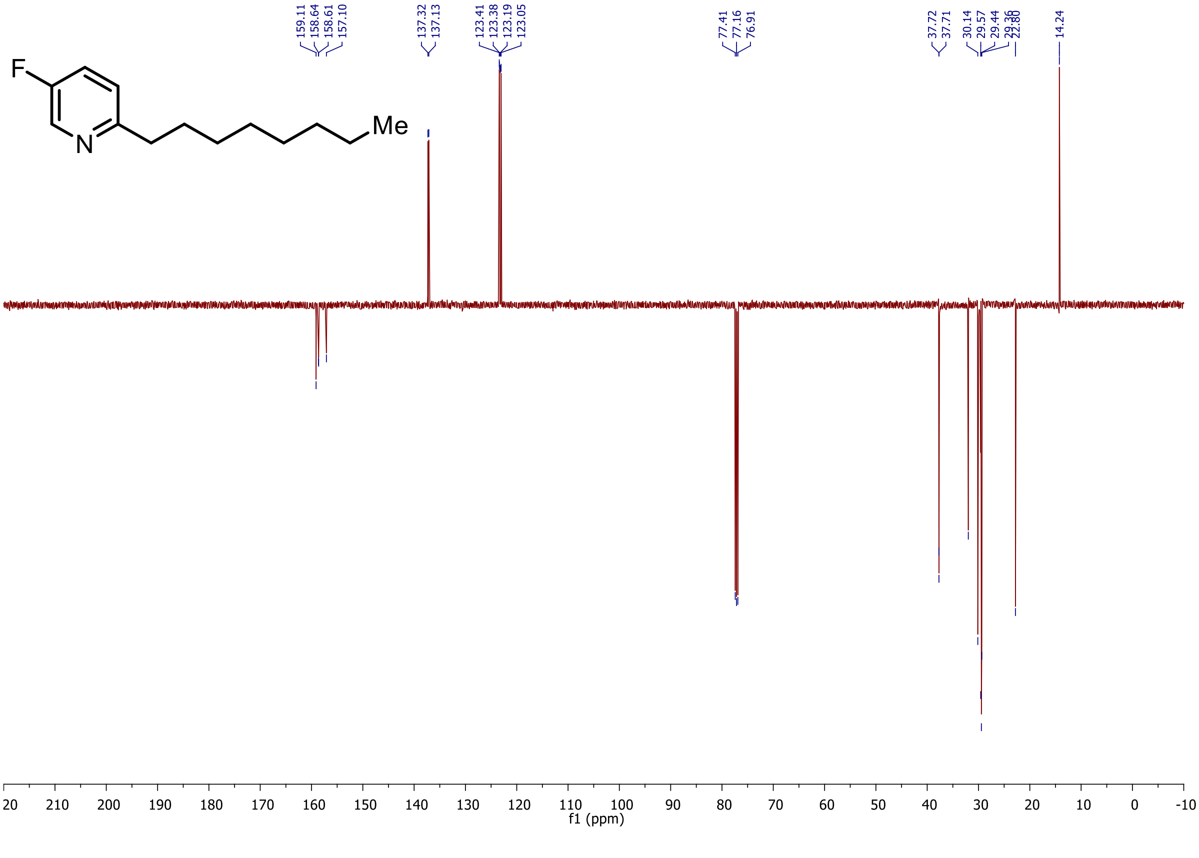

Figure S105. 5ac, ^{19}F {^{1}H} NMR, 376 MHz, $CDCl_3$

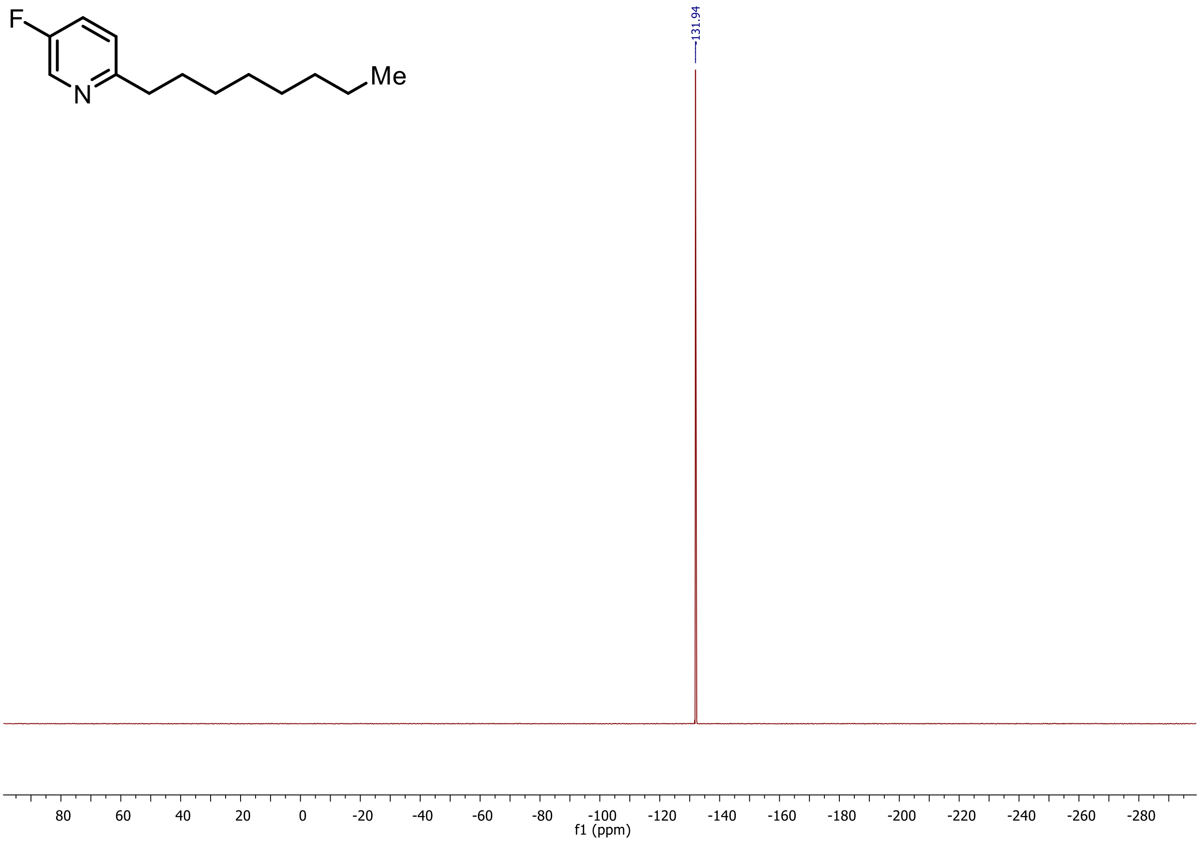

Figure S106. 5ad, ^{1}H NMR, 500 MHz, $CDCl_3$

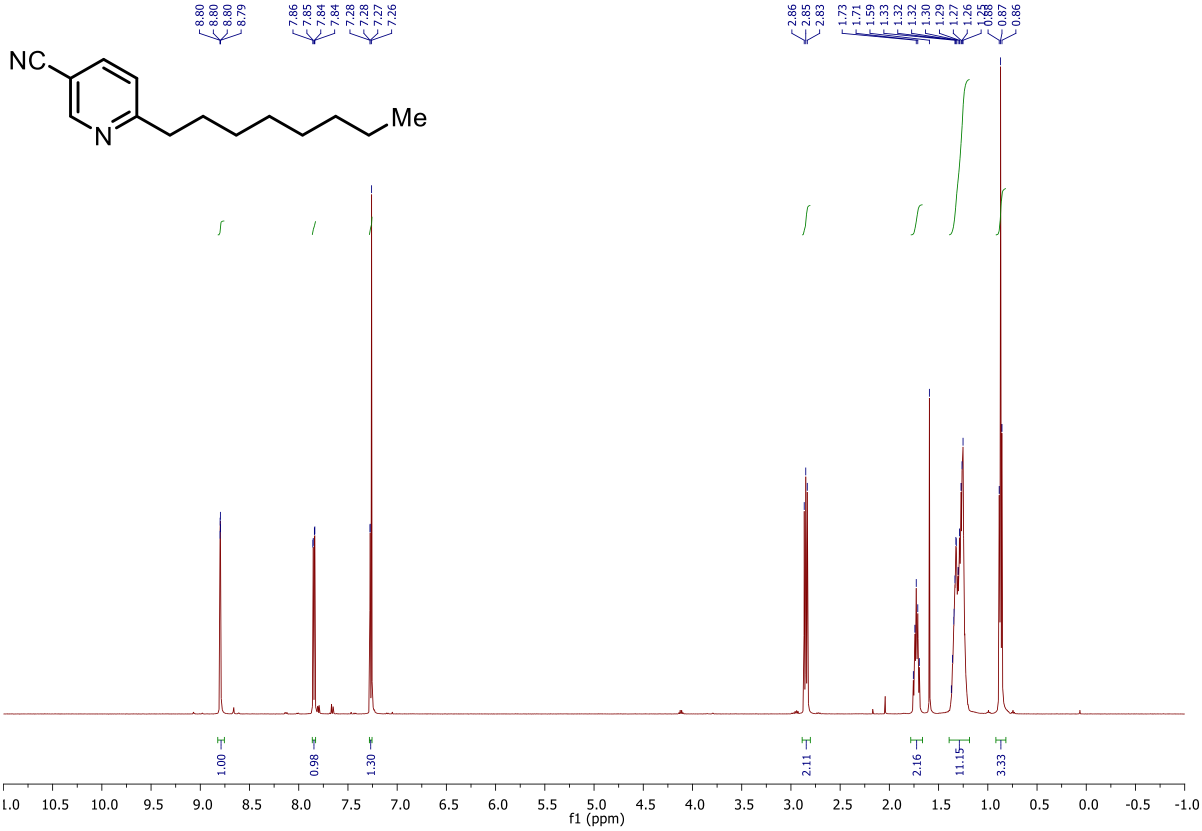

Figure S107. 5ad, $^{13}C\{^1H\}$ NMR, 126 MHz, $CDCl_3$

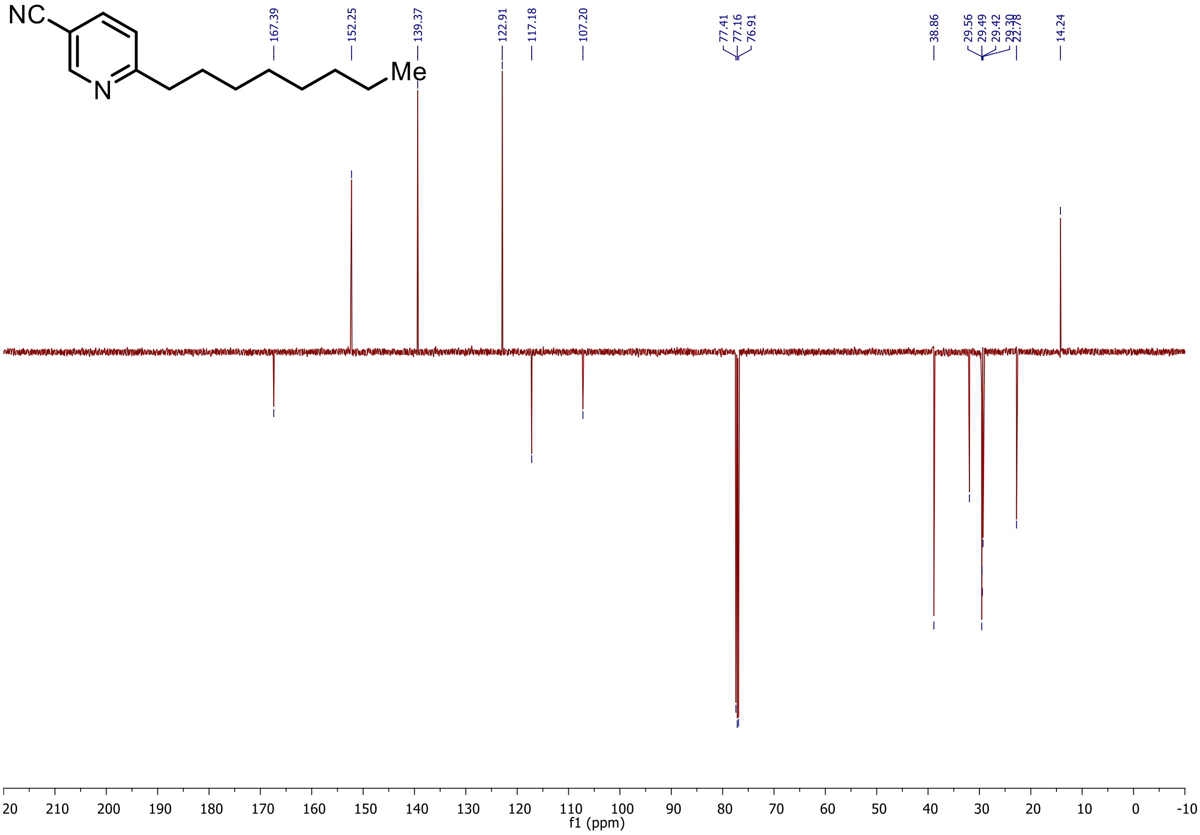

Figure S108. 5ae, ^{1}H NMR, 400 MHz, $CDCl_3$

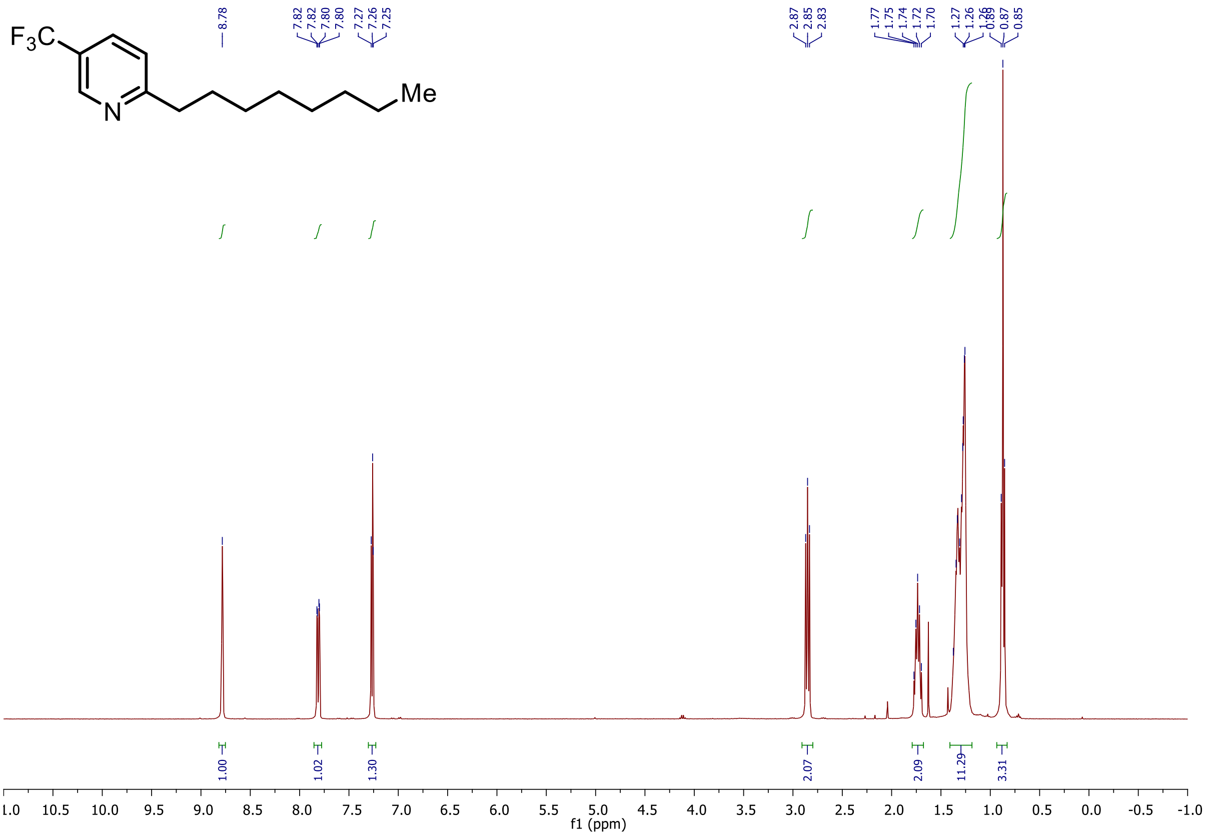

Figure S109. 5ae, ^{13}C {^{1}H} NMR, 101 MHz, $CDCl_3$

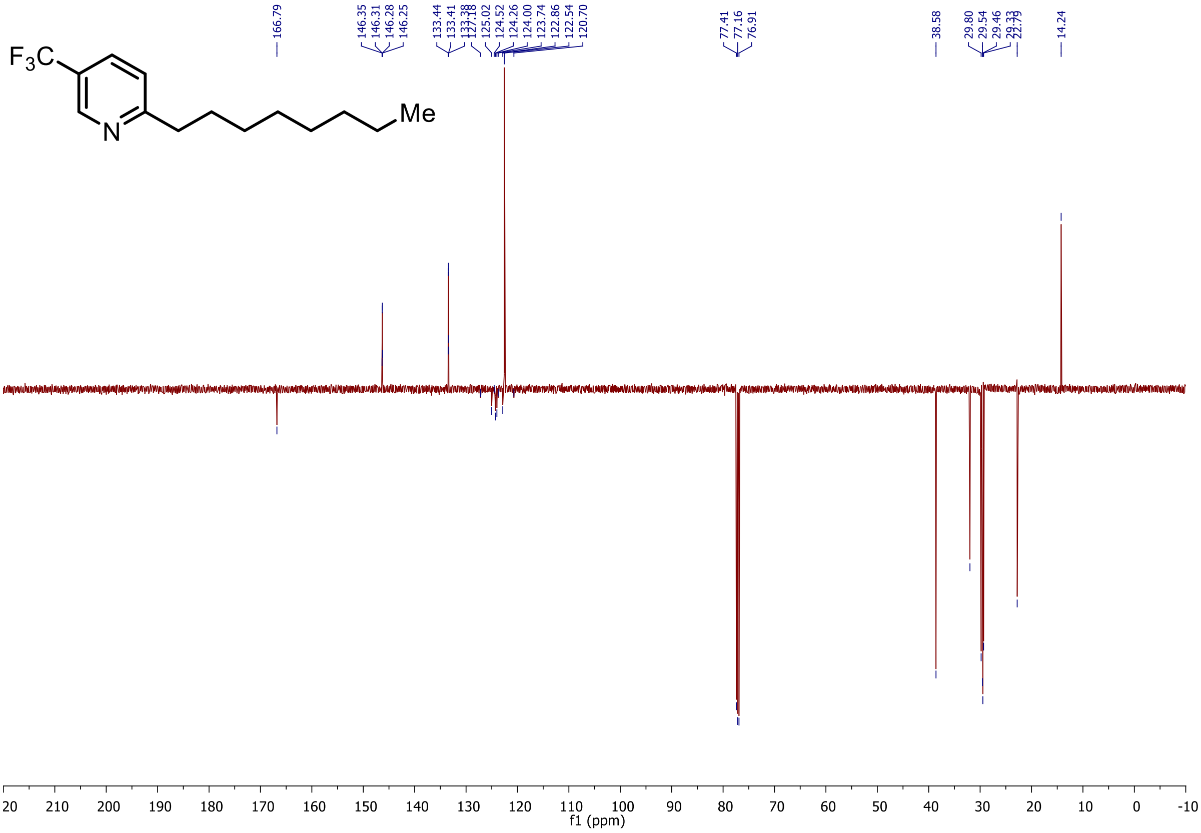

Figure S110. 5ae, ^{19}F {^{1}H} NMR, 376 MHz, $CDCl_3$

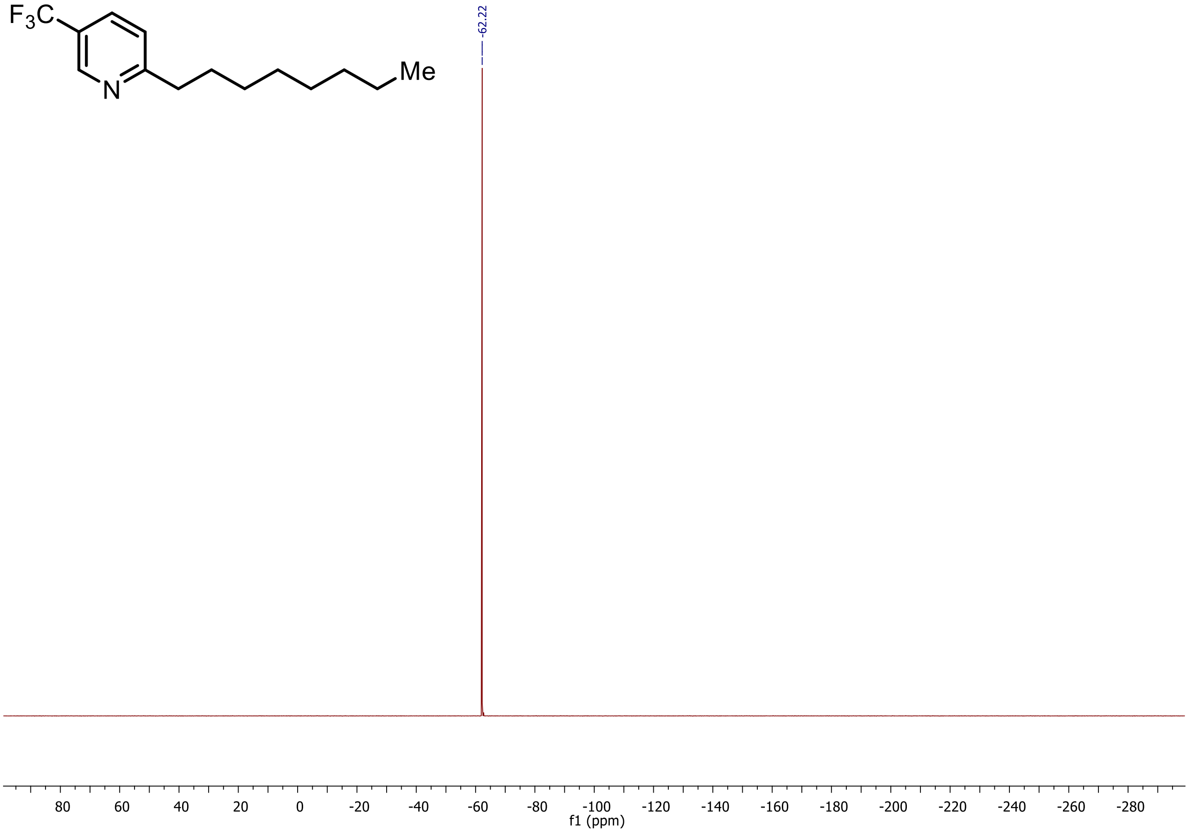

Figure S111. 5af, ^{1}H NMR, 500 MHz, $CDCl_3$

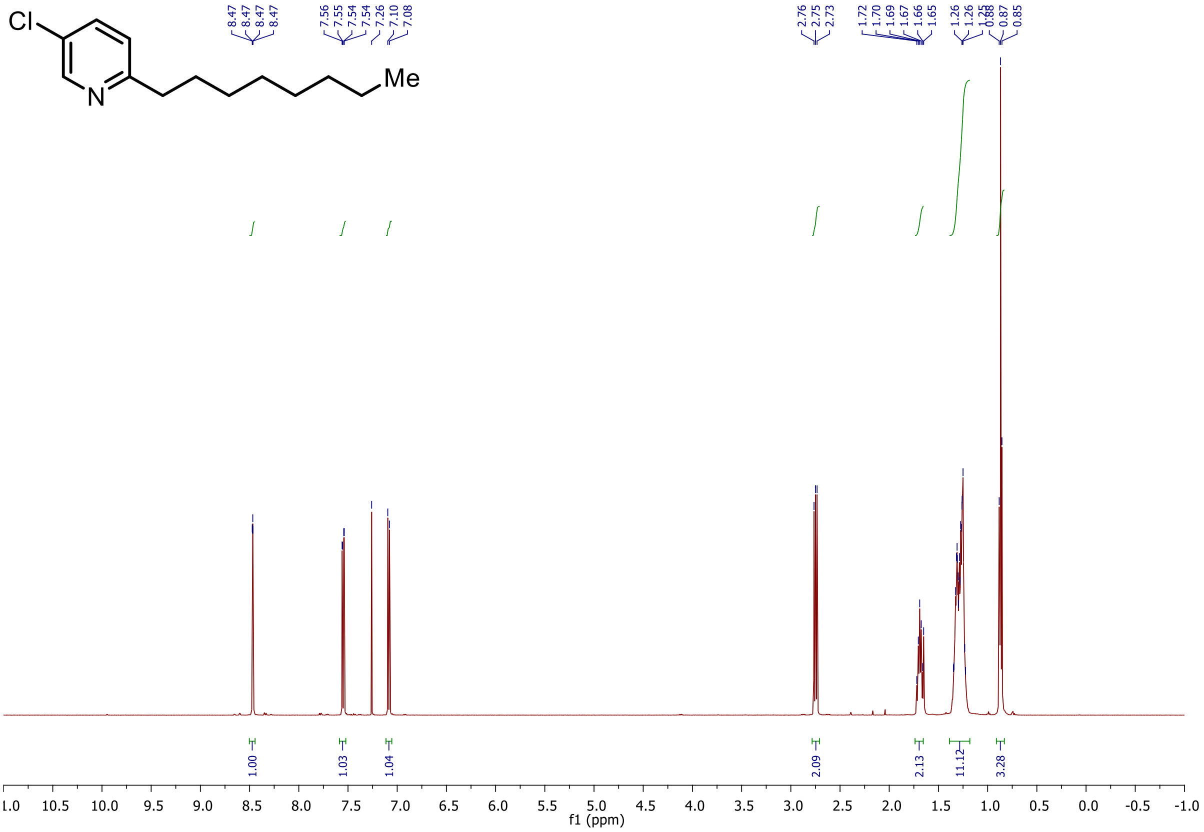

Figure S112. 5af, ^{13}C {^{1}H} NMR, 126 MHz, $CDCl_3$

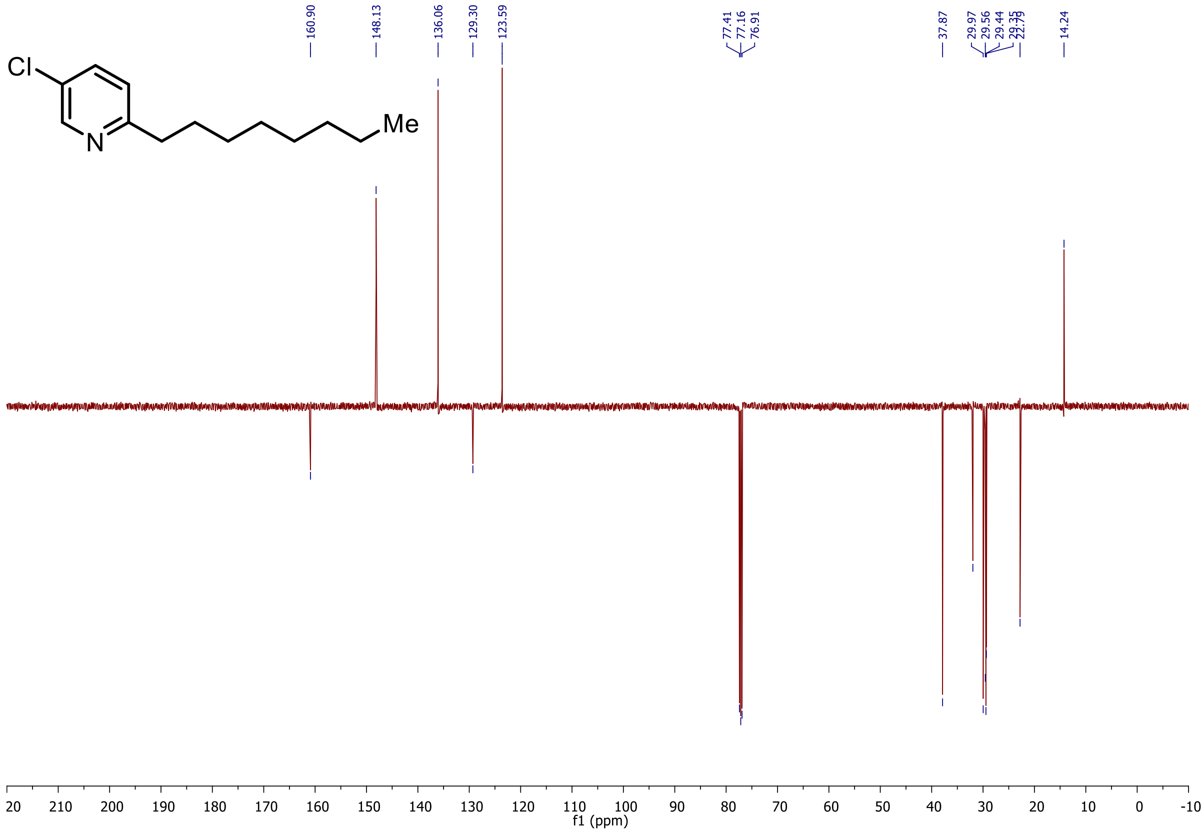

Figure S113. 5ag, ^{1}H NMR, 500 MHz, $CDCl_3$

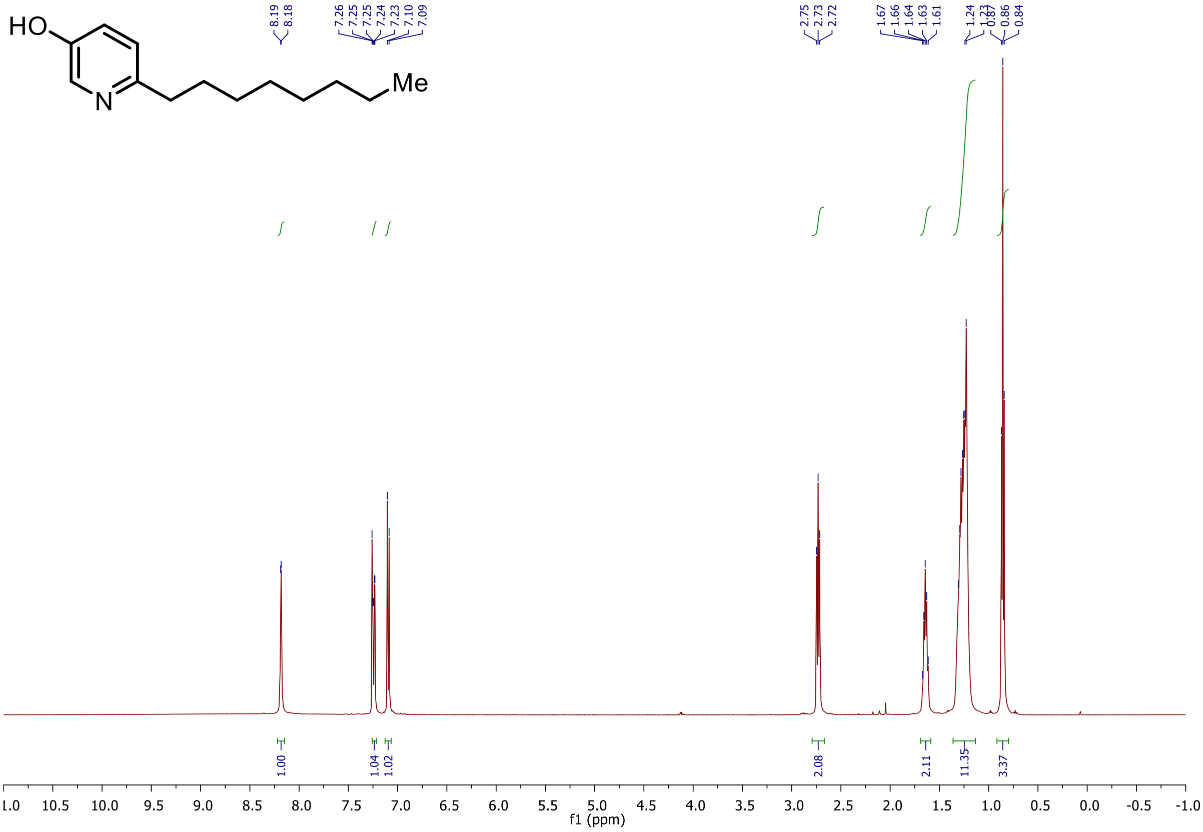

Figure S114. 5ag, ^{13}C {^{1}H} NMR, 126 MHz, $CDCl_3$

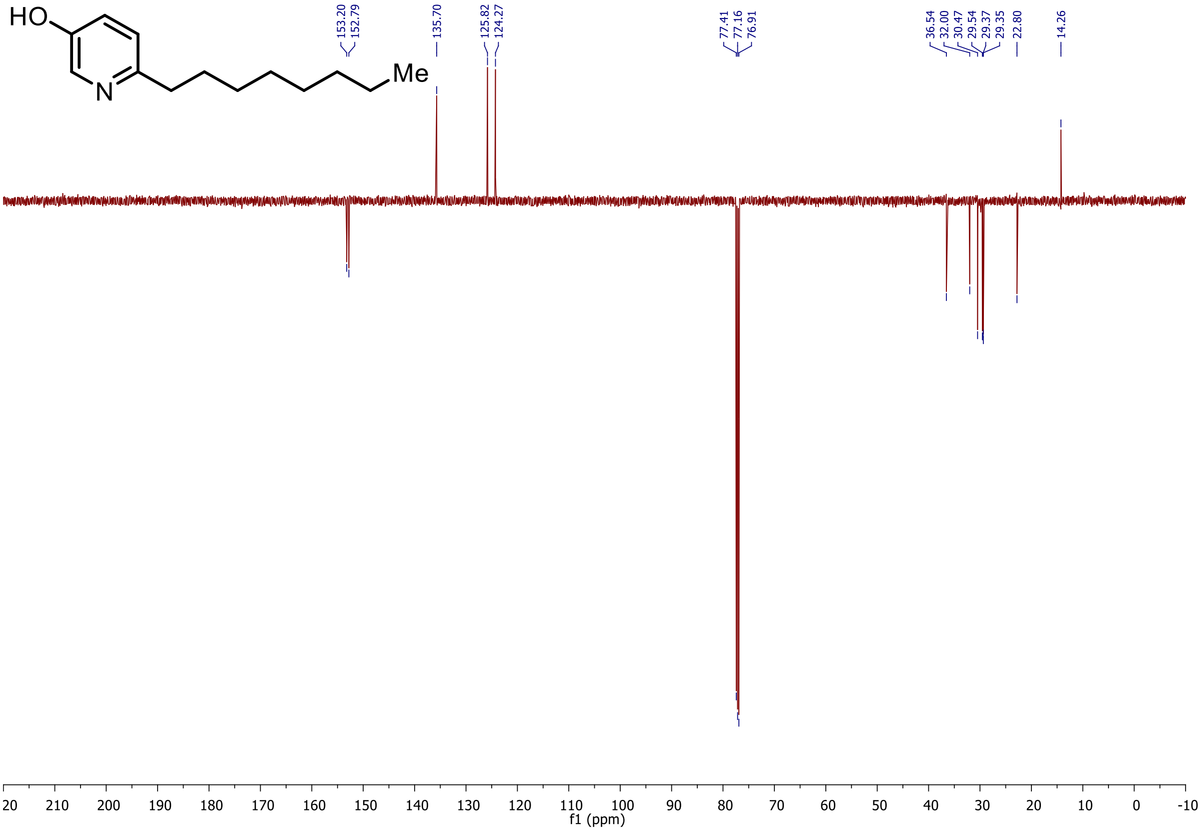

Figure S115. 5ah, ^{1}H NMR, 500 MHz, $CDCl_3$

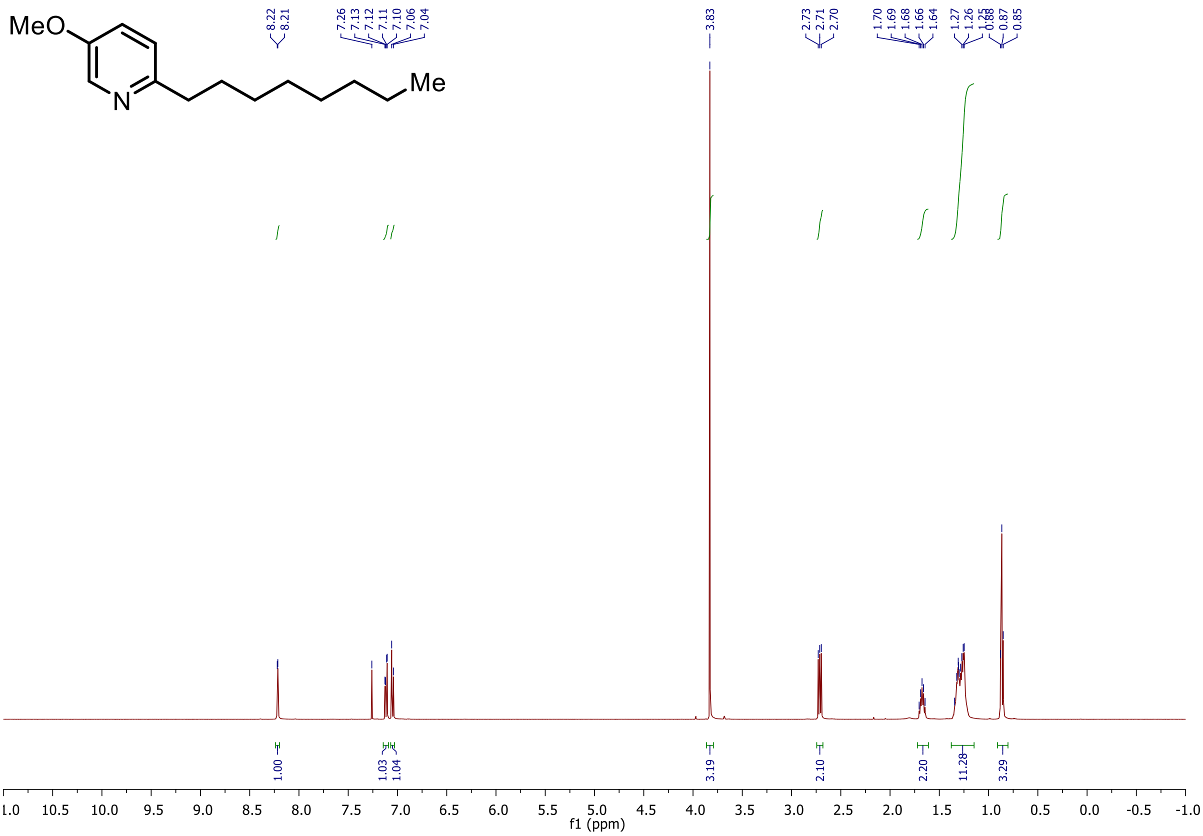

Figure S116. 5ah, $^{13}C\{^{1}H\}$ NMR, 126 MHz, $CDCl_3$

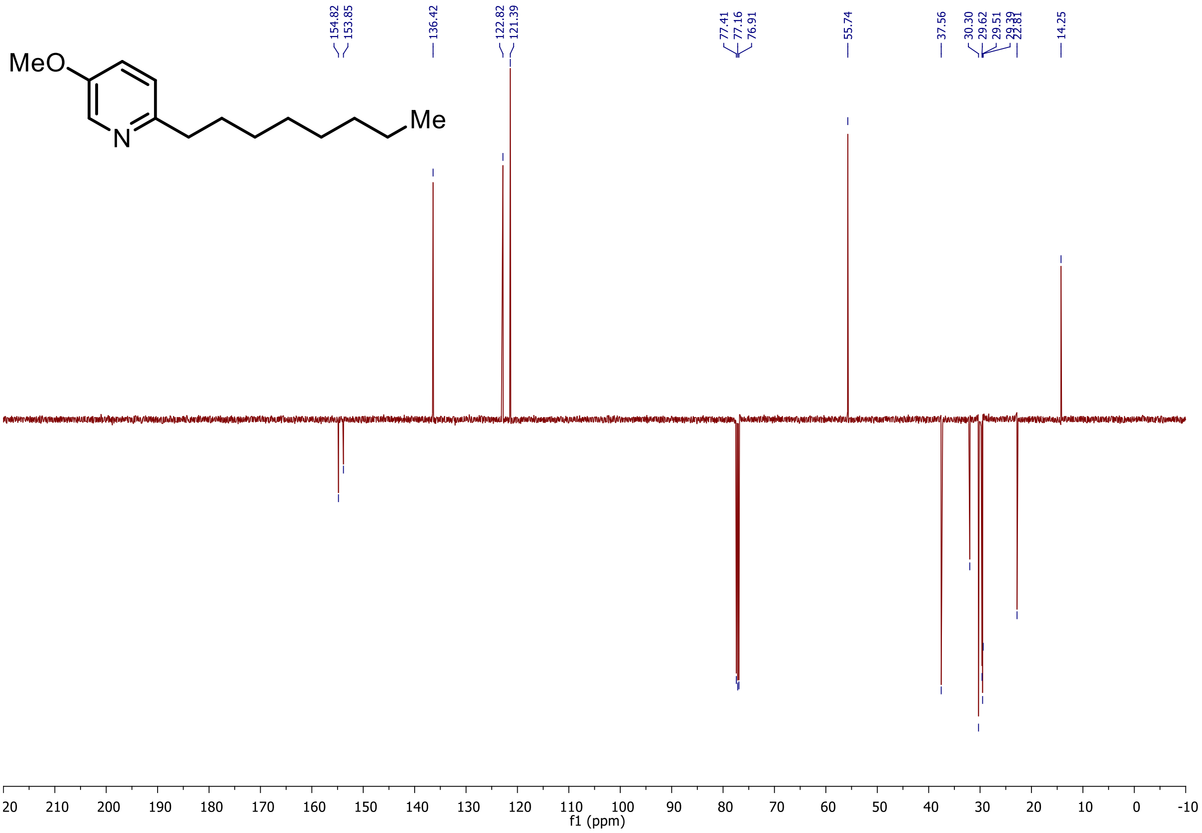

Figure S117. 5ai, ^{1}H NMR, 500 MHz, $CDCl_3$

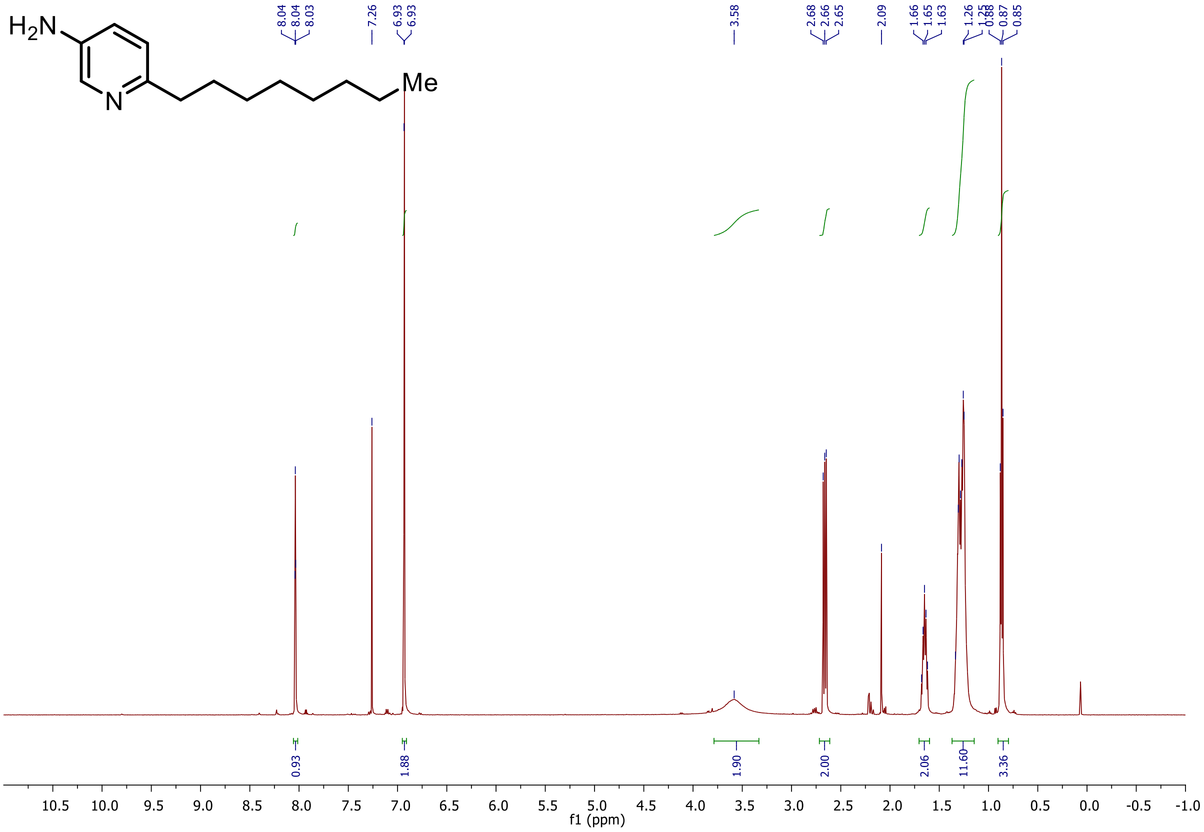

Figure S118. 5ai, ^{13}C {^{1}H} NMR, 126 MHz, $CDCl_3$

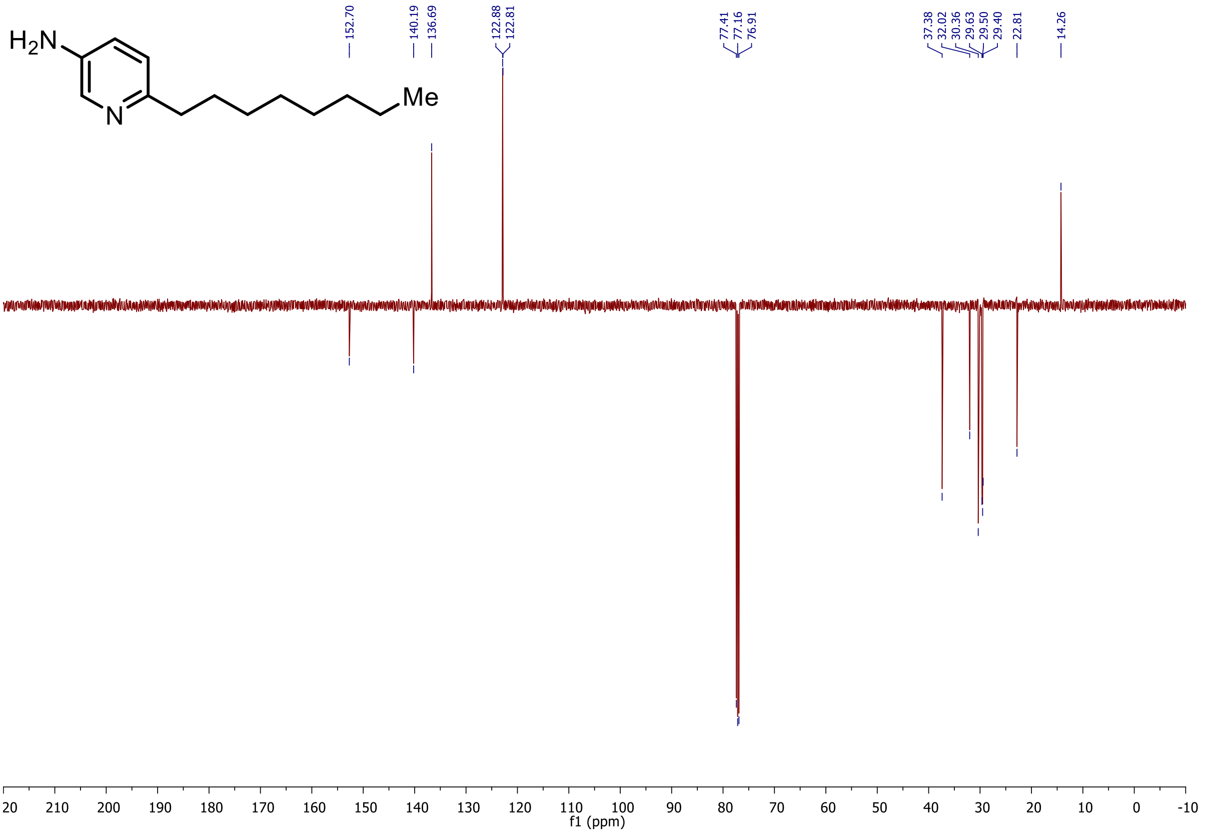

Figure S119. 5aj, ^{1}H NMR, 500 MHz, $CDCl_3$

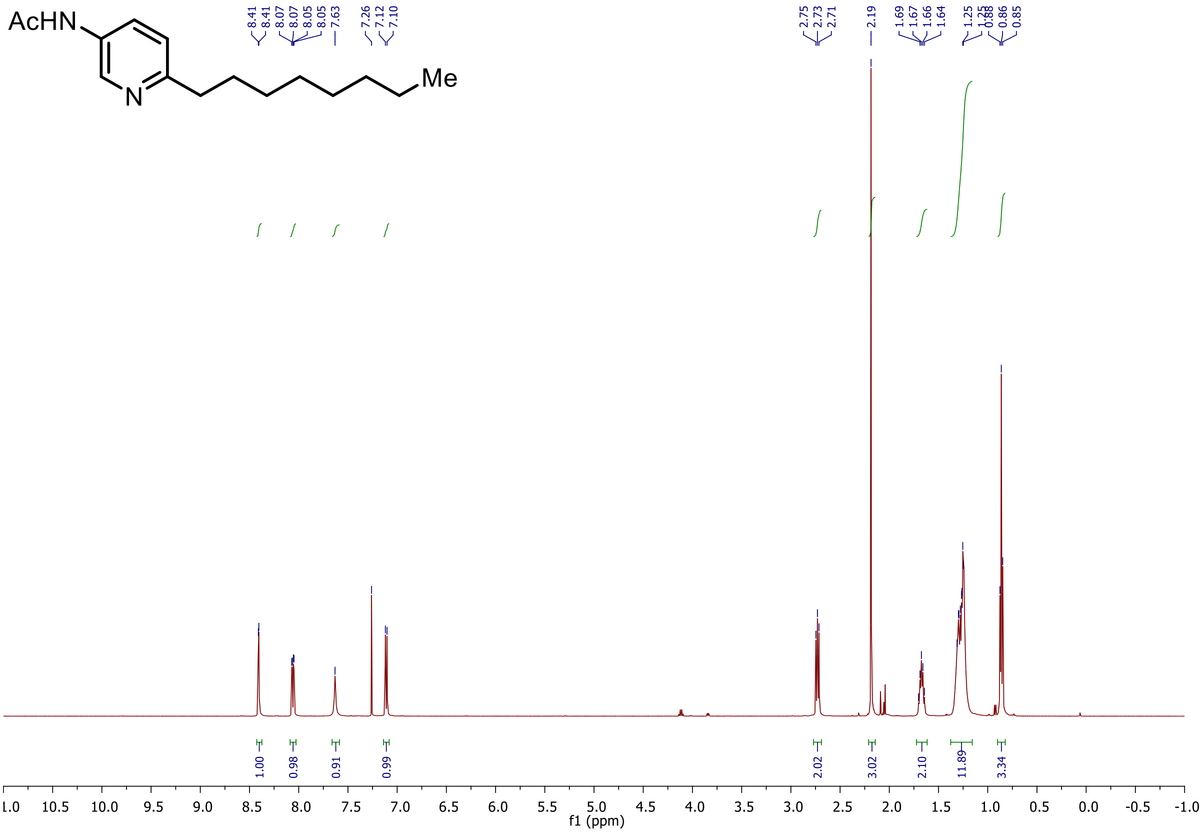

Figure S120. 5aj, ^{13}C {^{1}H} NMR, 126 MHz, $CDCl_3$

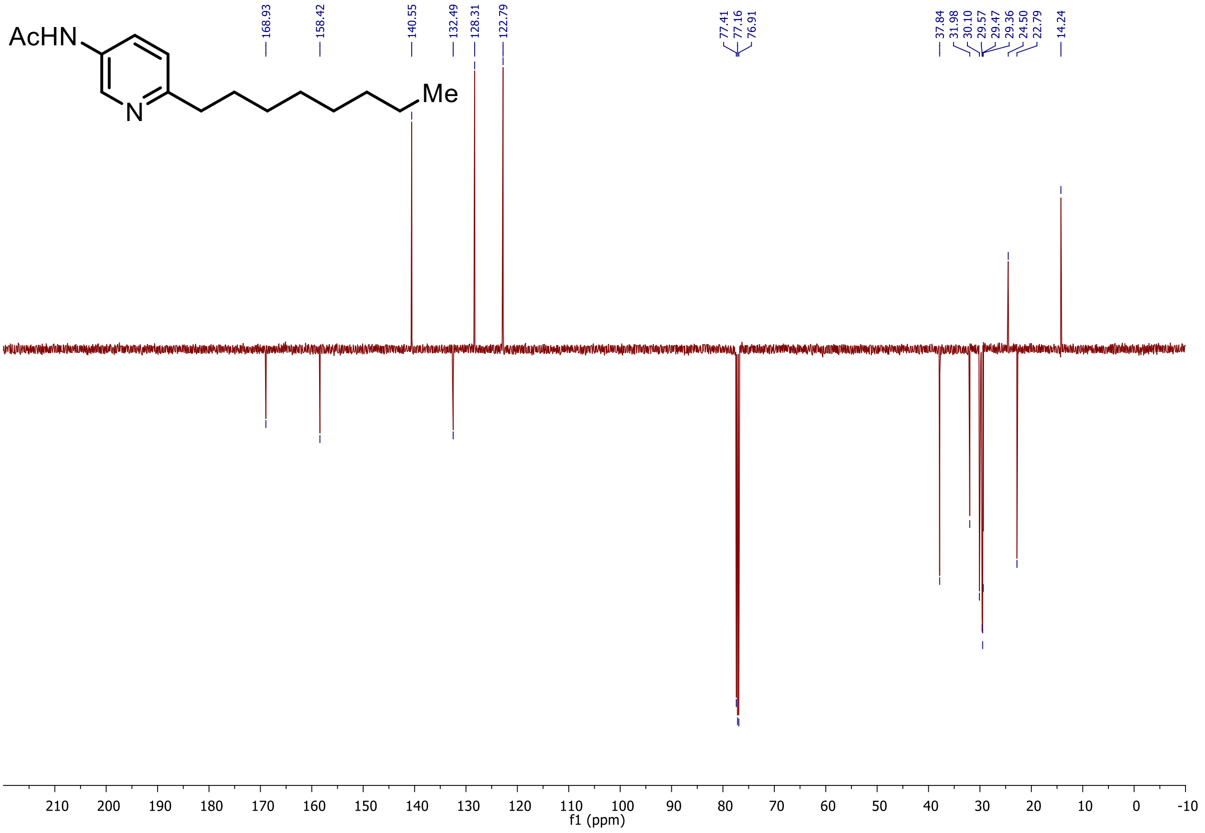

Figure S121. 5l, ^{1}H NMR, 500 MHz, $CDCl_3$

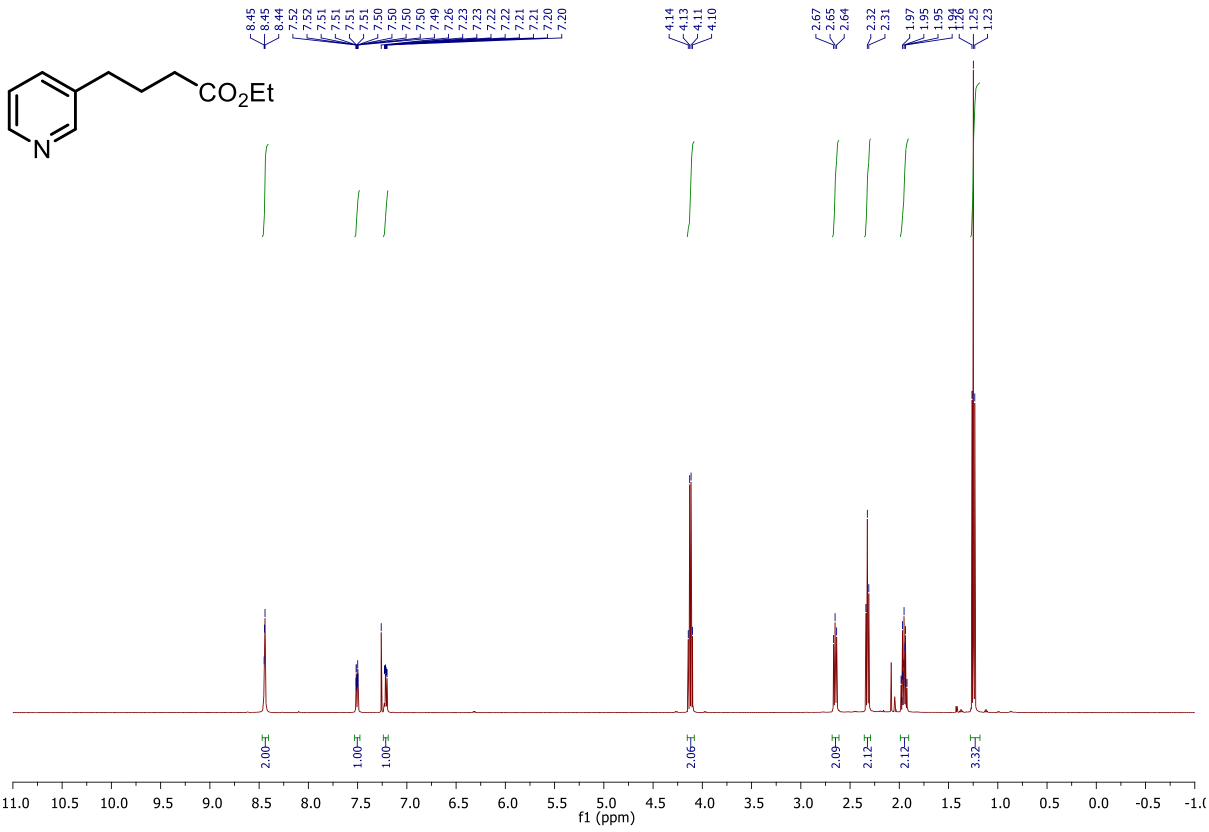

Figure S122. 5l, ^{13}C {^{1}H} NMR, 126 MHz, $CDCl_3$

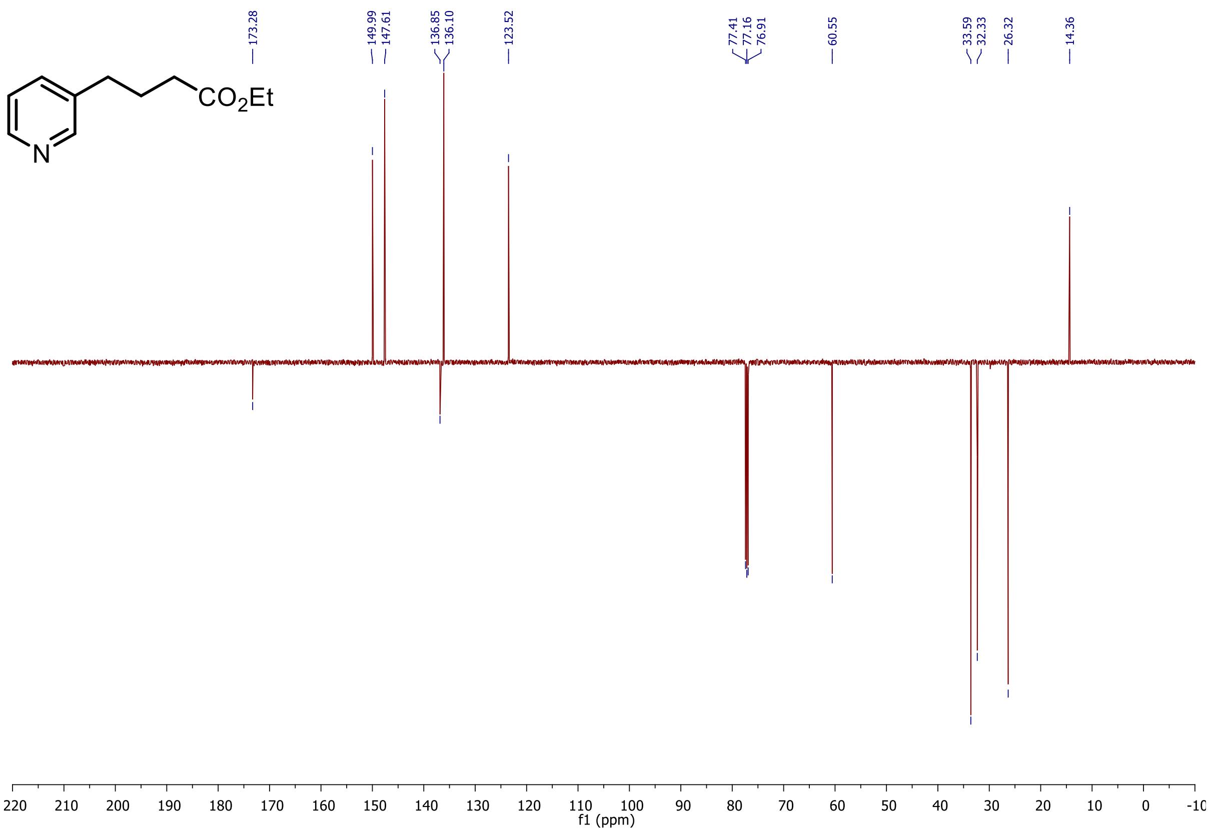

Figure S123. 5m, ^{1}H NMR, 500 MHz, $CDCl_3$

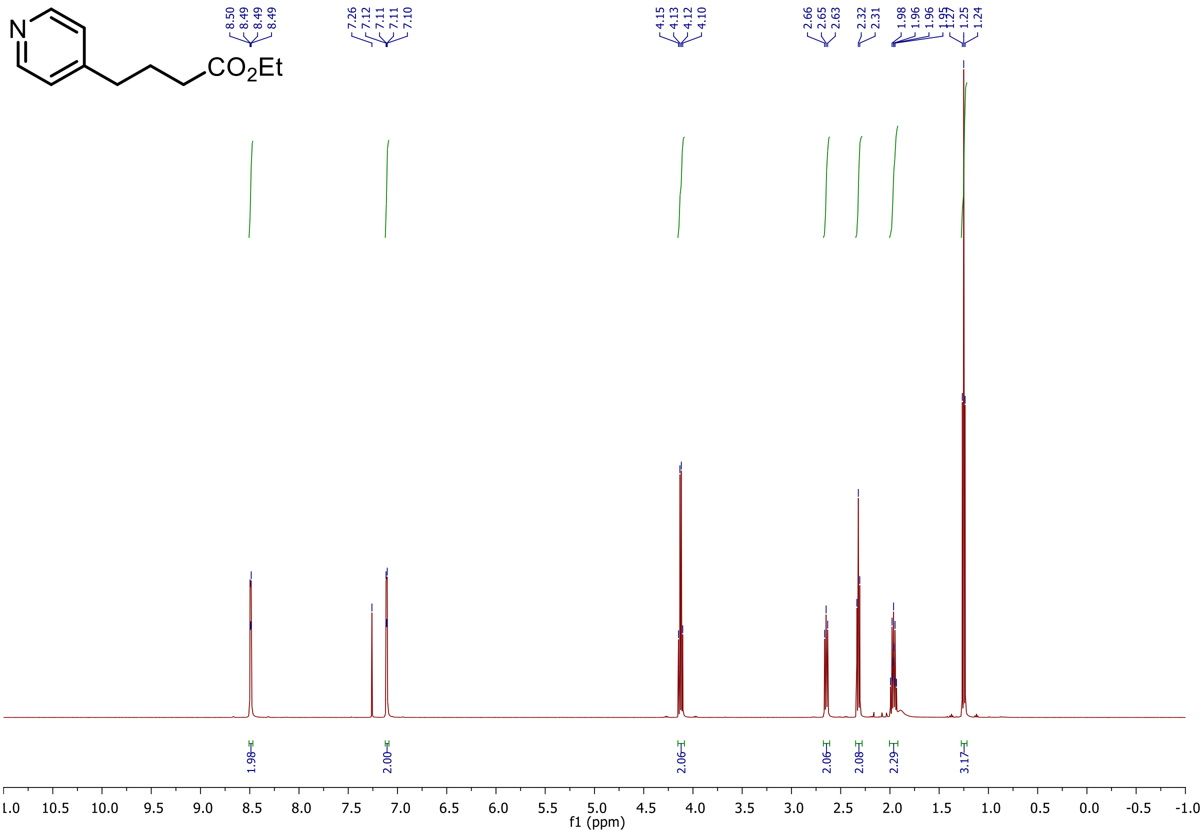

Figure S124. 5m, ^{13}C {^{1}H} NMR, 126 MHz, $CDCl_3$

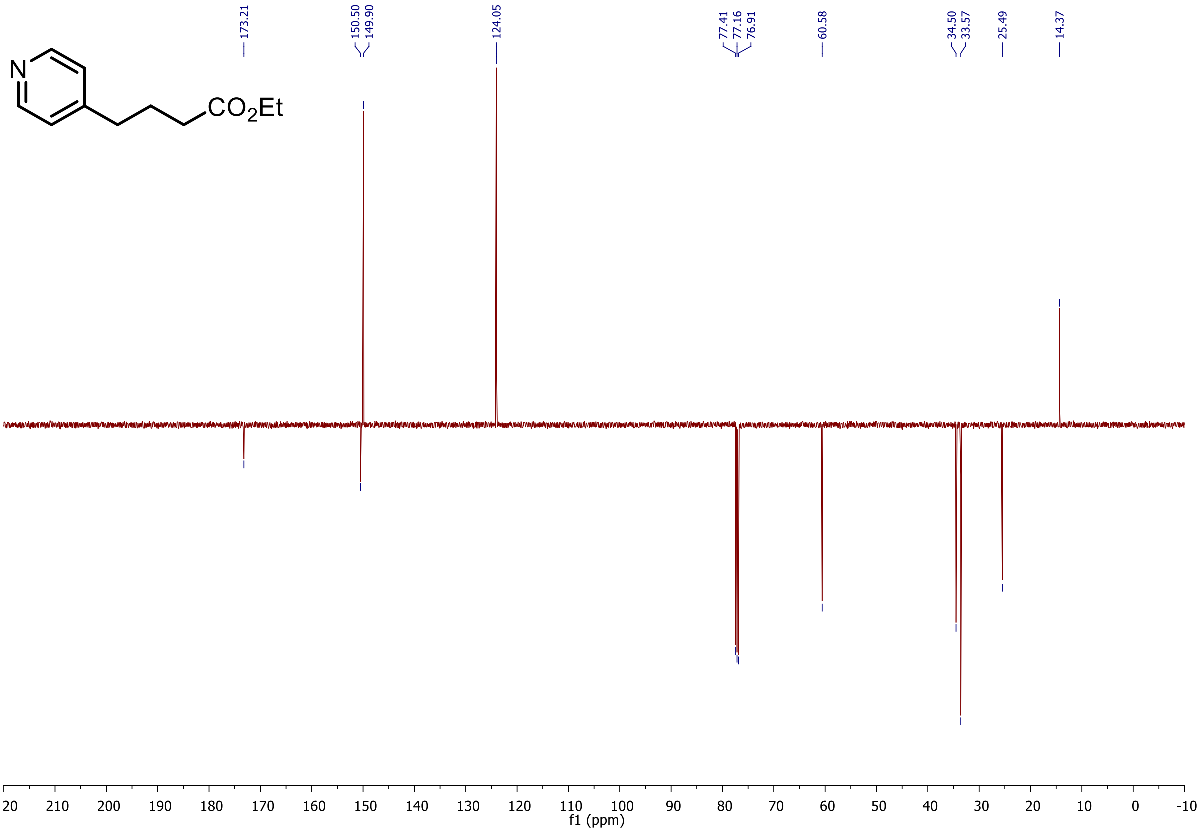

Figure S125. 5n, ^{1}H NMR, 400 MHz, $CDCl_3$

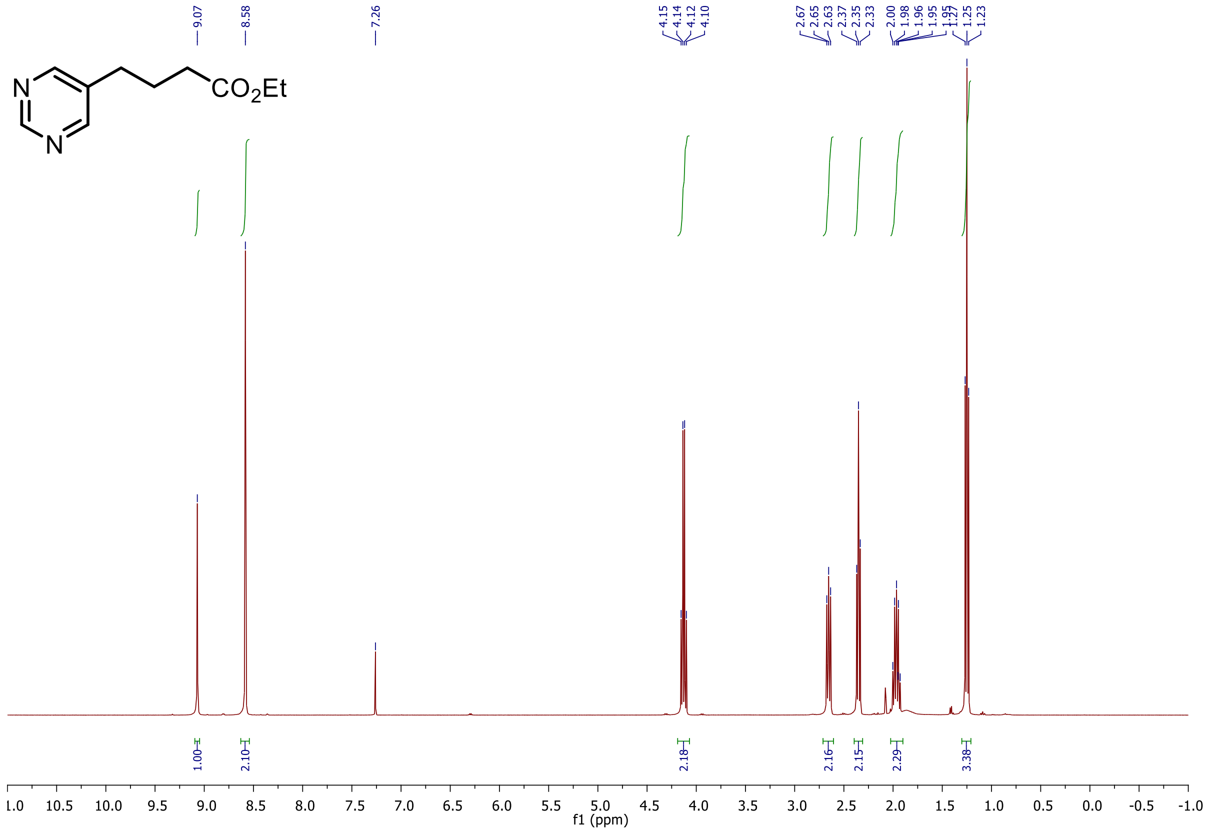

Figure S126. 5n, $^{13}C\ \{^1H\}$ NMR, 101 MHz, $CDCl_3$

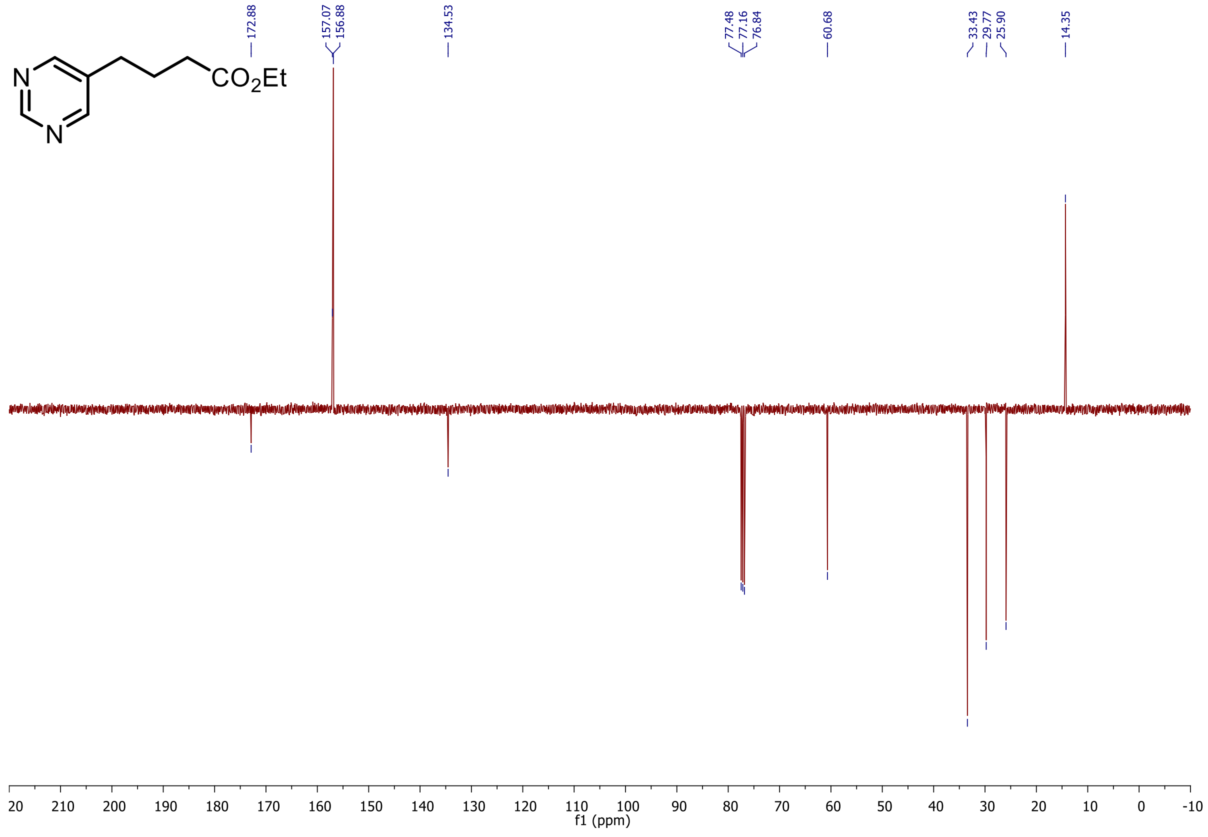

Figure S127. 5o, ^{1}H NMR, 500 MHz, $CDCl_3$

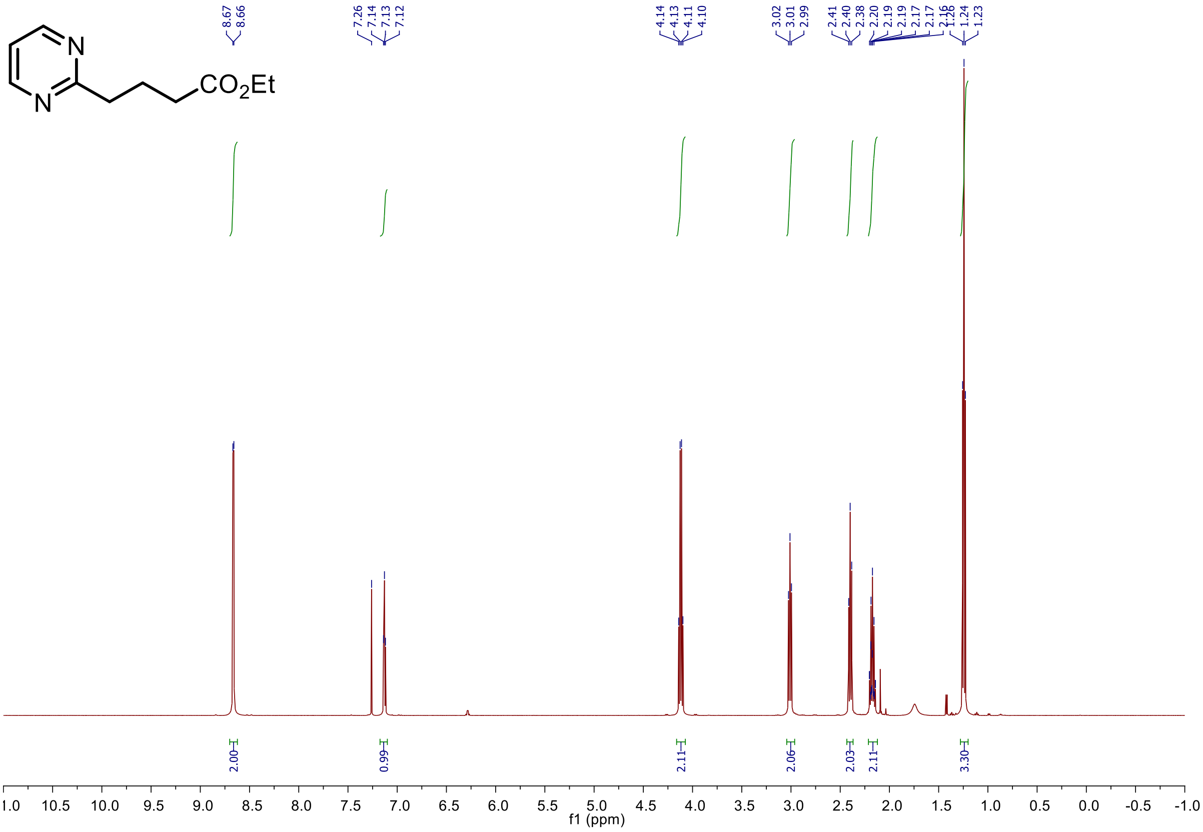

Figure S128. 5o, ^{13}C {^{1}H} NMR, 126 MHz, $CDCl_3$

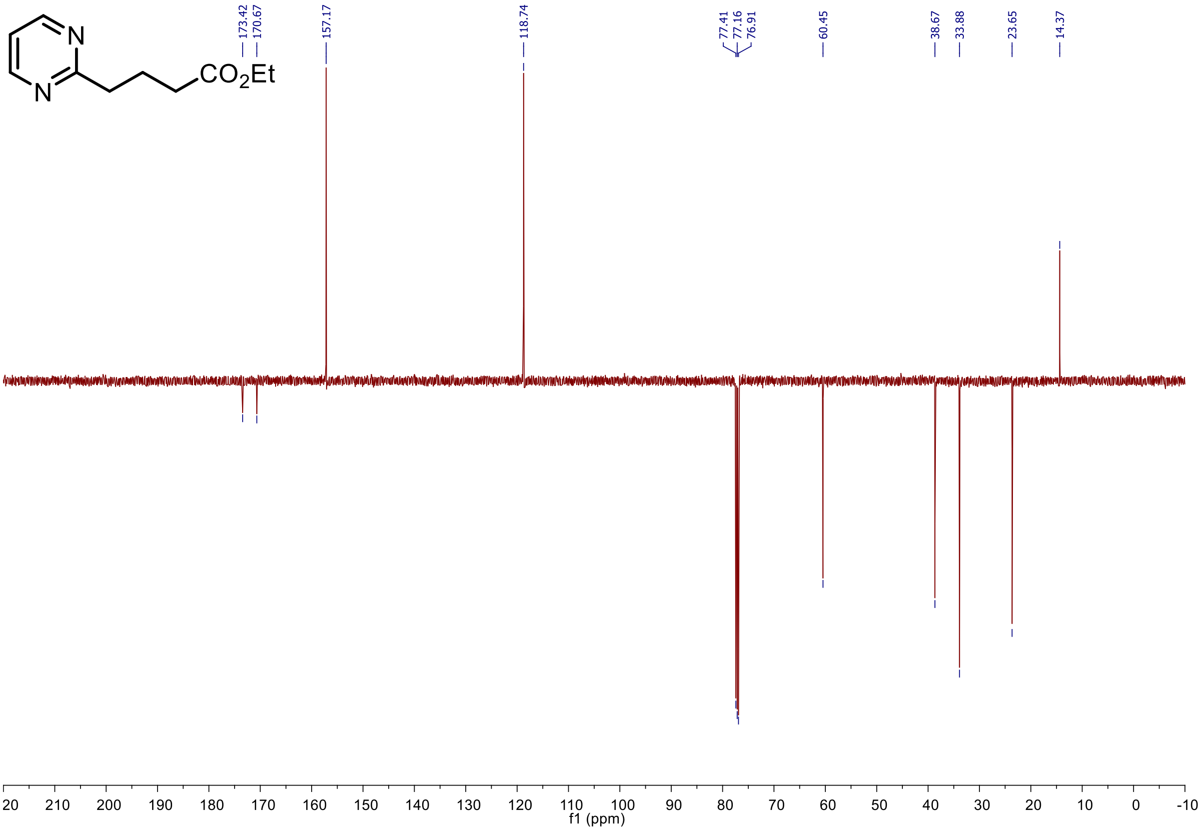

Figure S129. 5p, ^{1}H NMR, 400 MHz, $CDCl_3$

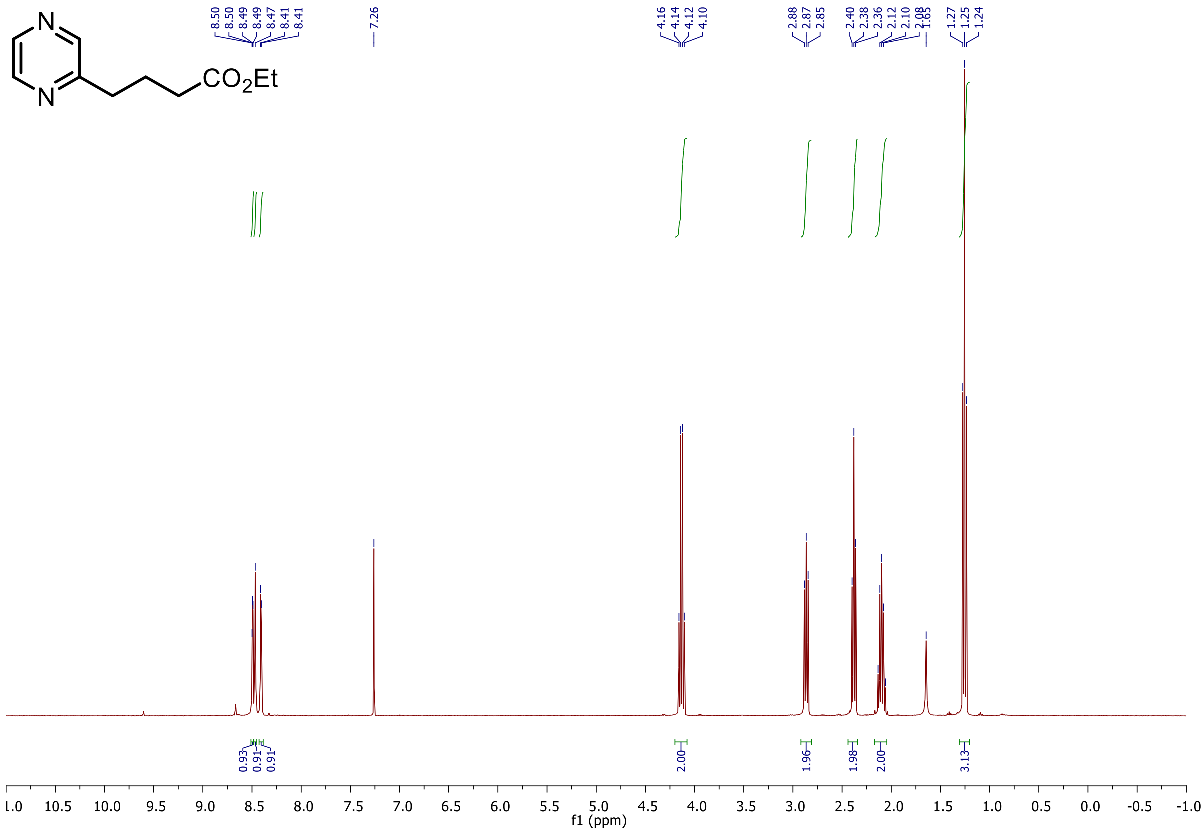

Figure S130. 5p, $^{13}C\{^{1}H\}$ NMR, 101 MHz, $CDCl_3$

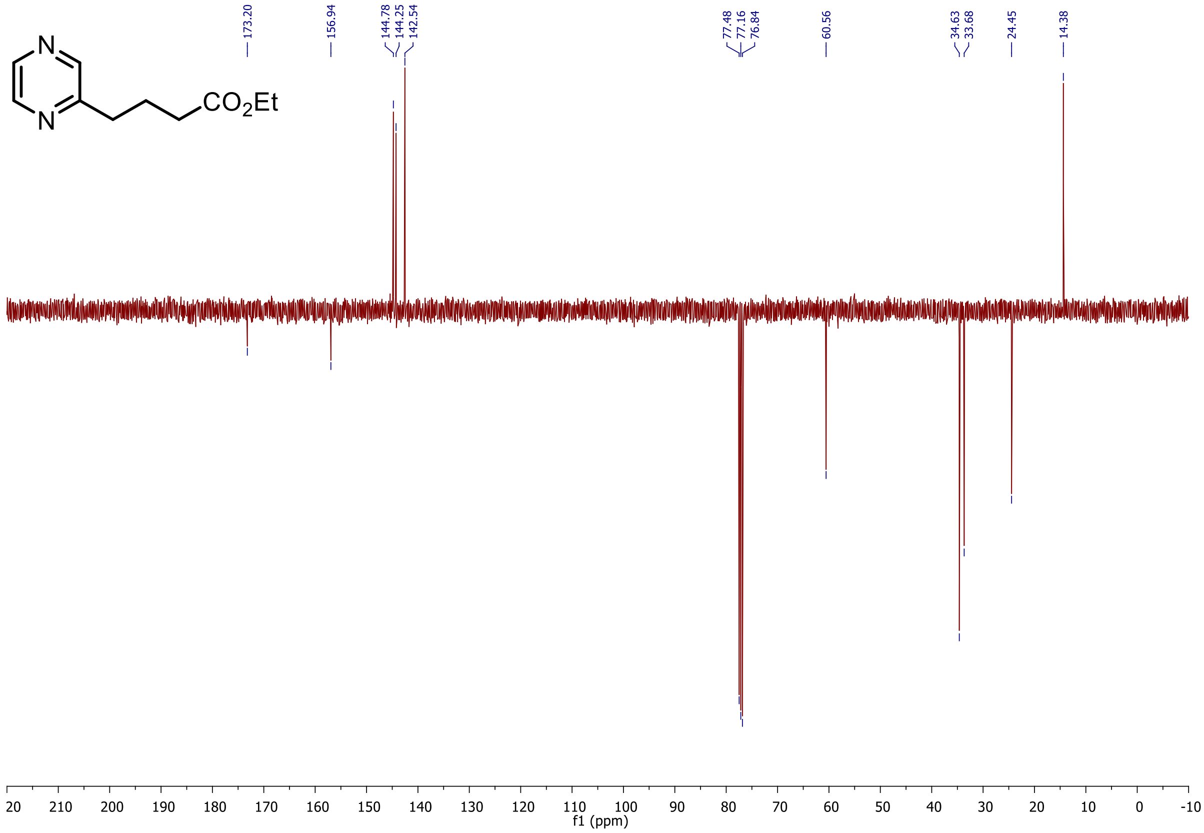

Figure S132. 5q, ^{1}H NMR, 500 MHz, $CDCl_3$

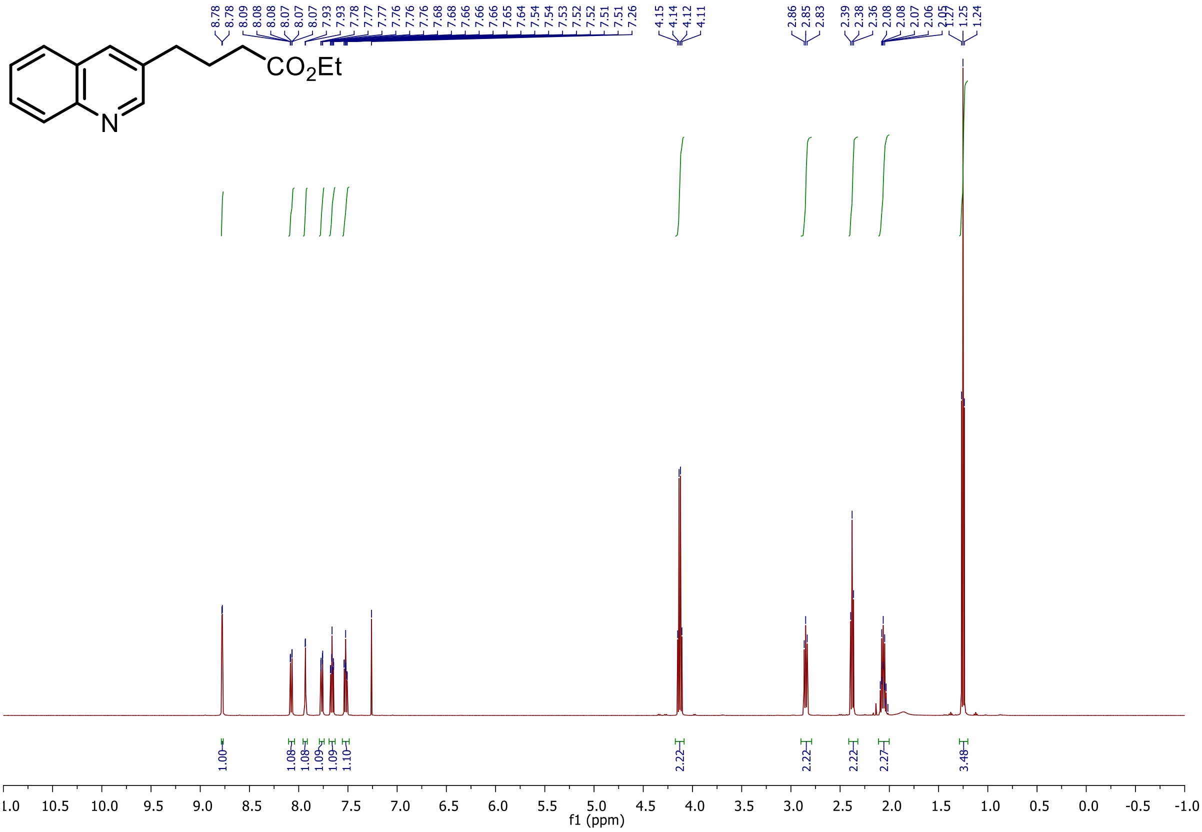

Figure S132. 5q, ^{13}C {^{1}H} NMR, 126 MHz, $CDCl_3$

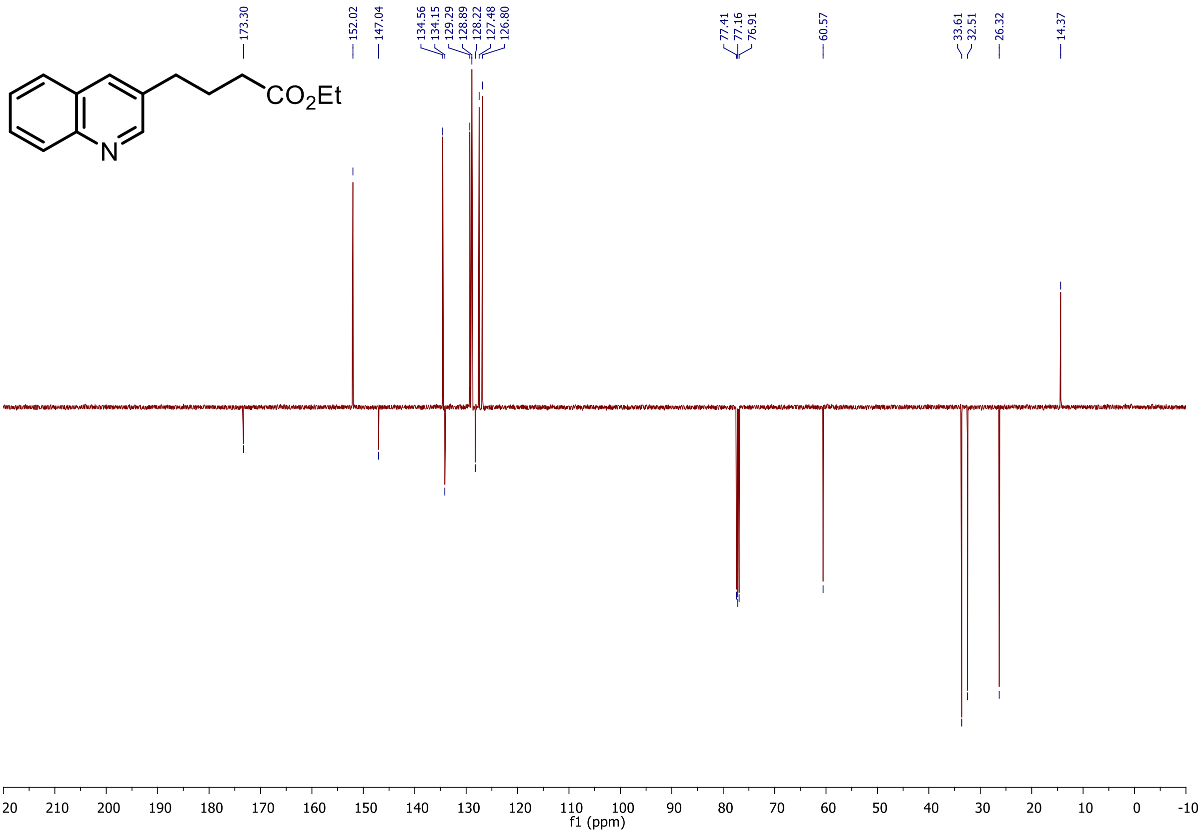

Figure S133. 5r, ^{1}H NMR, 500 MHz, $CDCl_3$

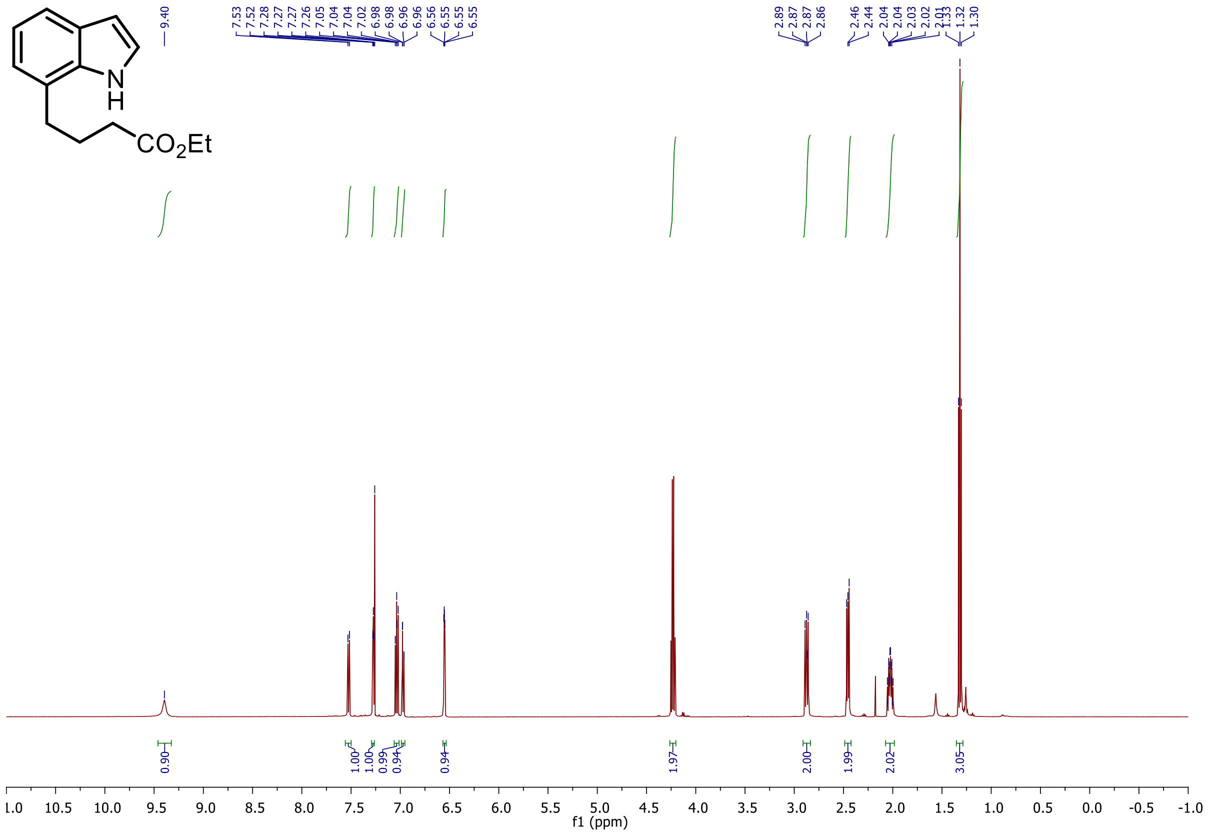

Figure S134. 5r, ^{13}C {^{1}H} NMR, 126 MHz, $CDCl_3$

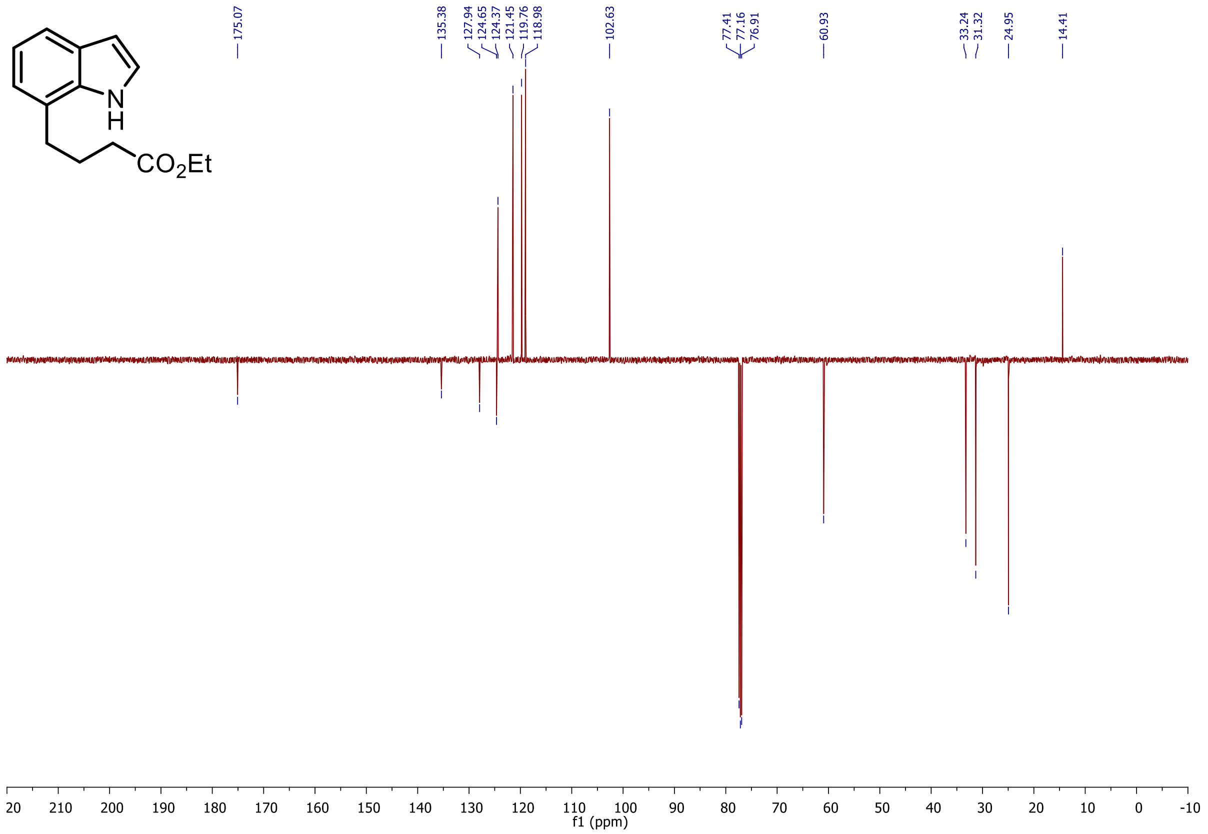

Figure S135. 5b, ^{1}H NMR, 500 MHz, $CDCl_3$

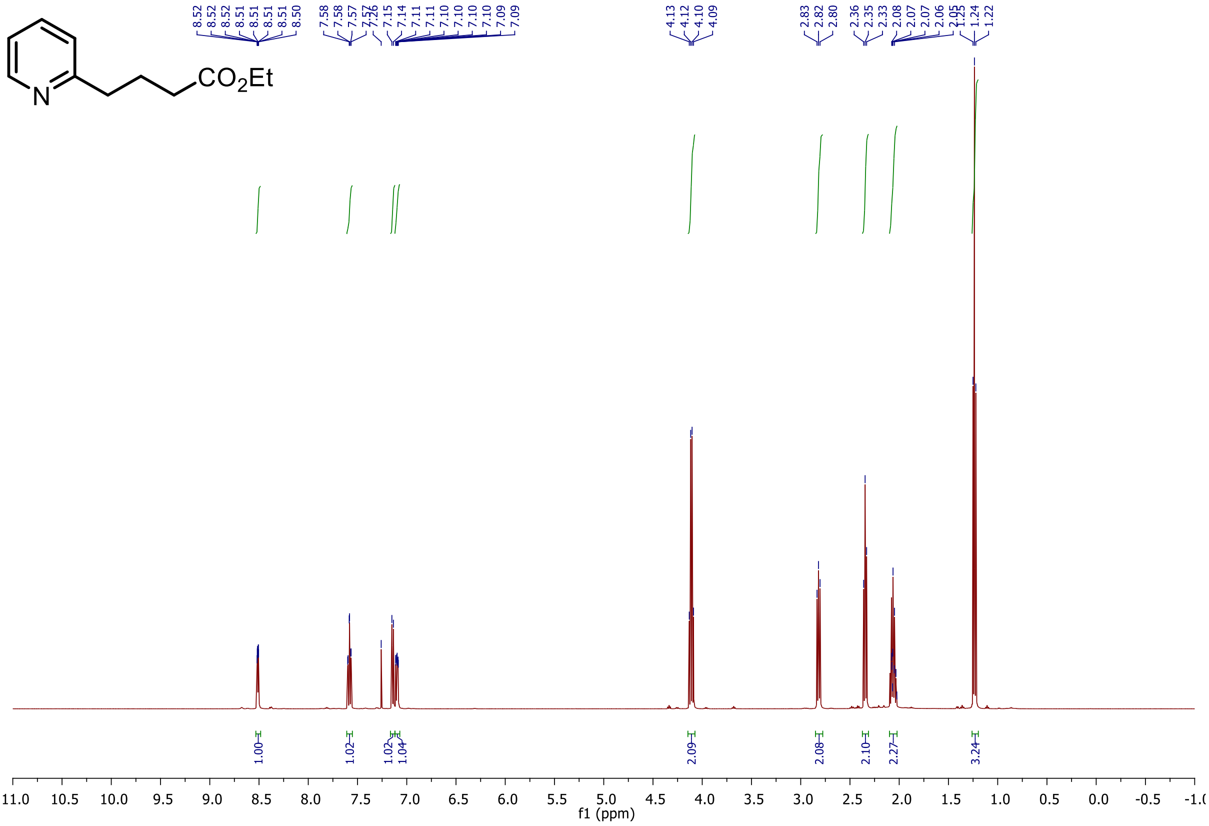

Figure S136. 5b, ^{13}C {^{1}H} NMR, 126 MHz, $CDCl_3$

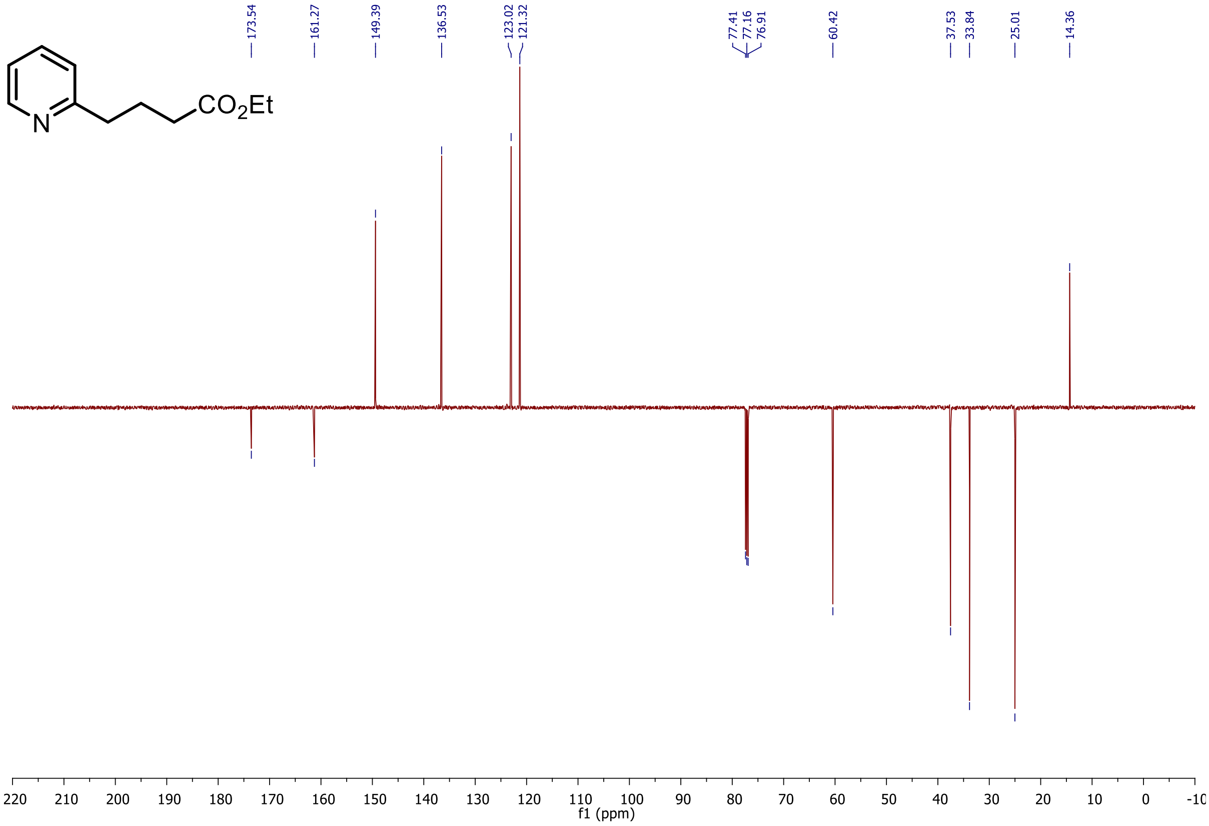

Figure S137. 5c, ^{1}H NMR, 500 MHz, $CDCl_3$

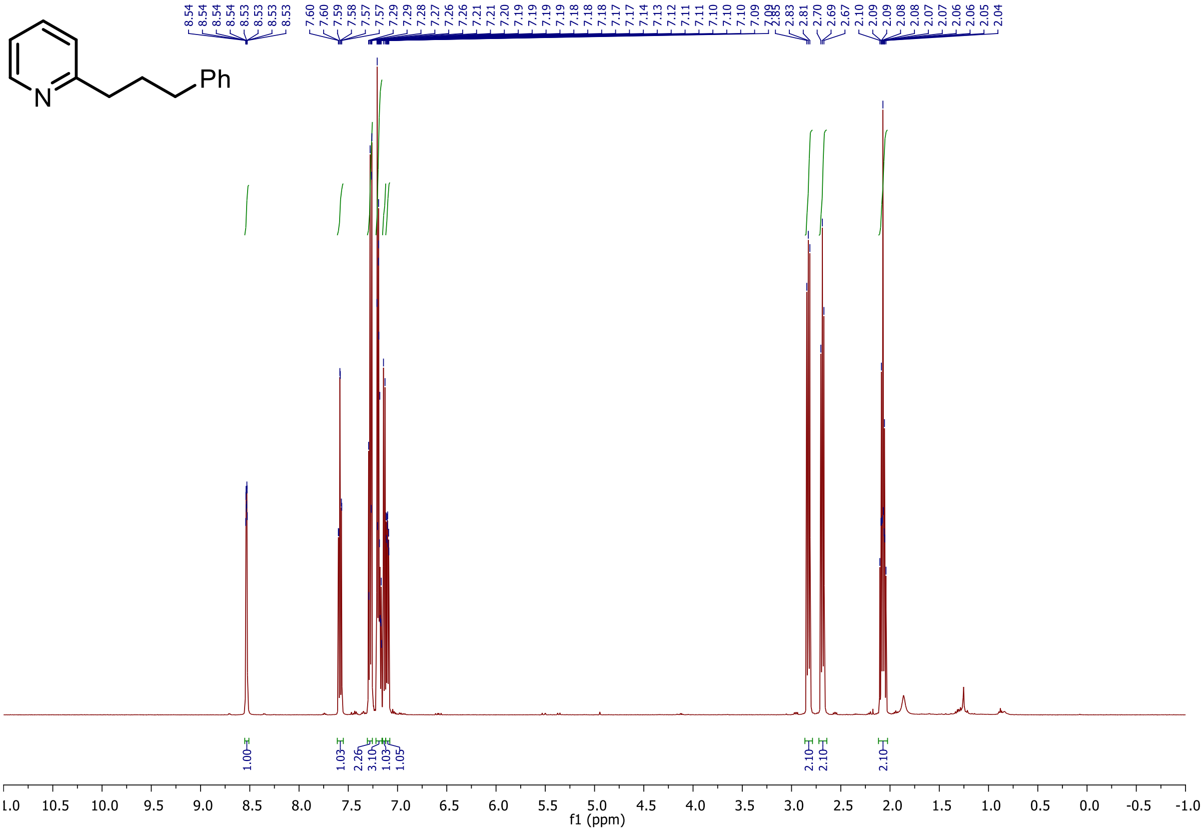

Figure S138. 5c, ^{13}C {^{1}H} NMR, 126 MHz, $CDCl_3$

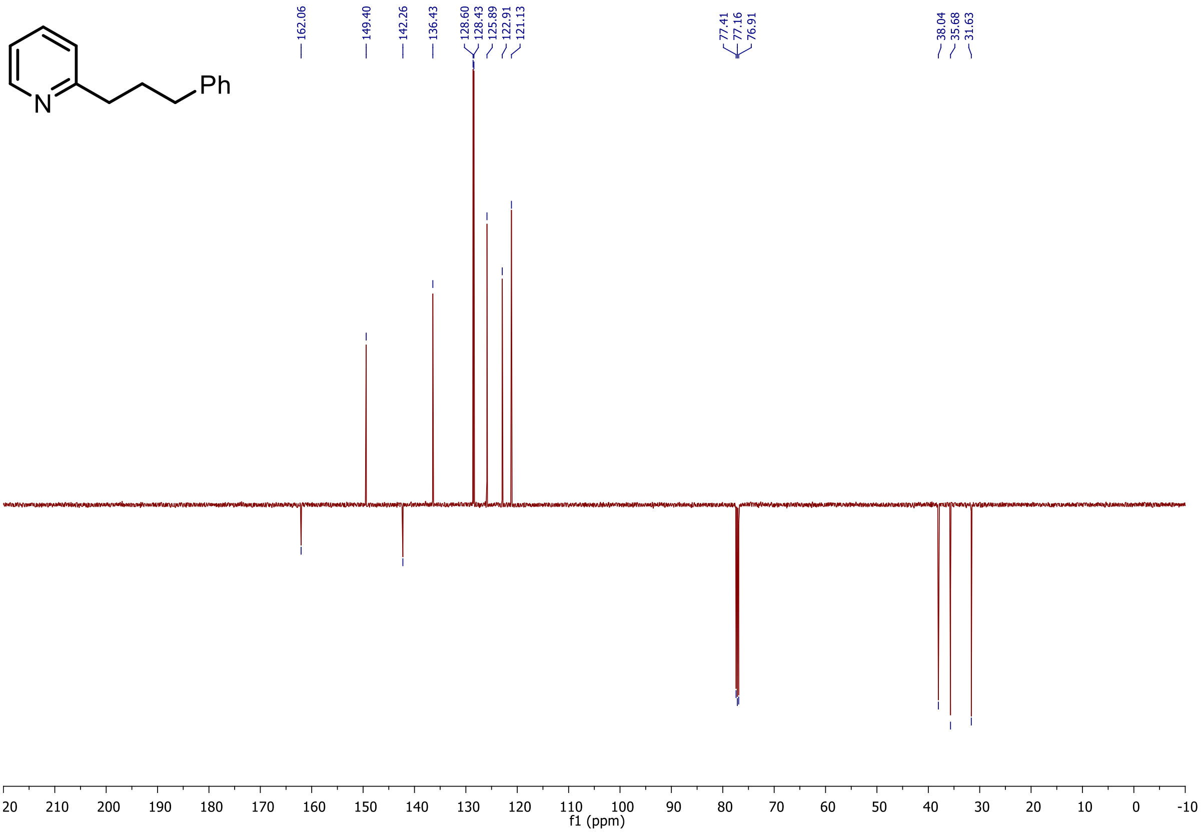

Figure S139. 5d, ^{1}H NMR, 500 MHz, $CDCl_3$

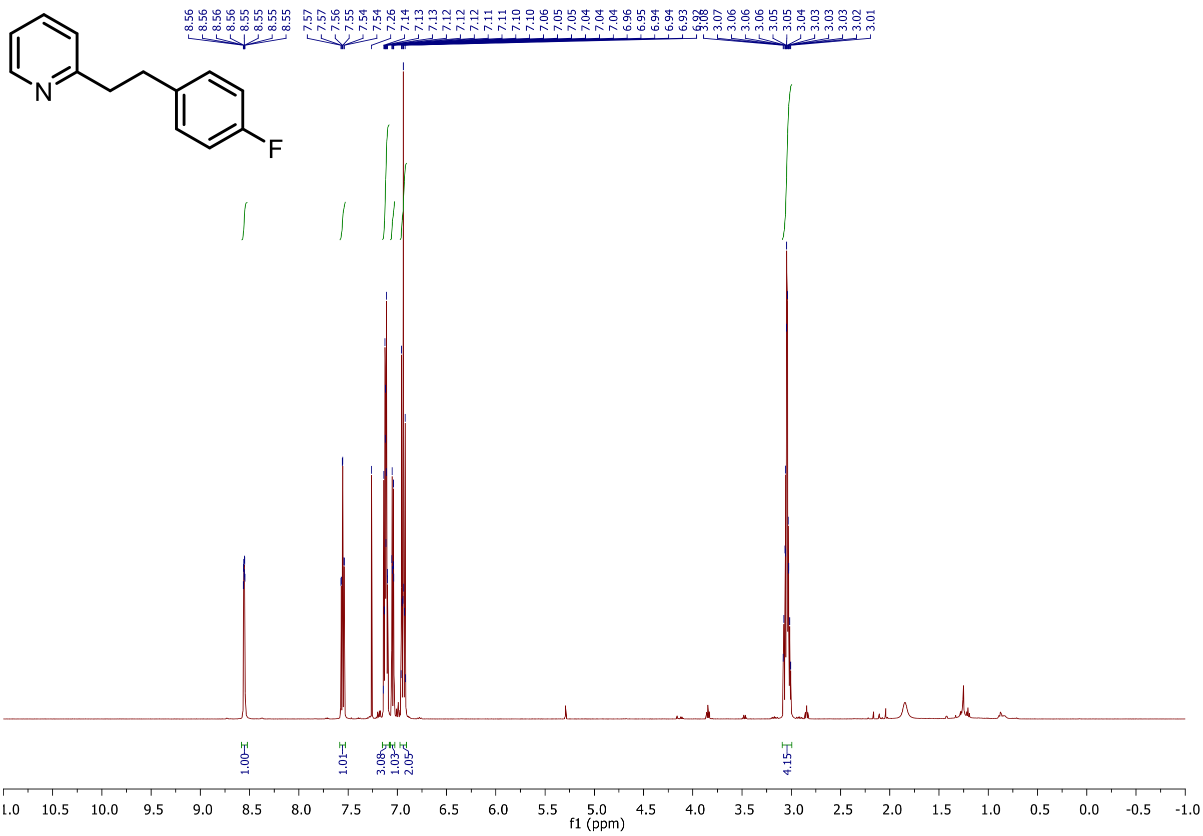

Figure S140. 5d, ^{13}C {^{1}H} NMR, 126 MHz, $CDCl_3$

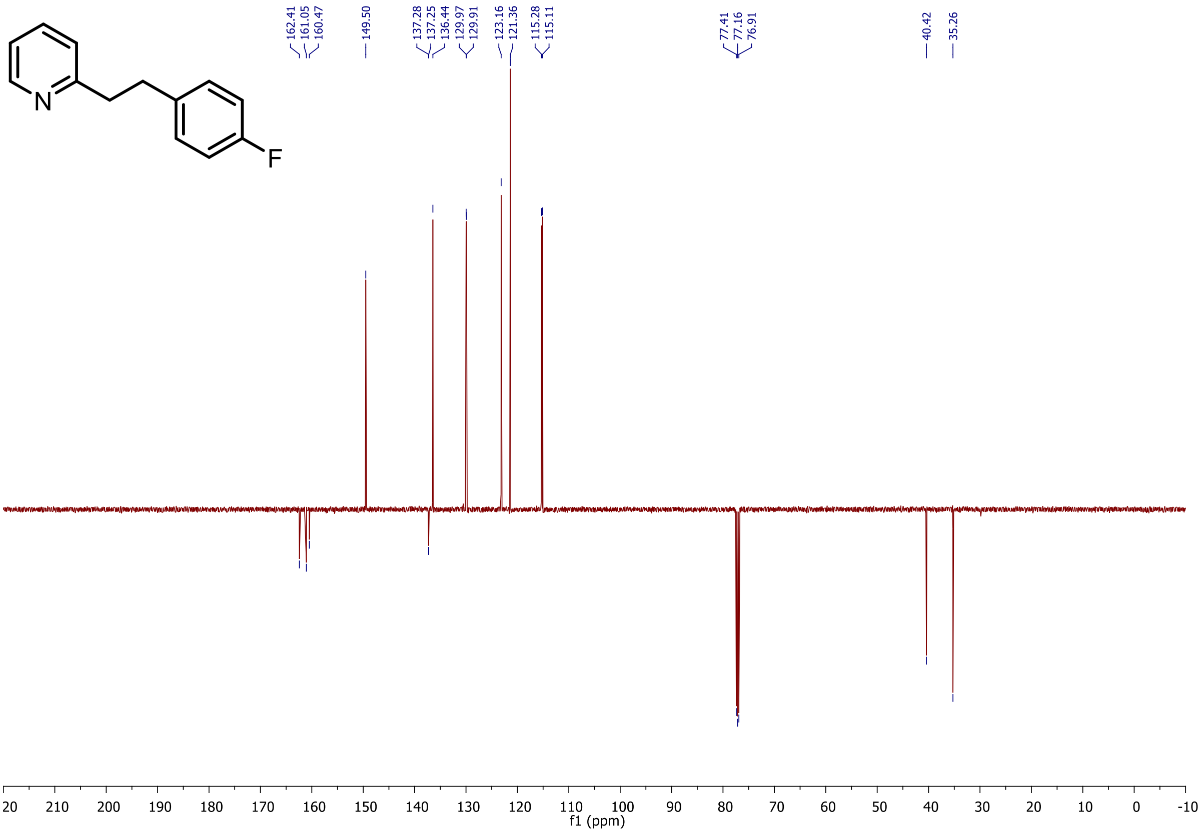

Figure S141. 5d, ^{19}F {^{1}H} NMR, 376 MHz, CDCl$_3$

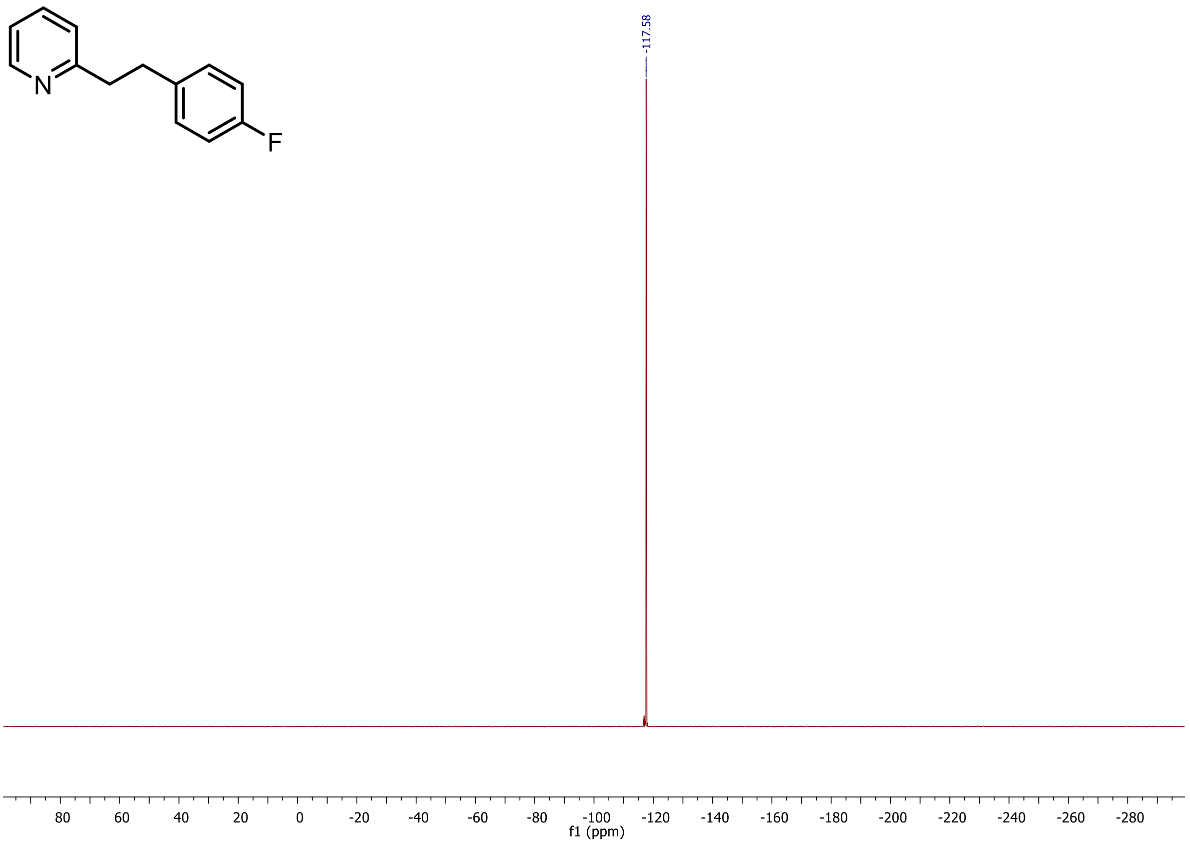

Figure S142. 5e, ^{1}H NMR, 500 MHz, CDCl$_3$

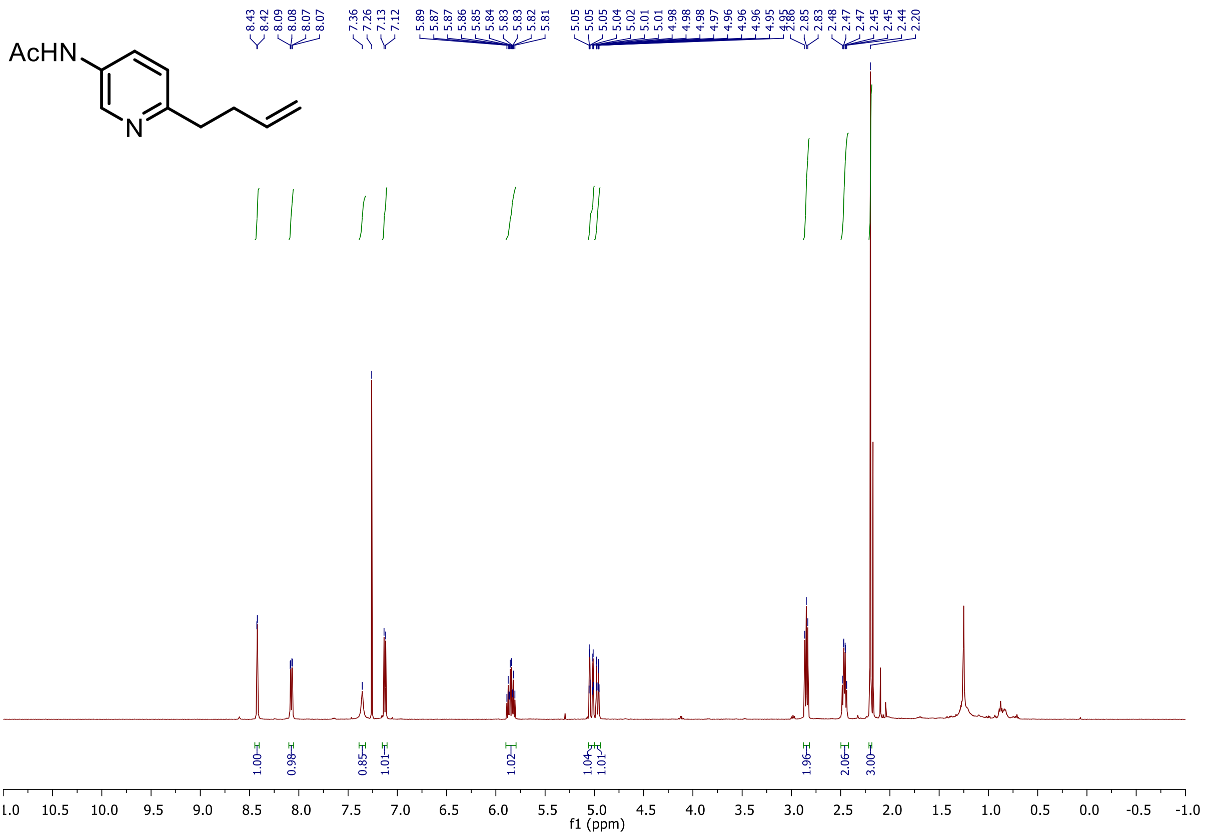

Figure S143. 5e, ^{13}C {^{1}H} NMR, 126 MHz, $CDCl_3$

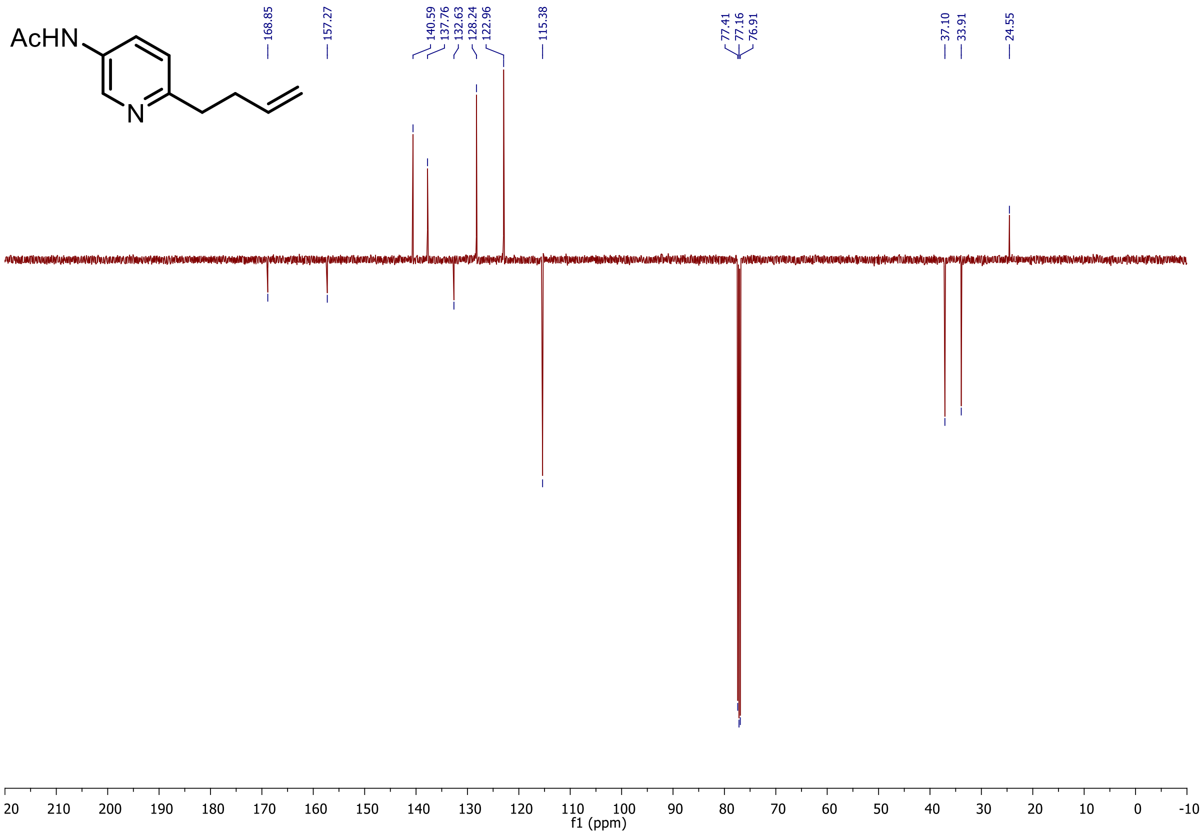

Figure S144. 5f, ^{1}H NMR, 500 MHz, $CDCl_3$

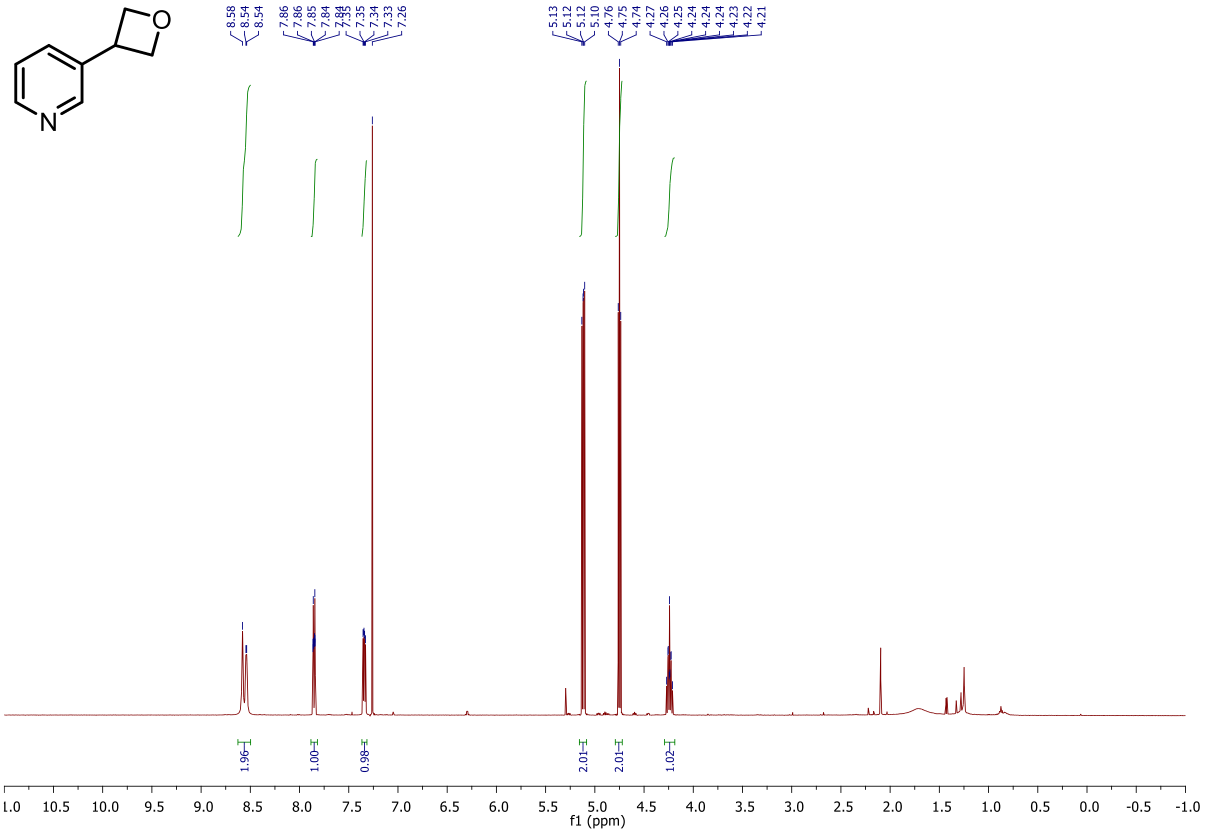

Figure S145. 5f, ^{13}C {^{1}H} NMR, 126 MHz, $CDCl_3$

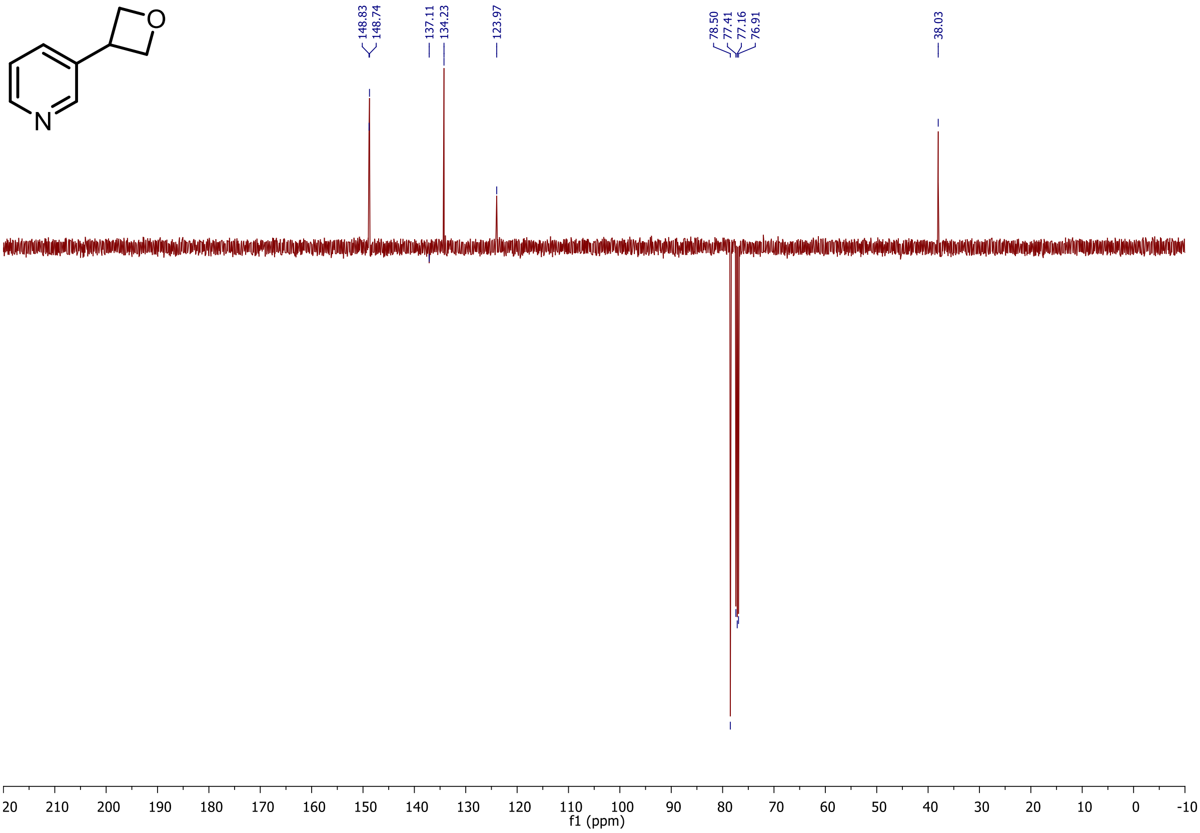

Figure S146. 5g, ^{1}H NMR, 400 MHz, $CDCl_3$

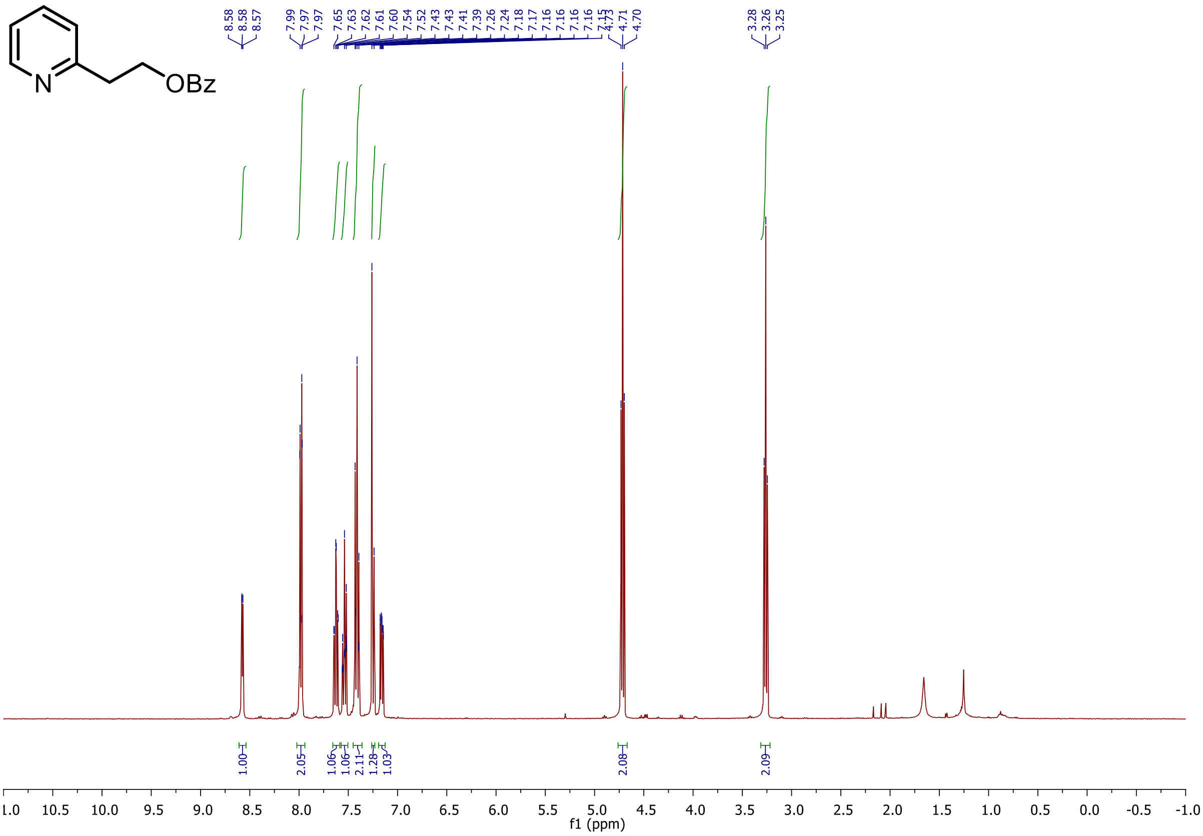

Figure S147. 5g, $^{13}C\{^{1}H\}$ NMR, 101 MHz, $CDCl_3$

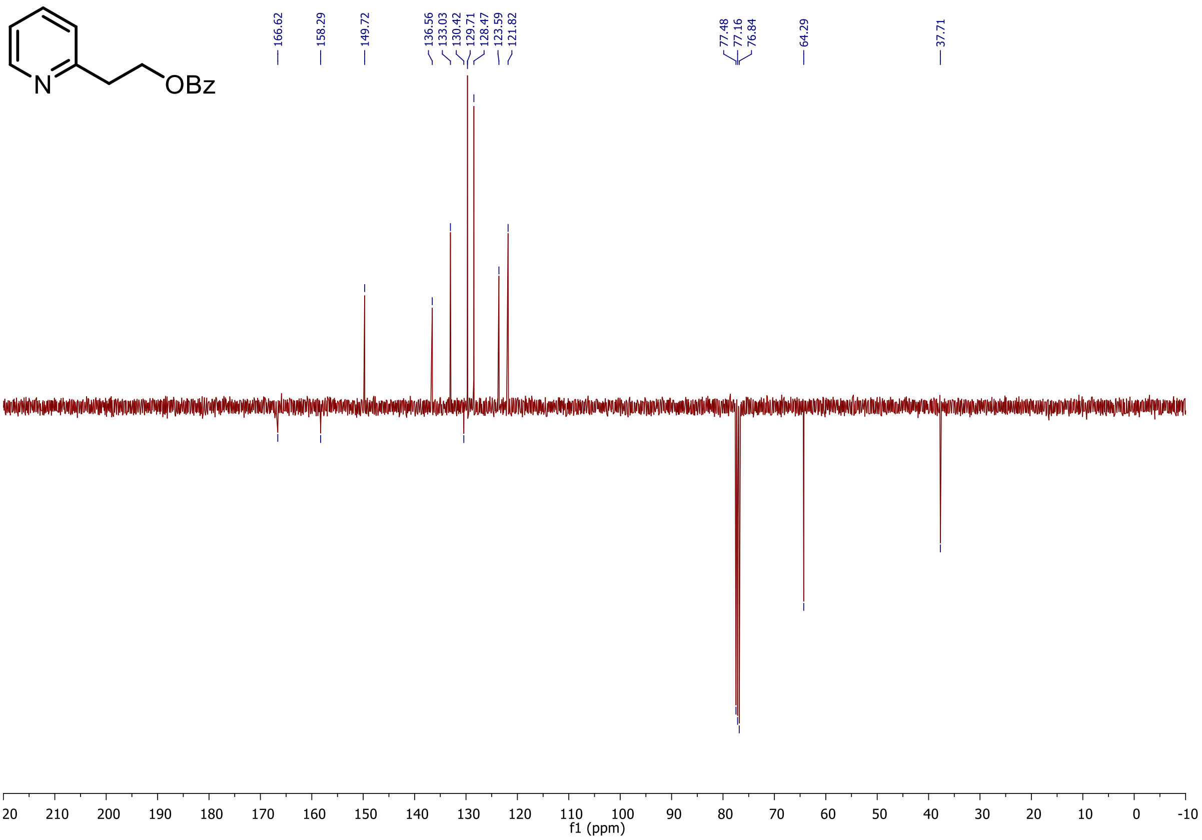

Figure S148. 5h, ^{1}H NMR, 500 MHz, $CDCl_3$

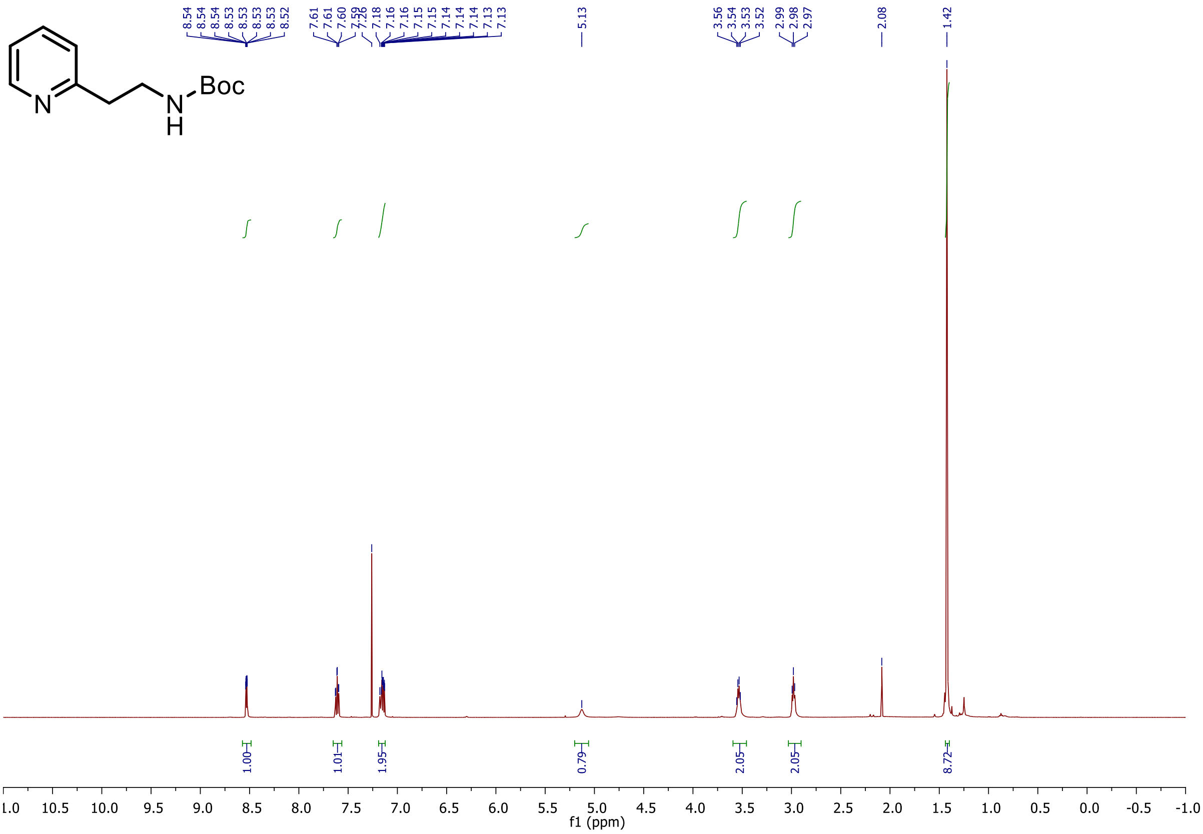

Figure S149. 5h, $^{13}C\ \{^{1}H\}$ NMR, 126 MHz, $CDCl_3$

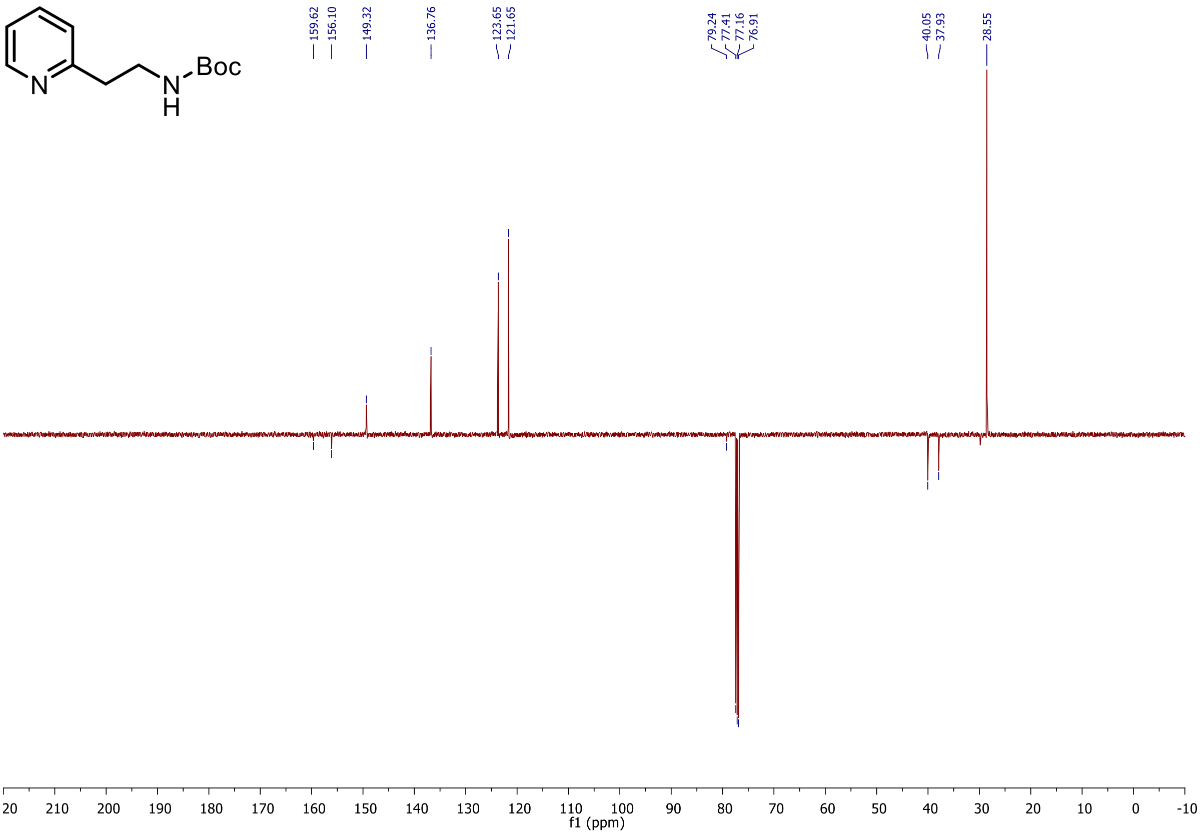

Figure S150. 5i, ^{1}H NMR, 500 MHz, $CDCl_3$

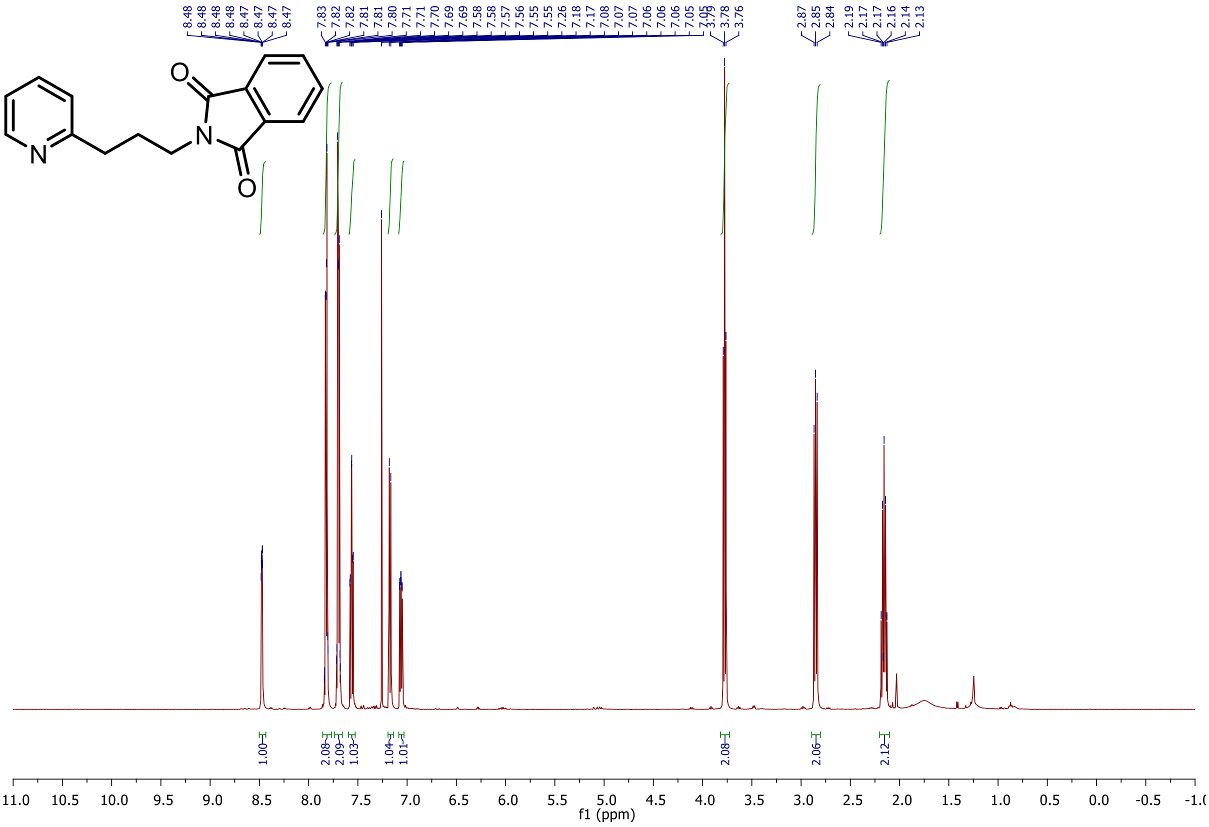

Figure S151. 5i, $^{13}C\{^1H\}$ NMR, 126 MHz, $CDCl_3$

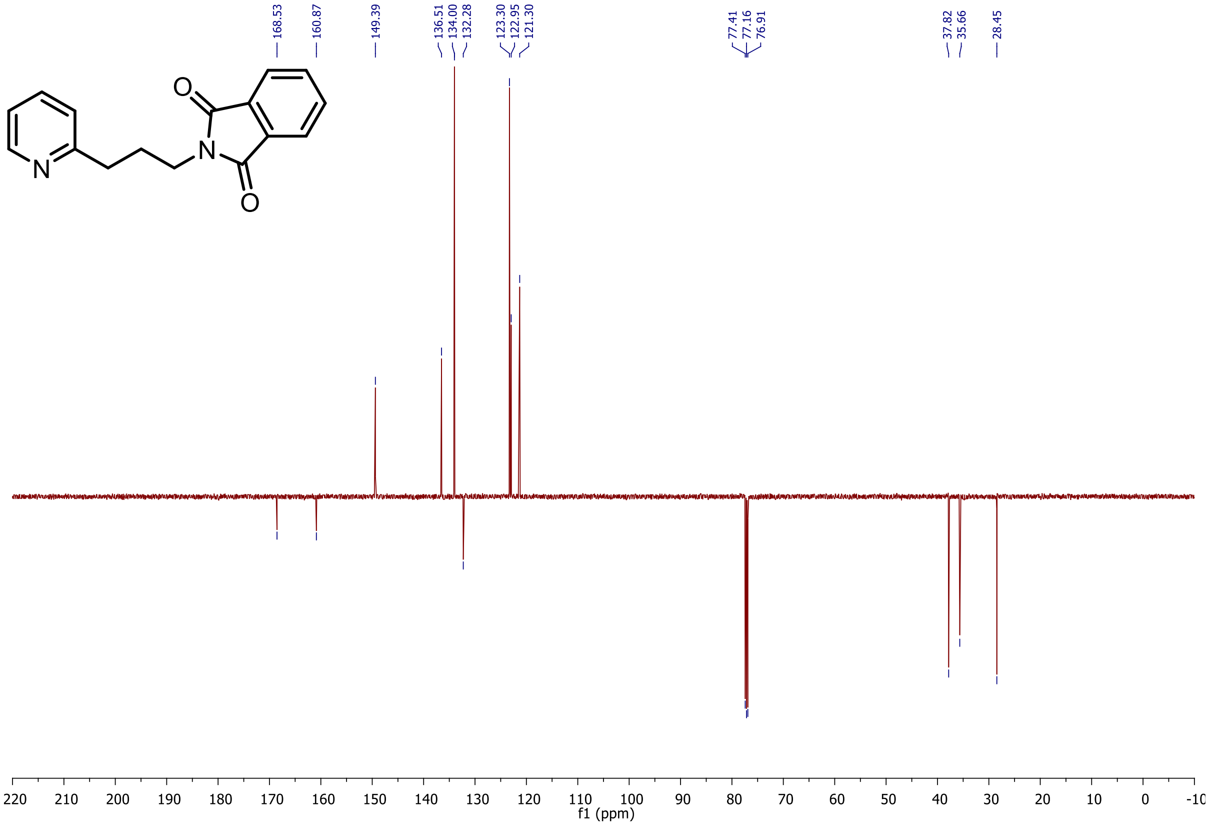

Figure S152. 5j, ^{1}H NMR, 500 MHz, $CDCl_3$

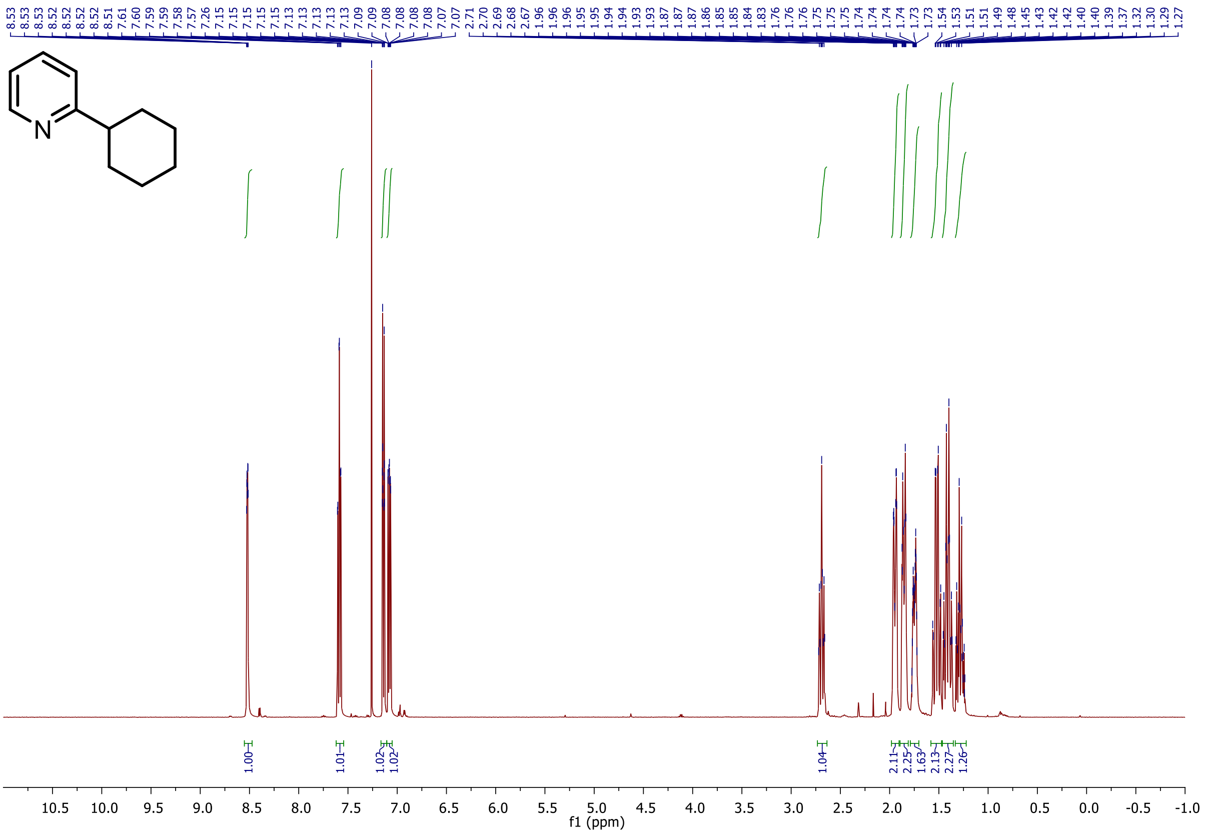

Figure S153. 5j, $^{13}C\{^{1}H\}$ NMR, 126 MHz, $CDCl_3$

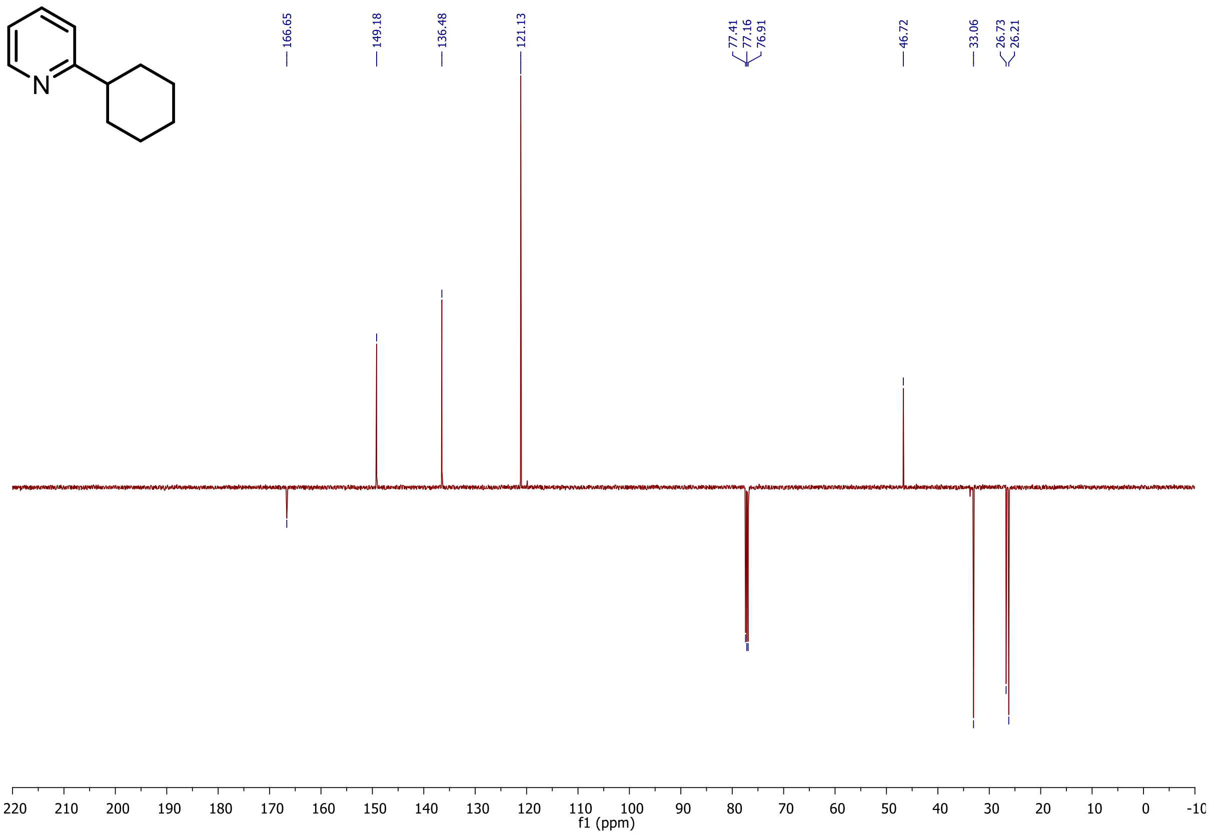

Figure S154. 5k, ^{1}H NMR, 500 MHz, $CDCl_3$

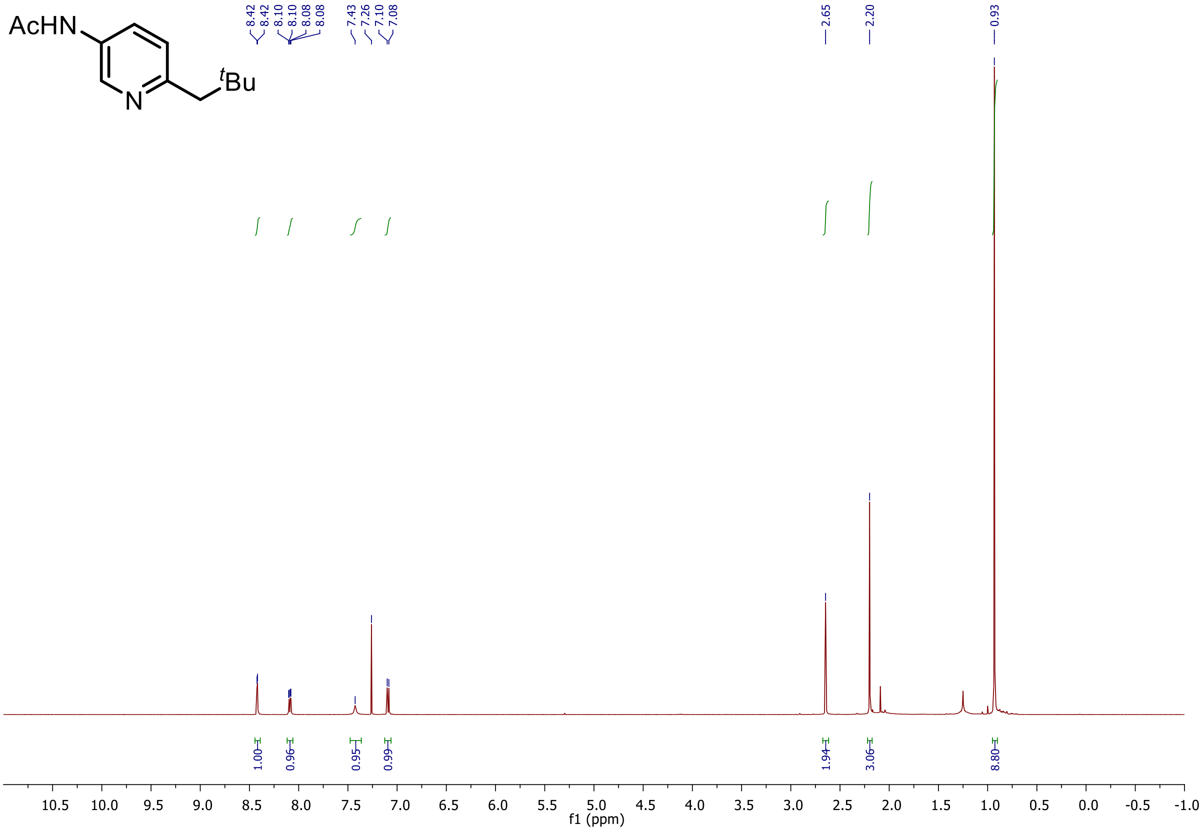

Figure S155. 5k, $^{13}C\{^1H\}$ NMR, 126 MHz, $CDCl_3$

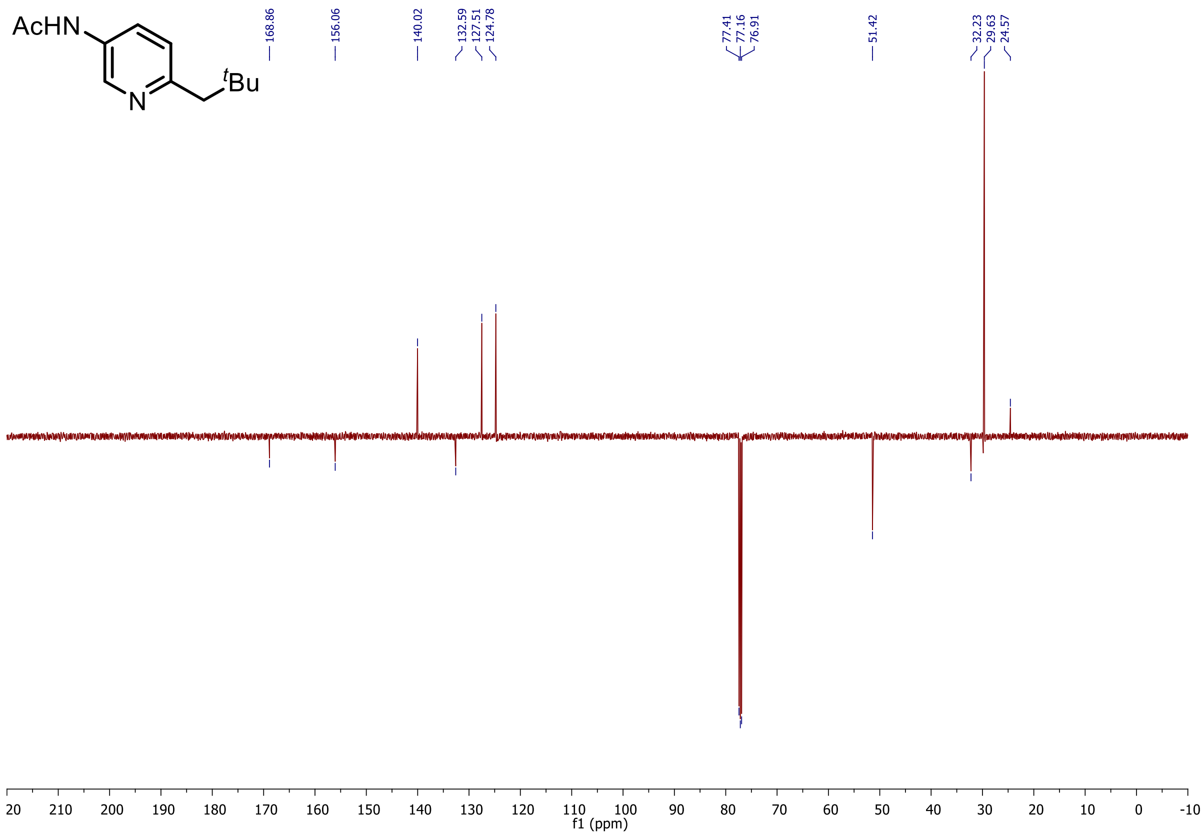

Figure S156. 5t, ^1H NMR, 500 MHz, $CDCl_3$

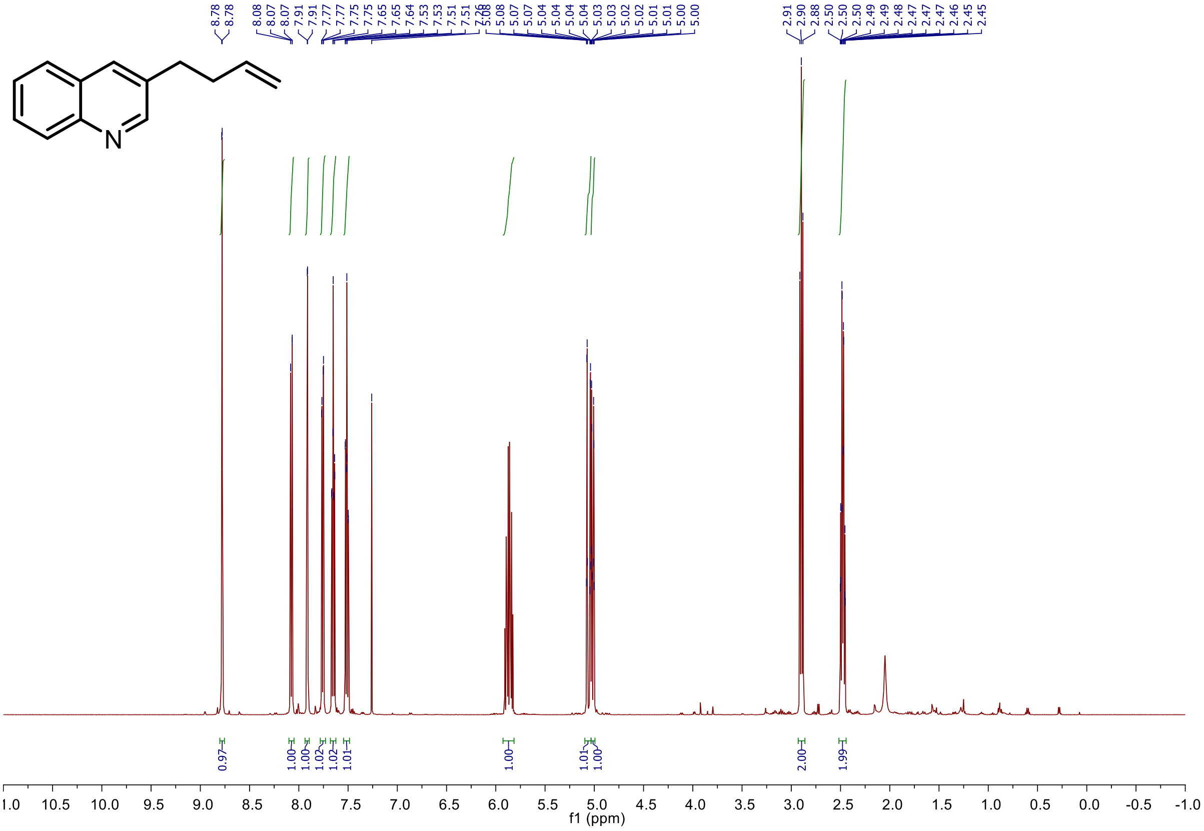

Figure S157. 5t, $^{13}C \{^{1}H\}$ NMR, 126 MHz, $CDCl_3$

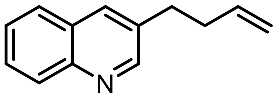

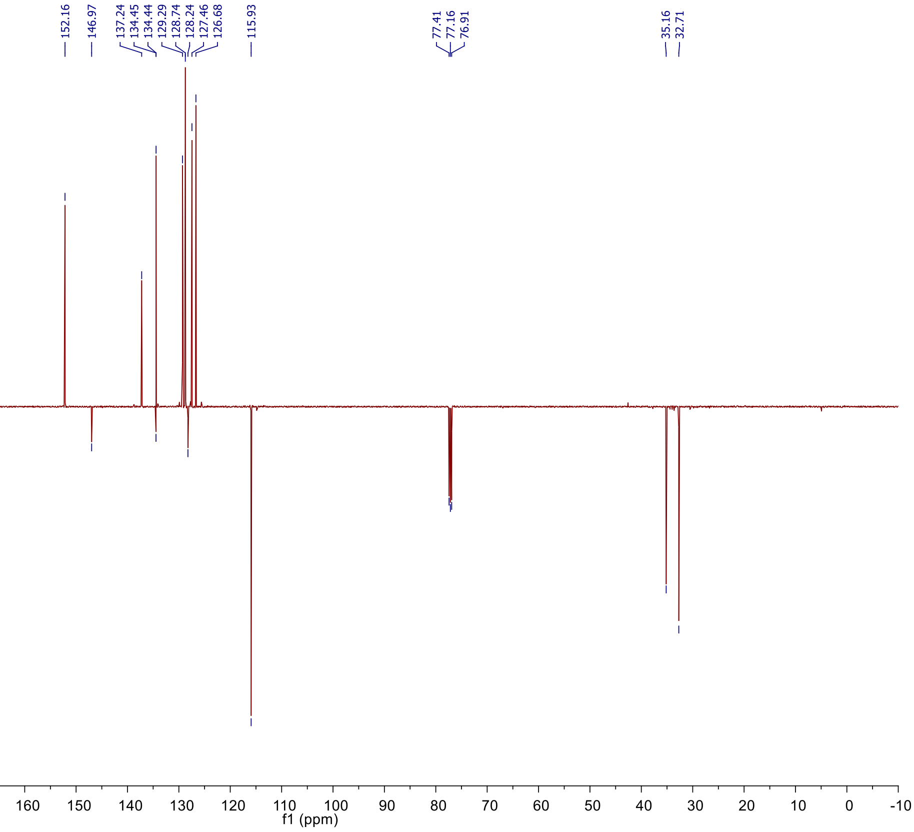

5. References

1. Meng, G.; Szostak, M. Palladium-catalyzed Suzuki–Miyaura coupling of amides by carbon–nitrogen cleavage: general strategy for amide N–C bond activation. *Org. Biomol. Chem.* **2016**, *14*, 5690–5707.

2. Bie, F.; Liu, X.; Shi, Y.; Cao, H.; Han, Y.; Szostak, M.; Liu, C. Rh-Catalyzed Base-Free Decarbonylative Borylation of Twisted Amides. *J. Org. Chem.* **2020**, *85*, 15676–15685.

3. Liu, Y.; Achtenhagen, M.; Liu, R.; Szostak, M. Transamidation of *N*-acyl-glutarimides with amines. *Org. Biomol. Chem.* **2018**, *16*, 1322–1329.

4. Bie, F.; Liu, X.; Cao, H.; Shi, Y.; Zhou, T.; Szostak, M.; Liu, C. Pd-Catalyzed Double-Decarbonylative Aryl Sulfide Synthesis through Aryl Exchange between Amides and Thioesters. *Org. Lett.* **2021**, *23*, 8098–8103.

5. Govindan, K.; Lin, W.-Y. Ring Opening/Site Selective Cleavage in N-Acyl Glutarimide to Synthesize Primary Amides. *Org. Lett.* **2021**, *23*, 1600–1605.

6. Zhuo, J.; Zhang, Y.; Li, Z.; Li, C. Nickel-Catalyzed Direct Acylation of Aryl and Alkyl Bromides with Acylimidazoles. *ACS Catal.* **2020**, *10*, 3895–3903.

7. Kerr, W. J.; Morrison, A. J.; Pazicky, M.; Weber, T. Modified Shapiro Reactions with Bismesitylmagnesium As an Efficient Base Reagent. *Org. Lett.* **2012**, *14*, 2250–2253.

8. Li, Y.; Li, Y.; Peng, L.; Wu, D.; Zhu, L.; Yin, G. Nickel-catalyzed migratory alkyl–alkyl cross-coupling reaction. *Chem. Sci.* **2020**, *11*, 10461–10464.

9. Wang, D.; Zhang, Z. Palladium-Catalyzed Cross-Coupling Reactions of Carboxylic Anhydrides with Organozinc Reagents. *Org. Lett.* **2003**, *5*, 4645–4648.

10. Zhang, P.; “Chip” Le, C.; MacMillan, D. W. C. Silyl Radical Activation of Alkyl Halides in Metallaphotoredox Catalysis: A Unique Pathway for Cross-Electrophile Coupling. *J. Am. Chem. Soc.* **2016**, *138*, 8084–8087.

11. Jafarpour, F.; Rajai-Daryasarei, S.; Gohari, M. H. Cascade cyclization versus chemoselective reduction: a solvent-controlled product divergence. *Org. Chem. Front.* **2020**, *7*, 3374–3381.

12. Kohler, D. G.; Gockel, S. N.; Kennemur, J. L.; Waller, P. J.; Hull, K. L. Palladium-catalysed *anti*-Markovnikov selective oxidative amination. *Nat. Chem.* **2018**, *10*, 333–340.

13. Blay, G.; Fernández, I.; Monje, B.; Pedro, J. R. Nucleophilic Benzoylation Using a Mandelic Acid Dioxolanone as a Synthetic Equivalent of the Benzoyl Carbanion. Oxidative Decarboxylation of α-Hydroxyacids. *Molecules* **2004**, *9*, 365–372.

14. Stevanović, D.; Pejović, A.; Damljanović, I. S.; Vukićević, M. D.; Dobrikov, G.; Dimitrov, V.; Denić, M. S.; Radulović, N. S.; Vukićević, R. D. Electrochemical Phenylselenoetherification as a Key Step in the Synthesis of (±)-Curcumene Ether. *Helvetica Chimica Acta* **2013**, *96*, 1103–1110.

15. Nambu, H.; Hata, K.; Matsugi, M.; Kita, Y. Efficient Synthesis of Thioesters and Amides from Aldehydes by Using an Intermolecular Radical Reaction in Water. *Chem. Eur J.* **2005**, *11*, 719–727.

16. Fialho, D. M.; Etemadi-Davan, E.; Langner, O. C.; Takale, B. S.; Gadakh, A.; Sambasivam, G.; Lipshutz, B. H. Copper-Catalyzed Asymmetric Reductions of Aryl/Heteroaryl Ketones under Mild Aqueous Micellar Conditions. *Org. Lett.* **2021**, *23*, 3282–3286.

17. Yang, X.-H.; Xie, J.-H.; Liu, W.-P.; Zhou, Q.-L. Catalytic Asymmetric Hydrogenation of δ-Ketoesters: Highly Efficient Approach to Chiral 1,5-Diols. *Angew. Chem. Int. Ed.* **2013**, *52*, 7833–7836.

18. Guijarro, D.; Pablo, Ó.; Yus, M. Synthesis of γ-, δ-, and ε-Lactams by Asymmetric Transfer Hydrogenation of *N*-(*tert*-Butylsulfinyl)iminoesters. *J. Org. Chem.* **2013**, *78*, 3647–3654.

19. Shi, R.; Hu, X. From Alkyl Halides to Ketones: Nickel-Catalyzed Reductive Carbonylation Utilizing Ethyl Chloroformate as the Carbonyl Source. *Angew. Chem. Int. Ed.* **2019**, *58*, 7454–7458.

20. Lee, G. S.; Won, J.; Choi, S.; Baik, M.-H.; Hong, S. H. Synergistic Activation of Amides and Hydrocarbons for Direct C(sp^3)–H Acylation Enabled by Metallaphotoredox Catalysis. *Angew. Chem. Int. Ed.* **2020**, *59*, 16933–16942.

21. Hunt, T.; Parsons, A. F.; Pratt, R. Radion Reactions To Form Vinylphosphonothioates. *Synlett* **2005**, *19*, 2978–2980.

22. Das, M.; Vu, M. D.; Zhang, Q.; Liu, X.-W. Metal-free visible light photoredox enables generation of carbyne equivalents *via* phosphonium ylide C–H activation. Chem. Sci., **2019**, *10*, 1687–1691.

23. Zhang, L.; Si, X.; Yang, Y.; Witzel, S.; Sekine, K.; Rudolph, M.; Rominger, F.; Hashmi, A. S. K. Reductive C–C Coupling by Desulfurizing Gold-Catalyzed Photoreactions. *ACS Catal.* **2019**, *9*, 6118–6123.

24. Nicolai, S.; Sedigh-Zadeh, R.; Waser, J. Pd(0)-Catalyzed Alkene Oxy- and Aminoalkynylation with Aliphatic Bromoacetylenes. *J. Org. Chem.* **2013**, *78*, 3783–3801.

25. Hansen, E. C.; Pedro, D. J.; Wotal, A. C.; Gower, N. J.; Nelson, J. D.; Caron, S.; Weix, D. J. New ligands for nickel catalysis from diverse pharmaceutical heterocycle libraries. *Nat. Chem.* **2016**, *8*, 1126–1130.

26. Hansen, E. C.; Li, C.; Yang, S.; Pedro, D.; Weix, D. J. Coupling of Challenging Heteroaryl Halides with Alkyl Halides via Nickel-Catalyzed Cross-Electrophile Coupling. *J. Org. Chem.* **2017**, *82*, 7085–7092.

27. Everson, D. A.; Buonomo, J. A.; Weix, D. J. Nickel-Catalyzed Cross-Electrophile Coupling of 2-Chloropyridines with Alkyl Bromides. *Synlett* **2014**, *25*, 233–238.

28. Vechorkin, O.; Proust, V.; Hu, X. Functional Group Tolerant Kumada−Corriu−Tamao Coupling of Nonactivated Alkyl Halides with Aryl and Heteroaryl Nucleophiles: Catalysis by a Nickel Pincer Complex Permits the Coupling of Functionalized Grignard Reagents. *J. Am. Chem. Soc.* **2009**, *131*, 9756–9766.

29. Su, Y. L.; Liu, G. X.; Liu, J. W.; Tram, L.; Qiu, H.; Doyle, M. P. Radical-Mediated Strategies for the Functionalization of Alkenes with Diazo Compounds. *J. Am. Chem. Soc.* **2020**, *142*, 13846–13855.

30. Dilauro, G.; Azzollini, C. S.; Vitale, P.; Salomone, A.; Perna, F. M.; Capriati, V. Scalable Negishi Coupling between Organozinc Compounds and (Hetero)Aryl Bromides under Aerobic Conditions when using Bulk Water or Deep Eutectic Solvents with no Additional Ligands. *Angew. Chem. Int. Ed.* **2021**, *60*, 10632–10636.
